# Substitution of animal-based with plant-based foods on cardiometabolic health and all-cause mortality: a systematic review and meta-analysis of prospective studies

**DOI:** 10.1186/s12916-023-03093-1

**Published:** 2023-11-16

**Authors:** Manuela Neuenschwander, Julia Stadelmaier, Julian Eble, Kathrin Grummich, Edyta Szczerba, Eva Kiesswetter, Sabrina Schlesinger, Lukas Schwingshackl

**Affiliations:** 1 Institute for Biometrics and Epidemiology, German Diabetes Center, Leibniz Center for Diabetes Research at Heinrich Heine University Düsseldorf, Auf’m Hennekamp 65, 40225 Düsseldorf, Germany; 2 German Center for Diabetes Research (DZD), Partner Düsseldorf, Düsseldorf, Germany; 3 Institute for Evidence in Medicine, Medical Center - University of Freiburg, Faculty of Medicine, University of Freiburg, Freiburg, Germany

**Keywords:** Diet, Cardiovascular disease, Type 2 diabetes, Mortality, Substitution, Plant-based food, Animal-based food

## Abstract

**Background:**

There is growing evidence that substituting animal-based with plant-based foods is associated with a lower risk of cardiovascular diseases (CVD), type 2 diabetes (T2D), and all-cause mortality. Our aim was to summarize and evaluate the evidence for the substitution of any animal-based foods with plant-based foods on cardiometabolic health and all-cause mortality in a systematic review and meta-analysis.

**Methods:**

We systematically searched MEDLINE, Embase, and Web of Science to March 2023 for prospective studies investigating the substitution of animal-based with plant-based foods on CVD, T2D, and all-cause mortality. We calculated summary hazard ratios (SHRs) and 95% confidence intervals (95% CI) using random-effects meta-analyses. We assessed the certainty of evidence (CoE) using the GRADE approach.

**Results:**

In total, 37 publications based on 24 cohorts were included. There was moderate CoE for a lower risk of CVD when substituting processed meat with nuts [SHR (95% CI): 0.73 (0.59, 0.91), *n* = 8 cohorts], legumes [0.77 (0.68, 0.87), *n* = 8], and whole grains [0.64 (0.54, 0.75), *n* = 7], as well as eggs with nuts [0.83 (0.78, 0.89), *n* = 8] and butter with olive oil [0.96 (0.95, 0.98), *n* = 3]. Furthermore, we found moderate CoE for an inverse association with T2D incidence when substituting red meat with whole grains/cereals [0.90 (0.84, 0.96), *n* = 6] and red meat or processed meat with nuts [0.92 (0.90, 0.94), *n* = 6 or 0.78 (0.69, 0.88), *n* = 6], as well as for replacing poultry with whole grains [0.87 (0.83, 0.90), *n* = 2] and eggs with nuts or whole grains [0.82 (0.79, 0.86), *n* = 2 or 0.79 (0.76, 0.83), *n* = 2]. Moreover, replacing red meat for nuts [0.93 (0.91, 0.95), *n* = 9] and whole grains [0.96 (0.95, 0.98), *n* = 3], processed meat with nuts [0.79 (0.71, 0.88), *n* = 9] and legumes [0.91 (0.85, 0.98), *n* = 9], dairy with nuts [0.94 (0.91, 0.97), *n* = 3], and eggs with nuts [0.85 (0.82, 0.89), *n* = 8] and legumes [0.90 (0.89, 0.91), *n* = 7] was associated with a reduced risk of all-cause mortality.

**Conclusions:**

Our findings indicate that a shift from animal-based (e.g., red and processed meat, eggs, dairy, poultry, butter) to plant-based (e.g., nuts, legumes, whole grains, olive oil) foods is beneficially associated with cardiometabolic health and all-cause mortality.

**Supplementary Information:**

The online version contains supplementary material available at 10.1186/s12916-023-03093-1.

## Background

The current food system has been shown to be detrimental to planetary health by depleting Earth’s resources and contributing to climate change, thus decreasing the quality and sufficiency of food [1, 2]. Furthermore, non-communicable diseases related to dietary choices such as cardiovascular diseases (CVD) and type 2 diabetes (T2D) highly contribute to deaths worldwide [3]. Therefore, the food system and current dietary habits negatively impact both planetary and human health [2]. Consequently, one of the approaches to address these problems is to change dietary habits [2, 4]. Plant-based diets are the focus of recent studies suggesting that increased intake of plant-based foods is not only beneficial for planetary health [2, 4], but also reduces the risk of T2D, CVD, and premature death [5, 6]. In contrast, the production and consumption of animal-based foods, especially red and processed meat, pose a high burden on the environment [2] and have been linked to increased risk for CVD, T2D, and mortality [7–9]. Such findings contribute to recommendations to reduce the intake of red and processed meat. However, to keep energy intake constant, other foods need to be consumed instead and the association with disease risks may depend on this substitution [10]. A promising option is the replacement of animal-based with plant-based foods. In this context, epidemiological studies investigated the substitution of animal-based foods with other protein sources and indicated that such a replacement is associated with reduced risk of CVD, T2D, and mortality [11–14]. A recent systematic review and meta-analysis found a lower risk for coronary heart disease (CHD) when replacing red meat with poultry, dairy, eggs, nuts, or legumes and a lower risk of all-cause mortality when substituting red meat with fish/seafood, poultry, eggs, or nuts [15]. However, the authors did not investigate further cardiometabolic endpoints, including CVD or T2D. Furthermore, the substitution of any animal based-foods with only plant-based foods has yet to be investigated in a systematic review and meta-analysis. Therefore, to clarify the strength and certainty of the overall evidence on this topic, there is an urgent need for a systematic review and meta-analysis examining the association between substituting animal-based with purely plant-based foods and cardiometabolic health outcomes, including CVD, stroke, myocardial infarction (MI), T2D, and mortality. Thus, it was our aim to summarize and evaluate the meta-evidence on the substitution of animal-based with plant-based foods regarding cardiometabolic health outcomes and all-cause mortality.

## Methods

Our protocol was pre-registered at PROSPERO (https://www.crd.york.ac.uk/prospero/display_record.php?ID=CRD42022302982). We followed the Preferred Reporting Items for Systematic Reviews and Meta-Analyses (PRISMA) statement 2020 [16]. All steps were conducted by at least two investigators, independently. Disagreements were solved by consensus.

### Deviations from the protocol

There were no deviations in methods, but we added all-cause mortality to our outcomes since cardiovascular disease and type 2 diabetes highly contribute to deaths worldwide [3].

### Search strategy and selection criteria

A systematic literature search was conducted on MEDLINE via Ovid, Embase via Ovid, and Web of Science (Clarivate) up to December 2021 using predefined search terms (Additional file 1: Search terms) applying no restrictions or filters. Additionally, we screened the reference lists of relevant publications for further studies and conducted a PubMed Similar Articles search. Moreover, an update of the literature search was performed in March 2023.

Studies were included if (1) substitution analyses of any animal-based foods (including red and processed meat, poultry, fish, shellfish, eggs, dairy, and dairy products) with any plant-based foods (including legumes, nuts, whole and refined grains, fruit, vegetables, soy, seeds, and oils) that specified the substitution were conducted; (2) cardiometabolic health outcomes, including CVD mortality; incidence of CVD, CHD, stroke, MI, and T2D; and all-cause mortality, were investigated; (3) they were prospective observational studies; and (4) the study was conducted among the general healthy population. The detailed exclusion criteria are displayed in Additional file 2: Table S1. If multiple publications reported results regarding the same association based on data from the same cohort, the study with more cases and/or a longer follow-up was included in order to avoid duplication. Thus, each cohort is only included once in each respective meta-analysis. In addition to the single outcomes, we investigated composite outcomes, such as total CVD, including CVD mortality, CVD incidence, CHD incidence, MI, and stroke, as well as total diabetes, including T2D incidence and mortality.

Relevant study characteristics were extracted (Additional file 2: Table S2).

### Risk of bias and certainty of evidence assessment

A risk of bias assessment for each study was conducted using the Risk of Bias in Non-randomized Studies of Interventions (ROBINS-I) tool [17]. It includes seven domains of bias due to (1) confounding, (2) selection of participants, (3) exposure assessment, (4) misclassification of exposure during follow-up, (5) missing data, (6) measurement of the outcome, and (7) selective reporting of the results (Additional file 2: Table S3).

The certainty of evidence for each association was evaluated using the updated Grading of Recommendations, Assessment, Development, and Evaluations (GRADE) approach [18]. In this updated approach, the initial certainty of evidence level is “high” for observational studies. However, the certainty of evidence is downgraded (up to three levels) unless the study design reduces confounding, selection, and information bias, as evaluated by ROBINS-I. Additionally, indication for inconsistency (as measured by the similarity of the point estimates, overlap of 95% confidence intervals, and statistical tests, such as *I* ^2^), indirectness (e.g., substantial differences in population or exposure), imprecision (wide 95% confidence interval and/or small number of events), and publication bias can lead to a downgrading, while large effects (SHR < 0.5 or > 2.0) and a dose–response gradient can lead to an upgrading [18, 19]. High and moderate certainty of evidence mean that it is very likely or probable that the true effect lies close to the estimated effect. Our confidence in the result is limited, if the certainty of evidence is rated as low or very low [19].

### Data analysis

For each substitution meta-analysis, we calculated summary hazard ratios (SHR) and 95% confidence intervals (95% CIs) using a random-effects model by DerSimonian and Laird, taking into account both within- and between-study variability [20, 21].

To ensure comparability of the results, we converted hazard ratios (HRs) and 95% CIs for standardized food portions, as previously applied, for the substituted foods [7–9]. To ensure that the adaption of the HRs and 95% CIs was not only correct for the substituted food but also for the replacement, we recalculated the portion size of the substitute according to the conversion of the replaced food. For example, if a study substituted 100 g/day of red meat by 30 g nuts/day, we calculated the HR and 95% CI for a substituted portion of 50 g/day of red meat by 15 g of nuts.

Moreover, we calculated *I* ^2^ and *tau*^2^ (*τ* ^2^) as measures of inconsistency and between-study variability, respectively, as well as 95% prediction intervals (95% PIs), which show the range in which the true effect of future studies will lie with 95% certainty [22, 23].

For some associations, single publications provided only pooled risk estimates of multiple cohorts and did not show risk estimates for the single studies separately. When we did not identify further relevant studies, we extracted the pooled risk estimates from these publications [11, 14, 24–29]. Furthermore, three studies reported risk estimates based on changes over time rather than the baseline consumption or a cumulative average [11, 29, 30]. Those risk estimates were not pooled and reported separately.

We planned to conduct sensitivity analyses, by excluding studies with a high risk of bias as well as leaving out one study at a time, and subgroup analyses by sex, region, dietary assessment method, or level of adjustment. However, due to the low number of studies, only the sensitivity analyses excluding one study at a time could be conducted.

Publication bias and small study effects were assessed using funnel plots and Egger’s test [31, 32], if at least ten studies were available [33]. Potential publication bias was indicated by the asymmetry of the funnel plot and a *p*-value of < 0.1 for Egger’s test [32].

All statistical analyses were conducted using the STATA version 14.1.

## Results

**Fig. 1:**
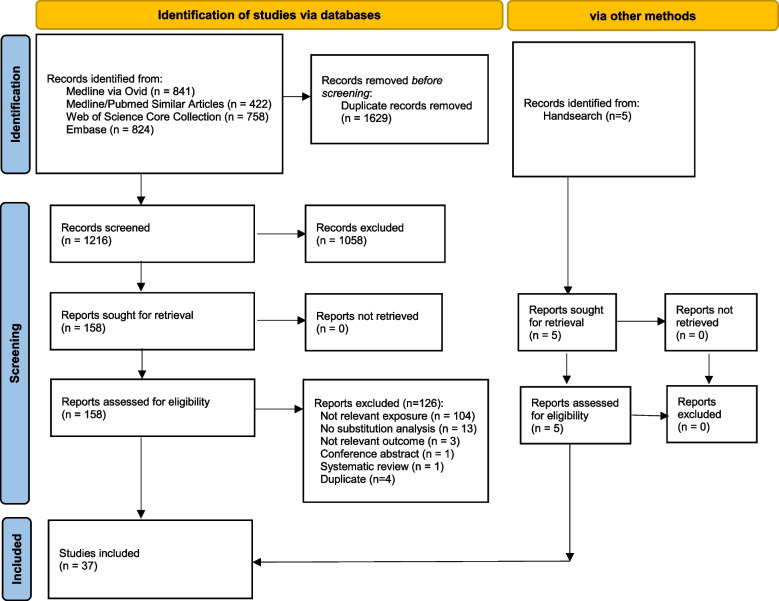
Study selection

Of the 1216 studies identified in our search after the removal of duplicates, 158 fulltext articles were considered for inclusion. Out of these, 126 publications were excluded, leaving 32 studies to be included in our analyses. A list of the excluded studies with reasons is provided in Additional file 2: Table S4 [12, 34–157]. Additionally, five relevant studies were identified via hand search. Thus, 37 studies were included in our final meta-analyses (Fig. 1). All identified publications were prospective cohort studies. No randomized controlled trials (RCTs) analyzed as observational studies were included.

Twenty-two publications including 12 cohorts were conducted in the USA [11, 13, 14, 24–29, 138, 154, 158–168], ten publications including seven cohorts in Europe [30, 156, 157, 169–175], and four studies including three cohorts in Asia [155, 176–178]. One publication included one US and one European cohort [179]. In all cohorts except for three (using consecutive 24-h recalls [173, 177, 178]), diet was assessed using validated food frequency questionnaires. The mean follow-up duration was 19 years. All cohorts except for five included both men and women. One cohort (Health Professionals’ Follow-up Study (HPFS)) only included men [11, 13, 24–29, 138, 154, 162, 163, 166] and four cohorts (Nurses’ Health Study (NHS), NHS II, Women’s Health Initiative (WHI), and Black Women’s Health Study (BWHS)) only women [11, 24–29, 138, 154, 158, 159, 161–166, 179] (Additional file 2: Table S2).

All publications were judged as moderate risk of bias, except for one [169] (Additional file 3: Fig. S1), which was judged as serious risk of bias due to insufficient adjustment of confounders (Additional file 3: Fig. S2). No study was judged as low risk of bias due to the possibility of residual confounding in observational studies and the possibility of measurement error in the dietary assessment.

### Total CVD

**Fig. 2:**
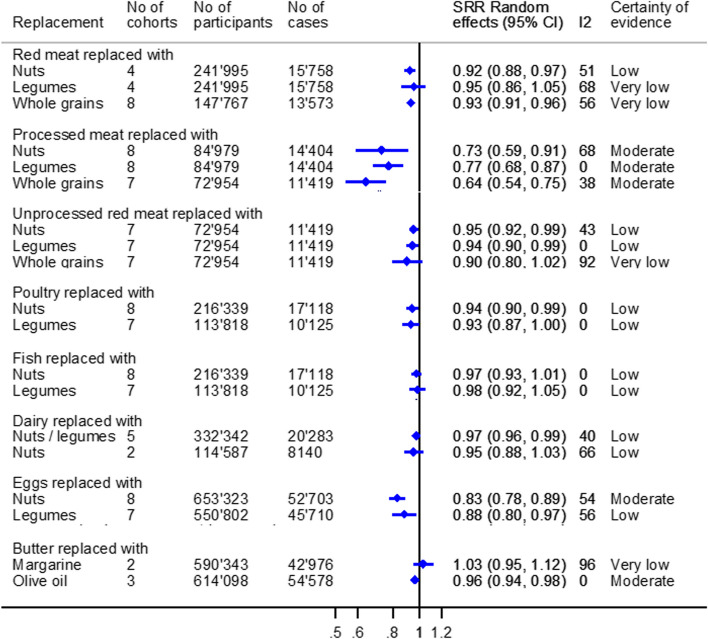
Substitution meta-analyses replacing animal-based foods with plant-based foods regarding total CVD (including incidence of CVD, CHD, MI, and CVD mortality). Portion sizes: red meat/(un)processed meat/poultry/fish/dairy: 50 g/day; eggs: 1 egg/day; nuts: 10–50 g/day; legumes: 13–50 g/day; whole grains: 10–30 g/day; butter: 5 g/day; olive oil: 5 g/day; and margarine: 5 g/day

The meta-analyses on total CVD are shown in Fig. 2 and Additional file 3: Fig. S3.

We observed an association with a lower incidence of total CVD for the substitution of processed meat (50 g/day) with nuts (28–50 g/day) [SHR (95% CI): 0.73 (0.59, 0.91), *I* ^2^ = 68%, *n*_cohorts_ = 8], legumes (50 g/day) [SHR (95% CI): 0.77 (0.68, 0.87) *I* ^2^ = 0%, *n*_cohorts_ = 8], or whole grains (30 g/day) [SHR (95% CI): 0.64 (0.54,0.75), *I* ^2^ = 38%, *n*_cohorts_ = 7]; one egg/day with nuts (25–28 g/day) [SHR (95% CI): 0.83 (0.78, 0.89), *I* ^2^ = 54%, *n*_cohorts_ = 8]; and butter (5 g/day) with olive oil (5 g/day) [SHR (95% CI): 0.96 (0.95, 0.98), *I* ^2^ = 0%, *n*_cohorts_ = 3] with moderate certainty of evidence. There was also an indication that replacing red meat with nuts, unprocessed red meat with nuts or legumes, poultry with nuts, and eggs with legumes was associated with a lower risk of total CVD; however, the certainty of evidence for these associations was low. There were no clear associations for the other meta-analyses, and the certainty of evidence was rated as low and very low (Additional file 2: Table S5).

### Single CVD outcomes

**Fig. 3:**
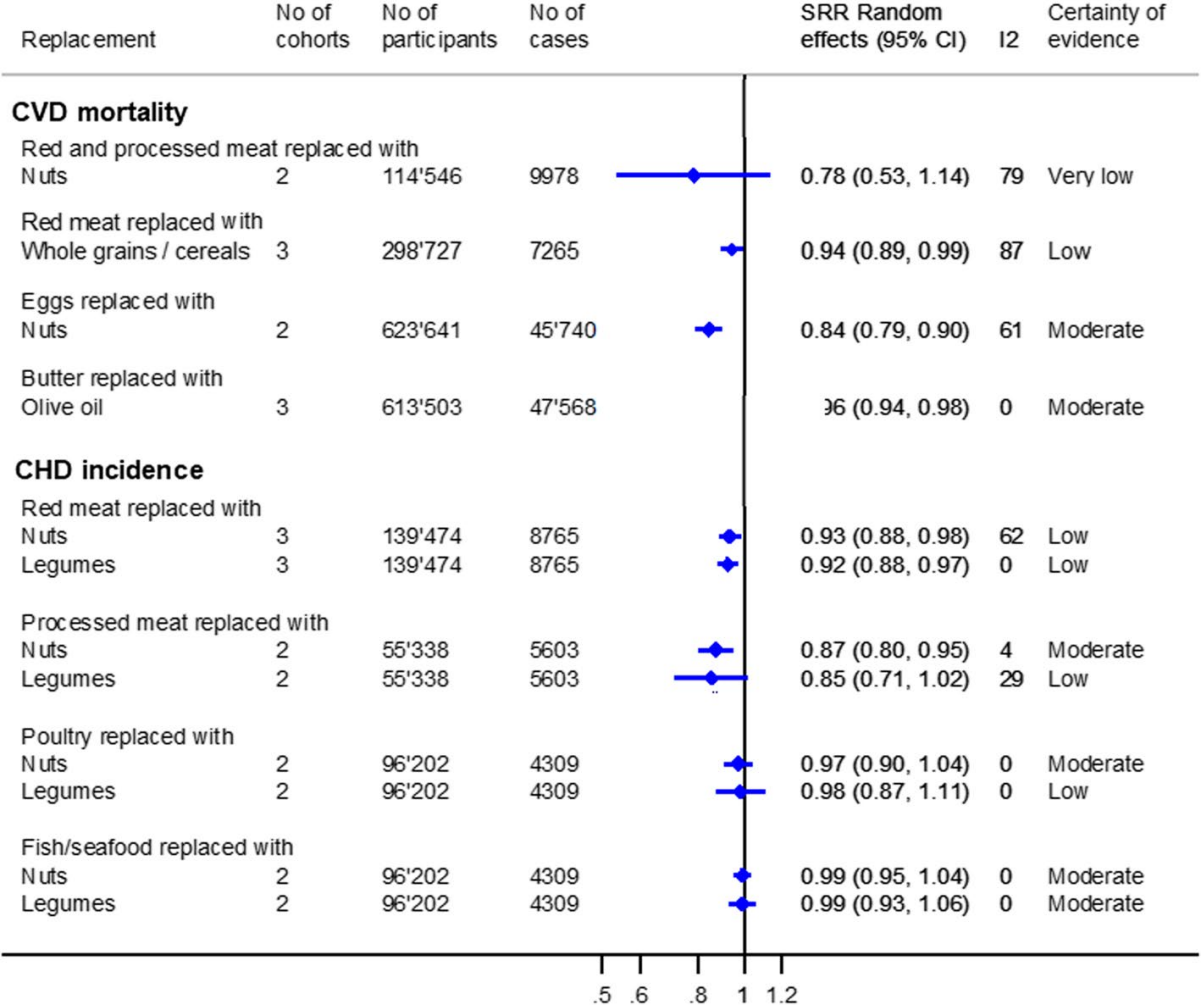
Substitution meta-analyses replacing animal-based foods with plant-based foods regarding single CVD outcomes. Portion sizes: red meat/processed meat/poultry/fish and seafood: 50 g/day; eggs: 1 egg/day; nuts: 10–28 g/day; legumes: 12.5–50 g/day; whole grains/cereals: 15–30 g/day; butter: 5 g/day; and olive oil: 5 g/day

Figure 3 and Additional file 3: Figs. S4 and S5 show the findings on CVD mortality and CHD incidence.

Regarding CVD mortality, there was moderate certainty of evidence that replacing one egg/day with nuts (25 g/day) was associated with lower CVD mortality [SHR (95% CI): 0.84 (0.79, 0.90), *I* ^2^ = 61%, *n*_cohorts_ = 2], as did replacing butter (5 g/day) with olive oil (5 g/day) [SHR (95% CI): 0.96 (0.94, 0.98), *I* ^2^ = 0%, *n*_cohorts_ = 3]. The other associations were rated as low and very low certainty of evidence (Additional file 2: Table S6).

There was moderate certainty of evidence that substituting processed meat (50 g/day) with nuts (28 g/day) was associated with a lower CHD incidence [SHR (95% CI): 0.87 (0.80, 0.95), *I* ^2^ = 4%, *n*_cohorts_ = 2] and that replacing poultry (50 g/day) with nuts (11–14 g/day), as well as fish/seafood (50 g/day) with nuts (7 g/day) or legumes (12.5 g/day), was not associated with CHD incidence. There was an indication that replacing red meat with nuts or legumes, as well as processed meat with legumes, was associated with a lower CHD risk, but the certainty of evidence for these associations was low (Additional file 2: Table S7).

### Type 2 diabetes

**Fig. 4:**
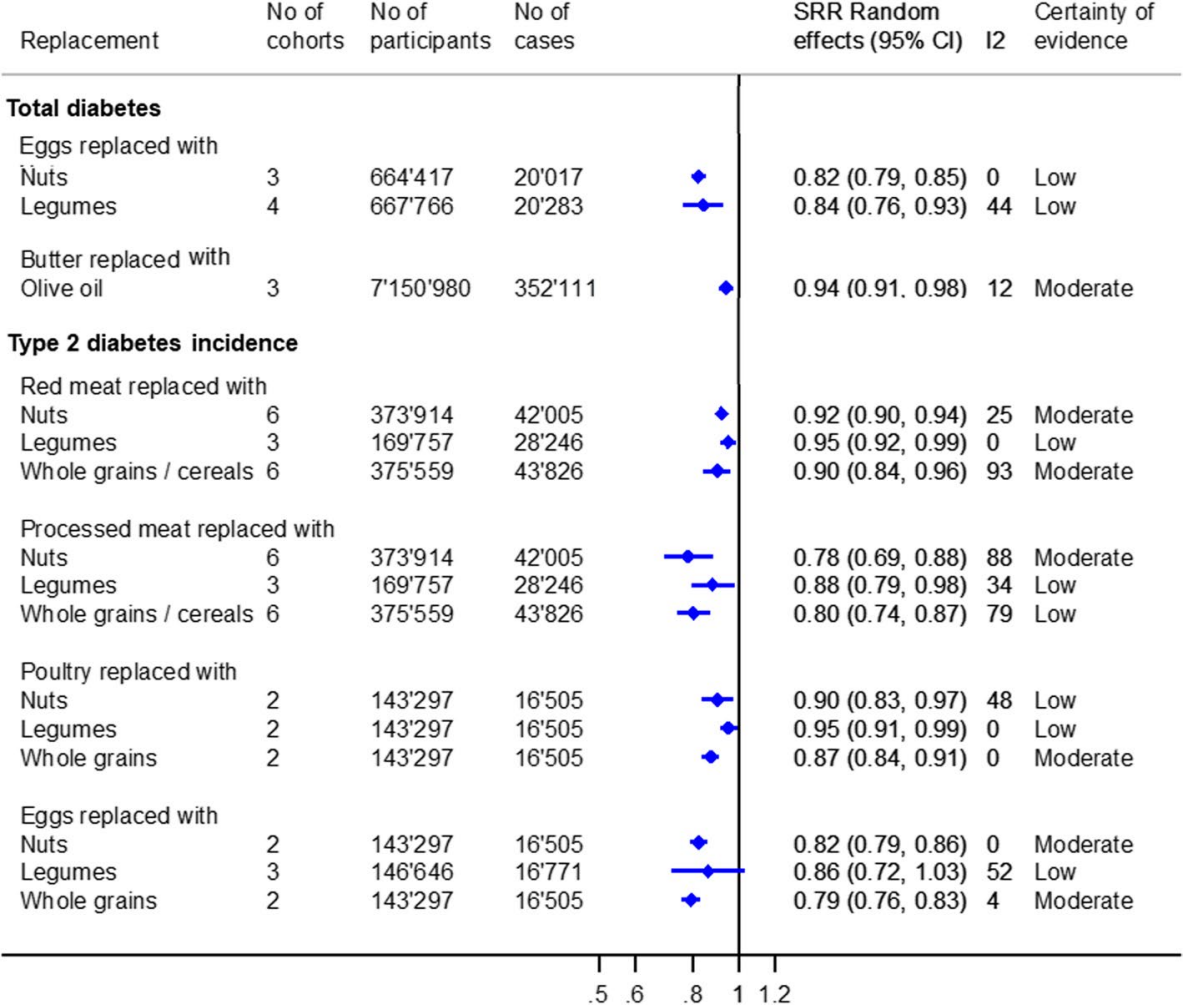
Substitution meta-analyses replacing animal-based foods with plant-based foods regarding type 2 diabetes incidence and total diabetes (type 2 diabetes incidence and diabetes mortality). Portion sizes: red and processed meat/red meat/processed meat: 50 g/day; nuts: 10–28 g/day; legumes: 18–50 g/day; whole grains/cereals: 11–30 g/day; butter: 5 g/day; and olive oil: 5 g/day

The meta-analyses regarding total T2D (incidence and mortality combined) and T2D incidence are shown in Fig. 4 and Additional file 3: Figs. S6 and S7.

There was moderate certainty of evidence for a lower risk of total T2D associated with replacing butter (5 g/day) with olive oil (5 g/day) [SHR (95% CI): 0.94 (0.91, 0.98), *I* ^2^ = 12%, *n*_cohorts_ = 3] (Additional file 2: Table S8). Moreover, we observed an association with a lower T2D incidence with moderate certainty of evidence when substituting red meat (50 g/day) with nuts (10 g/day) [SHR (95% CI): 0.92 (0.90, 0.94), *I* ^2^ = 25%, *n*_cohorts_ = 6] or whole grains/cereals (11–30 g/day) [SHR (95% CI): 0.90 (0.84, 0.96), *I* ^2^ = 93%, *n*_cohorts_ = 6], as well as processed meat (50 g/day) with nuts (10–28 g/day) [SHR (95% CI): 0.78 (0.69, 0.88), *I* ^2^ = 88%, *n*_cohorts_ = 6] and poultry (50 g/day) with whole grains (30 g/day) [SHR (95% CI): 0.87 (0.84, 0.91), *I* ^2^ = 0%, *n*_cohorts_ = 2]. Furthermore, replacing one egg/day with nuts (10 g/day) or whole grains (30 g/day) was also inversely associated with T2D incidence [SHR (95% CI): 0.82 (0.79, 0.86), *I* ^2^ = 0%, *n*_cohorts_ = 2, or 0.79 (0.76, 0.83), *I* ^2^ = 4%, *n*_cohorts_ = 2]. The remaining associations were rated as low or very low (Additional file 2: Table S9).

### All-cause mortality

**Fig. 5:**
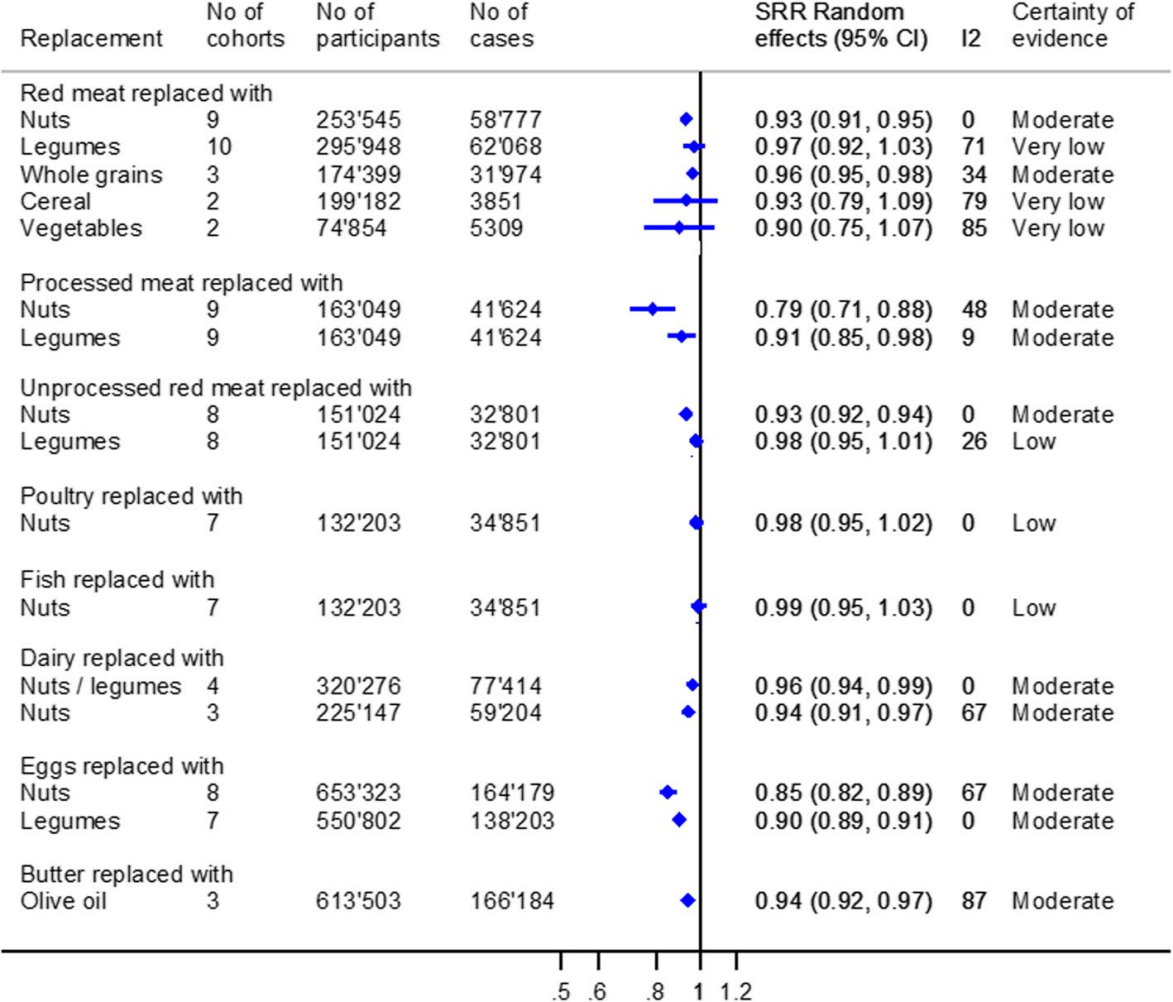
Substitution meta-analyses replacing animal-based foods with plant-based foods regarding all-cause mortality. Portion sizes: red meat/(un)processed meat/poultry/fish/dairy: 50 g/day; eggs: 1 egg/day; nuts: 10–50 g/day; legumes: 13–50 g/day; whole grains: 11–30 g/day; butter: 5 g/day; and olive oil: 5 g/day

The meta-analyses regarding all-cause mortality are shown in Fig. 5 and Additional file 3: Fig. S8.

We found moderate certainty of evidence for an association with a lower risk of all-cause mortality, when replacing red meat (50 g/day) with nuts (10–50 g/day) or whole grains (11–15 g/day) [SHR (95% CI): 0.93 (0.91, 0.95), *I* ^2^ = 0%, *n*_cohorts_ = 9, or 0.96 (0.95, 0.98), *I* ^2^ = 34%, *n*_cohorts_ = 3], processed meat (50 g/day) with nuts (28–50 g/day) or legumes (50 g/day) [SHR (95% CI): 0.79 (0.71, 0.88), *I* ^2^ = 48%, *n*_cohorts_ = 9 or 0.91 (0.85, 0.98), *I* ^2^ = 9%, *n*_cohorts_ = 9], and unprocessed red meat (50 g/day) with nuts (10–16 g/day) [SHR (95% CI): 0.93 (0.92, 0.94), *I* ^2^ = 0%, *n*_cohorts_ = 8]. Furthermore, replacing dairy (50 g/day) with nuts/legumes (7–50 g/day) or nuts only (7–50 g/day) [SHR (95% CI): 0.94 (0.92, 0.97), *I* ^2^ = 65%, *n*_cohorts_ = 6, or 0.94 (0.91, 0.97), *I* ^2^ = 67%, *n*_cohorts_ = 3], one egg/day with nuts (25–28 g/day) or legumes (25–50 g/day) [SHR (95% CI): 0.85 (0.82, 0.89), *I* ^2^ = 67%, *n*_cohorts_ = 8, or 0.90 (0.89, 0.91), *I* ^2^ = 0%, *n*_cohorts_ = 7], and butter (5 g/day) with olive oil (5 g/day) [SHR (95% CI): 0.94 (0.92, 0.97), *I* ^2^ = 87%, *n*_cohorts_ = 3] was also associated with a lower risk of all-cause mortality. The other associations were rated as low or very low certainty of evidence (Additional file 2: Table S10).

### Single study findings

A pooled analysis (based on two cohorts, only the pooled effect estimate was reported, and new meta-analysis was not possible) showed that replacing processed meat, yogurt, cheese, eggs, and butter with avocado was associated with a lower incidence of CVD (Additional file 3: Fig. S9) and CHD (Additional file 3: Fig. S10) [25]. Furthermore, the substitution of poultry, fish, and eggs with peanuts and peanut butter or with whole grain was associated with a lower T2D incidence (pooled analysis, based on three cohorts) [28]. The estimates on change over time were pooled from three cohorts, and the findings showed an inverse association with T2D when substituting red meat, as well as unprocessed and processed red meat with nuts and legumes (Additional file 3: Fig. S11) [11]. In addition, replacing processed meat, unprocessed red meat, dairy, and eggs with whole grains was associated with a lower risk of all-cause mortality (pooled analysis, based on six cohorts) [14, 24]. Furthermore, the estimates on change over time were pooled from two cohorts and showed that replacing red meat, processed meat, and unprocessed meat with nuts or whole grains was associated with a lower risk of all-cause mortality (Additional file 3: Fig. S12) [29].

Furthermore, several substitution analyses were only conducted in one single cohort. In these studies, there was an association with a lower risk of CVD mortality when substituting butter with canola oil (Additional file 3: Fig. S13) [167]. In addition, replacing butter with margarine was associated with an increased incidence of CVD (Additional file 3: Fig. S13) and CHD (Additional file 3: Fig. S14) [161]. Furthermore, there was an inverse association with T2D incidence for the substitution of milk with coffee or tea [172], as well as of sweetened milk-beverages with drinking water [173]. The study estimates based on change over time showed an inverse association with T2D for the replacement of red meat with whole grains or refined grains (Additional file 3: Fig. S15). Moreover, there were associations with a lower risk of all-cause mortality when substituting butter with margarine, corn oil, and canola oil [167], as well as animal cooking oil with plant oils other than peanut, soybean, canola, or salad oil (Additional file 3: Fig. S16) [177].

### Heterogeneity, subgroup, sensitivity and bias analysis

*I* ^2^ is reported in Figs. 2, 3, 4 and 5 and, together with *tau*^2^ and the 95% PIs, in the individual forest plots in Additional file 3: Figs. S3-S8. The only 95% PI that excluded the null value was for the association between the substitution of red meat with nuts on T2D incidence (95% PI: 0.85, 0.98), indicating that the true effects of future studies are expected to point in the same direction. All the other 95% PIs were wider and indicated that the true effect of future studies could be null or point in the opposite direction.

Since only few publications were available for the meta-analyses, which partly provided pooled risk estimates of multiple cohorts, but no single estimates [11, 14, 24, 26, 28, 29, 154, 159, 162, 163, 166], no subgroup analyses or assessment of publication bias was possible.

In sensitivity analyses leaving out one study at a time, the results remained mainly robust. The direction of the associations always remained the same; however, for several associations, the risk estimate changed in magnitude and precision. For the substitution of red and processed meat with nuts regarding CVD mortality, the SHR (95% CI) changed from 0.78 (0.53, 1.14) to 0.62 (0.44, 0.88) when excluding the study by Sun et al. [165] (WHI). Moreover, the association between the replacement of eggs with legumes regarding T2D incidence changed from an SHR (95% CI) of 0.86 (0.72, 1.03) to 0.63 (0.22, 1.84) and 0.60 (0.24, 1.55) when leaving out the study by Li et al. [179] (WHI) and Li et al. [179] (UK Biobank study (UKB)), respectively (data not shown).

## Discussion

This is the first systematic review and meta-analysis that summarized the associations between the substitution of animal-based with plant-based foods with a wide range of cardiometabolic outcomes, such as CVD mortality; incidence of CVD, CHD, and T2D; diabetes mortality, and all-cause mortality, and evaluated the risk of bias and certainty of evidence of the meta-findings. Our results indicate that replacing animal-based with plant-based foods is beneficially associated with cardiometabolic health. The substitution of red and processed meat with nuts, legumes, and whole grains reduced the risk of total CVD, CHD, and T2D and all-cause mortality with moderate certainty of evidence. We also observed moderate certainty of evidence for the association between the replacement of eggs with nuts and a lower incidence of total CVD and all-cause mortality. Additionally, replacing butter with olive oil lowered the incidence of total CVD, CVD mortality, total diabetes, and all-cause mortality with moderate certainty of evidence.

Furthermore, we found moderate certainty of evidence for an inverse association between the substitution of poultry with whole grains and T2D incidence and for the replacement of dairy products with nuts and legumes regarding all-cause mortality. For the other associations regarding the replacement of dairy products, poultry, or fish/seafood with plant-based foods, the certainty of evidence was low. However, our results indicated an inverse association for the substitution of poultry with nuts or legumes in relation to T2D incidence but no clear association for replacing dairy products, fish/seafood, or poultry with nuts or legumes regarding total CVD. In addition, no association was observed for the substitution of fish/seafood or poultry with nuts or legumes regarding all-cause mortality.

Our results are in agreement with a previous systematic review and meta-analysis investigating the replacement of red meat with other protein sources on CHD and all-cause mortality [15]. The authors observed a lower risk of CHD, when replacing red meat, processed red meat, and unprocessed red meat with nuts or legumes, and a lower risk of all-cause mortality when substituting red meat with nuts and processed red meat with nuts or legumes [15]. The certainty of evidence was assessed using NutriGrade [180] and was also rated as moderate for these associations [15]. However, they did not investigate further cardiometabolic endpoints, such as CVD mortality, CVD incidence, or T2D. Previous meta-analyses also support our findings that higher meat intake was associated with a risk of T2D [8], CHD, and stroke [7], as well as all-cause mortality [9]. This also applies to the associations between olive oil and CVD, T2D, and all-cause mortality [181, 182]. In contrast, a higher intake of eggs and butter was not associated with these outcomes in previous studies [7–9]. However, these studies included no substitution analyses. This suggests that not only the amount of these foods consumed is important but that it also plays a role what foods they are replaced with. Furthermore, our results indicate that the replacement of dairy products with nuts and legumes is associated with a lower risk of CVD or all-cause mortality. However, dairy includes a wide range of different products (e.g., milk, yogurt, cheese) with different associations with cardiometabolic outcomes. For example, contrary to our results, a higher intake of yogurt was inversely associated with risk for all-cause and CVD mortality [183]. Thus, associations for the replacement of different subtypes of dairy products with plant-based foods might differ, and more research is needed on this aspect. A network meta-analysis of randomized controlled trials comparing different food groups and their effect on intermediate diseases markers, such as blood lipids, blood pressure, and glycaemic control parameters, also found that plant-based foods, such as nuts, legumes, and whole grains, were most beneficial for all outcomes combined [184]. Given these results, stronger associations for the replacement of animal-based foods with legumes might have been expected. However, the network meta-analysis focussed on intermediate endpoints and rather reflects short-term effects due to the inclusion of RCTs with shorter follow-up times (mainly around 8–12 weeks). Therefore, long-term effects on hard endpoints might differ. However, the certainty of evidence for the majority of the findings on legumes was low or very low, limiting our confidence in the results. Thus, future studies might provide more insight into these associations. Moreover, in line with our results, a network meta-analysis comparing different types of oils and solid fats on blood lipids confirmed a more beneficial effect of olive oil compared to butter [185].

There are different mechanisms that might explain the observed associations. First, it is likely that persons favoring plant-based foods follow a more health-conscious lifestyle in general. However, the included studies all adjusted for important lifestyle confounders such as total energy intake, physical activity, alcohol intake, and smoking, and the associations persisted. Nevertheless, residual confounding cannot be ruled out.

Furthermore, processed meat contains saturated fatty acids, such as stearic and palmitic acid, which potentially increase the risk of CVD and T2D [108, 116]. Moreover, red and processed meats contain compounds promoting oxidative stress and chronic low-grade inflammation, such as heme iron, sodium, nitrates, and nitrites that might be associated with increased risk of CVD, T2D, and mortality [54, 186]. In contrast, plant-based foods such as nuts, legumes, and whole grains, as well as olive oil, contain high amounts of anti-oxidant and anti-inflammatory compounds, including fiber, phytochemicals, vitamins and minerals, and polyphenols that show beneficial associations with cardiovascular health and, in relation to cardiovascular health, obesity [187, 188]. Thus, both the reduction of animal-based foods, especially meat, and the increase of plant-based foods simultaneously contribute to the observed beneficial associations regarding cardiometabolic health.

Our findings also highlight the need for future research. First, some of the results still show some inconsistencies. For example, both the substitution of processed and unprocessed red meat with legumes were associated with a lower risk of total CVD, while the association for the replacement of red meat with legumes was less clear. This was mainly due to an imprecise estimate of the latter association also resulting in a very low level of the certainty of evidence. Therefore, more studies on this association are needed in order to provide further insights into the consistency of these associations and to improve our confidence in the findings. In general, to increase the certainty of evidence, well-conducted prospective observational studies on the associations with low and very low certainty of evidence and on the associations only investigated in single studies are needed. This includes for example the substitution of dairy products, especially subtypes of dairy products, poultry, or fish/seafood with plant-based products, or the replacement of animal-based foods with further plant-based foods, such as fruit and vegetables. These studies should minimize the risk of bias, e.g., by using models with a sufficient level of adjustment, and use standardized portion sizes (e.g., 50 g for meat/poultry/fish/seafood, 30 g for whole grains/legumes, 5 g for butter/olive oil) for both the substituted food and the replacement in order to improve the comparability between the results and to decrease heterogeneity between the studies. Furthermore, the majority of the studies investigated a theoretical substitution. Future studies should focus more on an observed change in food intake, as was done in three recent studies [11, 29, 30]. Moreover, more research is needed on the substitution of animal-based products with meat and dairy replacement foods, which strongly gained popularity in recent years.

### Strengths and limitations

This is the first systematic review and meta-analysis focussing on the association between the substitution of animal-based with plant-based foods on multiple cardiovascular outcomes, including 37 studies on CVD, CHD, T2D, related mortality, and all-cause mortality. We followed the recommended procedures (pre-registered protocol and reporting guidelines) and applied validated tools for the risk of bias and certainty of evidence assessments. Moreover, we used standardized portions for the substituted foods, which increased the comparability of the results.

However, our work also has several limitations. First, only few studies were available for some of the meta-analyses, and these findings should be interpreted with caution and more studies on this topic are needed. While some studies provided pooled risk estimates of multiple cohorts, no separate estimates for these single cohorts were available, and thus, subgroup and sensitivity analyses excluding studies with a high risk of bias or assessment of publication bias could not be conducted. Second, several substitution analyses were only investigated in one study. Therefore, no meta-analyses were possible. More prospective studies with substitution analyses for these associations are needed in order to further investigate this evidence. Third, while we increased the comparability of the results, using standardized portions for the substituted foods, this was not possible for the substitute simultaneously. We adapted the portion sizes of the replacement according to the conversion of the substituted foods. However, given the varying portion sizes between studies, the portions of the replacement still varied, leading to the comparison of different amounts of the replacement. This was considered in the indirectness domain in GRADE, contributing to the downgrading of the certainty of evidence for the respective associations. Fourth, due to the observational design of the included studies, unknown confounding cannot be ruled out. Therefore, no study could be judged as low risk of bias in ROBINS-I, leading to a downgrade in the GRADE risk of bias domain. Furthermore, given that the substitution models adjusted for total energy intake while controlling for different foods in grams per day, a residual energy substitution was introduced [10]. However, we only included prospective observational studies thus minimizing the risk of selection or recall bias. Fifth, to date, no study is available regarding meat and dairy replacement products, which have rapidly increased in popularity in recent years. Sixth, dairy products were mostly treated as one group, and more research is needed on the substitution of subtypes of dairy products with plant-based foods.

## Conclusions

Our findings suggest that a shift in diet from a high consumption of animal-based foods, especially red and processed meat, to plant-based foods (e.g., nuts, legumes, and whole grains) is associated with a lower risk of all-cause mortality, CVD, and T2D. Thus, a change in dietary habits towards an increment of plant-based products appears to be important for cardiometabolic health. However, more research is needed in order to strengthen the existing evidence and to investigate new associations, especially with a focus on meat and dairy replacement products.

### Supplementary Information

**Additional file 1. **Search terms.

**Additional file 2: Table S1.** Eligibility criteria by the PICOS statement. **Table S2.** Study characteristics. **Table S3.** Description and decision criteria for each domain in ROBINS-I.** Table S4.** List of excluded studies. **Table S5.** GRADE assessment for the substitution analyses regarding total CVD. **Table S6.** GRADE assessment for the substitution analyses regarding CVD mortality. **Table S7.** GRADE assessment for the substitution analyses regarding CHD incidence. **Table S8.** GRADE assessment for the substitution analyses regarding total diabetes. **Table S9.** GRADE assessment for the substitution analyses regarding type 2 diabetes incidence. **Table S10.** GRADE assessment for the substitution analyses regarding all-cause mortality.

**Additional file 3: Fig. S1.** Risk of bias of each study for each domain and overall. **Fig. S2.** Risk of bias of judgements within each bias domain. **Fig. S3.** Forest plots for the substitution analyses regarding total CVD. **Fig. S4.** Forest plots for the substitution analyses regarding CVD mortality. **Fig. S5.** Forest plots for the substitution analyses regarding CHD incidence. **Fig. S6.** Forest plot for the substitution analyses regarding total diabetes. **Fig. S7.** Forest plots for the substitution analyses regarding incidence of type 2 diabetes. **Fig. S8.** Forest plots for the substitution analyses regarding all-cause mortality. **Fig. S9.** Forest plot showing the results from extracted pooled analyses regarding CVD mortality and CVD incidence. **Fig. S10.** Forest plot showing the results from extracted pooled analyses regarding CHD and stroke incidence. **Fig. S11.** Forest plot showing the results from extracted pooled analyses regarding diabetes. **Fig. S12.** Forest plot showing the results from extracted pooled analyses regarding all-cause mortality. **Fig. S13.** Forest plot showing the results from single cohorts regarding CVD mortality and CVD incidence. **Fig. S14.** Forest plot showing the results from single cohorts regarding CHD, MI and stroke incidence. **Fig. S15.** Forest plot showing the results from single cohorts regarding diabetes. **Fig. S16.** Forest plot showing the results from single cohorts regarding all-cause mortality.

## Data Availability

This manuscript makes use of publicly available data from published studies; therefore, no original data are available for sharing.

## References

[1] Hemler EC, Hu FB (2019). Plant-based diets for personal, population, and planetary health. Adv Nutr.

[2] Fresan U, Sabate J (2019). Vegetarian diets: planetary health and its alignment with human health. Adv Nutr.

[3] GBD Diseases Injuries Collaborators (2020). Global burden of 369 diseases and injuries in 204 countries and territories, 1990–2019: a systematic analysis for the Global Burden of Disease Study 2019. Lancet.

[4] Moreno LA, Meyer R, Donovan SM, Goulet O, Haines J, Kok FJ (2021). Perspective: striking a balance between planetary and human health: is there a path forward?. Adv Nutr.

[5] Oussalah A, Levy J, Berthezene C, Alpers DH, Gueant JL (2020). Health outcomes associated with vegetarian diets: an umbrella review of systematic reviews and meta-analyses. Clin Nutr.

[6] GBD Diet Collaborators (2019). Health effects of dietary risks in 195 countries, 1990–2017: a systematic analysis for the Global Burden of Disease Study 2017. Lancet.

[7] Bechthold A, Boeing H, Schwedhelm C, Hoffmann G, Knuppel S, Iqbal K, De Henauw S, Michels N, Devleesschauwer B, Schlesinger S (2019). Food groups and risk of coronary heart disease, stroke and heart failure: a systematic review and dose-response meta-analysis of prospective studies. Crit Rev Food Sci Nutr.

[8] Schwingshackl L, Hoffmann G, Lampousi AM, Knuppel S, Iqbal K, Schwedhelm C, Bechthold A, Schlesinger S, Boeing H (2017). Food groups and risk of type 2 diabetes mellitus: a systematic review and meta-analysis of prospective studies. Eur J Epidemiol.

[9] Schwingshackl L, Schwedhelm C, Hoffmann G, Lampousi AM, Knuppel S, Iqbal K, Bechthold A, Schlesinger S, Boeing H (2017). Food groups and risk of all-cause mortality: a systematic review and meta-analysis of prospective studies. Am J Clin Nutr.

[10] Ibsen DB, Laursen ASD, Wurtz AML, Dahm CC, Rimm EB, Parner ET, Overvad K, Jakobsen MU (2021). Food substitution models for nutritional epidemiology. Am J Clin Nutr.

[11] Wurtz AML, Jakobsen MU, Bertoia ML, Hou T, Schmidt EB, Willett WC, Overvad K, Sun Q, Manson JE, Hu FB (2021). Replacing the consumption of red meat with other major dietary protein sources and risk of type 2 diabetes mellitus: a prospective cohort study. Am J Clin Nutr.

[12] Nielsen TB, Wurtz AML, Tjonneland A, Overvad K, Dahm CC (2022). Substitution of unprocessed and processed red meat with poultry or fish and total and cause-specific mortality. Br J Nutr.

[13] Al-Shaar L, Satija A, Wang DD, Rimm EB, Smith-Warner SA, Stampfer MJ, et al. Red meat intake and risk of coronary heart disease among US men: prospective cohort study. BMJ. 2020;371:m4141. 10.1136/bmj.m4141 PMC8030119 33268459

[14] Zhong VW, Allen NB, Greenland P, Carnethon MR, Ning H, Wilkins JT, Lloyd-Jones DM, Van Horn L (2021). Protein foods from animal sources, incident cardiovascular disease and all-cause mortality: a substitution analysis. Int J Epidemiol.

[15] Hidayat K, Chen JS, Wang HP, Wang TC, Liu YJ, Zhang XY, Rao CP, Zhang JW, Qin LQ (2022). Is replacing red meat with other protein sources associated with lower risks of coronary heart disease and all-cause mortality? A meta-analysis of prospective studies. Nutr Rev.

[16] Page MJ, McKenzie JE, Bossuyt PM, Boutron I, Hoffmann TC, Mulrow CD, Shamseer L, Tetzlaff JM, Akl EA, Brennan SE (2021). The PRISMA 2020 statement: an updated guideline for reporting systematic reviews. BMJ.

[17] Sterne JA, Hernan MA, Reeves BC, Savovic J, Berkman ND, Viswanathan M, Henry D, Altman DG, Ansari MT, Boutron I (2016). ROBINS-I: a tool for assessing risk of bias in non-randomised studies of interventions. BMJ.

[18] Schunemann HJ, Cuello C, Akl EA, Mustafa RA, Meerpohl JJ, Thayer K, Morgan RL, Gartlehner G, Kunz R, Katikireddi SV (2019). GRADE guidelines: 18. How ROBINS-I and other tools to assess risk of bias in nonrandomized studies should be used to rate the certainty of a body of evidence. J Clin Epidemiol.

[19] Balshem H, Helfand M, Schunemann HJ, Oxman AD, Kunz R, Brozek J, Vist GE, Falck-Ytter Y, Meerpohl J, Norris S (2011). GRADE guidelines: 3. Rating the quality of evidence. J Clin Epidemiol.

[20] Borenstein M, Hedges LV, Higgins JP, Rothstein HR (2010). A basic introduction to fixed-effect and random-effects models for meta-analysis. Res Synth Methods.

[21] DerSimonian R, Laird N (2015). Meta-analysis in clinical trials revisited. Contemp Clin Trials.

[22] Borenstein M, Higgins JP, Hedges LV, Rothstein HR (2017). Basics of meta-analysis: I(2) is not an absolute measure of heterogeneity. Res Synth Methods.

[23] Riley RD, Higgins JP, Deeks JJ (2011). Interpretation of random effects meta-analyses. BMJ.

[24] Ding M, Li J, Qi L, Ellervik C, Zhang X, Manson JE, et al. Associations of dairy intake with risk of mortality in women and men: three prospective cohort studies. BMJ. 2019;367:l6204. 10.1136/bmj.l6204 PMC6880246 31776125

[25] Pacheco LS, Li Y, Rimm EB, Manson JE, Sun Q, Rexrode K, Hu FB, Guasch-Ferre M (2022). Avocado consumption and risk of cardiovascular disease in US adults. J Am Heart Assoc.

[26] Guasch-Ferré M, Liu G, Li Y, Sampson L, Manson JE, Salas-Salvadó J (2020). Olive oil consumption and cardiovascular risk in U.S. adults. J Am Coll Cardiol.

[27] Bernstein AM, Pan A, Rexrode KM, Stampfer M, Hu FB, Mozaffarian D (2012). Dietary protein sources and the risk of stroke in men and women. Stroke.

[28] Malik VS, Li Y, Tobias DK, Pan A, Hu FB (2016). Dietary protein intake and risk of type 2 diabetes in US men and women. Am J Epidemiol.

[29] Zheng Y, Li Y, Satija A, Pan A, Sotos-Prieto M, Rimm E, Willett WC, Hu FB (2019). Association of changes in red meat consumption with total and cause specific mortality among US women and men: two prospective cohort studies. BMJ.

[30] Ibsen DB, Jakobsen MU, Halkjaer J, Tjonneland A, Kilpelainen TO, Parner ET, Overvad K (2021). Replacing red meat with other nonmeat food sources of protein is associated with a reduced risk of type 2 diabetes in a Danish cohort of middle-aged adults. J Nutr.

[31] Sterne JA, Sutton AJ, Ioannidis JP, Terrin N, Jones DR, Lau J, Carpenter J, Rucker G, Harbord RM, Schmid CH (2011). Recommendations for examining and interpreting funnel plot asymmetry in meta-analyses of randomised controlled trials. BMJ.

[32] Egger M, Davey Smith G, Schneider M, Minder C (1997). Bias in meta-analysis detected by a simple, graphical test. BMJ.

[33] Higgins JPT GSe. Cochrane Handbook for Systematic Reviews of Interventions Version 5.1.0. In: The Cochrane Collaboration; 2011. Updated March 2011. Chapter 10.4.

[34] Ardisson Korat AV, Li Y, Sacks F, Rosner B, Willett WC, Hu FB, Sun Q (2019). Dairy fat intake and risk of type 2 diabetes in 3 cohorts of US men and women. Am J Clin Nutr.

[35] Bazzano LA, He J, Ogden LG, Loria C, Vupputuri S, Myers L, Whelton PK (2001). Legume consumption and risk of coronary heart disease in US men and women: NHANES I Epidemiologic Follow-up Study. Arch Intern Med.

[36] InterAct C, Bendinelli B, Palli D, Masala G, Sharp SJ, Schulze MB, Guevara M, van der AD, Sera F, Amiano P (2013). Association between dietary meat consumption and incident type 2 diabetes: the EPIC-InterAct study. Diabetologia.

[37] Budhathoki S, Sawada N, Iwasaki M, Yamaji T, Goto A, Kotemori A, Ishihara J, Takachi R, Charvat H, Mizoue T (2019). Association of animal and plant protein intake with all-cause and cause-specific mortality in a Japanese cohort. JAMA Intern Med.

[38] Buil-Cosiales P, Zazpe I, Toledo E, Corella D, Salas-Salvado J, Diez-Espino J, Ros E, Fernandez-Creuet Navajas J, Santos-Lozano JM, Aros F (2014). Fiber intake and all-cause mortality in the Prevencion con Dieta Mediterranea (PREDIMED) study. Am J Clin Nutr.

[39] Chan R, Leung J, Woo J (2019). High protein intake is associated with lower risk of all-cause mortality in community-dwelling Chinese older men and women. J Nutr Health Aging.

[40] Chen GC, Chen LH, Mossavar-Rahmani Y, Kamensky V, Shadyab AH, Haring B, Wild RA, Silver B, Kuller LH, Sun Y (2021). Dietary cholesterol and egg intake in relation to incident cardiovascular disease and all-cause and cause-specific mortality in postmenopausal women. Am J Clin Nutr.

[41] Chen M, Li Y, Sun Q, Pan A, Manson JE, Rexrode KM, Willett WC, Rimm EB, Hu FB (2016). Dairy fat and risk of cardiovascular disease in 3 cohorts of US adults. Am J Clin Nutr.

[42] Chen M, Sun Q, Giovannucci E, Mozaffarian D, Manson JE, Willett WC, Hu FB (2014). Dairy consumption and risk of type 2 diabetes: 3 cohorts of US adults and an updated meta-analysis. BMC Med.

[43] Chen Z, Glisic M, Song M, Aliahmad HA, Zhang X, Moumdjian AC, Gonzalez-Jaramillo V, van der Schaft N, Bramer WM, Ikram MA (2020). Dietary protein intake and all-cause and cause-specific mortality: results from the Rotterdam Study and a meta-analysis of prospective cohort studies. Eur J Epidemiol.

[44] de Goede J, Geleijnse JM, Boer JM, Kromhout D, Verschuren WM (2010). Marine (n-3) fatty acids, fish consumption, and the 10-year risk of fatal and nonfatal coronary heart disease in a large population of Dutch adults with low fish intake. J Nutr.

[45] de Oliveira Otto MC, Mozaffarian D, Kromhout D, Bertoni AG, Sibley CT, Jacobs DR, Nettleton JA (2012). Dietary intake of saturated fat by food source and incident cardiovascular disease: the Multi-Ethnic Study of Atherosclerosis. Am J Clin Nutr.

[46] Dehghan M, Mente A, Rangarajan S, Mohan V, Lear S, Swaminathan S, Wielgosz A, Seron P, Avezum A, Lopez-Jaramillo P (2020). Association of egg intake with blood lipids, cardiovascular disease, and mortality in 177,000 people in 50 countries. Am J Clin Nutr.

[47] Dehghan M, Mente A, Rangarajan S, Sheridan P, Mohan V, Iqbal R, Gupta R, Lear S, Wentzel-Viljoen E, Avezum A (2018). Association of dairy intake with cardiovascular disease and mortality in 21 countries from five continents (PURE): a prospective cohort study. Lancet.

[48] Dehghan M, Mente A, Zhang X, Swaminathan S, Li W, Mohan V, Iqbal R, Kumar R, Wentzel-Viljoen E, Rosengren A (2017). Associations of fats and carbohydrate intake with cardiovascular disease and mortality in 18 countries from five continents (PURE): a prospective cohort study. Lancet.

[49] Diaz-Lopez A, Bullo M, Martinez-Gonzalez MA, Corella D, Estruch R, Fito M, Gomez-Gracia E, Fiol M, Garcia de la Corte  FJ, Ros E (2016). Dairy product consumption and risk of type 2 diabetes in an elderly Spanish Mediterranean population at high cardiovascular risk. Eur J Nutr.

[50] Diez-Espino J, Basterra-Gortari FJ, Salas-Salvado J, Buil-Cosiales P, Corella D, Schroder H, Estruch R, Ros E, Gomez-Gracia E, Aros F (2017). Egg consumption and cardiovascular disease according to diabetic status: the PREDIMED study. Clin Nutr.

[51] Djousse L, Gaziano JM (2008). Egg consumption in relation to cardiovascular disease and mortality: the Physicians’ Health Study. Am J Clin Nutr.

[52] Djousse L, Zhou G, McClelland RL, Ma N, Zhou X, Kabagambe EK, Talegawkar SA, Judd SE, Biggs ML, Fitzpatrick AL (2021). Egg consumption, overall diet quality, and risk of type 2 diabetes and coronary heart disease: a pooling project of US prospective cohorts. Clin Nutr.

[53] Du H, Guo Y, Bennett DA, Bragg F, Bian Z, Chadni M, Yu C, Chen Y, Tan Y, Millwood IY (2020). Red meat, poultry and fish consumption and risk of diabetes: a 9 year prospective cohort study of the China Kadoorie Biobank. Diabetologia.

[54] Etemadi A, Sinha R, Ward MH, Graubard BI, Inoue-Choi M, Dawsey SM, Abnet CC (2017). Mortality from different causes associated with meat, heme iron, nitrates, and nitrites in the NIH-AARP Diet and Health Study: population based cohort study. BMJ.

[55] Farvid MS, Malekshah AF, Pourshams A, Poustchi H, Sepanlou SG, Sharafkhah M, Khoshnia M, Farvid M, Abnet CC, Kamangar F (2017). Dairy food intake and all-cause, cardiovascular disease, and cancer mortality: the Golestan Cohort Study. Am J Epidemiol.

[56] Fung TT, Schulze M, Manson JE, Willett WC, Hu FB (2004). Dietary patterns, meat intake, and the risk of type 2 diabetes in women. Arch Intern Med.

[57] Fung TT, van Dam RM, Hankinson SE, Stampfer M, Willett WC, Hu FB (2010). Low-carbohydrate diets and all-cause and cause-specific mortality: two cohort studies. Ann Intern Med.

[58] Gammelmark A, Nielsen MS, Bork CS, Lundbye-Christensen S, Tjonneland A, Overvad K, Schmidt EB (2016). Association of fish consumption and dietary intake of marine n-3 PUFA with myocardial infarction in a prospective Danish cohort study. Br J Nutr.

[59] Guasch-Ferre M, Babio N, Martinez-Gonzalez MA, Corella D, Ros E, Martin-Pelaez S, Estruch R, Aros F, Gomez-Gracia E, Fiol M (2015). Dietary fat intake and risk of cardiovascular disease and all-cause mortality in a population at high risk of cardiovascular disease. Am J Clin Nutr.

[60] Guasch-Ferre M, Zong G, Willett WC, Zock PL, Wanders AJ, Hu FB, Sun Q (2019). Associations of monounsaturated fatty acids from plant and animal sources with total and cause-specific mortality in two US prospective cohort studies. Circ Res.

[61] Hansen MD, Wurtz AML, Hansen CP, Tjonneland A, Rimm EB, Johnsen SP, Schmidt EB, Overvad K, Jakobsen MU (2021). Substitutions between potatoes and other vegetables and risk of ischemic stroke. Eur J Nutr.

[62] Hu FB, Bronner L, Willett WC, Stampfer MJ, Rexrode KM, Albert CM, Hunter D, Manson JE (2002). Fish and omega-3 fatty acid intake and risk of coronary heart disease in women. JAMA.

[63] Hu FB, Stampfer MJ, Manson JE, Ascherio A, Colditz GA, Speizer FE, Hennekens CH, Willett WC (1999). Dietary saturated fats and their food sources in relation to the risk of coronary heart disease in women. Am J Clin Nutr.

[64] Hu FB, Stampfer MJ, Manson JE, Rimm E, Colditz GA, Speizer FE, Hennekens CH, Willett WC (1999). Dietary protein and risk of ischemic heart disease in women. Am J Clin Nutr.

[65] Hu Y, Ding M, Sampson L, Willett WC, Manson JE, Wang M, Rosner B, Hu FB, Sun Q (2020). Intake of whole grain foods and risk of type 2 diabetes: results from three prospective cohort studies. BMJ.

[66] Huang J, Liao LM, Weinstein SJ, Sinha R, Graubard BI, Albanes D (2020). Association between plant and animal protein intake and overall and cause-specific mortality. JAMA Intern Med.

[67] Ibsen DB, Overvad K, Laursen ASD, Halkjaer J, Tjonneland A, Kilpelainen TO, Parner ET, Jakobsen MU (2021). Changes in intake of dairy product subgroups and risk of type 2 diabetes: modelling specified food substitutions in the Danish Diet, Cancer and Health cohort. Eur J Nutr.

[68] Ibsen DB, Warberg CK, Wurtz AML, Overvad K, Dahm CC (2019). Substitution of red meat with poultry or fish and risk of type 2 diabetes: a Danish cohort study. Eur J Nutr.

[69] Iqbal R, Dehghan M, Mente A, Rangarajan S, Wielgosz A, Avezum A, Seron P, AlHabib KF, Lopez-Jaramillo P, Swaminathan S (2021). Associations of unprocessed and processed meat intake with mortality and cardiovascular disease in 21 countries [Prospective Urban Rural Epidemiology (PURE) Study]: a prospective cohort study. Am J Clin Nutr.

[70] Jakobsen MU, Dethlefsen C, Joensen AM, Stegger J, Tjonneland A, Schmidt EB, Overvad K (2010). Intake of carbohydrates compared with intake of saturated fatty acids and risk of myocardial infarction: importance of the glycemic index. Am J Clin Nutr.

[71] Jang J, Shin MJ, Kim OY, Park K (2018). Longitudinal association between egg consumption and the risk of cardiovascular disease: interaction with type 2 diabetes mellitus. Nutr Diabetes.

[72] Jiang R, Manson JE, Stampfer MJ, Liu S, Willett WC, Hu FB (2002). Nut and peanut butter consumption and risk of type 2 diabetes in women. JAMA.

[73] Joshipura KJ, Hung HC, Li TY, Hu FB, Rimm EB, Stampfer MJ, Colditz G, Willett WC (2009). Intakes of fruits, vegetables and carbohydrate and the risk of CVD. Public Health Nutr.

[74] Kelemen LE, Kushi LH, Jacobs DR, Cerhan JR (2005). Associations of dietary protein with disease and mortality in a prospective study of postmenopausal women. Am J Epidemiol.

[75] Key TJ, Appleby PN, Bradbury KE, Sweeting M, Wood A, Johansson I, Kuhn T, Steur M, Weiderpass E, Wennberg M (2019). Consumption of meat, fish, dairy products, and eggs and risk of ischemic heart disease. Circulation.

[76] Kuhn T, Teucher B, Kaaks R, Boeing H, Weikert C, Buijsse B (2013). Fish consumption and the risk of myocardial infarction and stroke in the German arm of the European Prospective Investigation into Cancer and Nutrition (EPIC-Germany). Br J Nutr.

[77] Kvist K, Laursen ASD, Overvad K, Jakobsen MU (2020). Substitution of milk with whole-fat yogurt products or cheese is associated with a lower risk of myocardial infarction: the Danish Diet. Cancer and Health cohort J Nutr.

[78] Kyro C, Tjonneland A, Overvad K, Olsen A, Landberg R (2018). Higher whole-grain intake is associated with lower risk of type 2 diabetes among middle-aged men and women: the Danish Diet, Cancer, and Health Cohort. J Nutr.

[79] Lajous M, Tondeur L, Fagherazzi G, de Lauzon-Guillain B, Boutron-Ruaualt MC, Clavel-Chapelon F (2012). Processed and unprocessed red meat consumption and incident type 2 diabetes among French women. Diabetes Care.

[80] Larsson SC, Virtamo J, Wolk A (2011). Red meat consumption and risk of stroke in Swedish women. Stroke.

[81] Lasota AN, Gronholdt MM, Bork CS, Lundbye-Christensen S, Schmidt EB, Overvad K (2019). Substitution of poultry and red meat with fish and the risk of peripheral arterial disease: a Danish cohort study. Eur J Nutr.

[82] Lee JE, McLerran DF, Rolland B, Chen Y, Grant EJ, Vedanthan R, Inoue M, Tsugane S, Gao YT, Tsuji I (2013). Meat intake and cause-specific mortality: a pooled analysis of Asian prospective cohort studies. Am J Clin Nutr.

[83] Li Y, Hruby A, Bernstein AM, Ley SH, Wang DD, Chiuve SE, Sampson L, Rexrode KM, Rimm EB, Willett WC (2015). Saturated fats compared with unsaturated fats and sources of carbohydrates in relation to risk of coronary heart disease: a prospective cohort study. J Am Coll Cardiol.

[84] Liu S, Manson JE, Lee IM, Cole SR, Hennekens CH, Willett WC, Buring JE (2000). Fruit and vegetable intake and risk of cardiovascular disease: the Women’s Health Study. Am J Clin Nutr.

[85] Liu S, van der Schouw YT, Soedamah-Muthu SS, Spijkerman AMW, Sluijs I (2019). Intake of dietary saturated fatty acids and risk of type 2 diabetes in the European Prospective Investigation into Cancer and Nutrition-Netherlands cohort: associations by types, sources of fatty acids and substitution by macronutrients. Eur J Nutr.

[86] Mao L, Zhang Y, Wang W, Zhuang P, Wu F, Jiao J (2020). Plant-sourced and animal-sourced monounsaturated fatty acid intakes in relation to mortality: a prospective nationwide cohort study. Eur J Nutr.

[87] Miller V, Mente A, Dehghan M, Rangarajan S, Zhang X, Swaminathan S, Dagenais G, Gupta R, Mohan V, Lear S (2017). Fruit, vegetable, and legume intake, and cardiovascular disease and deaths in 18 countries (PURE): a prospective cohort study. Lancet.

[88] O’Connor LM, Lentjes MA, Luben RN, Khaw KT, Wareham NJ, Forouhi NG (2014). Dietary dairy product intake and incident type 2 diabetes: a prospective study using dietary data from a 7-day food diary. Diabetologia.

[89] Ozawa M, Yoshida D, Hata J, Ohara T, Mukai N, Shibata M, Uchida K, Nagata M, Kitazono T, Kiyohara Y (2017). Dietary protein intake and stroke risk in a general Japanese population: the Hisayama Study. Stroke.

[90] Pan A, Sun Q, Bernstein AM, Manson JE, Willett WC, Hu FB (2013). Changes in red meat consumption and subsequent risk of type 2 diabetes mellitus: three cohorts of US men and women. JAMA Intern Med.

[91] Pan XF, Yang JJ, Lipworth LP, Shu XO, Cai H, Steinwandel MD (2021). Cholesterol and egg intakes with cardiometabolic and all-cause mortality among Chinese and low-income Black and White Americans. Nutrients.

[92] Papier K, Fensom GK, Knuppel A, Appleby PN, Tong TYN, Schmidt JA, Travis RC, Key TJ, Perez-Cornago A (2021). Meat consumption and risk of 25 common conditions: outcome-wide analyses in 475,000 men and women in the UK Biobank study. BMC Med.

[93] Park K, Son J, Jang J, Kang R, Chung HK, Lee KW (2017). Unprocessed meat consumption and incident cardiovascular diseases in Korean adults: the Korean Genome and Epidemiology Study (KoGES). Nutrients.

[94] Patel PS, Forouhi NG, Kuijsten A, Schulze MB, van Woudenbergh GJ, Ardanaz E, Amiano P, Arriola L, Balkau B, Barricarte A (2012). The prospective association between total and type of fish intake and type 2 diabetes in 8 European countries: EPIC-InterAct Study. Am J Clin Nutr.

[95] Patterson E, Larsson SC, Wolk A, Akesson A (2013). Association between dairy food consumption and risk of myocardial infarction in women differs by type of dairy food. J Nutr.

[96] Petermann-Rocha F, Parra-Soto S, Gray S, Anderson J, Welsh P, Gill J, Sattar N, Ho FK, Celis-Morales C, Pell JP (2021). Vegetarians, fish, poultry, and meat-eaters: who has higher risk of cardiovascular disease incidence and mortality? A prospective study from UK Biobank. Eur Heart J.

[97] Praagman J, de Jonge EA, Kiefte-de Jong JC, Beulens JW, Sluijs I, Schoufour JD, Hofman A, van der Schouw YT, Franco OH (2016). Dietary saturated fatty acids and coronary heart disease risk in a Dutch middle-aged and elderly population. Arterioscler Thromb Vasc Biol.

[98] Preis SR, Stampfer MJ, Spiegelman D, Willett WC, Rimm EB (2010). Lack of association between dietary protein intake and risk of stroke among middle-aged men. Am J Clin Nutr.

[99] Rebello SA, Koh H, Chen C, Naidoo N, Odegaard AO, Koh WP, Butler LM, Yuan JM, van Dam RM (2014). Amount, type, and sources of carbohydrates in relation to ischemic heart disease mortality in a Chinese population: a prospective cohort study. Am J Clin Nutr.

[100] Rohrmann S, Overvad K, Bueno-de-Mesquita HB, Jakobsen MU, Egeberg R, Tjonneland A, Nailler L, Boutron-Ruault MC, Clavel-Chapelon F, Krogh V (2013). Meat consumption and mortality–results from the European Prospective Investigation into Cancer and Nutrition. BMC Med.

[101] Ruggiero E, Di Castelnuovo A, Costanzo S, Persichillo M, De Curtis A, Cerletti C, Donati MB, de Gaetano G, Iacoviello L, Bonaccio M (2021). Egg consumption and risk of all-cause and cause-specific mortality in an Italian adult population. Eur J Nutr.

[102] Seidelmann SB, Claggett B, Cheng S, Henglin M, Shah A, Steffen LM, Folsom AR, Rimm EB, Willett WC, Solomon SD (2018). Dietary carbohydrate intake and mortality: a prospective cohort study and meta-analysis. Lancet Public Health.

[103] Sinha R, Cross AJ, Graubard BI, Leitzmann MF, Schatzkin A (2009). Meat intake and mortality: a prospective study of over half a million people. Arch Intern Med.

[104] Sluijs I, Forouhi NG, Beulens JW, van der Schouw YT, Agnoli C, Arriola L, Balkau B, Barricarte A, Boeing H, Bueno-de-Mesquita HB (2012). The amount and type of dairy product intake and incident type 2 diabetes: results from the EPIC-InterAct Study. Am J Clin Nutr.

[105] Sluik D, Brouwer-Brolsma EM, Berendsen AAM, Mikkila V, Poppitt SD, Silvestre MP, Tremblay A, Perusse L, Bouchard C, Raben A (2019). Protein intake and the incidence of pre-diabetes and diabetes in 4 population-based studies: the PREVIEW project. Am J Clin Nutr.

[106] Son J, Lee Y, Park K (2019). Effects of processed red meat consumption on the risk of type 2 diabetes and cardiovascular diseases among Korean adults: the Korean Genome and Epidemiology Study. Eur J Nutr.

[107] Song M, Fung TT, Hu FB, Willett WC, Longo VD, Chan AT, Giovannucci EL (2016). Association of animal and plant protein intake with all-cause and cause-specific mortality. JAMA Intern Med.

[108] Steur M, Johnson L, Sharp SJ, Imamura F, Sluijs I, Key TJ, Wood A, Chowdhury R, Guevara M, Jakobsen MU (2021). Dietary fatty acids, macronutrient substitutions, food sources and incidence of coronary heart disease: findings from the EPIC-CVD case-cohort study across nine European countries. J Am Heart Assoc.

[109] Talaei M, Wang YL, Yuan JM, Pan A, Koh WP (2017). Meat, dietary heme iron, and risk of type 2 diabetes mellitus: the Singapore Chinese Health Study. Am J Epidemiol.

[110] Tharrey M, Mariotti F, Mashchak A, Barbillon P, Delattre M, Fraser GE (2018). Patterns of plant and animal protein intake are strongly associated with cardiovascular mortality: the Adventist Health Study-2 cohort. Int J Epidemiol.

[111] van Nielen M, Feskens EJ, Mensink M, Sluijs I, Molina E, Amiano P, Ardanaz E, Balkau B, Beulens JW, Boeing H (2014). Dietary protein intake and incidence of type 2 diabetes in Europe: the EPIC-InterAct case-cohort study. Diabetes Care.

[112] Veno SK, Bork CS, Jakobsen MU, Lundbye-Christensen S, Bach FW, McLennan PL (2018). Substitution of fish for red meat or poultry and risk of ischemic stroke. Nutrients.

[113] Virtanen HEK, Koskinen TT, Voutilainen S, Mursu J, Tuomainen TP, Kokko P, Virtanen JK (2017). Intake of different dietary proteins and risk of type 2 diabetes in men: the Kuopio Ischaemic Heart Disease Risk Factor Study. Br J Nutr.

[114] Virtanen HEK, Voutilainen S, Koskinen TT, Mursu J, Kokko P, Ylilauri MPT, Tuomainen TP, Salonen JT, Virtanen JK (2019). Dietary proteins and protein sources and risk of death: the Kuopio Ischaemic Heart Disease Risk Factor Study. Am J Clin Nutr.

[115] Virtanen JK, Mursu J, Tuomainen TP, Voutilainen S (2014). Dietary fatty acids and risk of coronary heart disease in men: the Kuopio Ischemic Heart Disease Risk Factor Study. Arterioscler Thromb Vasc Biol.

[116] Vissers LET, Rijksen J, Boer JMA, Verschuren WMM, van der Schouw YT, Sluijs I (2019). Fatty acids from dairy and meat and their association with risk of coronary heart disease. Eur J Nutr.

[117] Voortman T, Chen Z, Girschik C, Kavousi M, Franco OH, Braun KVE (2021). Associations between macronutrient intake and coronary heart disease (CHD): the Rotterdam Study. Clin Nutr.

[118] Wallin A, Di Giuseppe D, Orsini N, Akesson A, Forouhi NG, Wolk A (2017). Fish consumption and frying of fish in relation to type 2 diabetes incidence: a prospective cohort study of Swedish men. Eur J Nutr.

[119] Wang DD, Li Y, Chiuve SE, Stampfer MJ, Manson JE, Rimm EB, Willett WC, Hu FB (2016). Association of specific dietary fats with total and cause-specific mortality. JAMA Intern Med.

[120] Wurtz AM, Hansen MD, Tjonneland A, Rimm EB, Schmidt EB, Overvad K, Jakobsen MU (2016). Substitutions of red meat, poultry and fish and risk of myocardial infarction. Br J Nutr.

[121] Wurtz AML, Hansen MD, Tjonneland A, Rimm EB, Schmidt EB, Overvad K, Jakobsen MU (2021). Replacement of potatoes with other vegetables and risk of myocardial infarction in the Danish Diet, Cancer and Health cohort. Br J Nutr.

[122] Xia PF, Pan XF, Chen C, Wang Y, Ye Y, Pan A (2020). Dietary intakes of eggs and cholesterol in relation to all-cause and heart disease mortality: a prospective cohort study. J Am Heart Assoc.

[123] Xu L, Lam TH, Jiang CQ, Zhang WS, Zhu F, Jin YL, Woo J, Cheng KK, Thomas GN (2019). Egg consumption and the risk of cardiovascular disease and all-cause mortality: Guangzhou Biobank Cohort Study and meta-analyses. Eur J Nutr.

[124] Zhang Y, Zhuang P, Mao L, Chen X, Wang J, Cheng L, Ding G, Jiao J (2019). Current level of fish and omega-3 fatty acid intakes and risk of type 2 diabetes in China. J Nutr Biochem.

[125] Zhong VW, Van Horn L, Cornelis MC, Wilkins JT, Ning H, Carnethon MR, Greenland P, Mentz RJ, Tucker KL, Zhao L (2019). Associations of dietary cholesterol or egg consumption with incident cardiovascular disease and mortality. JAMA.

[126] Zhong VW, Van Horn L, Greenland P, Carnethon MR, Ning H, Wilkins JT, Lloyd-Jones DM, Allen NB (2020). Associations of processed meat, unprocessed red meat, poultry, or fish intake with incident cardiovascular disease and all-cause mortality. JAMA Intern Med.

[127] Zhou C, Zhang Z, Liu M, Zhang Y, Li H, He P, Li Q, Liu C, Qin X (2021). Dietary carbohydrate intake and new-onset diabetes: a nationwide cohort study in China. Metabolism.

[128] Zhuang P, Mao L, Wu F, Wang J, Jiao J, Zhang Y (2020). Cooking oil consumption is positively associated with risk of type 2 diabetes in a Chinese nationwide cohort study. J Nutr.

[129] Zhuang P, Zhang Y, He W, Chen X, Chen J, He L, Mao L, Wu F, Jiao J (2019). Dietary fats in relation to total and cause-specific mortality in a prospective cohort of 521 120 individuals with 16 years of follow-up. Circ Res.

[130] Zong G, Li Y, Sampson L, Dougherty LW, Willett WC, Wanders AJ, Alssema M, Zock PL, Hu FB, Sun Q (2018). Monounsaturated fats from plant and animal sources in relation to risk of coronary heart disease among US men and women. Am J Clin Nutr.

[131] Bellavia A, Stilling F, Wolk A (2016). High red meat intake and all-cause cardiovascular and cancer mortality: is the risk modified by fruit and vegetable intake?. Am J Clin Nutr.

[132] Buijsse B, Boeing H, Drogan D, Schulze MB, Feskens EJ, Amiano P, Barricarte A, Clavel-Chapelon F, de Lauzon-Guillain B, Fagherazzi G (2015). Consumption of fatty foods and incident type 2 diabetes in populations from eight European countries. Eur J Clin Nutr.

[133] Ericson U, Hellstrand S, Brunkwall L, Schulz CA, Sonestedt E, Wallstrom P, Gullberg B, Wirfalt E, Orho-Melander M (2015). Food sources of fat may clarify the inconsistent role of dietary fat intake for incidence of type 2 diabetes. Am J Clin Nutr.

[134] Haring B, Misialek JR, Rebholz CM, Petruski-Ivleva N, Gottesman RF, Mosley TH, Alonso A (2015). Association of dietary protein consumption with incident silent cerebral infarcts and stroke: the Atherosclerosis Risk in Communities (ARIC) study. Stroke.

[135] Tong TYN, Appleby PN, Key TJ, Dahm CC, Overvad K, Olsen A, Tjonneland A, Katzke V, Kuhn T, Boeing H (2020). The associations of major foods and fibre with risks of ischaemic and haemorrhagic stroke: a prospective study of 418 329 participants in the EPIC cohort across nine European countries. Eur Heart J.

[136] Teymoori F, Asghari G, Farhadnejad H, Nazarzadeh M, Atifeh M, Mirmiran P, Azizi F (2020). Various proline food sources and blood pressure: substitution analysis. Int J Food Sci Nutr.

[137] Pan A, Malik VS, Hao T, Willett WC, Mozaffarian D, Hu FB (2013). Changes in water and beverage intake and long-term weight changes: results from three prospective cohort studies. Int J Obes (Lond).

[138] Schmid D, Song M, Zhang X, Willett WC, Vaidya R, Giovannucci EL, Michels KB (2020). Yogurt consumption in relation to mortality from cardiovascular disease, cancer, and all causes: a prospective investigation in 2 cohorts of US women and men. J Biochem.

[139] Bajracharya R, Katzke V, Mukama T, Kaaks R (2023). Effect of iso-caloric substitution of animal protein for other macro nutrients on risk of overall, cardiovascular and cancer mortality: prospective evaluation in EPIC-Heidelberg cohort and systematic review. Nutrients.

[140] Hu Y, Willett WC, Manson JAE, Rosner B, Hu FB, Sun Q (2022). Intake of whole grain foods and risk of coronary heart disease in US men and women. BMC Med.

[141] Orlich MJ, Sabate J, Mashchak A, Fresan U, Jaceldo-Siegl K, Miles F, Fraser GE (2022). Ultra-processed food intake and animal-based food intake and mortality in the Adventist Health Study-2. Am J Clin Nutr.

[142] Stuber JM, Vissers LET, Verschuren WMM, Boer JMA, van der Schouw YT, Sluijs I (2021). Substitution among milk and yogurt products and the risk of incident type 2 diabetes in the EPIC-NL cohort. J Hum Nutr Diet.

[143] Zeng X, Li X, Zhang Z, Li H, Wang Y, Zhu Y, Hu A, Zhao Q, Tang M, Zhang X (2022). A prospective study of carbohydrate intake and risk of all-cause and specific-cause mortality. Eur J Nutr.

[144] Eslamparast T, Sharafkhah M, Poustchi H, Hashemian M, Dawsey SM, Freedman ND, Boffetta P, Abnet CC, Etemadi A, Pourshams A (2017). Nut consumption and total and cause-specific mortality: results from the Golestan Cohort Study. Int J Epidemiol.

[145] Liu W, Hu B, Dehghan M, Mente A, Wang C, Yan R, Rangarajan S, Tse LA, Yusuf S, Liu X (2021). Fruit, vegetable, and legume intake and the risk of all-cause, cardiovascular, and cancer mortality: a prospective study. Clin Nutr.

[146] Xu XY, Kabir A, Barr ML, Schutte AE. Different types of long-term milk consumption and mortality in adults with cardiovascular disease: a population-based study in 7236 Australian adults over 8.4 years. Nutrients. 2022;14(3):704. 10.3390/nu14030704 PMC8839098 35277068

[147] Das A, Cumming R, Naganathan V, Blyth F, Couteur DGL, Handelsman DJ, Waite LM, Ribeiro RVR, Simpson SJ, Hirani V (2022). Associations between dietary intake of total protein and sources of protein (plant vs. animal) and risk of all-cause and cause-specific mortality in older Australian men: the Concord Health and Ageing in Men Project. J Hum Nutr Diet.

[148] Alshahrani SM, Fraser GE, Sabaté J, Knutsen R, Shavlik D, Mashchak A (2019). Red and processed meat and mortality in a low meat intake population. Nutrients.

[149] Hosseini-Esfahani F, Beheshti N, Koochakpoor G, Mirmiran P, Azizi F (2022). Meat food group intakes and the risk of type 2 diabetes incidence. Front Nutr.

[150] Bonaccio M, Ruggiero E, Di Castelnuovo A, Costanzo S, Persichillo M, De Curtis A, Cerletti C, Donati MB, de Gaetano G, Iacoviello L (2017). Fish intake is associated with lower cardiovascular risk in a Mediterranean population: prospective results from the Moli-sani Study. Nutr Metab Cardiovasc Dis.

[151] Liu X, Guasch-Ferré M, Tobias DK, Li Y (2021). Association of walnut consumption with total and cause-specific mortality and life expectancy in U.S. adults. Nutrients.

[152] Ibsen DB, Levitan EB, Akesson A, Gigante B, Wolk A (2022). The DASH diet is associated with a lower risk of heart failure: a cohort study. Eur J Prev Cardiol.

[153] Zheng J, Zhu T, Yang G, Zhao L, Li F, Park Y-M (2022). The isocaloric substitution of plant-based and animal-based protein in relation to aging-related health outcomes: a systematic review. Nutrients.

[154] Guasch-Ferre M, Li Y, Willett WC, Sun  Q, Sampson L, Salas-Salvado J, Martinez-Gonzalez MA, Stampfer MJ, Hu FB (2022). Consumption of olive oil and risk of total and cause-specific mortality among U.S. adults. J Am Coll Cardiol.

[155] Lim CGY, Tai ES, van Dam RM (2022). Replacing dietary carbohydrates and refined grains with different alternatives and risk of cardiovascular diseases in a multi-ethnic Asian population. Am J Clin Nutr.

[156] Thao U, Lajous M, Laouali N, Severi G, Boutron-Ruault MC, MacDonald CJ: Replacing processed red meat with alternative protein sources is associated with a reduced risk of hypertension and diabetes in a prospective cohort of French women. Br J Nutr 2022:1–31. 10.1017/S0007114522002689 36045127

[157] Wang M, Ma H, Song Q, Zhou T, Hu Y, Heianza Y, Manson JE, Qi L (2022). Red meat consumption and all-cause and cardiovascular mortality: results from the UK Biobank study. Eur J Nutr.

[158] Bernstein AM, Sun Q, Hu FB, Stampfer MJ, Manson JE, Willett WC (2010). Major dietary protein sources and risk of coronary heart disease in women. Circulation.

[159] Guasch-Ferre M, Hruby A, Salas-Salvado J, Martinez-Gonzalez MA, Sun Q, Willett WC, Hu FB (2015). Olive oil consumption and risk of type 2 diabetes in US women. Am J Clin Nutr.

[160] Haring B, Gronroos N, Nettleton JA, von Ballmoos MCW, Selvin E, Alonso A (2014). Dietary protein intake and coronary heart disease in a large community based cohort: results from the Atherosclerosis Risk in Communities (ARIC) study. PLoS ONE.

[161] Liu Q, Rossouw JE, Roberts MB, Liu S, Johnson KC, Shikany JM, Manson JE, Tinker LF, Eaton CB (2017). Theoretical effects of substituting butter with margarine on risk of cardiovascular disease. Epidemiology.

[162] Pan A, Sun Q, Bernstein AM, Schulze MB, Manson JE, Stampfer MJ, Willett WC, Hu FB (2012). Red meat consumption and mortality results from 2 prospective cohort studies. Arch Intern Med.

[163] Pan A, Sun Q, Bernstein AM, Schulze MB, Manson JE, Willett WC, Hu FB (2011). Red meat consumption and risk of type 2 diabetes: 3 cohorts of US adults and an updated meta-analysis. Am J Clin Nutr.

[164] Sheehy S, Palmer JR, Rosenberg L (2020). High consumption of red meat is associated with excess mortality among African-American women. J Nutr.

[165] Sun Y, Liu B, Snetselaar LG, Wallace RB, Shadyab AH, Kroenke CH, Haring B, Howard BV, Shikany JM, Valdiviezo C (2021). Association of major dietary protein sources with all-cause and cause-specific mortality: prospective cohort study. J Am Heart Assoc.

[166] Wu H, Flint AJ, Qi Q, van Dam RM, Sampson LA, Rimm EB, Holmes MD, Willett WC, Hu FB, Sun Q (2015). Association between dietary whole grain intake and risk of mortality: two large prospective studies in US men and women. JAMA Intern Med.

[167] Zhang Y, Zhuang P, Wu F, He W, Mao L, Jia W, Zhang Y, Chen X, Jiao J (2021). Cooking oil/fat consumption and deaths from cardiometabolic diseases and other causes: prospective analysis of 521,120 individuals. BMC Med.

[168] Zhuang P, Wu F, Mao L, Zhu F, Zhang Y, Chen X, Jiao J, Zhang Y (2021). Egg and cholesterol consumption and mortality from cardiovascular and different causes in the United States: a population-based cohort study. PLoS Med.

[169] Dominguez LJ, Bes-Rastrollo M, Basterra-Gortari FJ, Gea A, Barbagallo M, Martinez-Gonzalez MA (2018). Should we recommend reductions in saturated fat intake or in red/processed meat consumption? The SUN prospective cohort study. Clin Nutr.

[170] Becerra-Tomas N, Diaz-Lopez A, Rosique-Esteban N, Ros E, Buil-Cosiales P, Corella D, Estruch R, Fito M, Serra-Majem L, Aros F (2018). Legume consumption is inversely associated with type 2 diabetes incidence in adults: a prospective assessment from the PREDIMED study. Clin Nutr.

[171] Ibsen DB, Steur M, Imamura F, Overvad K, Schulze MB, Bendinelli B, Guevara M, Agudo A, Amiano P, Aune D (2020). Replacement of red and processed meat with other food sources of protein and the risk of type 2 diabetes in European populations: the EPIC-InterAct study. Diabetes Care.

[172] Imamura F, Schulze MB, Sharp SJ, Guevara M, Romaguera D, Bendinelli B, Salamanca-Fernandez E, Ardanaz E, Arriola L, Aune D (2019). Estimated substitution of tea or coffee for sugar-sweetened beverages was associated with lower type 2 diabetes incidence in case-cohort analysis across 8 European countries in the EPIC-InterAct study. J Nutr.

[173] O’Connor L, Imamura F, Lentjes MA, Khaw KT, Wareham NJ, Forouhi NG (2015). Prospective associations and population impact of sweet beverage intake and type 2 diabetes, and effects of substitutions with alternative beverages. Diabetologia.

[174] van den Brandt PA (2019). Red meat, processed meat, and other dietary protein sources and risk of overall and cause-specific mortality in the Netherlands Cohort Study. Eur J Epidemiol.

[175] Wurtz AML, Hansen MD, Tjonneland A, Rimm EB, Schmidt EB, Overvad K, Jakobsen MU (2016). Substitution of meat and fish with vegetables or potatoes and risk of myocardial infarction. Br J Nutr.

[176] Farvid MS, Malekshah AF, Pourshams A, Poustchi H, Sepanlou SG, Sharafkhah M, Khoshnia M, Farvid M, Abnet CC, Kamangar F (2017). Dietary protein sources and all-cause and cause-specific mortality: the Golestan Cohort Study in Iran. Am J Prev Med.

[177] Wu F, Mao L, Zhuang P, Chen X, Jiao J, Zhang Y (2020). Plant-sourced cooking oil consumption is associated with lower total mortality in a longitudinal nationwide cohort study. Clin Nutr.

[178] Zhuang P, Jiao J, Wu F, Mao L, Zhang Y (2020). Egg and egg-sourced cholesterol consumption in relation to mortality: findings from population-based nationwide cohort. Clin Nutr.

[179] Li J, Glenn AJ, Yang Q, Ding D, Zheng L, Bao W, Beasley J, LeBlanc E, Lo K, Manson JE (2022). Dietary protein sources, mediating biomarkers, and incidence of type 2 diabetes: findings from the Women’s Health Initiative and the UK Biobank. Diabetes Care.

[180] Schwingshackl L, Knuppel S, Schwedhelm C, Hoffmann G, Missbach B, Stelmach-Mardas M, Dietrich S, Eichelmann F, Kontopantelis E, Iqbal K (2016). Perspective: NutriGrade: a scoring system to assess and judge the meta-evidence of randomized controlled trials and cohort studies in nutrition research. Adv Nutr.

[181] Schwingshackl L, Lampousi AM, Portillo MP, Romaguera D, Hoffmann G, Boeing H (2017). Olive oil in the prevention and management of type 2 diabetes mellitus: a systematic review and meta-analysis of cohort studies and intervention trials. Nutr Diabetes.

[182] Martinez-Gonzalez MA, Sayon-Orea C, Bullon-Vela V, Bes-Rastrollo M, Rodriguez-Artalejo F, Yusta-Boyo MJ, Garcia-Solano M (2022). Effect of olive oil consumption on cardiovascular disease, cancer, type 2 diabetes, and all-cause mortality: a systematic review and meta-analysis. Clin Nutr.

[183] Tutunchi H, Naghshi S, Naemi M, Naeini F, Esmaillzadeh A (2023). Yogurt consumption and risk of mortality from all causes, CVD and cancer: a comprehensive systematic review and dose-response meta-analysis of cohort studies. Public Health Nutr.

[184] Schwingshackl L, Hoffmann G, Iqbal K, Schwedhelm C, Boeing H (2018). Food groups and intermediate disease markers: a systematic review and network meta-analysis of randomized trials. Am J Clin Nutr.

[185] Schwingshackl L, Bogensberger B, Bencic A, Knuppel S, Boeing H, Hoffmann G (2018). Effects of oils and solid fats on blood lipids: a systematic review and network meta-analysis. J Lipid Res.

[186] Misra R, Balagopal P, Raj S, Patel TG (2018). Red meat consumption (heme iron intake) and risk for diabetes and comorbidities?. Curr Diab Rep.

[187] Widmer RJ, Flammer AJ, Lerman LO, Lerman A (2015). The Mediterranean diet, its components, and cardiovascular disease. Am J Med.

[188] Schlesinger S, Neuenschwander M, Schwedhelm C, Hoffmann G, Bechthold A, Boeing H, Schwingshackl L (2019). Food groups and risk of overweight, obesity, and weight gain: a systematic review and dose-response meta-analysis of prospective studies. Adv Nutr.

