# Supplementary material for: Substitution of animal-based with plant-based foods on cardiometabolic health and all-cause mortality: a systematic review and meta-analysis of prospective studies
Source: BMC Med. 2023 Nov 16;21:404. doi: 10.1186/s12916-023-03093-1 (PMC10652524; doi:10.1186/s12916-023-03093-1)
Supplement: Supplementary file 1 — Additional file 1. Search terms. [file 12916_2023_3093_MOESM1_ESM.docx]

**Search terms**

Embase

| \| **#** \| **Searches** \| **Results** \| \| --- \| --- \| --- \| \| 1 \| exp meat/ \| 56801 \| \| 2 \| exp dairy product/ \| 110499 \| \| 3 \| ((((meat or animal* or lamb or beef or pork or chicken or fish or poultry or dairy or milk) not (breast-milk or human-milk)) or egg or eggs or yoghurt* or yogurt* or butter or cheese*) adj9 (intake* or diet* or consum* or nutrion* or food* or eat or eating or meal*)).tw,kw. \| 175280 \| \| 4 \| or/1-3 \| 283801 \| \| 5 \| exp plant protein/ \| 121010 \| \| 6 \| exp nut/ \| 19529 \| \| 7 \| exp soy food/ \| 4265 \| \| 8 \| ((plant* or nonmeat or non-meat or margarine or nut* or fruit or fruits or vegetab* or vegetarian or vegan or potatoe* or legumes or grain* or soy or soya or tofu or bean or beans or pea or peas or lentil or lentils or seed or seeds) adj9 (intake* or diet* or consum* or nutrion* or food* or eat or eating or meal*)).tw,kw. \| 218540 \| \| 9 \| ((other or alternative) adj3 (protein* or fat or fats)).tw,kw. \| 95298 \| \| 10 \| or/5-9 \| 442133 \| \| 11 \| 4 and 10 \| 51544 \| \| 12 \| (substitut* or replac* or exchang*).mp. \| 1538925 \| \| 13 \| 11 and 12 \| 4245 \| \| 14 \| cohort analysis/ \| 778691 \| \| 15 \| follow up/ \| 1767830 \| \| 16 \| longitudinal study/ \| 163979 \| \| 17 \| prospective study/ \| 728524 \| \| 18 \| (prospective or cohort* or observational or longitudinal or follow-up or cases or (case* and control*)).tw,kw. \| 6095331 \| \| 19 \| or/14-18 \| 6679974 \| \| 20 \| 13 and 19 \| 647 \| \| 21 \| **limit 20 to embase** \| **382** \| |
| --- | --- | --- | --- | --- | --- | --- | --- | --- | --- | --- | --- | --- | --- | --- | --- | --- | --- | --- | --- | --- | --- | --- | --- | --- | --- | --- | --- | --- | --- | --- | --- | --- | --- | --- | --- | --- | --- | --- | --- | --- | --- | --- | --- | --- | --- | --- | --- | --- | --- | --- | --- | --- | --- | --- | --- | --- | --- | --- | --- | --- | --- | --- | --- | --- | --- | --- |

Medline via Ovid

| **#** | **Searches** | **Results** |
| --- | --- | --- |
| 1 | exp meat/ | 74741 |
| 2 | exp Dairy Products/ | 101519 |
| 3 | ((((meat or animal$ or lamb or beef or pork or chicken or fish or poultry or dairy or milk) not (breast-milk or human-milk)) or egg or eggs or yoghurt* or yogurt* or butter or cheese*) adj9 (intake* or diet* or consum* or nutrion* or food* or eat or eating or meal*)).ti,ab,kf. | 148374 |
| 4 | or/1-3 | 284802 |
| 5 | exp Plant Proteins/ | 200550 |
| 6 | exp nuts/ | 3674 |
| 7 | exp Soy Foods/ | 7409 |
| 8 | ((plant* or nonmeat or non-meat or margarine or nut* or fruit or fruits or vegetab* or vegetarian or vegan or potatoe* or legumes or grain* or soy or soya or tofu or bean or beans or pea or peas or lentil or lentils or seed or seeds) adj9 (intake* or diet* or consum* or nutrion* or food* or eat or eating or meal*)).ti,ab,kf. | 177077 |
| 9 | ((other or alternative) adj3 (protein* or fat or fats)).ti,ab,kf. | 83890 |
| 10 | or/5-9 | 454948 |
| 11 | 4 and 10 | 38619 |
| 12 | (substitut* or replac* or exchang*).mp. | 1273557 |
| 13 | 11 and 12 | 3350 |
| 14 | cohort Studies/ or follow-up studies/ or longitudinal studies/ or prospective studies/ | 1519581 |
| 15 | (prospective or cohort$ or observational or longitudinal or follow-up or cases or (case$ and control$)).ti,ab,kf. | 4222834 |
| 16 | 14 or 15 | 4706516 |
| 17 | **13 and 16** | **399** |

Web of Science

| **#** | **Searches** | **Results** |
| --- | --- | --- |
| 1 | TI=(((meat OR animal? OR lamb OR beef OR pork OR chicken OR fish OR poultry OR dairy OR milk OR egg OR eggs OR yoghurt* OR yogurt* OR butter OR cheese*) NEAR/9 (intake* OR diet* OR consum* OR nutrion* OR food* OR eat OR eating OR meal*))) OR AB=((((meat OR animal? OR lamb OR beef OR pork OR chicken OR fish OR poultry OR dairy OR milk OR egg OR eggs OR yoghurt* OR yogurt* OR butter OR cheese*) NEAR/9 (intake* OR diet* OR consum* OR nutrion* OR food* OR eat OR eating OR meal*)))) | 218,129 |
| 2 | TI=(((plant OR nonmeat OR non-meat OR margarine OR nut* OR fruit OR fruits OR vegetab* OR vegetarian OR vegan OR potatoe* OR legumes OR grain* OR soy OR soya OR tofu OR bean OR beans OR pea OR peas OR lentil OR lentils OR seed OR seeds) NEAR/9 (intake* OR diet* OR consum* OR nutrion* OR food* OR eat OR eating OR meal*)) ) OR AB=(((plant OR nonmeat OR non-meat OR margarine OR nut* OR fruit OR fruits OR vegetab* OR vegetarian OR vegan OR potatoe* OR legumes OR grain* OR soy OR soya OR tofu OR bean OR beans OR pea OR peas OR lentil OR lentils OR seed OR seeds) NEAR/9 (intake* OR diet* OR consum* OR nutrion* OR food* OR eat OR eating OR meal*))) | 275,813 |
| 3 | TI=(((other OR alternative) NEAR/3 (protein* OR fat OR fats))) OR AB= (((other OR alternative) NEAR/3 (protein* OR fat OR fats))) | 98,864 |
| 4 | #2 OR #3 | 371,931 |
| 5 | #1 AND #4 | 46,132 |
| 6 | TI=(substitut* OR replac* OR exchang*) OR AB=(substitut* OR replac* OR exchang*) | 2,234,352 |
| 7 | #5 AND #6 | 5,813 |
| 8 | TI=(prospective OR cohort? OR observational OR longitudinal OR follow-up OR cases OR (case? AND control?)) OR AB=(prospective OR cohort? OR observational OR longitudinal OR follow-up OR cases OR (case? AND control?)) | 7,231,418 |
| 9 | **#7 AND #8** | [**364**](https://www.webofscience.com/wos/woscc/summary/a1d1a920-4f25-47dc-b913-329e97ac2da3-16c5ac32/relevance/1) |
