# Supplementary material for: Substitution of animal-based with plant-based foods on cardiometabolic health and all-cause mortality: a systematic review and meta-analysis of prospective studies
Source: BMC Med. 2023 Nov 16;21:404. doi: 10.1186/s12916-023-03093-1 (PMC10652524; doi:10.1186/s12916-023-03093-1)
Supplement: Supplementary file 2 — Additional file 2: Table S1. Eligibility criteria by the PICOS statement. Table S2. Study characteristics. Table S3. Description and decision criteria for each domain in ROBINS-I. Table S4. List of excluded studies. Table S5. GRADE assessment for the substitution analyses regarding total CVD. Table S6. GRADE assessment for the substitution analyses regarding CVD mortality. Table S7. GRADE assessment for the substitution analyses regarding CHD incidence. Table S8. GRADE assessment for the substitution analyses regarding total diabetes. Table S9. GRADE assessment for the substitution analyses regarding type 2 diabetes incidence. Table S10. GRADE assessment for the substitution analyses regarding all-cause mortality. [file 12916_2023_3093_MOESM2_ESM.docx]

**Table S1:** Eligibilty criteria by the PICOS statement

|  | **Inclusion criteria** | **Exclusion criteria** |
| --- | --- | --- |
| **P** (population) | General healthy adult population | Specific patient populations, studies on children, adolescents or pregnant women |
| **I** (intervention/exposure) | Plant-based food (e.g. vegetables, fruit, grains, legumes, soy, nuts, seeds and oils) | Supplements or only one particular bioactive plant compound |
| **C** (comparison) | Substitution of animal-based foods (e.g. meat, poultry, fish, shellfish, eggs and dairy) | No substitution of animal-based foods |
| **O** (outcome) | Cardiometabolic health outcomes (type 2 diabetes, cardiovascular disease, coronary heart disease, myocardial infarction, stroke, related morality), all-cause mortality | Biomarkers of cardiometabolic risk (e.g. fasting glucose, blood lipids, etc.) |
| **S** (study design) | Prospective observational studies and randomized controlled trials analysed as observational studies | In vitro/animal experiments, cross-sectional and retrospective case-control studies |

**Table S2:** Study characteristics

| **Author, year, cohort name, country** | **Follow-up (years)** | **Number of participants, sex, age** | **Number of cases** | **Outcome** | **Outcome assessment** | **Exposure assessment** | **Food (unit)** | **Replacement (unit)** | **RR (95% CI)** | **Adjustment factors** |
| --- | --- | --- | --- | --- | --- | --- | --- | --- | --- | --- |
| Al-Shaar, 2020, HPFS,  USA | 30 | 43‘272, M, 53 years | 4456 | CHD | Self-report, validation via medical records, state vital records, National Death Index, reports by next of kin or postal system | Validated, semi-quantitative FFQ | Red meat  (1 serving/d = 4-6 oz)  Unprocessed red meat  (1 serving/d = 4-6 oz)  Processed meat  (1 serving/d = 1 piece/slice or 2oz, 2 slices of bacon) | Nuts  (1 serving/d = small packet or 28g)  Legumes  (1 serving/d = ½ cup)  Soy  (>=2 servings (6-8 oz)/w)  Whole grains  (1 serving/d = 32 g of bread or 200 g cooked brown rice or cereals)  Nuts  (1 serving/d = small packet or 28g)  Legumes  (1 serving/d = ½ cup)  Soy  (>=2 servings (6-8 oz)/w)  Whole grains  (1 serving/d = 32 g of bread or 200 g cooked brown rice or cereals)  Nuts  (1 serving/d = small packet or 28g)  Legumes  (1 serving/d = ½ cup)  Soy  (>=2 servings (6-8 oz)/w)  Whole grains  (1 serving/d = 32 g of bread or 200 g cooked brown rice or cereals) | 0.89 (0.82, 0.96)  0.82 (0.70, 0.96)  0.67 (0.48, 0.93)  0.62 (0.53, 0.73)  0.89 (0.81, 0.98)  0.83 (0.70, 0.99)  0.66 (0.48, 0.92)  0.61 (0.51, 0.72)  0.85 (0.77, 0.94)  0.80 (0.68, 0.95)  0.66 (0.47, 0.91)  0.59 (0.49, 0.69) | Age, year of questionnaire return, race or ethnicity, marital status, living arrangement, profession, work status, smoking,  physical activity, body  mass index, alcohol,  multivitamin use, aspirin use, family history of early  coronary heart disease or stroke, total energy intake. intakes of poultry, fish, egg, combined plant protein sources of nuts, legumes, and soy, whole grains, fruit, vegetables, and  coffee, in addition to total milk, yoghurt, cheese, and other dairy products, and glycemic index |
| Beccera-Tomás, 2018, PREDIMED, Spain | 4,3 | 3349, W/M, 67 years | 266 | T2D incidence | Medical records | Validated, semi-quantitative FFQ | Eggs  (30 g/d)  Fish  (75 g/d)  Meat  ( 75 g/d) | Legumes  (30 g/d) | 0.50 (0.25, 0.99)  0.58 (0.32, 1.05)  0.59 (0.34, 1.03) | Age, sex, intervention group, alcohol intake, total energy intake, smoking,  educational, physical activity, baseline hypertension, hypercholesterolemia, use of lipid-lowering drugs, use of antihypertensive drugs, fasting plasma glucose at baseline, MedDiet adherence and BMI |
| Bernstein, 2010, NHS,  USA | 26 | 84‘134, W, 58 years | 3162 | CHD | Self-report, validated via medical records, state vital records, National Death Index, next of kin or postal system | Validated, semi-quantitative FFQ | Fish  (1 serving/d = 6-8 oz)  Poultry  (1 serving/d = 3-6 oz)  Red meat  (1 serving/d = 4-6 oz) | Nuts  (1 serving/d = small packet or 28g)  Beans  (1 serving/d = ½ cup)  Nuts  (1 serving/d = small packet or 28g)  Beans  (1 serving/d = ½ cup)  Nuts  (1 serving/d = small packet or 28g)  Beans  (1 serving/d = ½ cup) | 0.96 (0.73, 1.27)  0.94 (0.59, 1.50)  0.87 (0.68, 1.11)  0.85 (0.54, 1.34)  0.70 (0.58, 0.83)  0.66 (0.44, 1.01) | Age, calendar time, total energy, cereal fiber, alcohol, trans unsaturated fatty acids, body mass Index, physical activity, smoking, menopausal status, parental history of early myocardial infarction,  years of multivitamin use,  vitamin E supplement use, and aspirin use. |
| Bernstein, 2012, NHS, HPFS,  USA | 22-26 | 127‘160, M/W, 62 years (HPFS), 57 years (NHS) | 4030 | Stroke | Self-report, validated via medical records, state vital records, National Death Index, next of kin or postal system | Validated, semi-quantitative FFQ | Red meat  (1 serving/d = 4-6 oz) | Legumes  (1 serving/d = ½ cup)  Nuts  (1 serving/d = small packet or 28g) | - 1. (0.85, 1.20)   0.83 (0.73, 0.96) | Age, calendar time,included dietary protein sources, total energy,cereal fiber, alcohol, fruit and vegetables, trans unsaturated fatty acids BMI, physical activity, smoking,  menopausal status in women,  parental history of early myocardial infarction, years of multivitamin use, vitamin E  supplement use and aspirin use |
| Ding, 2019, NHS, NHS II; HPFS,  USA | 29-32 | 217‘755, M/W, 50 years (NHS), 36 years (NHS II), 54 years (HPFS) | 51‘438  12‘143 | All-cause mortality  CVD mortality | State vital records, National Death Index, next of kin or postal system, medcal records | Validated, semi-quantitative FFQ | Total dairy  (1 serving/d = e.g. 240 ml of milk)  Total dairy  (1 serving/d = e.g. 240 ml of milk) | Nuts and legumes  (1 serving/d = small packet or 28g/1 oz or 1/2 cup)  Whole grains  (1 serving/d = 32 g of bread or 200 g cooked brown rice or cereals)  Nuts and legumes  (1 serving/d = small packet or 28g/1 oz or 1/2 cup)  Whole grains  (1 serving/d = 32 g of bread or 200 g cooked brown rice or cereals) | 0.86 (0.78, 0.95)  0.89 (0.84, 0.93)  0.89 (0.84, 0.93)  0.93 (0.85, 1.01) | Age, family history of cancer, family history of cardiovascular disease, baseline disease  status (hypertension, hypercholesterolemia), BMI,  physical activity,  AHEI, total energy intake, smoking, alcohol, postmenopausal status and  and current postmenopausal hormone use |
| Dominguez,2018, SUN cohort.  Spain | 9.5 | 18‘540, M/W, 56.4 years | 255 | All-cause mortality | National Death Index, next of kin, professional associations, postal system | Validated, semi-quantitative FFQ | Vegetables  (1 serving/d)  Fruits and nuts  (100 g/d)  Cereals  (100 g/d) | Red meat  (100 g/d) | 1.51 (1.15, 1.97)  1.37 (1.07, 1.75)  1.41 (1.04,1.87) | Age, sex, year of entering the cohort, years of university of education, BMI, smoking, physical activity, hours per day spent watching television, baseline hyper-cholesterolemia, baseline hypertension, history of depression, history of  cardiovascular disease, history of cancer, history of diabetes, prescription of special diets at baseline and snacking between meals. |
| Farvid, 2017, Golestan cohort study,  Iran | 8.1 | 42‘403, M/W, 52 years | 3291 | All-cause mortality | Family members, friends, or local health workers, validated via medical records | Validated FFQ | Legumes  (1 serving/d) | Red meat  (1 serving/d) | 0.68 (0.50, 0.93) | Age, gender, place of residence, marital status, educational level, ethnicity, smoking, opium use, BMI, systolic blood pressure, family history of cancer, physical activity, medication, wealth score, alcohol, and total energy intake |
| Guasch-Ferre, 2015. NHS, NHS II,  USA | 22 | 145‘087, W, 56 years (NHS), 36 years (NHS II) | 9652 | T2D incidence | Self-report, validated via supplementary questionnaire (symptoms, diagnostic tests, treatment), criteria according to National Diabetes Data Group/ADA | Validated, semi-quantitative FFQ | Butter  (8 g/d) | Olive oil  (8 g/d) | 0.92 (0.87, 0.97) | Age, ethnicity, ancestry, smoking, alcohol, physical activity, family history of  diabetes, history of hypertension, history of hypercholesterolemia, multivitamin use, postmenopausal status and menopausal hormone use, AHEI and total energy intake |
| Guasch-Ferre, 2020, HPFS, NHS, USA | 24 | 92‘978, W/M, 66 years | 4229  9797  3802 | CHD  CVD  Stroke | Self-report, validated via medical records, state vital records, National Death Index, next of kin or postal system | Validated, semi-quantitative FFQ | Butter  (5 g/d)  Butter  (5 g/d)  Butter  (5 g/d) | Olive oil  (5 g/d)  Olive oil  (5 g/d)  Olive oil  (5 g/d) | 0.94 (0.89, 1.01)  0.95 (0.91, 1.00)  0.96 (0.89, 1.04) | Age, ethnicity, Southern European/Mediterranean ancestry, smoking, alcohol, physical activity, family history of diabetes, family history of myocardial infarction, family history of cancer, baseline diabetes mellitus, baseline hypertension or antihypertensive medication use, baseline hypercholesterolemia or cholesterol-lowering medication use, multivitamin use, aspirin use, in women postmenopausal status and menopausal hormone use, total energy intake, body mass index, for red meat, fruits and vegetables, nuts, soda, whole grains intake, trans fat, and mutually adjusted for other types of fat |
| Guasch-Ferre, 2022, HPFS, NHS,  USA | 28 | 92‘383, M/W, 56 years (NHS), 57 years (HPFS) | 36‘856  8821 | All-cause mortality  CVD mortality | State vital records, National Death Index, next of kin or postal system, medcal records | Validated, semi-quantitative FFQ | Butter  (10 g/d)  Butter  (10 g/d) | Olive oil  (10 g/d)  Olive oil  (10 g/d) | 0.86 (0.83, 0.88)  0.93 (0.87, 0.98) | Age, ethnicity, ancestry, married, living alone,  smoking, alcohol, physical activity, family history of diabetes, myocardial infarction or cancer, multivitamin use, aspirin use, in women postmenopausal status and menopausal hormone use, energy intake, BMI, red meat, fruits and vegetables, nuts, soda, whole grains, and the intake of trans fat, and mutually adjusted  for the intake of other types of fat. |
| Haring, 2014, ARIC,  USA | 22 | 12‘066, W/M, 54 years | 1147 | CHD | Information from study visits, yearly  telephone follow-up calls, review of hospital discharge lists and medical charts, death certificates, next-of-kin interviews, and  physician-completed questionnaires | 66-item FFQ | Processed meat  (1 serving/d)  Red meat  (1 serving/d)  Poultry  (1 serving/d)  High-fat dairy  (1 serving/d)  Low-fat dairy  (1 serving/d)  Fish/seafood  (1 serving/d)  Eggs  (1 serving/d) | Nuts  (1 serving/d)  Legumes  (1 seriving/d)  Nuts  (1 serving/d)  Legumes  (1 seriving/d)  Nuts  (1 serving/d)  Legumes  (1 seriving/d)  Nuts  (1 serving/d)  Legumes  (1 seriving/d)  Nuts  (1 serving/d)  Legumes  (1 seriving/d)  Nuts  (1 serving/d)  Legumes  (1 seriving/d)  Nuts  (1 serving/d)  Legumes  (1 seriving/d) | 0.94 (0.79, 1.10)  0.97 (0.74, 1.27)  0.81 (0.65, 1.02)   - 1. 0.61, 1.18)   1.00 (0.79, 1.27)  1.04 (0.76, 1.44)  0.90 (0.78, 1.03)  0.93 (0.73, 1.19)  0.97 (0.84, 1.11)   - 1. (0.79, 1.28)   0.95 (0.74, 1.22)  0.99 (0.72, 1.38)   - 1. (0.83, 1.22)   1.05 (0.79, 1.39) | Age, sex, race, study center, total energy intake, smoking, education, systolic blood pressure, use of antihypertensive medication, HDLc, total cholesterol, use of lipid lowering medication, body mass index, waist-to-hip  ratio, alcohol intake, sports-related physical activity, leisure-related physical activity, carbohydrate intake, fiber intake, magnesium intake, and all the food items in the table |
| Ibsen, 2020,  EPIC-Interact, Europe | 12.3 | 26‘460, W/M, 47-61 years | 11‘741 | T2D incidence | Self-report, linkage to primary care, secondary care, drug, hospital admissions and mortality data, verification via medical records | Validated FFQ | Red and processed meat  (50 g/d)  Red meat  (50 g/d)  Processed meat  (50 g/d) | Legumes  (50 g/d)  Nuts  (10 g/d)  Cereals  (30 g/d)  Legumes  (50 g/d)  Nuts  (10 g/d)  Cereals  (30 g/d)  Legumes  (50 g/d)  Nuts  (10 g/d)  Cereals  (30 g/d) | - 1. (0.86, 1.19)   0.90 (0.84, 0.96)  0.92 (0.88, 0.96)  0.99 (0.87, 1.13)  0.91 (0.84, 0.98)  0.94 (0.89, 0.99)   - 1. (0.83, 1.25)   0.88 (0.81, 0.95)  0.89 (0.83, 0.95) | Age, sex, center, education, physical activity, smoking status, total energy intake, alcohol, fruit, vegetables, sweets, soft drinks, coffee, tea, other dairy products, and BMI. |
| Ibsen, 2021, DCH,  Denmark | 10 | 39‘437, W/M, 61.3 years | 3916 | T2D incidence | Danish National Diabetes Register | Validated, semi-quantitative FFQ | Red meat  (100 g/d) | Whole grains  (30 g/d)  Refined grains  /30 g/d) | 0.84 (0.78, 0.91)  0.89 (0.82, 0.96) | Age, sex, total energy, education, alcohol, smoking, physical activity, family history of diabetes, intakes of fruit, vegetables, sugar-sweetened beverages, cpffee, tea, BMI, waist circumference, history of hypertension, history of hypercholesterolemia |
| Imamura, 2019, EPIC-Interact, Europe | 15 | 27‘662, W/M, 52-56 years | 12‘333 | T2D incidence | Self-report, linkage to primary care, secondary care, drug, hospital admissions and mortality data, verification via medical records | Validated FFQ | Sugar-sweetened beverages  (250 g/d)  Fruit juice  (250 g/d)  Milk  (250 g/d)  Milk  (250 g/d) | Milk  (250 g/d)  Milk  (250 g/d)  Coffee  (250 g/d)  Tea  (250 g/d) | 0.91 (0.82, 1.02)   - 1. (0.90, 1.18)   0.85 (0.78, 0.92)  0.82 (0.74, 0.91) | Recruitment centers, age, sex, education, marital status, hormon replacement therapy, menopausal status, history of oral contraceptive use, hypertension, dyslipidemia, familty history of diabetes, prevalent diseases (coronary heart disease and stroke), BMI, waist circumference, smoking, physical activity, alcohol consumption, dietary supplement use, and  dietary consumption (total energy intake, vegetables, fruits, nuts, cheese, yogurt, red meats, processed meats, fish, confectionary, and cereals), mututal adjustment of all beverages |
| Li, 2022, WHI, USA | 15.8 | 108‘681, W, 63 years | 15‘842 | T2D incidence | Self-administered questionnaire, validation studies with use of medical  records and biomarkers conducted | Validated FFQ | Red meat  (50 g/d)    Processed meat  (50 g/d)  Eggs  (50 g/d)  Poultry  (50 g/d) | Whole grains  (30 g/d)  Nuts  (10 g/d)  Legumes  (50 g/d)  Whole grains  (30 g/d)  Nuts  (10 g/d)  Legumes  (50 g/d)  Whole grains  (30 g/d)  Nuts  (10 g/d)  Legumes  (50 g/d)  Whole grains  (30 g/d)  Nuts  (10 g/d)  Legumes  (50 g/d) | 0.87 (0.85, 0.90)  0.92 (0.90, 0.94)  0.95 (0.91, 0.99)  0.79 (0.75, 0.84)  0.84 (0.79, 0.89)   - 1. (0.81, 0.92)   0.78 (0.75, 0.81)  0.82 (0.79, 0.86)  0.85 (0.81, 0.89)   - 1. (0.84, 0.91)   0.92 (0.89, 0.96)  0.95 (0.91, 1.00) | Age, study group indicator, race/ethnicity,  region of residence, family income, education, and family history of diabetes, smoking,  alcohol intake, physical activity, hormone replacement therapy, multivitamin use, antihypertensive  medication use, total energy intake, modified AHEI-2010 score, and intake of other protein sources |
| Li, 2022, UKB, UK | 11.4 | 34‘616, W/M, 58 years | 663 | T2D incidence | Medical records | 24h-recall, repeated five times | Red meat  (50 g/d)  Processed meat  (50 g/d)  Eggs  (50 g/d)  Poultry  (50 g/d) | Whole grains  (30 g/d)  Nuts  (10 g/d)  Legumes  (50 g/d)  Whole grains  (30 g/d)  Nuts  (10 g/d)  Legumes  (50 g/d)  Whole grains  (30 g/d)  Nuts  (10 g/d)  Legumes  (50 g/d)  Whole grains  (30 g/d)  Nuts  (10 g/d)  Legumes  (50 g/d) | 0.80 (0.71, 0.89)  0.81 (0.71, 0.91)  0.91 (0.75, 1.10)  0.70 (0.59, 0.83)  0.71 (0.59, 0.85)  0.80 (0.62, 1.02)  0.84 (0.73, 0.96)  0.85 (0.73, 0.99)  0.96 (0.77, 1.19)  0.84 (0.75, 0.93)  0.84 (0.75, 0.96)  0.95 (0.78, 1.16) | Age, sex, residence area,  Townsend deprivation index at recruitment, smoking status, alcohol, physical activity, family history of diabetes, multivitamin use, antihypertensive medication treatment, total energy  intake, and other protein sources |
| Liu, 2017, WHI,  USA | 13.2 | 69‘223, W, 63.1 years | 4229  5507  1550  1864 | CHD  CVD  Stroke  MI | Medical record review of self-reported hospitalizations and ascertained by the trained physician adjudicator | FFQ | Butter  (1 teaspoon)  Butter  (1 teaspoon)  Butter  (1 teaspoon)  Butter  (1 teaspoon) | Stick margarine  (1 teaspoon)  Tub margarine  (1 teaspoon)  Low fat margarine  (1 teaspoon)  Margarine  (1 teaspoon)  Stick margarine  (1 teaspoon)  Tub margarine  (1 teaspoon)  Low fat margarine  (1 teaspoon)  Margarine  (1 teaspoon)  Stick margarine  (1 teaspoon)  Tub margarine  (1 teaspoon)  Low fat margarine  (1 teaspoon)  Margarine  (1 teaspoon)  Stick margarine  (1 teaspoon)  Tub margarine  (1 teaspoon)  Low fat margarine  (1 teaspoon)  Margarine  (1 teaspoon) | 1.00 (0.99, 1.10)  1.00 (0.97, 1.10)  1.00 (0.97, 1.10)  1.10 (1.00, 1.10)  1.00 (0.99, 1.10)  1.00 (0.97, 1.10)  1.10 (0.97, 1.10)  1.10 (1.00. 1.10)  1.10 (0.97, 1.10)  1.00 (0.93, 1.10)  1.10 (0.93, 1.10)  1.10 (0.97, 1.20)  1.00 (0.97, 1.10)  0.95 (0.89, 1.00)  0.98 (0.90, 1.10)  1.00 (0.94, 1.10) | Age, region, race/ethnicity, income, physical activity, BMI, smoking, total energy, hypertension, family history of MI, postmenopausal hormone use, aspirin use, and hysterectomy. |
| Lim, 2022, MEC, China, Malay  sia, India | 10.1 | 12‘408, W/M, 45.6 years | 746 | CVD | Linkage with data from the National Registry  of Diseases Office, confirmed via medical records | Validated FFQ | Refined grains  (1 serving/d) | Red meat  (1 serving/d)  Poultry  (1 serving/d)  Seafood  (1 serving/d)  Dairy  (1 serving/d)  Egg  (1 serving/d) | 0.87 (0.68, 1.11)  0.93 (0.73, 1.19)  0.95 (0.84, 1.08)  0.90 (0.82, 0.98)  1.02 (0.84, 1.25) | Age, sex, ethnicity, total energy intake, physical activity, smoking, alcohol, educational, history of diabetes, hypertension, and dyslipidemia, family history  of heart disease, menopausal status, BMI, intakes of fiber, cholesterol, and other macronutrients except for carbohydrates |
| Malik, 2016, NHS, NHS II, HPFS,  USA | 20 | 205‘802, W/M, 53 years (HPFS), 50 years (NHS), 36 years (NHS II) | 15‘580 | T2D incidence | Self-report, validated via supplementary questionnaire (symptoms, diagnostic tests, treatment), criteria according to National Diabetes Data Group/ADA | Validated, semi-quantitative FFQ | Dairy  (1 serving/d = e.g. 240 ml/8 oz of milk)  Poultry  (1 serving/d = 3-6 oz)  Eggs  (1 serving/d = 1 egg)  Red meat  (1 serving/d = 4-6 oz)  Fish  (1 serving/d = 6-8 oz)  Processed meat  (1 serving/d = 1 piece/slice or 2oz, 2 slices of bacon)  Dairy  (1 serving/d = e.g. 240 ml/8 oz of milk)  Poultry  (1 serving/d = 3-6 oz)  Eggs  (1 serving/d = 1 egg)  Red meat  (1 serving/d = 4-6 oz)  Fish  (1 serving/d = 6-8 oz)  Processed meat  (1 serving/d = 1 piece/slice or 2oz, 2 slices of bacon) | Peanuts and peanut butter  (1 serving/d = small packet or 28 g)  Whole grain  (1 serving/d = 32 g of bread or 200 g cooked brown rice or cereals) | 0.98 (0.93, 1.03)  0.91 (0.84, 0.98)  0.89 (0.82, 0.97)  0.89 (0.84, 0.95)  0.88 (0.81, 0.97)  0.79 (0.73, 0.85)  0.97 (0.95, 1.00)  0.89 (0.84, 0.94)  0.88 (0.83, 0.94)  0.88 (0.85, 0.92)  0.85 (0.79, 0.92)  0.78 (0.74, 0.83) | Age, family history of  diabetes, smoking, alcohol, physical activity, race/ethnicity, total energy intake, postmenopausal hormone use, oral contraceptive use, intakes of sugar-sweetened beverages, fruit, and vegetables, and  body mass index, other food sources of animal protein, refined grains, and potatoes. |
| O’Connor, 2015, EPIC-Norfolk, UK | 10.8 | 24‘653, W/M, 59 years | 847 | T2D incidence | Self-report, medication brought to follow-up health check, verifcation through record linkage | 7 day food diary | Sweetened-milk beverage  (280 g/d) | Artificially sweetened beverages  (336 g/d)  Drinking water  (280 g/d)  Unsweetened beverages  (280 g/d) | 0.84 (0.67, 1.00)  0.80 (0.67, 0.94)  0.75 (0.63, 0.86) | Age, sex, social class, education, family  history of diabetes, physical activity, smoking, alcohol, season, total energy intake, BMI and waist circumference |
| Pacheco, 2022, NHS, HPFS, USA | 14.2 (NHS)  13,3 (HPFS) | 110‘487, W/M, 53 years | 14‘274  9185  5290 | CVD  CHD  Stroke | Self-report, self-reported incident cases verified through medical records | Validated FFQ | Butter  (40 g/d)  Eggs  (40 g/d)  Yogurt  (40 g/d)  Cheese  (40 g/d)  Processed meat  (40 g/d)  Dairy  (40 g/d)  Butter  (40 g/d)  Eggs  (40 g/d)  Yogurt  (40 g/d)  Cheese  (40 g/d)  Processed meat  (40 g/d)  Dairy  (40 g/d)  Butter  (40 g/d)  Eggs  (40 g/d)  Yogurt  (40 g/d)  Cheese  (40 g/d)  Processed meat  (40 g/d)  Dairy  (40 g/d) | Avocado  (40 g/d) | 0.78 (0.63, 0.96)   - 1. (0.73, 0.93)   0.89 (0.80, 0.99)  0.87 (0.77, 0.98)  0.82 (0.72, 0.94)  0.91 (0.79, 1.05)  0.69 (0.53, 0.90)  0.73 (0.62, 0.85)  0.79 (0.69, 0.90)  0.75 (0.65, 0.87)  0.73 (0.62, 0.86)   - 1. (0.70, 1.01)   0.8(0.63, 1.25)  1.00 (0.83, 1.19)  1.05 (0.91, 1.22)  1.07 (0.90, 1.28)  0.99 (0.80, 1.21)  0.98 (0.79, 1.23) | Age, race, ancestry, alcohol intake, smoking status,  physical activity, family  history of diabetes, family history of myocardial infarction, family history of cancer, baseline diabetes, baseline hypertension or antihypertensive medication use, baseline hypercholesterolemia or cholesterol-lowering  medication use, multivitamin use, aspirin use, postmenopausal status and menopausal hormone use, (only in women), total energy intak, BMI, red and processed meat, fruits and vegetables, nuts, soda, whole grains, eggs, tortilla, breads, cheese intakes, and mutually adjusted for other types of fat-containing foods. |
| Pan, 2011, HPFS, NHS, NHS II,  USA | 16-28 | 204‘157, W/M, 53 years (HPFS), 46 years (NHS), 36 years (NHS II) | 13‘759 | T2D incidence | Self-report, validated via supplementary questionnaire (symptoms, diagnostic tests, treatment), criteria according to National Diabetes Data Group/ADA | Validated, semi-quantitative FFQ | Unprocessed red meat  (1 serving/d = 4-6 oz)  Processed red meat  (1 serving/d = 1 piece/slice or 2oz, 2 slices of bacon)  Red meat  (1 serving/d = 4-6 oz) | Nuts  (1 serving/d = small packet or 28g)  Whole grains  (1 serving/d = 32 g of bread or 200 g cooked brown rice or cereals)  Nuts  (1 serving/d = small packet or 28g)  Whole grains  (1 serving/d = 32 g of bread or 200 g cooked brown rice or cereals)  Nuts  (1 serving/d = small packet or 28g)  Whole grains  (1 serving/d = 32 g of bread or 200 g cooked brown rice or cereals) | 0.80 (0.74, 0.87)  0.76 (0.72, 0.80)  0.68 (0.63, 0.74)  0.65 (0.61, 0.70)  0.79 (0.74, 0.86)  0.77 (0.73, 0.80) | Age, BMI, alcohol, physical activity, smoking, menopausal status and hormone use in women, family history of diabetes, history of hypertension and hypercholesterolemia, total energy intake. |
| Pan, 2012, HPFS, NHS,  USA | 22 (HPFS), 28 (NHS) | 121‘342, M/W,  53 years (HPFS), 46 years (NHS) | 23‘926 | All-cause mortality | State vital records, National Death Index, next of kin or postal system, medcal records | Validated, semi-quantitative FFQ | Unprocessed red meat  (1 serving/d = 4-6 oz)  Processed red meat  (1 serving/d = 1 piece/slice or 2oz, 2 slices of bacon)  Red meat  (1 serving/d = 4-6 oz) | Nuts  (1 serving/d = small packet or 28g)  Legumes  (1 serving/d = ½ cup)  Nuts  (1 serving/d = small packet or 28g)  Legumes  (1 serving/d = ½ cup)  Nuts  (1 serving/d = small packet or 28g)  Legumes  (1 serving/d = ½ cup) | 0.82 (0.78, 0.87)  0.92 (0.87, 0.97)   - 1. (0.68, 0.89)   0.87 (0.79, 0.95)  0.81 (0.77, 0.86)  0.90 (0.86, 0.94) | Age, BMI, alcohol, physical activity, smoking, race, menopausal status and hormone use in women,  family history of diabetes, myocardial infarction, or cancer, history of diabetes,  hypertension, or hypercholesterolemia, total energy intake, and the corresponding two  dietary variables in the models. |
| Schmid, 2020, NHS, HPFS,  USA | 32 (NHS), 26 (HPFS) | 82‘348,  W, 60 years (NHS),  M, 63 years (HPFS) | 20‘831  12‘397 | All-cause mortality | State vital records, National Death Index, next of kin or postal system, medcal records | Validated, semi-quantitative FFQ | Yogurt  (1 cup/d) | Nuts  (1 serving/d = small packet or 28g)  Whole grains  (1 serving/d = 32 g of bread or 200 g cooked brown rice or cereals)  Nuts  (1 serving/d = small packet or 28g)  Whole grains  (1 serving/d = 32 g of bread or 200 g cooked brown rice or cereals) | 0.73 (0.67, 0.79)   - 1. (0.79, 0.94)   0.86 (0.78, 0.95)  0.93 (0.84, 1.02) | Age, 2-y follow-up cycle,  height, BMI, BMI at age 18 y (women) or 21 y (men), ethnicity, physical activity,  smoking, history of hypertension, history of hypercholesterolemia, history of diabetes, history of cancer, family history of diabetes, family history of myocardial infarction, current  multivitamin use, regular aspirin use, menopausal status and hormone use in women, total energyand alcohol |
| Sheehy, 2020, BWHS,  USA | 22 | 56‘314, W,  39 years | 5054 | All-cause mortality | National Death Index, next of kin, the  Social Security Administration Death Master File, and the US Postal  Service | Validated FFQ | Red meat  (1 serving/d) | Vegetables  (1 serving/d)  Whole grains  (1serving/d) | 0.94 (0.90, 0.98)  0.89 (0.83, 0.95) | Age, BMI, education, geographic region, neighborhood socioeconomic status, physical activity, smoking, family history of myocardial infarction, family history of cancer, history of diabetes, history of hypertension, history of hyper-cholesterolemia, total energy intake, and modified 2010 AHEI without red meat (components include alcohol consumption). |
| Sun, 2021, WHI,  USA | 18.1 | 102‘521, W, 63 years | 25‘976  6993 | All-cause mortality  CVD mortality | Death certificates,  medical records, autopsy reports, or by linkage to the National Death Index | Validated FFQ | Red meat  (1 serving/d = 2 oz)  Poultry  (1 serving/d = 2 oz)  Fish  (1 serving/d = 2 oz)  Eggs  (1 serving/d = 2 oz)  Dairy products  (1 serving/d = 2 oz)  Red meat  (1 serving/d = 2 oz)  Poultry  (1 serving/d = 2 oz)  Fish  (1 serving/d = 2 oz)  Eggs  (1 serving/d = 2 oz)  Dairy products  (1 serving/d = 2 oz) | Nuts  (1 serving/d = 2 oz)  Legumes  (1 serving/d = 2 oz)  Nuts  (1 serving/d = 2 oz)  Nuts  (1 serving/d = 2 oz)  Nuts  (1 serving/d = 2 oz)  Nuts  (1 serving/d = 2 oz)  Nuts  (1 serving/d = 2 oz)  Legumes  (1 serving/d = 2 oz)  Nuts  (1 serving/d = 2 oz)  Nuts  (1 serving/d = 2 oz)  Nuts  (1 serving/d = 2 oz)  Nuts  (1 serving/d = 2 oz) | 0.94 (0.90, 0.99)  1.02 (0.96,1.08)  0.97 (0.92, 1.03)  0.98 (0.92, 1.05)  0.72 (0.67, 0.78)  0.94 (0.89,0.99)  0.92 (0.84, 1.01)  1.10 (0.99, 1.23)  0.97 (0.87, 1.08)  0.94 (0.83, 1.06)  0.66 (0.57, 0.76)  0.90 (0.82, 0.99) | Age, race/ethnicity, socioeconomic status, BMI, hormone use history,  lifestyle, baseline health status, family history of heart  attack/stroke, total energy intake, whole grain consumption, vegetable consumption, fruit  consumption, sugar-sweetened  beverage consumption,  and mutual adjustment for other protein sources. |
| Thao, 2022, E3N, France | 18.7 | 71´081, W, 52.8 years | 2681 | Type 2 diabetes | Self-report, validation questionnaires,  and drug reimbursement files from 1993 until last validation of cases in 2012 | Validated FFQ | Red and processed meat (150 g/week) | Pulses (150 g/week)  Vegetables (150 g/week) | 0.96 (0.88, 1.04)  0.93 (0.86, 1.00) | BMI, physical activity, total calories, smoking, total energy intake,  alcohol, sugar sweetened beverage intake, education, family history of  cardiovascular disease, and prevalent hypertension |
| Van den Brandt, 2019. NLCS:  Netherlands | 10 | 12‘025, M/W, 61 years | 8823  2985 | All-cause mortality  CVD mortality | Linkage with Dutch Central Bureau of Genealogy and Statistics Netherlands | Validated, semi-quantitative FFQ | Processed meat  (50 g/d)  Processed meat  (50 g/d) | Nuts  (50 g/d)  Legumes  (50 g/d)  Nuts  (50 g/d)  Legumes  (50 g/d) | 0.65 (0.49, 0.85)  0.94 (0.71, 1.25)  0.62 (0.44, 0.88)  0.82 (0.58, 1.15) | Age, smoking, history of physician-diagnosed hypertension and diabetes, body height, BMI, physical activity,education, alcohol, vegetables and fruit, energy, use of nutritional  supplements, postmenopausal HRT |
| Wang, 2022, UK Biobank Study,  UK | 8.6 | 180‘642, M/W, 55.7 years | 3596  655 | All-cause mortality  CVD mortality | Death certificates held by the National Health Service Information Center in England and Wales and the National Health Service Central Register  in Scotland | Validated FFQ | Red meat  (1 serving/w)  Red meat  (1 serving/w) | Cereal  (1 serving/w)  Cereal  (1 serving/w) | 0.99 (0.96, 1.01)  0.95 (0.90, 1.00) | Age, sex, race, UK Biobank assessment center, average total annual household income, Townsend Deprivation index, alcohol,BMI, physical activity and total energy intake |
| Wu, 2015, HPFS, NHS,  USA | 24-26 | 118‘085, M/W,  50 years (NHS), 53 years (HPFS) | 26‘920  6610 | All-cause mortality  CVD mortality | State vital records, National Death Index, next of kin or postal system, medcal records | Validated, semi-quantitative FFQ | Red meat  (1 serving/d = 4-6 oz)  Red meat  (1 serving/d = 4-6 oz) | Whole grains  (1 serving/d = 32 g of bread or 200 g cooked brown rice or cereals)  Whole grains  (1 serving/d = 32 g of bread or 200 g cooked brown rice or cereals) | 0.90 (0.87, 0.93)  0.80 (0.75, 0.86) | Age, ethnicity, BMI, smoking, alcohol, physical activity, multivitamin use, aspirin use, a family history of heart disease, cancer, or diabetes, and history of hypertension, high cholesterol, or diabetes at baseline, total energy, and modified aHEI (whole grain excluded), menopausal status and postmenopausal hormone use in women |
| Wu, 2020, CHNS,  China | 14 | 14‘305, M/W, 41 years | 1006 | All-cause mortality | Report from family  members | 3 consecutive 24-h recalls,  changes in the home food inventory | Animal cooking oil  (1 tablespoon) | Plant oil  (1 tablespoon)  Peanut oil  (1 tablespoon)  Soybeant oil  (1 tablespoon)  Canola oil  (1 tablespoon)  Salad oil  (1 tablespoon)  Other plant oil  (1 tablespoon) | 0.96 (0.92, 0.998)  0.95 (0.92, 1.001)  0.98 (0.93, 1.03)  0.97 (0.92, 1.03)  0.92 (0.85, 1.001)  0.83 (0.72, 0.94) | Age, gender, BMI. education,  marital status, income, geographical location, and site, physical activity, smoking, alcohol, history of hypertension, history of diabetes, total energy, red meat, white meat,  vegetables, and fruit. |
| Würtz, 2021, HPFS, NHS, NHS II,  USA | 8 | 148‘853, W/M, 53 years (HPFS), 52 years (NHS), 40 years (NHS II) | 8763 | T2D incidence | Self-report, validated via supplementary questionnaire (symptoms, diagnostic tests, treatment), criteria according to National Diabetes Data Group/ADA | Validated, semi-quantitative FFQ | Red meat  (1 serving/d = 4-6 oz)  Unprocessed red meat  (1 serving/d = 4-6 oz)  Processed red meat  (1 serving/d = 1 piece/slice or 2oz, 2 slices of bacon) | Legumes  (1 serving/d = ½ cup)  Nuts  (1 serving/d = small packet or 28g)  Legumes  (1 serving/d = ½ cup)  Nuts  (1 serving/d = small packet or 28g)  Legumes  (1 serving/d = ½ cup)  Nuts  (1 serving/d = small packet or 28g) | 0.93 (0.85, 1.02)  0.85 (0.80, 0.91)  0.93 (0.83, 1.03)  0.86 (0.79, 0.93)  0.85 (0.76, 0.95)  0.79 (0.71, 0.87) | Age, calendar time, energy  marital status, race family history of diabetes, history of hypertension, history of hypercholesterolemia, BMI,  alcohol, modified AHEI, smoking, physical activity,  and for women initial  menopausal status and use of postmenopausal hormones, initial intake of red meat, poultry, seafood, low-fat dairy, high-fat dairy, eggs, legumes, and nuts |
| Würtz, 2016, DCH,  Denmark | 13.6 | 26‘029,  W, 56 years  M, 55 years | 656  1694 | MI | Danish National Patient Register, Danish Cause of Death Register; medical records,  validated according to criteria by the American Heart Association and the European Society of Cardiology | Validated, semi-quantitative FFQ | Red meat  (150 g/w)  Unprocessed red meat  (150 g/w)  Processed red meat  (150 g/w)  Fish  (150 g/w)  Lean fish  (150 g/w)  Fatty fish  (150 g/w)  Poultry  (150 g/w)  Red meat  (150 g/w)  Unprocessed red meat  (150 g/w)  Processed red meat  (150 g/w)  Fish  (150 g/w)  Lean fish  (150 g/w)  Fatty fish  (150 g/w)  Poultry  (150 g/w)  Red meat  (150 g/w)  Unprocessed red meat  (150 g/w)  Processed red meat  (150 g/w)  Fish  (150 g/w)  Lean fish  (150 g/w)  Fatty fish  (150 g/w)  Poultry  (150 g/w)  Red meat  (150 g/w)  Unprocessed red meat  (150 g/w)  Processed red meat  (150 g/w)  Fish  (150 g/w)  Lean fish  (150 g/w)  Fatty fish  (150 g/w)  Poultry  (150 g/w) | Vegetables  (150 g/w)  Potatoes  (150 g/w)  Vegetables  (150 g/w)  Potatoes  (150 g/w) | 0.94 (0.89, 0.99)  0.92 (0.86, 0.99)  0.98 (0.88, 1.09)   - 1. (0.96, 1.13)   0.94 (0.83, 1.06)  1.21 (1.03, 1.42)  0.99 (0.90, 1.10)  0.93 (0.88, 0.98)  0.91 (0.85, 0.98)  0.97 (0.87, 1.08)  1.03 (0.95, 1.12)  0.93 (0.83, 1.05)  1.20 (1.02, 1.41)  0.98 (0.89, 1.09)  0.97 (0.94, 1.00)  0.99 (0.95, 1.02)  0.94 (0.90, 0.98)  0.99 (0.95, 1.03)  0.97 (0.91, 1.04)  1.02 (0.94, 1.11)  0.96 (0.90, 1.01)  0.97 (0.95, 1.00)  0.99 (0.96, 1.02)  0.94 (0.91, 0.98)  1.00 (0.96, 1.04)  0.98 (0.92, 1.04)   - 1. (0.94, 1.11)   0.96 (0.91, 1.01) | Age, total energy, alcohol, BMI, waist circumference, smoking, physical activity, duration of schooling, menopausal status, use of hormone replacement therapy, fruits, sweets, soft drinks, lean dairy products, fatty dairy products, potato chips, refined cereals, wholegrain cereals and nuts. |
| Zhang, 2021, NIH-AARP,  USA | 16 | 521‘120, M/W, 62 years | 129‘328  38‘747  3512 | All-cause mortality  CVD mortality  Diabetes mortality | National Death Index Plus | Validated FFQ | Butter  (1 tablespoon)  Butter  (1 tablespoon)  Butter  (1 tablespoon) | Margarine  (1 tablespoon)  Corn oil  (1 tablespoon)  Canola oil  (1 tablespoon)  Olive oil  (1 tablespoon)  Margarine  (1 tablespoon)  Corn oil  (1 tablespoon)  Canola oil  (1 tablespoon)  Olive oil  (1 tablespoon)  Margarine  (1 tablespoon)  Corn oil  (1 tablespoon)  Canola oil  (1 tablespoon)  Olive oil  (1 tablespoon) | 0.97 (0.96, 0.98)  0.95 (0.93, 0.98)  0.94 (0.92, 0.96)  0.93 (0.91, 0.96)  0.99 (0.97, 1.02)  0.96 (0.92, 1.00)  0.93 (0.89, 0.97)  0.93 (0.89, 0.97)  0.95 (0.88, 1.02)  0.88 (0.77, 1.02)  0.98 (0.86, 1.11)  0.85 (0.73, 0.98) | Age, sex, BMI, race, education, marital status, household income,  smoking, alcohol, physical activity, perceived health condition, history of heart disease, stroke, diabetes, and cancer at baseline, Healthy Eating Index-2015, total energy intake, and consumption of remaining oils where appropriate (butter, margarine, lard, corn oil,  canola oil, olive oil, and other vegetable oils) |
| Zheng, 2019, NHS, HPFS;  USA | 12 | 81‘469, M/W, 60 years | 14‘019 | All-cause mortality | State vital records, National Death Index, next of kin or postal system, medcal records | Validated, semi-quantitative FFQ | Red meat  (1 serving/d = 4-6 oz)  Processed red meat  (1 serving/d = 1 piece/slice or 2oz, 2 slices of bacon)  Unprocessed red meat  (1 serving/d = 4-6 oz) | Nuts  (1 serving/d = small packet or 28g)  Legumes  (1 serving/d = ½ cup)  Whole grains  (1 serving/d = 32 g of bread or 200 g cooked brown rice or cereals)  Vegetables withouth legumes  (1 serving/d)  Nuts  (1 serving/d = small packet or 28g)  Legumes  (1 serving/d = ½ cup)  Whole grains  (1 serving/d = 32 g of bread or 200 g cooked brown rice or cereals)  Vegetables withouth legumes  (1 serving/d)  Nuts  (1 serving/d = small packet or 28g)  Legumes  (1 serving/d = ½ cup)  Whole grains  (1 serving/d = 32 g of bread or 200 g cooked brown rice or cereals)  Vegetables withouth legumes  (1 serving/d) | 0.84 (0.81, 0.88)  0.99 (0.90, 1.08)  0.91 (0.84, 0.98)  0.95 (0.90, 0.99)  0.80 (0.76, 0.86)  0.94 (0.83, 1.06)  0.87 (0.78, 0.96)   - 1. (0.83, 0.99)   0.80 (0.80, 0.88)  0.99 (0.90, 1.09)  0.91 (0.83, 0.99)  0.95 (0.89, 1.01) | Age, race, BMI, family history of myocardial infarction, diabetes, and cancer; updated aspirin use; multivitamin use, menopausal status and use of postmenopausal hormones, smoking, physical activity, alcohol, total energy intake,fruits  and sugar-sweetened beverages. |
| Zhong, 2021, ARIC, CARDIA, CHS, FHS, FOS, MESA,  USA | 19 | 69‘223, M/W, 53.7 years | 5507  8875 | CVD  All-cause mortality | International  Classification of Diseases codes, diagnostic procedures and  review of medical records and/or autopsy data | Validated FFQ | Eggs  (1 egg/d)  Processed meat  (two slices of bacon, two small links of sausage or one hot dog / d)  Unprocessed red meat  (85.05 g/d)  Poultry  (85.05 g/d)  Fish  (85.05 g/d)  Eggs  (1 egg/d)  Processed meat  (two slices of bacon, two small links of sausage or one hot dog / d)  Unprocessed red meat  (85.05 g/d)  Poultry  (85.05 g/d)  Fish  (85.05 g/d) | Nuts  (28.35 g/d)  Legumes  (1/2 cup/d)  Whole grains  (1 cup / d or one slice of whole grain bread /d)  Nuts  (28.35 g/d)  Legumes  (1/2 cup/d)  Whole grains  (1 cup / d or one slice of whole grain bread /d)  Nuts  (28.35 g/d)  Legumes  (1/2 cup/d)  Whole grains  (1 cup / d or one slice of whole grain bread /d)  Nuts  (28.35 g/d)  Legumes  (1/2 cup/d)  Whole grains  (1 cup / d or one slice of whole grain bread /d)  Nuts  (28.35 g/d)  Legumes  (1/2 cup/d)  Whole grains  (1 cup / d or one slice of whole grain bread /d)  Nuts  (28.35 g/d)  Legumes  (1/2 cup/d)  Whole grains  (1 cup / d or one slice of whole grain bread /d)  Nuts  (28.35 g/d)  Legumes  (1/2 cup/d)  Whole grains  (1 cup / d or one slice of whole grain bread /d)  Nuts  (28.35 g/d)  Legumes  (1/2 cup/d)  Whole grains  (1 cup / d or one slice of whole grain bread /d)  Nuts  (28.35 g/d)  Legumes  (1/2 cup/d)  Whole grains  (1 cup / d or one slice of whole grain bread /d)  Nuts  (28.35 g/d)  Legumes  (1/2 cup/d)  Whole grains  (1 cup / d or one slice of whole grain bread /d) | 0.79 (0.71, 0.88)  0.82 (0.72, 0.94)  0.83 (0.77, 0.90)  0.66 (0.53, 0.83)  0.69 (0.55, 0.87)  0.70 (0.57, 0.85)  0.87 (0.79, 0.95)  0.90 (0.80, 1.02)  0.91 (0.86, 0.97)  0.86 (0.78, 0.95)  0.89 (0.78, 1.02)  0.90 (0.84, 1.10)  0.93 (0.84, 1.03)  0.96 (0.84, 1.10)  0.97 (0.90, 1.05)  0.78 (0.72, 0.86)  0.89 (0.80, 1.00)  0.83 (0.78, 0.89)  0.85 (0.78, 0.93)  0.97 (0.87, 1.09)  0.90 (0.85, 0.95)  0.89 (0.82, 0.96)  1.01 (0.90, 1.13)  0.94 (0.89, 0.98)  0.96 (0.88, 1.05)  1.10 (0.98, 1.23)  1.02 (0.95, 1.09)  0.98 (0.90, 1.07)  1.12 (0.99, 1.26)  1.04 (0.97, 1.11) | Age, sex, race and ethnicity  Education, total energy, smoking, physical activity, alcohol, hormone therapy, AHEI, (excluding alcohol, meats, nuts, legumes and whole grains), eggs, processed meat, unprocessed red meat, poultry, fish, nuts, legumes, whole grains, |
| Zhuang, 2020,  CHNS,  China | 15 | 18‘914, M/W, 43 years | 1429 | All-cause mortality | Report from family  members | 3 consecutive 24-h recalls,  changes in the home food inventory | Eggs  (50 g/d) | Nuts/legumes  (50 g/d) | 0.93 (0.88, 0.99) | Age, sex, nationality, marital status, BM, household income, urbanization index, education, physical activity, smoking, alcohol, history of CVD, cancer, diabetes,  and hypertension, total energy intake, consumption of cereals, potatoes, dairy products, nuts/legumes, red meat, white meat, sugar-sweetened beverages, vegetables, and fruit |
| Zhuang, 2021,  NIH-AARP,  USA | 16 | 521‘120, M/W, 62 years | 129‘328  38‘747  3512 | All-cause mortality  CVD mortality  Diabetes mortality | National Death Index Plus | Validated FFQ | Egg  (1 serving = 25 g/d)  Egg  (1 serving = 25 g/d)  Egg  (1 serving = 25 g/d) | Nuts  (25 g/d)  Legumes  (25 g/d)  Nuts  (25 g/d)  Legumes  (25 g/d)  Nuts  (25 g/d)  Legumes  (25 g/d) | 0.87 (0.85, 0.89)  0.90 (0.89, 0.91)   - 1. (0.83, 0.91)   0.91 (0.89, 0.93)  0.83 (0.73, 0.95)  0.89 (0.83, 0.95) | Age, sex, BMI, race, education, marital status, household income, smoking, alcohol, physical activity, history of hypertension, hypercholesteremia, heart disease, stroke, diabetes, and cancer at baseline, total energy intake, intakes of fruit, vegetables, potatoes, whole grains, refined grains, coffee, and sugar-sweetened  beverages |

AHEI: Alternative Healthy Eating Index; ARIC: Atherosclerosis Risk in Communities Study; BMI: body mass index; BWHS: Black Women’s Health Study; CARDIA: The Coronary Artery Risk Development in Young Adults; CHD: coronary heart disease; CHNS: China Health and Nutrition Survey; CHS: Cardiovascular Health Study; CI: confidence interval; CVD: cardiovascular disease; d: day; DCH: Diet, Cancer and Health cohort; EPIC: European Prospective Investigation into Cancer; FFQ: Food-frequency questionnaire; FHS: Framingham Heart Study; FOS: Framingham Offspring Study; HDLc: high-densitiy lipoprotein cholesterol; HPFS: Health Professionals Follow-up Study; HRT: hormone replacement therapy; m: men; MEC: Multi-ethnic cohort; MESA: Multi-Ethnic Study of Atherosclerosis; MI: myocardial infarction; NHS: Nurses‘ Health Study; NIH-AARP: National Institutes of Health-American Association of Retired Persons Diet and Health Study; NLCS: Netherlands Cohort Study; RR: relative risk; SUN: Seguimiento University of Navarra; T2D: type 2 diabetes; UKB: UK biobank study; w: women; WHI: Women’s Health Initiative

**Table S3:** Description and decision criteria for each domain in ROBINS-I

| **Domain** | **Explanation** | **Judgements** |
| --- | --- | --- |
| **Bias due to confounding** | - Is there potential for confounding of the effect of exposure in this study? - Did the authors use a multivariable-adjusted analysis method that controlled at least for age, sex, smoking, physical activity, education/socioeconomic status, alcohol, total energy intake and include mutual adjustment of relevant food groups (at least the substituted food groups)? - Did the authors avoid adjusting for post-exposure variables?   Notes: **Confounding is expected in all observational studies, low risk of bias was not assigned to any study.** Time-varying confounding was expected to be unlikely and is not expected to cause risk of bias in the present study. | Low risk of bias: No bias expected due to confounding, including time-varying confounding.  Moderate risk of bias: Confounding is expected: age, sex, smoking, physical activity, education/socioeconomic status, alcohol, total energy intake have been appropriately controlled for in a multivariable-adjusted analysis and the model included mutual adjustment of relevant food groups (at least the substituted food groups)  *or* confounding is expected: age, sex, smoking, physical activity, alcohol, total energy intake have been appropriately controlled for in a multivariable-adjusted analysis and the model included mutual adjustment of relevant food groups (at least the substituted food groups) *and* education/socioeconomic status is not expected to vary substantially within the cohort (e.g. NHS, HPFS)  *or* the authors statistically investigated whether the confounding domains have an effect on the risk estimate and excluded the confounder from the multivariable model if there was no effect on the overall effect estimate.  Serious risk of bias: At least one known important domain was not measured or appropriately controlled for  No information: No information on which confounders the analysis has been controlled for. |
| **Bias due to selection of participants** | - Was selection of participants into the study based on participants characteristics observed after start of the study/exposure assessment? - Do start of follow-up and start of exposure coincide for most participants? Were methods used that are likely to correct for the presence of selection biases?   Notes: In observational studies, it is unlikely that post-exposure variables influenced selection of participants into the study. Exclusion of participants may be mostly based on missing data, which will be considered in the domain referring to missings (see below). The start of follow-up is considered to coincide with the baseline exposure assessment. However, participants are already exposed at start of the study, which might have influenced outcome measured that occurred shortly after start of the study. | Low risk of bias: All participants who would have been eligible for the target study were included in the study; *and* the authors conducted a sensitivity analysis excluding cases of the respective outcome (e.g. CVD, mortality, type 2 diabetes) which occurred <2 years after the start of the study and the results did not change.  Moderate risk of bias: Selection into the study may have been related to exposure and outcome (e.g. inclusion of postmenopausal women only); *and* the authors used appropriate methods to correct for the selection bias;  *or* the authors conducted no sensitivity analysis excluding cases of the respective outcome (e.g. CVD, mortality, type 2 diabetes) which occurred <2 years after start.  Serious risk of bias: Selection into the study was related to exposure and outcome (e.g. only participants with prediabetes were included in the analysis for type 2 diabetes, or specific patients groups for analysis on mortality); *and* this could not be corrected for in the analyses;  *or* start of follow up and start of exposure do not coincide *and* the rate ratio is not constant over time.  No information: No information is reported about selection of participants into the study. |
| **Bias due to exposure assessment** | - Were exposure groups clearly defined and adequately assessed? - Was the information used to define the exposure groups based on reasonable a priori data?   Note: The start of follow-up is considered to coincide with the baseline exposure assessment. **Any dietary assessment method involves measurement error, thus, no study was assigned low risk of bias.** | Low risk of bias: Exposure status was well defined (substitution per serving, plausible substitution portions (e.g. 100 g meat)); *and* no measurement error is expected in its assessment.  Moderate risk of bias: Exposure status is well defined (substitution per serving, plausible substitution portions); *and* exposure was measured using a validated tool (e.g. a validated FFQ).  Serious risk of bias: Exposure status is not well defined, e.g. implausible substitution portions (e.g. 100 g nuts); *and* exposure was measured using not validated tools.  No information: No definition of exposure or no explanation of the source of information about exposure status is reported. |
| **Bias due to misclassification during follow-up** | - Were there deviations from the exposure beyond what would be expected in usual practice? - Were these deviations unbalanced between groups and likely to have affected the outcome?   Notes: Repeated measurements of the exposure are mostly not available in observational studies. It is not expected that there are high changes in diet in healthy participants. Changes in diet may be similar between studies and may also be similar between groups (differential misclassification is not expected). Recent studies have shown that diet is constant or change only slightly over time (Feldman 2017 IJBNPA, VanWormer 2017 Preventive Medicine Reports). Thus, if repeated measures are not available, moderate risk of bias could be assigned to a study. | Low risk of bias: Repeated measurements of the exposure status during follow-up are available. No or only slight changes were observed and the changes were considered in the analysis.  Moderate risk of bias: Repeated measurements of the exposure are not available, but high changes are not expected during follow-up (compare notes)  or repeated measurements of the exposure status during follow-up are available and some changes in lifestyle factors were observed. The analysis was appropriate to estimate the effect of changes in lifestyle factors, allowing for deviations that were likely to impact on the outcome;  Serious risk of bias: Exposure status is measured during follow-up and high changes in lifestyle factors have been observed, and the analysis was not appropriate to estimate the effect of changes in lifestyle factors, allowing for deviations that were likely to impact on the outcome.  No information: No information on deviations from the exposure is reported. |
| **Bias due to missing data** | - Were there missing outcome data? - Were participants excluded due to missing data on exposure status? - Were participants excluded due to missing data on other variables needed for analysis?   Notes: Missing data on exposure variables and other variables are expected to be missing at random and not related to exposure or outcome that have been assessed during follow-up. | Low risk of bias: Little loss-to-follow-up (<20%) and data on exposure and other variables were reasonably complete (<10% missing data) and was unlikely to introduce bias;  *or* the analysis addressed missing data and is likely to have removed any risk of bias.  Moderate risk of bias: There is a proportion of missing data in the original cohort or a high proportion of loss-to-follow-up; *and* the analysis is unlikely to have removed the risk of bias arising from the missing data (e.g. using logistic regression).  Serious risk of bias: High proportions (>50%) of missing data; *and* the analysis is unlikely to have removed the risk of bias arising from the missing data;  *or* missing data were addressed inappropriately in the analysis;  *or* the nature of the missing data means that the risk of bias cannot be removed through appropriate analysis.  No information: No information is reported about missing data or the potential for data to be missing. |
| **Bias due to measurement of the outcome** | - Could the outcome measure have been influenced by knowledge of the exposure status? - Were the methods of outcome assessment comparable across exposure groups? - Were any systematic error in measurement of the outcome related to exposure status?   Notes: In observational studies, it is not expected that outcome assessors were aware of exposure status of the participants. | Low risk of bias: The methods of outcome assessment were comparable across exposure groups; *and* the outcome measure was unlikely to be influenced by knowledge of the exposure status of study participants; *and* any error in measuring the outcome is unrelated to exposure status (i.e. objective measures such as confirmed medical records, record linkage and death certificates).  Moderate risk of bias: The methods of outcome assessment were comparable across exposure groups; *and* any error in measuring the outcome may be minimally related to exposure status *or* if the outcome measure was not reliable measured (i.e. confirmed records are not available for the whole study population).  Serious risk of bias: The methods of outcome assessment were not comparable across exposure groups;  *or* the outcome measure was subjective (i.e. self-report of CVD, type 2 diabetes, etc. by study participants or next of kin); *and* error in measuring the outcome was related to exposure status.  No information: No information is reported about the methods of outcome assessment. |
| **Bias due to selective reporting of the results** | - Is the reported effect estimate likely to be selected from multiple analyses of exposure-outcome relationship? - Is the reported effect estimate likely to be selected from different subgroups?   Notes: In observational studies, it is unusual to publish an a priori analysis plan or protocol. Multiple outcome measurements for the definition of CVD, mortality, type 2 diabetes, etc. are not expected. | Low risk of bias: There is a clear description of all analysis and the analyses are consistent and all reported results correspond to all intended outcomes, analyses and sub-cohorts.  Moderate risk of bias: The analyses are clearly defined; *and* there is indication of selection of the reported analysis from among multiple analyses; *and* there is indication of selection of the cohort or subgroups for analysis and reporting on basis of the results (e.g. estimates not shown for all analyses).  Serious risk of bias: There is a high risk of selective reporting from among multiple analyses; *or* the cohort or subgroup is selected from a larger study for analysis and appears to be reported based on the results.  No information: There is too little information to make a judgement. |
| **Overall judgement** | Low risk of bias | The study is judged to be at low risk of bias for all domains. |
|  | Moderate risk of bias | The study is judged to be at low or moderate risk of bias for all domains. |
|  | Serious risk of bias | The study is judged to be at serious risk of bias in at least one domain. |
|  | No information | There is no clear indication that the study is at serious risk of bias and there is a lack of information in one or more key domains of bias. |

**Table S4:** List of excluded studies

| Not relevant exposure [12, 34-130] |
| --- |
| No substitution analysis conducted [131-135] |
| Not relevant outcome [136, 137] |
| Conference abstract [138] |
| *Update* |
| Not relevant exposure [139-143] |
| No substitution analysis conducted [144-151] |
| Not relevant outcome [152] |
| Systematic review [153] |
| Already included [154-157] |

**Table S5:** GRADE assessment for the substitution analyses regarding total CVD

| **Certainty assessment** | | | | | | | **No of participants** | **No of cases** | **Effect** | | **Certainty** |
| --- | --- | --- | --- | --- | --- | --- | --- | --- | --- | --- | --- |
| **№ of studies** | **Study design** | **Risk of bias** | **Inconsistency** | **Indirectness** | **Imprecision** | **Other considerations** |  |  | **Relative (95% CI)** | **Absolute (95% CI)*** |  |
| **Red meat replaced by nuts** | | | | | | | | | | | |
| 4 | observational studies | serious^a^ | not serious | not serious | serious^b^ | none | 241‘995 | 15‘758 | **RR 0.92** (0.88 to 0.97) | **5 fewer per 1.000** (from 8 fewer to 2 fewer) | ⨁⨁◯◯ Low |
| **Red meat replaced by legumes** | | | | | | | | | | | |
| 4 | observational studies | serious^a^ | serious^c^ | not serious | serious^b^ | none | 241‘995 | 15‘758 | **RR 0.95** (0.86 to 1.05) | **3 fewer per 1.000** (from 9 fewer to 3 more) | ⨁◯◯◯ Very low |
| **Red meat replaced by whole grains** | | | | | | | | | | | |
| 8 | observational studies | serious^a^ | serious^c^ | not serious | serious^b^ | none | 147‘767 | 13‘573 | **RR 0.93** (0.91 to 0.96) | **4 fewer per 1.000** (from 8 fewer to 1 fewer) | ⨁◯◯◯ Very low |
| **Processed meat replaced by nuts** | | | | | | | | | | | |
| 8 | observational studies | serious^a^ | not serious | not serious | not serious | none | 84‘979 | 14‘404 | **RR 0.73** (0.59 to 0.91) | **46 fewer per 1.000** (from 69 fewer to 15 fewer) | ⨁⨁⨁◯ Moderate |
| **Processed meat replaced by legumes** | | | | | | | | | | | |
| 8 | observational studies | serious^a^ | not serious | not serious | not serious | none | 84‘979 | 14‘404 | **RR 0.77** (0.68 to 0.87) | **39 fewer per 1.000** (from 54 fewer to 22 fewer) | ⨁⨁⨁◯ Moderate |
| **Processed meat replaced by whole grains** | | | | | | | | | | | |
| 7 | observational studies | serious^a^ | not serious | not serious | not serious | none | 72‘954 | 11‘419 | **RR 0.64** (0.54 to 0.75) | **56 fewer per 1.000** (from 72 fewer to 39 fewer) | ⨁⨁⨁◯ Moderate |
| **Unprocessed red meat replaced by nuts** | | | | | | | | | | | |
| 7 | observational studies | serious^a^ | not serious | not serious | serious^b^ | none | 72‘954 | 11‘419 | **RR 0.95** (0.91 to 0.98) | **8 fewer per 1.000** (from 14 fewer to 3 fewer) | ⨁⨁◯◯ Low |
| **Unprocessed red meat replaced by legumes** | | | | | | | | | | | |
| 7 | observational studies | serious^a^ | not serious | not serious | serious^b^ | none | 72‘954 | 11‘419 | **RR 0.94** (0.90 to 0.99) | **9 fewer per 1.000** (from 16 fewer to 2 fewer) | ⨁⨁◯◯ Low |
| **Unprocessed red meat replaced by whole grains** | | | | | | | | | | | |
| 7 | observational studies | serious^a^ | serious^c^ | not serious | serious^b^ | none | 72‘954 | 11‘419 | **RR 0.90** (0.78 to 1.01) | **16 fewer per 1.000** (from 33 fewer to 2 more) | ⨁◯◯◯ Very low |
| **Poultry replaced by nuts** | | | | | | | | | | | |
| 8 | observational studies | serious^a^ | not serious | not serious | serious^b^ | none | 216‘339 | 17‘118 | **RR 0.94** (0.89 to 0.98) | **5 fewer per 1.000** (from 9 fewer to 2 fewer) | ⨁⨁◯◯ Low |
| **Poultry replaced by legumes** | | | | | | | | | | | |
| 7 | observational studies | serious^a^ | not serious | not serious | serious^b^ | none | 113‘818 | 10‘125 | **RR 0.93** (0.87 to 1.00) | **6 fewer per 1.000** (from 12 fewer to 0 fewer) | ⨁⨁◯◯ Low |
| **Fish replaced by nuts** | | | | | | | | | | | |
| 8 | observational studies | serious^a^ | not serious | not serious | serious^b^ | none | 216‘339 | 17‘118 | **RR 0.97** (0.93 to 1.01) | **2 fewer per 1.000** (from 6 fewer to 1 more) | ⨁⨁◯◯ Low |
| **Fish replaced by legumes** | | | | | | | | | | | |
| 7 | observational studies | serious^a^ | not serious | not serious | serious^b^ | none | 113‘818 | 10‘125 | **RR 0.98** (0.92 to 1.05) | **2 fewer per 1.000** (from 7 fewer to 4 more) | ⨁⨁◯◯ Low |
| **Dairy replaced by nuts/legumes** | | | | | | | | | | | |
| 5 | observational studies | serious^a^ | not serious^d^ | serious^e^ | not serious | none | 332‘342 | 20‘283 | **RR 0.97** (0.96 to 0.99) | **2 fewer per 1.000** (from 2 fewer to 1 fewer) | ⨁⨁◯◯ Low |
| **Dairy replaced by nuts** | | | | | | | | | | | |
| 2 | observational studies | serious^a^ | not serious^d^ | not serious | serious^b^ | none | 114‘587 | 8140 | **RR 0.95** (0.88 to 1.03) | **4 fewer per 1.000** (from 9 fewer to 2 more) | ⨁⨁◯◯ Low |
| **Dairy replaced by whole / refined grains** | | | | | | | | | | | |
| 4 | observational studies | serious^a^ | serious^c^ | serious^f^ | not serious | none | 230‘163 | 12‘889 | **RR 1.00** (0.96 to 1.05) | **0 fewer per 1.000** (from 2 fewer to 3 more) | ⨁◯◯◯ Very low |
| **Eggs replaced by nuts** | | | | | | | | | | | |
| 8 | observational studies | serious^a^ | not serious | not serious | not serious | none | 653‘323 | 52‘703 | **RR 0.83** (0.78 to 0.89) | **14 fewer per 1.000** (from 18 fewer to 9 fewer) | ⨁⨁⨁◯ Moderate |
| **Eggs replaced by legumes** | | | | | | | | | | | |
| 7 | observational studies | serious^a^ | not serious | not serious | serious^b^ | none | 550‘802 | 45‘710 | **RR 0.88** (0.80 to 0.97) | **10 fewer per 1.000** (from 17 fewer to 2 fewer) | ⨁⨁◯◯ Low |
| **Butter replaced by margarine** | | | | | | | | | | | |
| 2 | observational studies | serious^a^ | serious^c^ | not serious | serious^b^ | none | 590‘343 | 42‘976 | **RR 1.03** (0.95 to 1.12) | **2 more per 1.000** (from 4 fewer to 9 more) | ⨁◯◯◯ Very low |
| **Butter replaced by olive oil** | | | | | | | | | | | |
| 3 | observational studies | serious^a^ | not serious | not serious | not serious | none | 614‘098 | 54‘578 | **RR 0.96** (0.94 to 0.98) | **3 fewer per 1.000** (from 4 fewer to 2 fewer) | ⨁⨁⨁◯ Moderate |

*****Absolute risks were calculated in GRADEpro (<https://www.gradepro.org/>) as the difference between the baseline risk of an outcome (e.g., in persons receiving control intervention or estimated in the observational studies) and the risk of outcome after the intervention is applied, i.e. the risk of an outcome in persons who were exposed or received an intervention. The calculation of the absolute effect was based on the relative magnitude of an effect and baseline risk. Absolute effect calculation option is selected by default and GRADEpro calculates the absolute effect risk once at least one of the baseline risk values is provided and the magnitude of the relative effect (RR, OR or HR) has been entered.

**CI:** confidence interval; CVD: cardiovascular disease; **RR:** risk ratio

#### Explanations

a. Downgraded by one level for risk of bias, since all studies were rated with a moderate risk of bias.

b. Downgraded by one level for imprecision since 95% CI crosses threshold of a minimal important difference (absolute risk: 5 fewer and/or 5 more events per 1.000).

c. Downgraded by one level for inconsistency since point estimates and 95% CI did not fully overlap between studies and ^I2^ was substantial.

d. Not downgraded for inconsistency although 95% CI did not overlap between studies and I^2^ was high, but differences in unit of replacement may explain inconsistency.

e. Downgraded by one level for indirectness since the replacing food category was in one study "nuts", whereas in the other studies "nuts and legumes".

f. Downgraded by one level for indirectness since the replacing food category was in one study "whole grains", whereas in the other study "refined grains".

**Table S6:** GRADE assessment for the substitution analyses regarding CVD mortality

| **Certainty assessment** | | | | | | | **No of participants** | **No of cases** | **Effect** | | **Certainty** |
| --- | --- | --- | --- | --- | --- | --- | --- | --- | --- | --- | --- |
| **№ of studies** | **Study design** | **Risk of bias** | **Inconsistency** | **Indirectness** | **Imprecision** | **Other considerations** |  |  | **Relative (95% CI)** | **Absolute* (95% CI)** |  |
| **Red and processed meat replaced by nuts** | | | | | | | | | | | |
| 2 | observational studies | serious^a^ | serious^b^ | not serious | serious^c^ | none | 114‘546 | 9978 | **RR 0.78** (0.54 to 1.15) | **19 fewer per 1.000** (from 40 fewer to 13 more) | ⨁◯◯◯ Very low |
| **Red meat replaced by whole grains / cereals** | | | | | | | | | | | |
| 3 | observational studies | serious^a^ | serious^b^ | not serious | not serious | none | 298‘727 | 7265 | **RR 0.94** (0.90 to 1.00) | **1 fewer per 1.000** (from 2 fewer to 0 fewer) | ⨁⨁◯◯ Low |
| **Eggs replaced by nuts** | | | | | | | | | | | |
| 2 | observational studies | serious^a^ | not serious | not serious | not serious | none | 623‘641 | 45‘740 | **RR 0.84** (0.79 to 0.90) | **12 fewer per 1.000** (from 15 fewer to 7 fewer) | ⨁⨁⨁◯ Moderate |
| **Butter replaced by olive oil** | | | | | | | | | | | |
| 3 | observational studies | serious^a^ | not serious | not serious | not serious | none | 613‘503 | 47‘568 | **RR 0.96** (0.94 to 0.98) | **3 fewer per 1.000** (from 5 fewer to 2 fewer) | ⨁⨁⨁◯ Moderate |

******Absolute risks were calculated in GRADEpro (<https://www.gradepro.org/>) as the difference between the baseline risk of an outcome (e.g., in persons receiving control intervention or estimated in the observational studies) and the risk of outcome after the intervention is applied, i.e. the risk of an outcome in persons who were exposed or received an intervention. The calculation of the absolute effect was based on the relative magnitude of an effect and baseline risk. Absolute effect calculation option is selected by default and GRADEpro calculates the absolute effect risk once at least one of the baseline risk values is provided and the magnitude of the relative effect (RR, OR or HR) has been entered.

**CI:** confidence interval; CVD: cardiovascular disease; **RR:** risk ratio

#### Explanations

a. Downgraded by one level for risk of bias, since all studies were rated with a moderate risk of bias.

b. Downgraded by one level for inconsistency since point estimates and 95% CI did not fully overlap between studies and I^2^ was substantial.

c. Downgraded by one level for imprecision since 95% CI crosses threshold of a minimal important difference (absolute risk: 5 fewer and/or 5 more events per 1.000).

**Table S7:** GRADE assessment for the substitution analyses regarding CHD incidence

| **Certainty assessment** | | | | | | | **No of participants** | **No of cases** | **Effect** | | **Certainty** |
| --- | --- | --- | --- | --- | --- | --- | --- | --- | --- | --- | --- |
| **№ of studies** | **Study design** | **Risk of bias** | **Inconsistency** | **Indirectness** | **Imprecision** | **Other considerations** |  |  | **Relative (95% CI)** | **Absolute* (95% CI)** |  |
| **Red meat replaced by nuts** | | | | | | | | | | | |
| 3 | observational studies | serious^a^ | not serious | not serious | serious^b^ | none | 139‘474 | 8765 | **RR 0.93** (0.88 to 0.98) | **4 fewer per 1.000** (from 8 fewer to 1 fewer) | ⨁⨁◯◯ Low |
| **Red meat replaced by legumes** | | | | | | | | | | | |
| 3 | observational studies | serious^a^ | not serious | not serious | serious^b^ | none | 139‘474 | 8765 | **RR 0.92** (0.88 to 0.97) | **5 fewer per 1.000** (from 8 fewer to 2 fewer) | ⨁⨁◯◯ Low |
| **Processed meat replaced by nuts** | | | | | | | | | | | |
| 2 | observational studies | serious^a^ | not serious | not serious | not serious | none | 55‘338 | 5603 | **RR 0.87** (0.80 to 0.95) | **13 fewer per 1.000** (from 20 fewer to 5 fewer) | ⨁⨁⨁◯ Moderate |
| **Processed meat replaced by legumes** | | | | | | | | | | | |
| 2 | observational studies | serious^a^ | not serious | not serious | serious^b^ | none | 55‘338 | 5603 | **RR 0.85** (0.71 to 1.02) | **15 fewer per 1.000** (from 29 fewer to 2 more) | ⨁⨁◯◯ Low |
| **Poultry replaced by nuts** | | | | | | | | | | | |
| 2 | observational studies | serious^a^ | not serious | not serious | not serious | none | 96‘202 | 4309 | **RR 0.97** (0.90 to 1.04) | **1 fewer per 1.000** (from 4 fewer to 2 more) | ⨁⨁⨁◯ Moderate |
| **Poultry replaced by legumes** | | | | | | | | | | | |
| 2 | observational studies | serious^a^ | not serious | not serious | serious^b^ | none | 96‘202 | 4309 | **RR 0.98** (0.87 to 1.11) | **1 fewer per 1.000** (from 6 fewer to 5 more) | ⨁⨁◯◯ Low |
| **Fish / seafood replaced by nuts** | | | | | | | | | | | |
| 2 | observational studies | serious^a^ | not serious | not serious | not serious | none | 96‘202 | 4309 | **RR 0.99** (0.95 to 1.04) | **0 fewer per 1.000** (from 2 fewer to 2 more) | ⨁⨁⨁◯ Moderate |
| **Fish / seafood replaced by legumes** | | | | | | | | | | | |
| 2 | observational studies | serious^a^ | not serious | not serious | not serious | none | 96‘202 | 4309 | **RR 0.99** (0.93 to 1.06) | **0 fewer per 1.000** (from 3 fewer to 3 more) | ⨁⨁⨁◯ Moderate |

*****Absolute risks were calculated in GRADEpro (<https://www.gradepro.org/>) as the difference between the baseline risk of an outcome (e.g., in persons receiving control intervention or estimated in the observational studies) and the risk of outcome after the intervention is applied, i.e. the risk of an outcome in persons who were exposed or received an intervention. The calculation of the absolute effect was based on the relative magnitude of an effect and baseline risk. Absolute effect calculation option is selected by default and GRADEpro calculates the absolute effect risk once at least one of the baseline risk values is provided and the magnitude of the relative effect (RR, OR or HR) has been entered.

**CI:** confidence interval; **CHD**: coronary heart disease; **RR:** risk ratio

#### Explanations

a. Downgraded by one level for risk of bias, since all studies were rated with a moderate risk of bias.

b. Downgraded by one level for imprecision since 95% CI crosses threshold of a minimal important difference (absolute risk: 5 fewer and/or 5 more events per 1.000).

**Table S8:** GRADE assessment for the substitution analyses regarding total diabetes

| **Certainty assessment** | | | | | | | **No of participants** | **No of cases** | **Effect** | | **Certainty** |
| --- | --- | --- | --- | --- | --- | --- | --- | --- | --- | --- | --- |
| **№ of studies** | **Study design** | **Risk of bias** | **Inconsistency** | **Indirectness** | **Imprecision** | **Other considerations** |  |  | **Relative (95% CI)** | **Absolute^*^ (95% CI)** |  |
| **Eggs replaced by nuts** | | | | | | | | | | | |
| 3 | observational studies | serious^a^ | not serious | serious^b^ | not serious | none | 664‘417 | 20‘017 | **RR 0.82**  (0.79 to 0.85) | **5 fewer per 1.000** (from 6 fewer to 5 fewer) | ⨁⨁◯◯ Low |
| **Eggs replaced by legumes** | | | | | | | | | | | |
| 4 | observational studies | serious^a^ | not serious | not serious | serious^c^ | none | 667‘766 | 20‘283 | **RR 0.84** (0.76 to 0.93) | **5 fewer per 1.000** (from 7 fewer to 2 fewer) | ⨁⨁◯◯ Low |
| **Butter replaced by olive oil** | | | | | | | | | | | |
| 3 | observational studies | serious^a^ | not serious | not serious | not serious | none | 666‘207 | 13‘164 | **RR 0.94** (0.91 to 0.98) | **1 fewer per 1.000** (from 2 fewer to 0 fewer) | ⨁⨁⨁◯ Moderate |

*****Absolute risks were calculated in GRADEpro (<https://www.gradepro.org/>) as the difference between the baseline risk of an outcome (e.g., in persons receiving control intervention or estimated in the observational studies) and the risk of outcome after the intervention is applied, i.e. the risk of an outcome in persons who were exposed or received an intervention. The calculation of the absolute effect was based on the relative magnitude of an effect and baseline risk. Absolute effect calculation option is selected by default and GRADEpro calculates the absolute effect risk once at least one of the baseline risk values is provided and the magnitude of the relative effect (RR, OR or HR) has been entered.

**CI:** confidence interval; **RR:** risk ratio

#### Explanations

a. Downgraded by one level for risk of bias, since all studies were rated with a moderate risk of bias

b. Downgraded by one level for indirectness, since replacement unit ranges from 10g/d to 50 g/d

c. Downgraded by one level for imprecision since 95% CI crosses threshold of a minimal important difference (absolute risk: 5 fewer and/or 5 more events per 1.000).

**Table S9:** GRADE assessment for the substitution analyses regarding type 2 diabetes incidence

| **Certainty assessment** | | | | | | | **No of partcipants** | **No of cases** | **Effect** | | **Certainty** |
| --- | --- | --- | --- | --- | --- | --- | --- | --- | --- | --- | --- |
| **№ of studies** | **Study design** | **Risk of bias** | **Inconsistency** | **Indirectness** | **Imprecision** | **Other considerations** |  |  | **Relative (95% CI)** | **Absolute* (95% CI)** |  |
| **Red meat replaced by nuts** | | | | | | | | | | | |
| 6 | observational studies | serious^a^ | not serious | not serious | not serious | none | 373‘914 | 42‘005 | **RR 0.92** (0.90 to 0.94) | **9 fewer per 1.000** (from 11 fewer to 7 fewer) | ⨁⨁⨁◯ Moderate |
| **Red meat replaced by legumes** | | | | | | | | | | | |
| 3 | observational studies | serious^a^ | not serious | not serious | serious^b^ | none | 169‘757 | 28‘246 | **RR 0.95** (0.92 to 0.99) | **8 fewer per 1.000** (from 13 fewer to 2 fewer) | ⨁⨁◯◯ Low |
| **Red meat replaced by whole grains / cereals** | | | | | | | | | | | |
| 6 | observational studies | serious^a^ | not serious^c^ | not serious | not serious | none | 375‘559 | 43‘826 | **RR 0.90** (0.84 to 0.96) | **12 fewer per 1.000** (from 19 fewer to 5 fewer) | ⨁⨁⨁◯ Moderate |
| **Processed meat replaced by nuts** | | | | | | | | | | | |
| 6 | observational studies | serious^a^ | not serious^c^ | not serious | not serious | none | 373‘914 | 42‘005 | **RR 0.78** (0.69 to 0.88) | **25 fewer per 1.000** (from 35 fewer to 13 fewer) | ⨁⨁⨁◯ Moderate |
| **Processed meat replaced by legumes** | | | | | | | | | | | |
| 3 | observational studies | serious^a^ | not serious | not serious | serious^b^ | none | 169‘757 | 28‘246 | **RR 0.88** (0.79 to 0.98) | **20 fewer per 1.000** (from 35 fewer to 3 fewer) | ⨁⨁◯◯ Low |
| **Processed meat replaced by whole grains / cereals** | | | | | | | | | | | |
| 6 | observational studies | serious^a^ | serious^d^ | not serious | not serious | none | 375‘559 | 43‘826 | **RR 0.80** (0.74 to 0.87) | **23 fewer per 1.000** (from 30 fewer to 15 fewer) | ⨁⨁◯◯ Low |
| **Poultry replaced by nuts** | | | | | | | | | | | |
| 2 | observational studies | serious^a^ | not serious | not serious | serious^b^ | none | 143‘297 | 16‘505 | **RR 0.90** (0.83 to 0.97) | **12 fewer per 1.000** (from 20 fewer to 3 fewer) | ⨁⨁◯◯ Low |
| **Poultry replaced by legumes** | | | | | | | | | | | |
| 2 | observational studies | serious^a^ | not serious | not serious | serious^b^ | none | 143‘297 | 16‘505 | **RR 0.95** (0.91 to 0.99) | **6 fewer per 1.000** (from 10 fewer to 1 fewer) | ⨁⨁◯◯ Low |
| **Poultry replaced by whole grains** | | | | | | | | | | | |
| 2 | observational studies | serious^a^ | not serious | not serious | not serious | none | 143‘297 | 16‘505 | **RR 0.87** (0.83 to 0.90) | **15 fewer per 1.000** (from 20 fewer to 12 fewer) | ⨁⨁⨁◯ Moderate |
| **Eggs replaced by nuts** | | | | | | | | | | | |
| 2 | observational studies | serious^a^ | not serious | not serious | not serious | none | 143‘297 | 16‘505 | **RR 0.82** (0.79 to 0.86) | **21 fewer per 1.000** (from 24 fewer to 16 fewer) | ⨁⨁⨁◯ Moderate |
| **Eggs replaced by legumes** | | | | | | | | | | | |
| 3 | observational studies | serious^a^ | not serious | not serious | serious^b^ | none | 146‘646 | 16‘771 | **RR 0.86** (0.72 to 1.04) | **16 fewer per 1.000** (from 32 fewer to 5 more) | ⨁⨁◯◯ Low |
| **Eggs replaced by whole grains** | | | | | | | | | | | |
| 2 | observational studies | serious^a^ | not serious | not serious | not serious | none | 143‘297 | 16‘505 | **RR 0.79** (0.75 to 0.82) | **24 fewer per 1.000** (from 29 fewer to 21 fewer) | ⨁⨁⨁◯ Moderate |

*****Absolute risks were calculated in GRADEpro (<https://www.gradepro.org/>) as the difference between the baseline risk of an outcome (e.g., in persons receiving control intervention or estimated in the observational studies) and the risk of outcome after the intervention is applied, i.e. the risk of an outcome in persons who were exposed or received an intervention. The calculation of the absolute effect was based on the relative magnitude of an effect and baseline risk. Absolute effect calculation option is selected by default and GRADEpro calculates the absolute effect risk once at least one of the baseline risk values is provided and the magnitude of the relative effect (RR, OR or HR) has been entered.

**CI:** confidence interval; **RR:** risk ratio

#### Explanations

a. Downgraded by one level for risk of bias, since all studies were rated with a moderate risk of bias.

b. Downgraded by one level for imprecision since 95% CI crosses threshold of a minimal important difference (absolute risk: 5 fewer and/or 5 more events per 1.000).

c. Not downgraded for inconsistency although 95% CI did not overlap between studies and I^2^ was very high, but differences in unit of replacement may explain inconsistency.

d. Downgraded by one level for inconsistency since point estimates and 95% CI did not fully overlap between studies and I^2^ was substantial.

**Table S10:** GRADE assessment for the substitution analyses regarding all-cause mortality

| **Certainty assessment** | | | | | | | **No of participants** | **No of cases** | **Effect** | | **Certainty** |  |
| --- | --- | --- | --- | --- | --- | --- | --- | --- | --- | --- | --- | --- |
| **№ of studies** | **Study design** | **Risk of bias** | **Inconsistency** | **Indirectness** | **Imprecision** | **Other considerations** |  |  | **Relative (95% CI)** | **Absolute* (95% CI)** |  |  |
| **Red meat replaced by nuts** | | | | | | | | | | | | |
| 9 | observational studies | serious^a^ | not serious | not serious | not serious | none | 253‘545 | 58‘777 | **RR 0.93** (0.91 to 0.95) | **16 fewer per 1.000** (from 21 fewer to 12 fewer) | ⨁⨁⨁◯ Moderate |  |
| **Red meat replaced by legumes** | | | | | | | | | | | | |
| 10 | observational studies | serious^a^ | serious^b^ | not serious | serious^c^ | none | 295‘948 | 62‘068 | **RR 0.97** (0.92 to 1.03) | **6 fewer per 1.000** (from 17 fewer to 6 more) | ⨁◯◯◯ Very low |  |
| **Red meat replaced by whole grains** | | | | | | | | | | | | |
| 3 | observational studies | serious^a^ | not serious | not serious | not serious | none | 174‘399 | 31‘974 | **RR 0.96** (0.94 to 0.97) | **7 fewer per 1.000** (from 11 fewer to 6 fewer) | ⨁⨁⨁◯ Moderate |  |
| **Red meat replaced by cereals** | | | | | | | | | | | | |
| 2 | observational studies | very serious^d^ | serious^b^ | not serious | not serious | none | 199‘182 | 3851 | **RR 0.93** (0.79 to 1.09) | **1 fewer per 1.000** (from 4 fewer to 2 more) | ⨁◯◯◯ Very low |  |
| **Red meat replaced by vegetables** | | | | | | | | | | | | |
| 2 | observational studies | very serious^d^ | not serious | not serious | serious^c^ | none | 74‘854 | 5309 | **RR 0.90** (0.75 to 1.07) | **7 fewer per 1.000** (from 18 fewer to 5 more) | ⨁◯◯◯ Very low |  |
| **Processed meat replaced by nuts** | | | | | | | | | | | | |
| 9 | observational studies | serious^a^ | not serious | not serious | not serious | none | 163‘049 | 41‘624 | **RR 0.79** (0.71 to 0.89) | **54 fewer per 1.000** (from 74 fewer to 28 fewer) | ⨁⨁⨁◯ Moderate |  |
| **Processed meat replaced by legumes** | | | | | | | | | | | | |
| 9 | observational studies | serious^a^ | not serious | not serious | not serious | none | 163‘049 | 41‘624 | **RR 0.91** (0.85 to 0.98) | **23 fewer per 1.000** (from 38 fewer to 5 fewer) | ⨁⨁⨁◯ Moderate |  |
| **Unprocessed red meat replaced by nuts** | | | | | | | | | | | | |
| 8 | observational studies | serious^a^ | not serious | not serious | not serious | none | 151‘024 | 32‘801 | **RR 0.93** (0.92 to 0.94) | **15 fewer per 1.000** (from 17 fewer to 13 fewer) | ⨁⨁⨁◯ Moderate |  |
| **Unprocessed red meat replaced by legumes** | | | | | | | | | | | | |
| 8 | observational studies | serious^a^ | not serious | not serious | serious^c^ | none | 151‘024 | 32‘801 | **RR 0.98** (0.95 to 1.01) | **4 fewer per 1.000** (from 11 fewer to 2 more) | ⨁⨁◯◯ Low |  |
| **Poultry replaced by nuts** | | | | | | | | | | | | |
| 7 | observational studies | serious^a^ | not serious | not serious | serious^c^ | none | 132‘203 | 34‘851 | **RR 0.98** (0.94 to 1.01) | **5 fewer per 1.000** (from 16 fewer to 3 more) | ⨁⨁◯◯ Low |  |
| **Fish replaced by nuts** | | | | | | | | | | | | |
| 7 | observational studies | serious^a^ | not serious | not serious | serious^c^ | none | 132‘203 | 34‘851 | **RR 0.99** (0.95 to 1.03) | **3 fewer per 1.000** (from 13 fewer to 8 more) | ⨁⨁◯◯ Low |  |
| **Dairy replaced by nuts / legumes** | | | | | | | | | | | | |
| 4 | observational studies | serious^a^ | not serious | not serious | not serious | none | 320‘276 | 77‘414 | **RR 0.96** (0.93 to 0.98) | **15 fewer per 1.000** (from 20 fewer to 7 fewer) | ⨁⨁⨁◯ Moderate |  |
| **Dairy replaced by nuts** | | | | | | | | | | | | |
| 3 | observational studies | serious^a^ | not serious | not serious | not serious | none | 225‘147 | 59‘204 | **RR 0.94** (0.91 to 0.97) | **16 fewer per 1.000** (from 24 fewer to 8 fewer) | ⨁⨁⨁◯ Moderate |  |
| **Eggs replaced by nuts** | | | | | | | | | | | | |
| 8 | observational studies | serious^a^ | not serious | not serious | not serious | none | 653‘323 | 164‘179 | **RR 0.85** (0.81 to 0.88) | **38 fewer per 1.000** (from 48 fewer to 30 fewer) | ⨁⨁⨁◯ Moderate |  |
| **Eggs replaced by legumes** | | | | | | | | | | | | |
| 7 | observational studies | serious^a^ | not serious | not serious | not serious | none | 550‘802 | 138‘203 | **RR 0.90** (0.89 to 0.91) | **25 fewer per 1.000** (from 28 fewer to 23 fewer) | ⨁⨁⨁◯ Moderate |  |
| **Butter replaced by olive oil** | | | | | | | | | | | | |
| 3 | observational studies | serious^a^ | not serious | not serious | not serious | none | 613‘503 | 166‘184 | **RR 0.94** (0.92 to 0.97) | **16 fewer per 1.000** (from 22 fewer to 8 fewer) | ⨁⨁⨁◯ Moderate |  |

*****Absolute risks were calculated in GRADEpro (<https://www.gradepro.org/>) as the difference between the baseline risk of an outcome (e.g., in persons receiving control intervention or estimated in the observational studies) and the risk of outcome after the intervention is applied, i.e. the risk of an outcome in persons who were exposed or received an intervention. The calculation of the absolute effect was based on the relative magnitude of an effect and baseline risk. Absolute effect calculation option is selected by default and GRADEpro calculates the absolute effect risk once at least one of the baseline risk values is provided and the magnitude of the relative effect (RR, OR or HR) has been entered.

**CI:** confidence interval; **RR:** risk ratio

#### Explanations

a. Downgraded by one level for risk of bias, since all studies were rated with a moderate risk of bias.

b. Downgraded by one level for inconsistency since point estimates and 95% CI did not fully overlap between studies and I^2^ was substantial.

c. Downgraded by one level for imprecision since 95% CI crosses threshold of a minimal important difference (absolute risk: 5 fewer and/or 5 more events per 1.000).

d. Downgraded by two levels for risk of bias, since one included study was rated with a high risk of bias.
