# Supplementary material for: Substitution of animal-based with plant-based foods on cardiometabolic health and all-cause mortality: a systematic review and meta-analysis of prospective studies
Source: BMC Med. 2023 Nov 16;21:404. doi: 10.1186/s12916-023-03093-1 (PMC10652524; doi:10.1186/s12916-023-03093-1)
Supplement: Supplementary file 3 — Additional file 3: Fig. S1. Risk of bias of each study for each domain and overall. Fig. S2. Risk of bias of judgements within each bias domain. Fig. S3. Forest plots for the substitution analyses regarding total CVD. Fig. S4. Forest plots for the substitution analyses regarding CVD mortality. Fig. S5. Forest plots for the substitution analyses regarding CHD incidence. Fig. S6. Forest plot for the substitution analyses regarding total diabetes. Fig. S7. Forest plots for the substitution analyses regarding incidence of type 2 diabetes. Fig. S8. Forest plots for the substitution analyses regarding all-cause mortality. Fig. S9. Forest plot showing the results from extracted pooled analyses regarding CVD mortality and CVD incidence. Fig. S10. Forest plot showing the results from extracted pooled analyses regarding CHD and stroke incidence. Fig. S11. Forest plot showing the results from extracted pooled analyses regarding diabetes. Fig. S12. Forest plot showing the results from extracted pooled analyses regarding all-cause mortality. Fig. S13. Forest plot showing the results from single cohorts regarding CVD mortality and CVD incidence. Fig. S14. Forest plot showing the results from single cohorts regarding CHD, MI and stroke incidence. Fig. S15. Forest plot showing the results from single cohorts regarding diabetes. Fig. S16. Forest plot showing the results from single cohorts regarding all-cause mortality. [file 12916_2023_3093_MOESM3_ESM.docx]

**Fig. S1:** Risk of bias of each study for each domain and overall


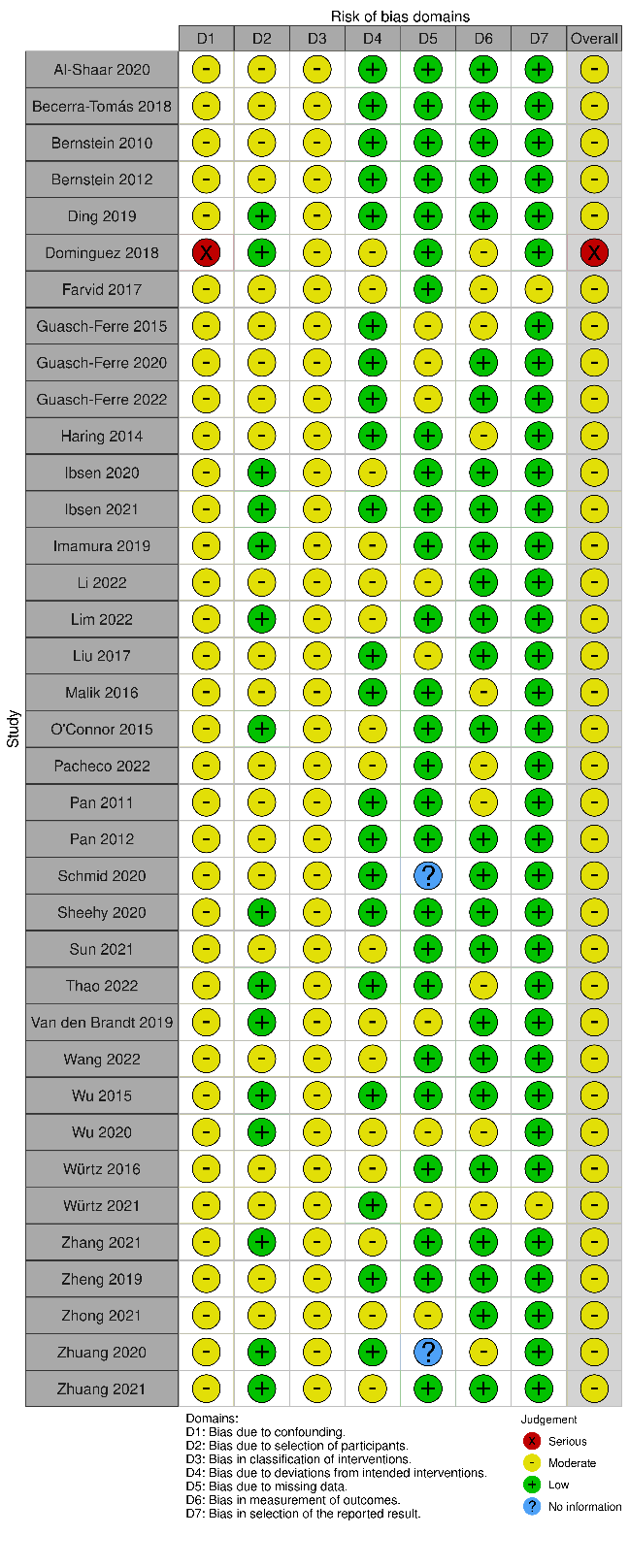

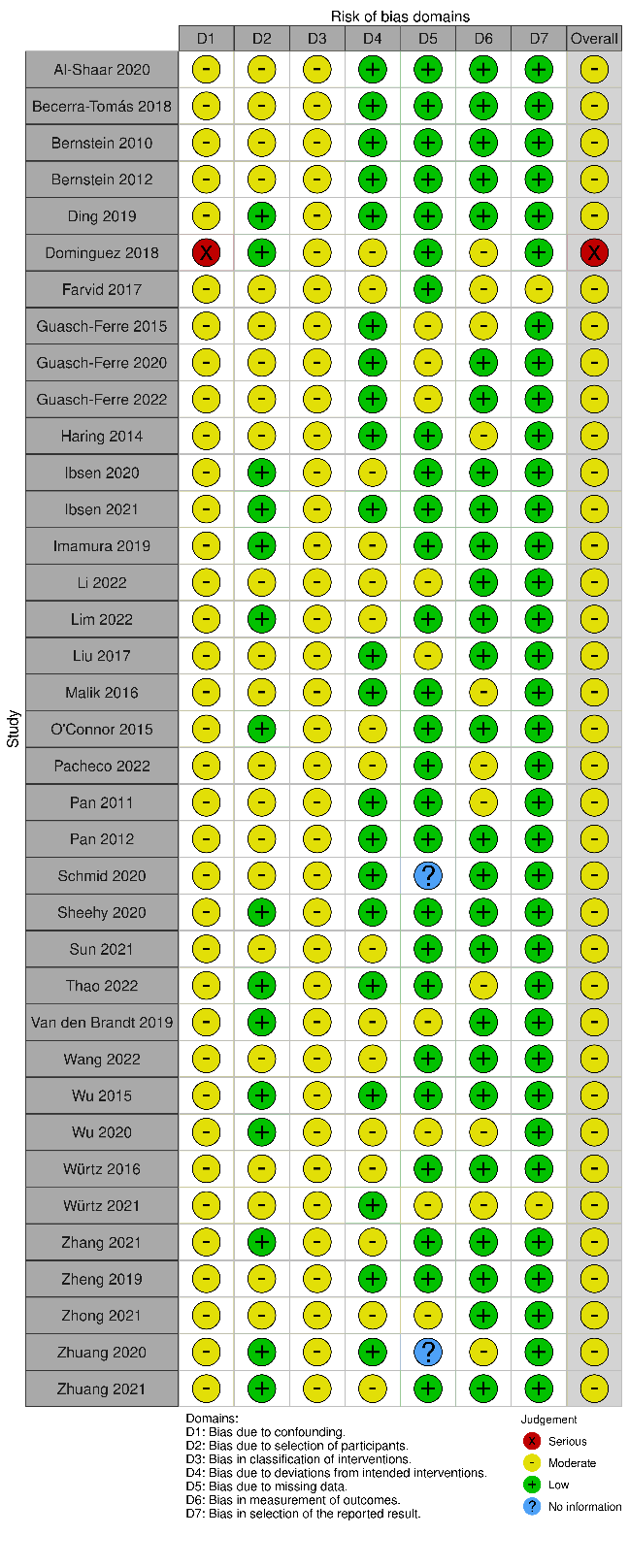


**Fig. S2:** Risk of bias of judgements within each bias domain


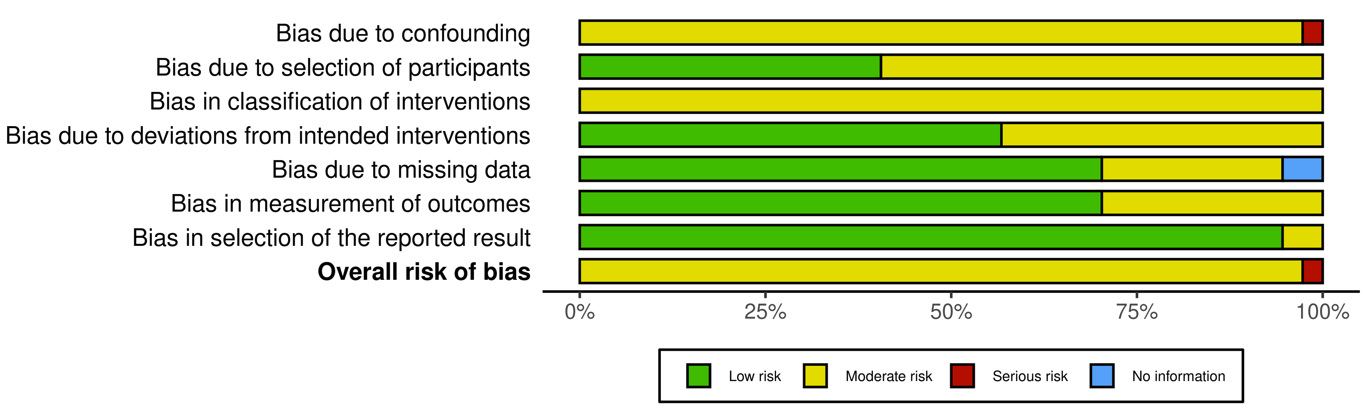


Bias due to confounding

Bias due to selection of participants

Bias due to exposure assessment

Bias due to misclassification during follow-up

Bias due to missing data

Bias in measurement of outcomes

Bias in selection of the reported result

**Overall risk of bias**

**Fig. S3:** Forest plots regarding total CVD for the substitution of A) red meat with nuts, B) red meat with legumes, C) red meat with whole grains, D) processed meat with nuts, E) processed meat with legumes, F) processed meat with whole grains, G) unprocessed red meat with nuts, H) unprocessed red meat with legumes, I) unprocessed red meat with whole grains, J) poultry with nuts, K) poultry with legumes, L) fish with nuts, M) fish with legumes, N) dairy with nuts / legumes, O) dairy with nuts, P) eggs with nuts, Q) eggs with legumes, R) butter with margarine and S) butter with olive oil

| A) | 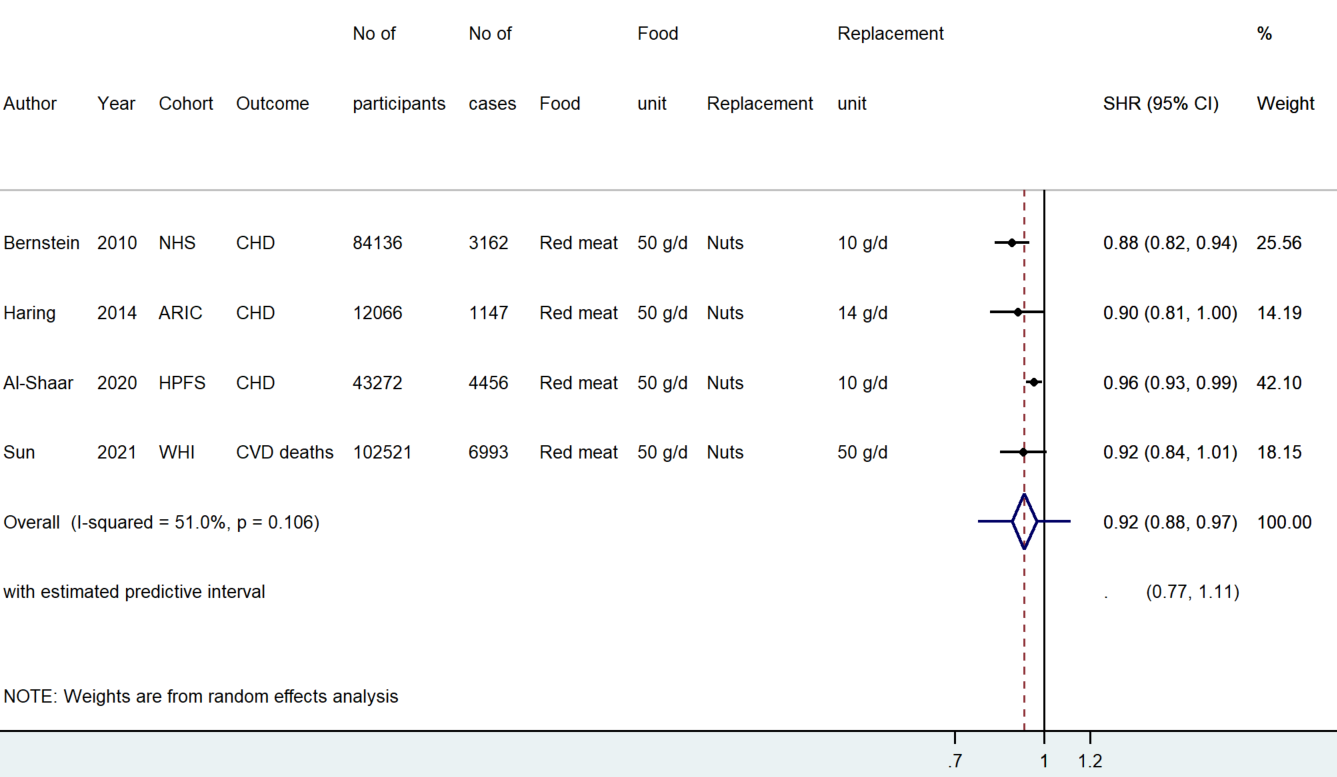  tau^2^ = 0.0012 |
| --- | --- |
| B) | 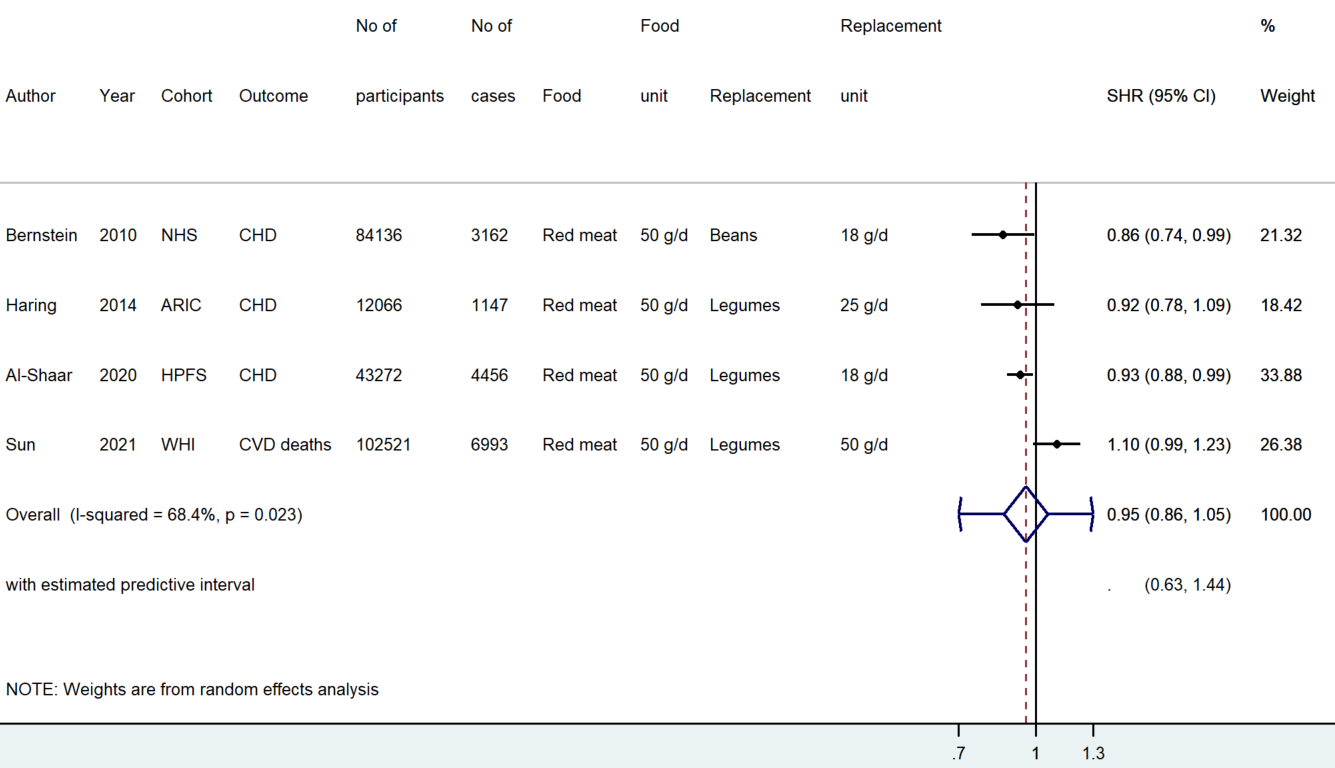  tau^2^ = 0.0067 |
| C) | 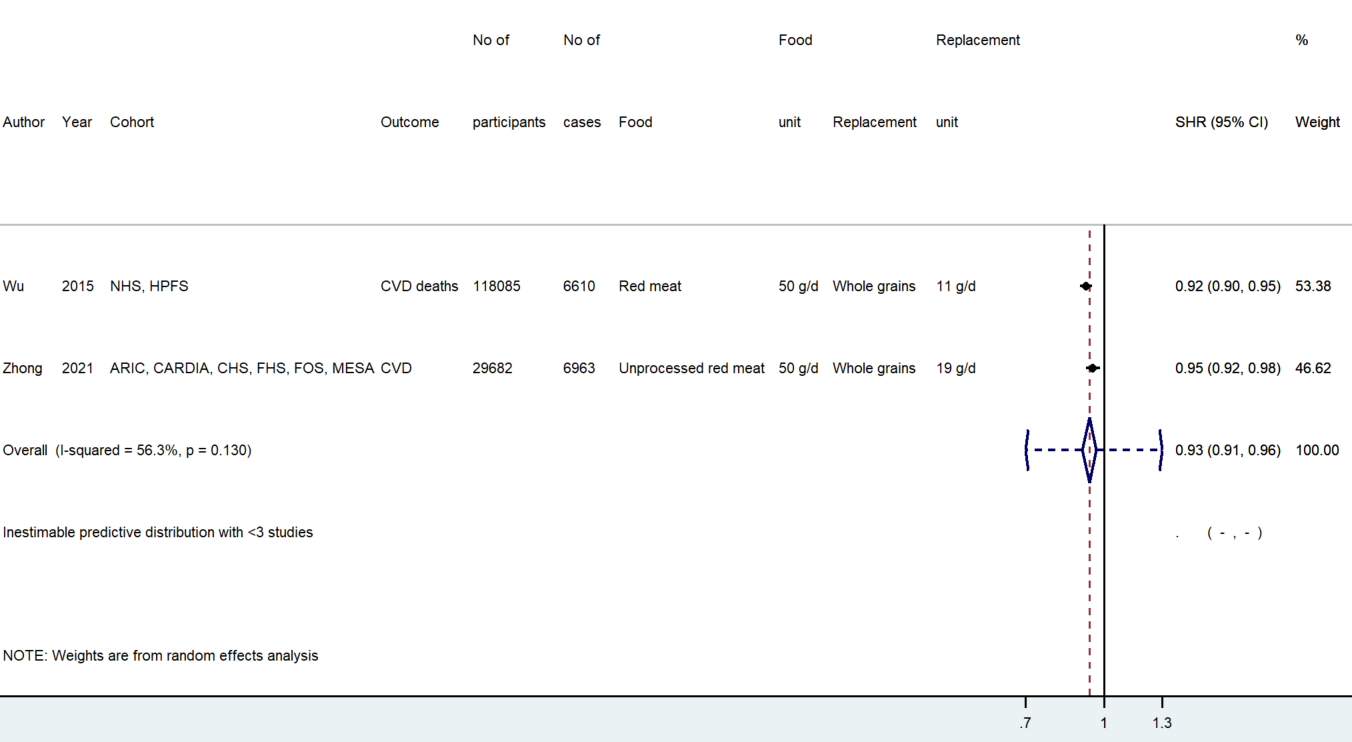  tau2 = 0.003 |
| D) | 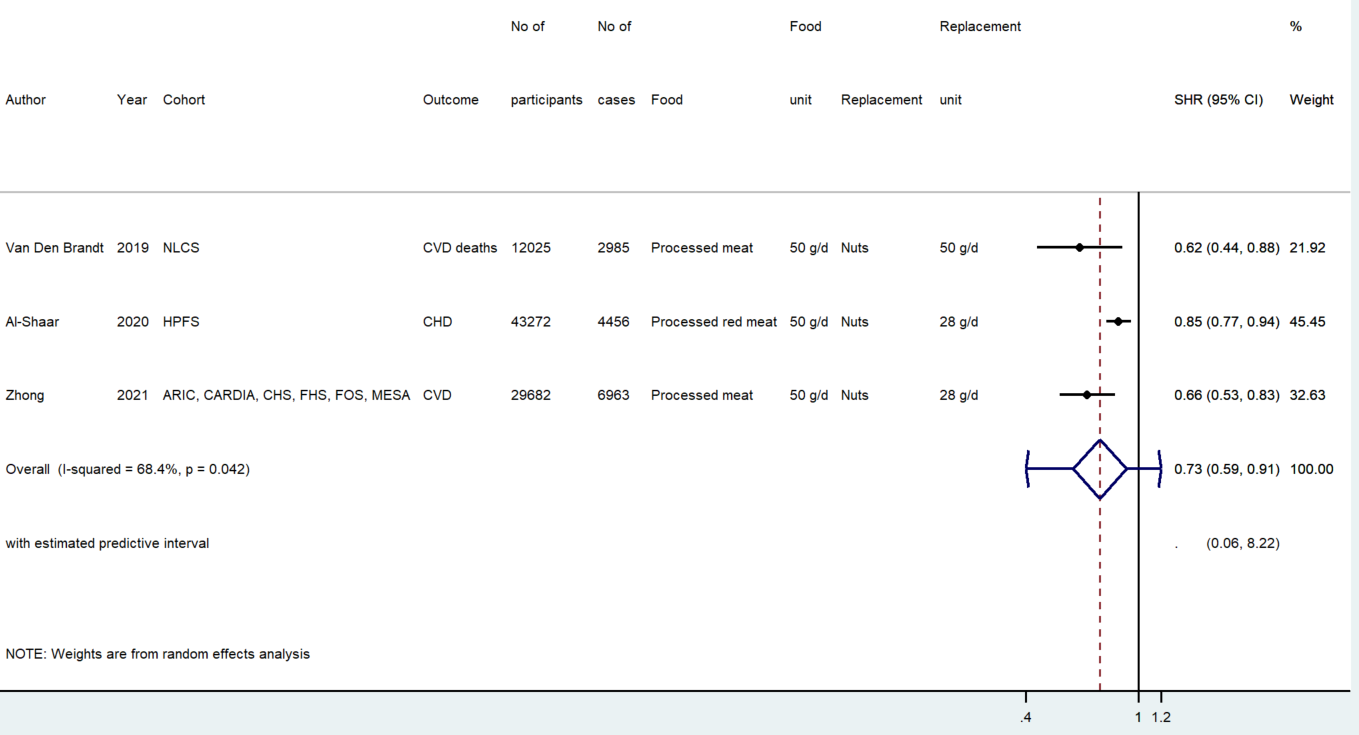  tau2 = 0.0241 |
| E) | 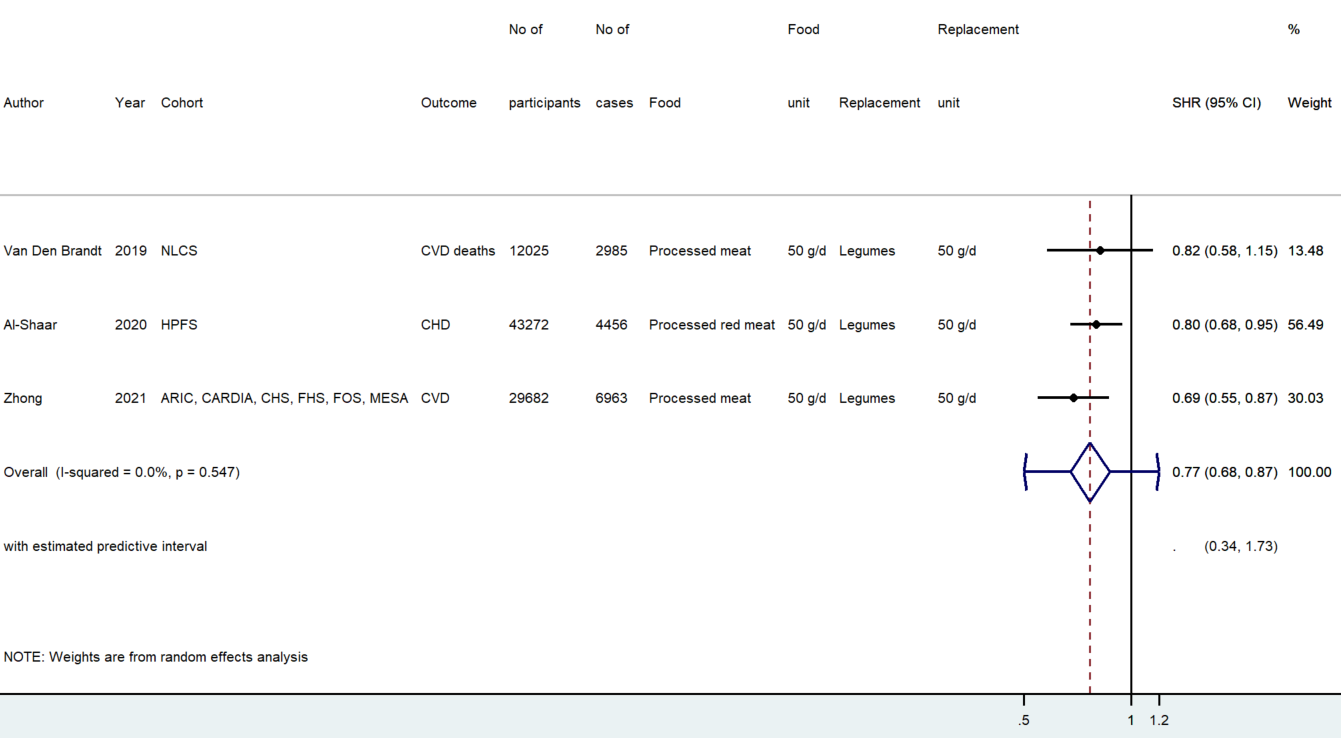  tau^2^ = 0.0000 |
| F) | 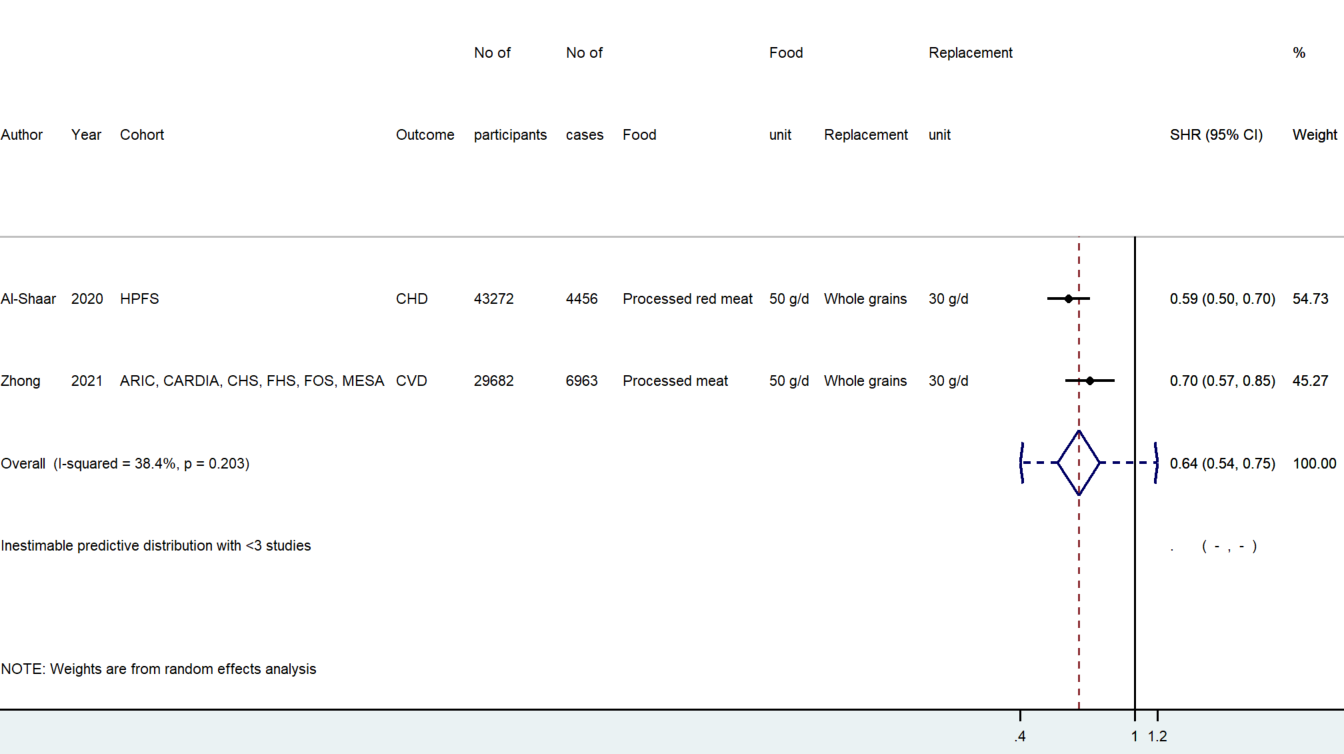  tau^2^ = 0.0056 |
| G) | 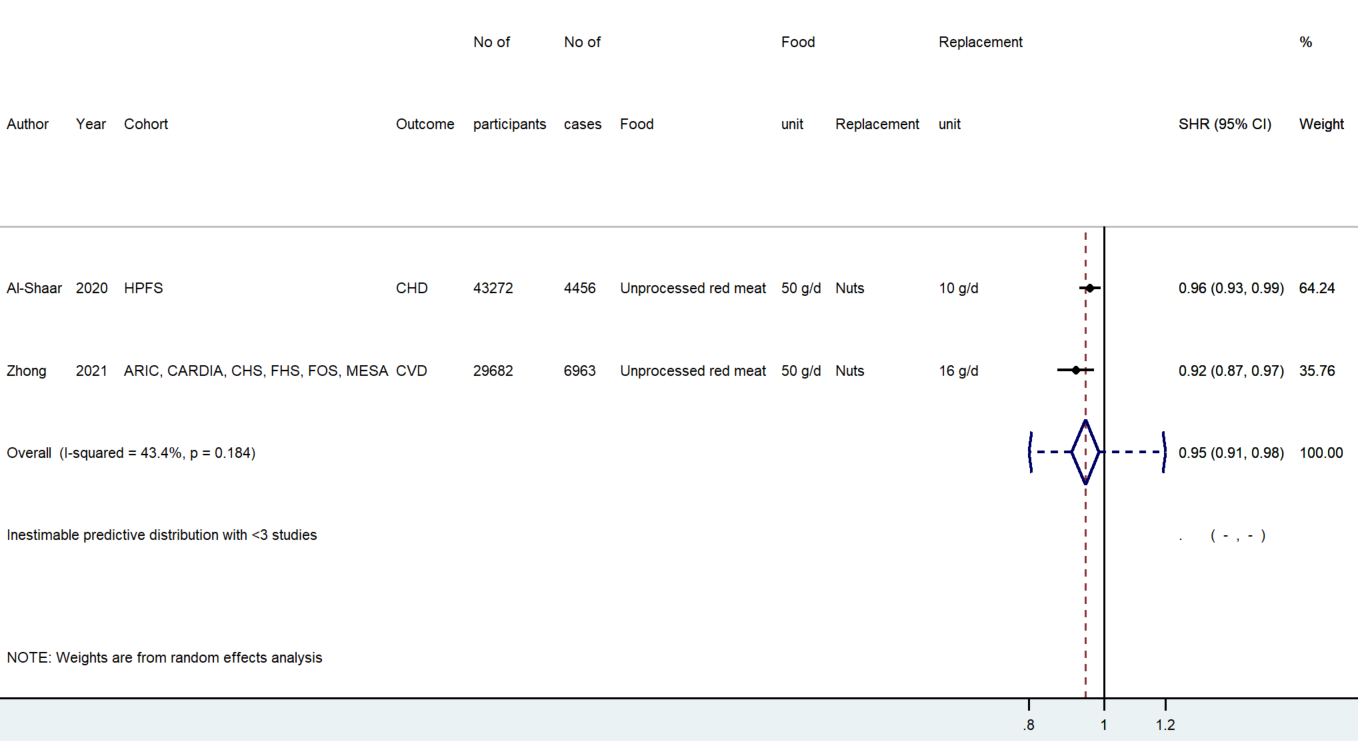  tau^2^ = 0.0004 |
| H) | 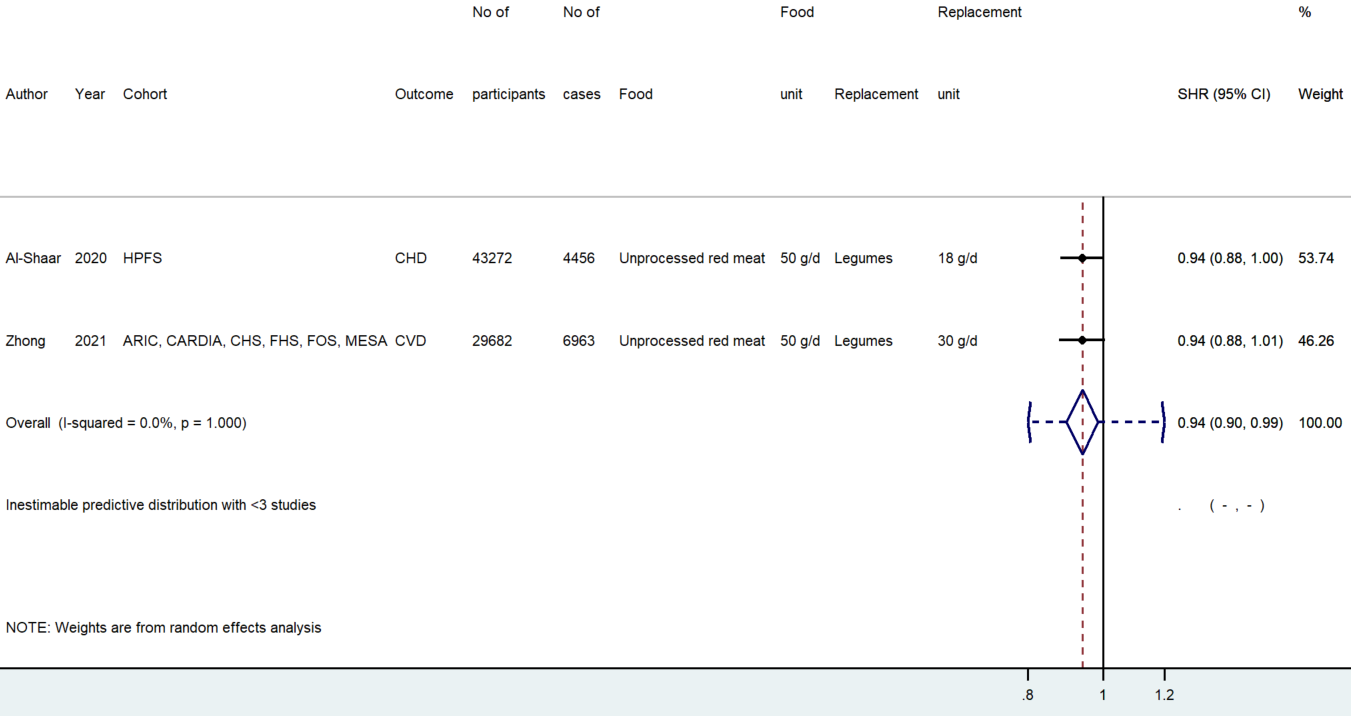  tau^2^ = 0.0000 |
| I) | 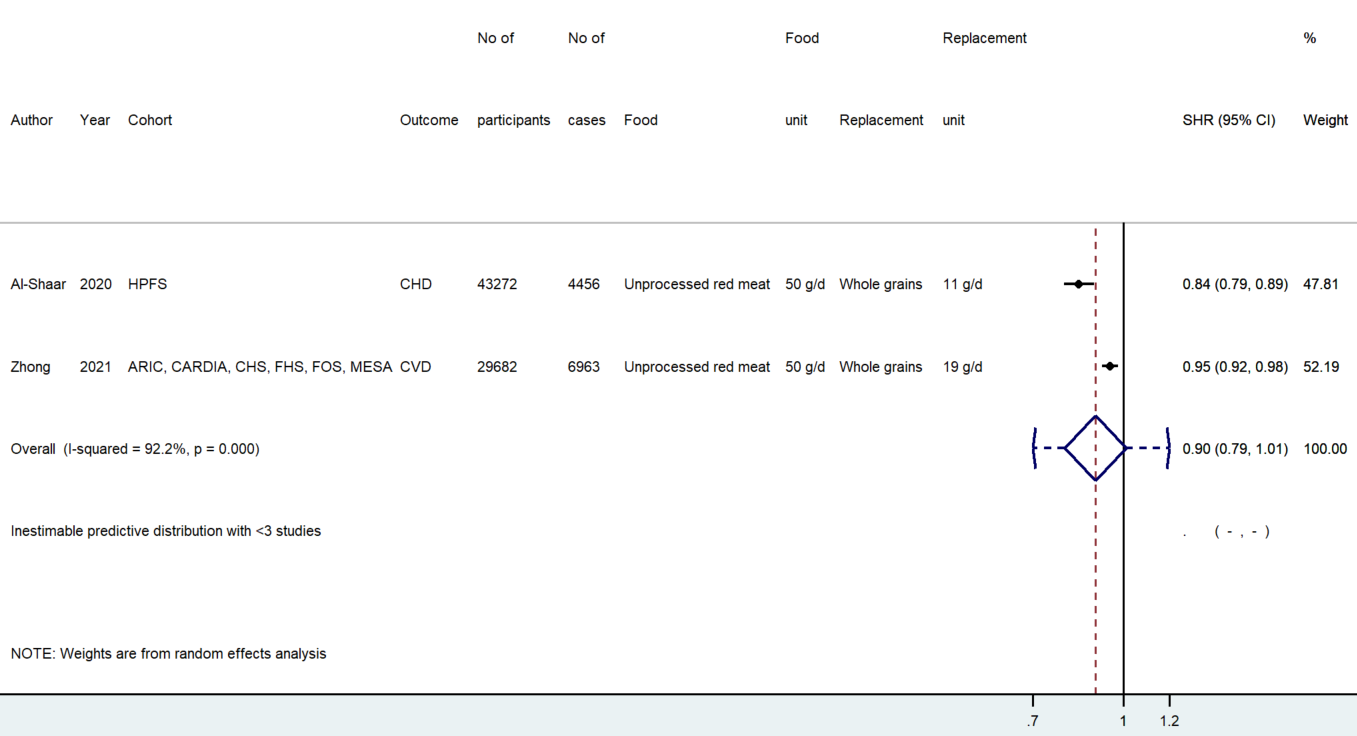  tau^2^ = 0.0070 |
| J) | 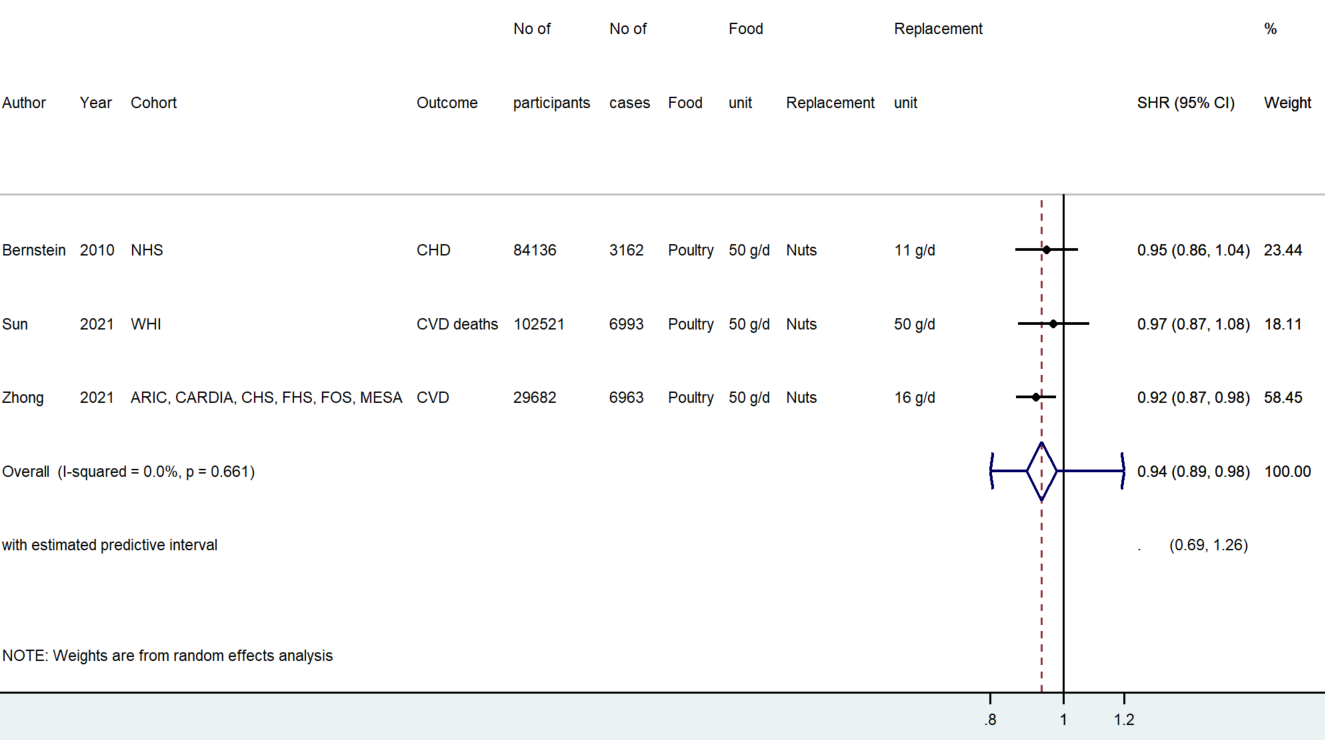  tau^2^ = 0.0000 |
| K) | 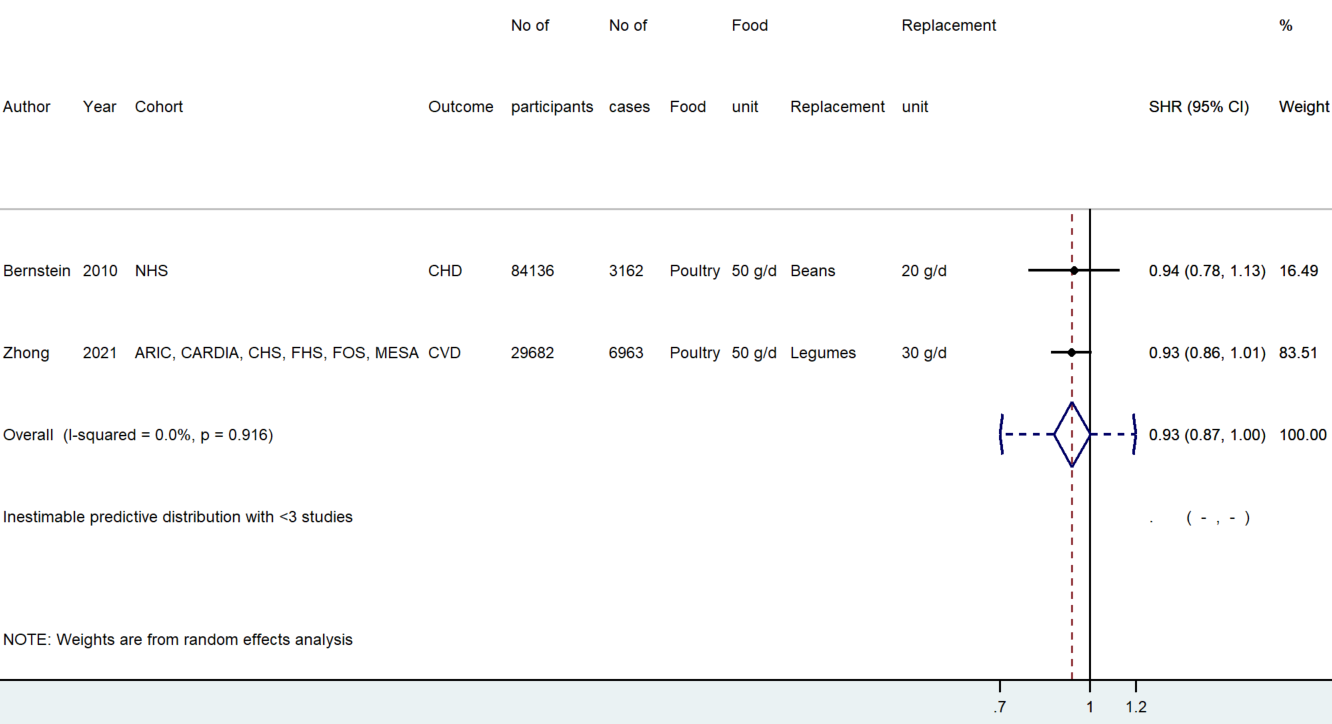  tau^2^ = 0.0000 |
| L) | 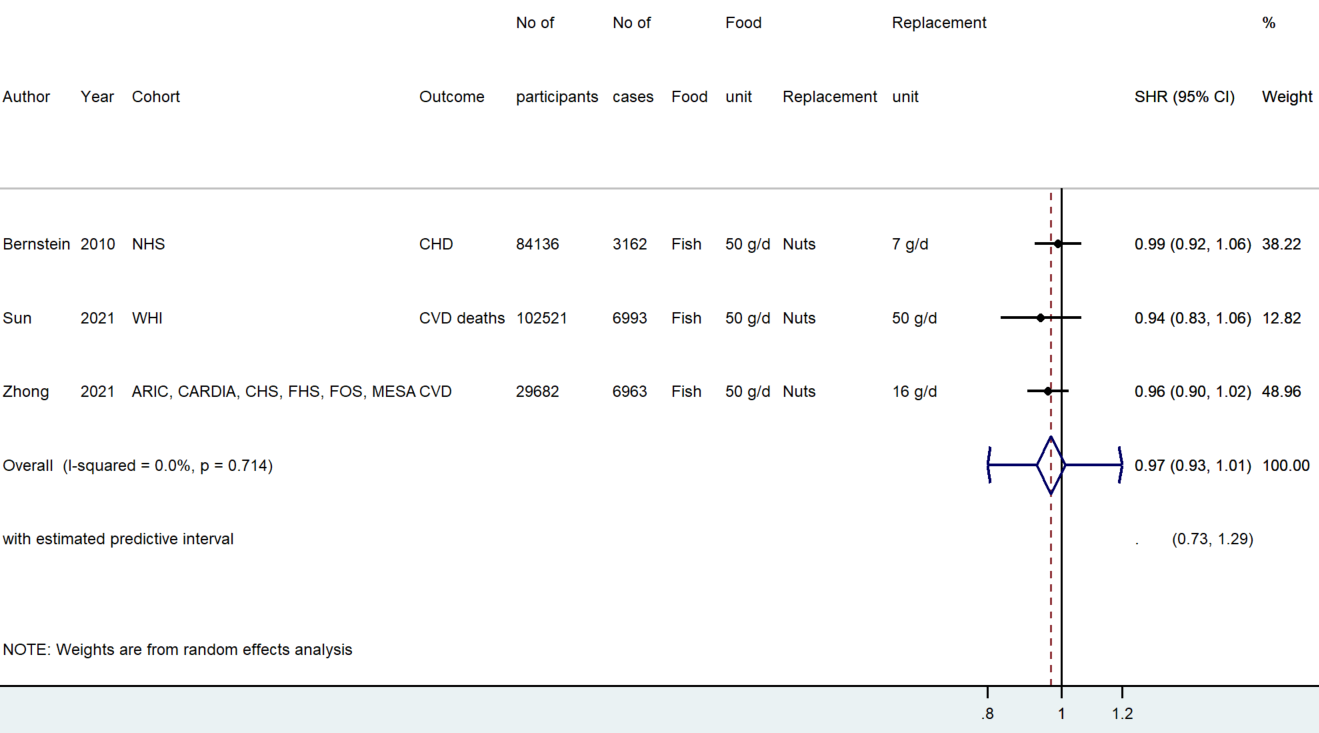  tau^2^ = 0.0000 |
| M) | 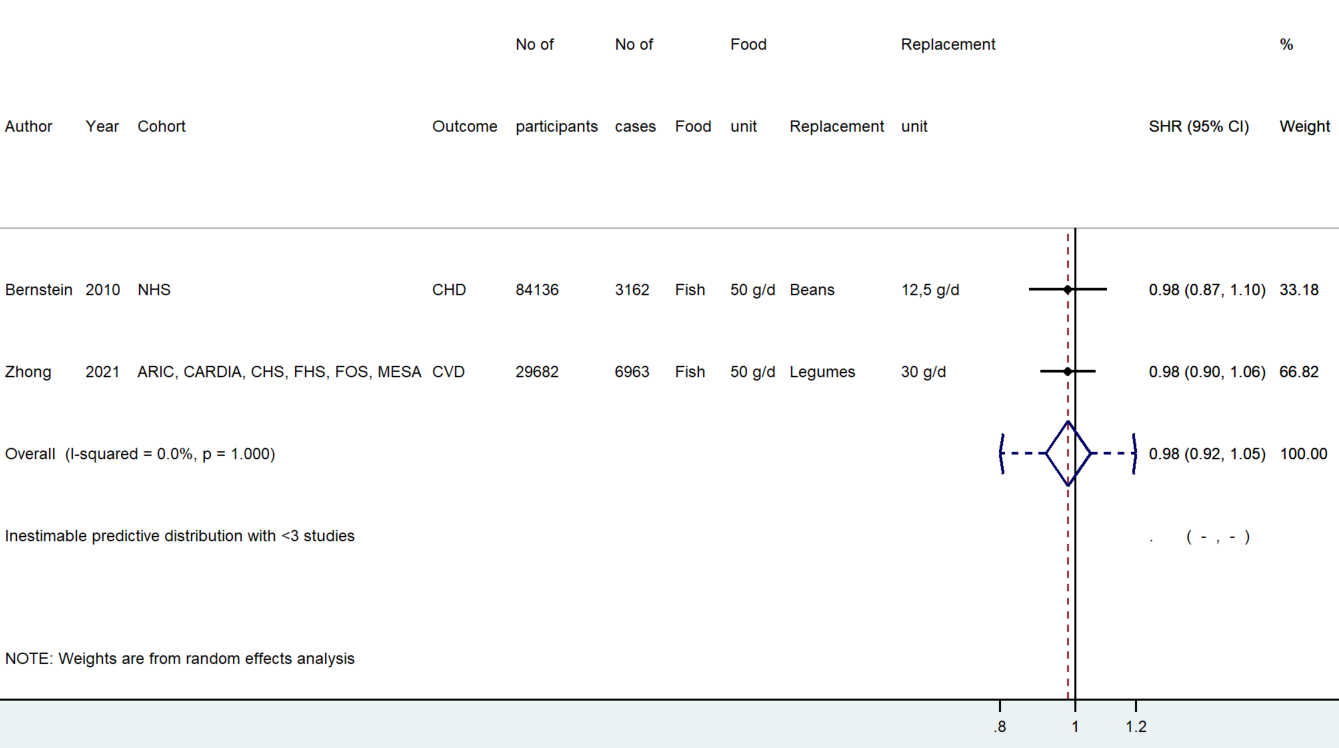  tau^2^ = 0.0000 |
| N) | 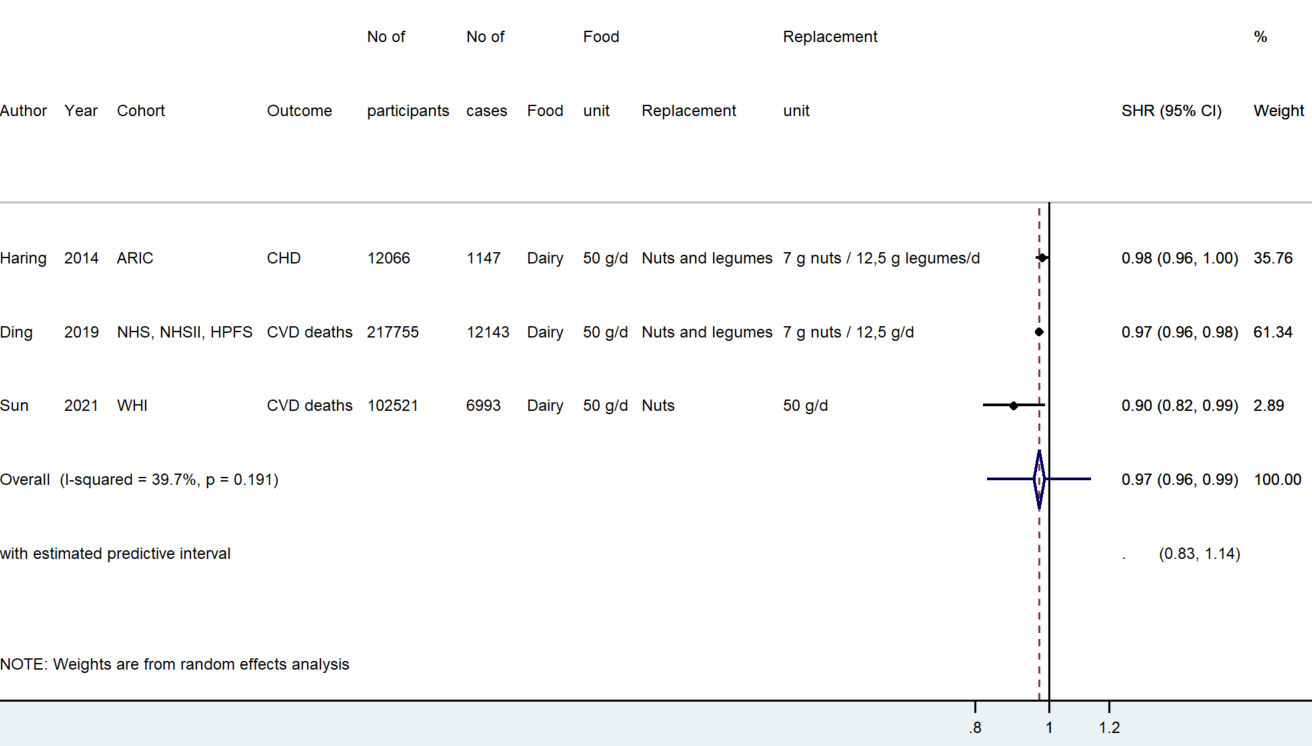  tau^2^ = 0.0001 |
| O) | 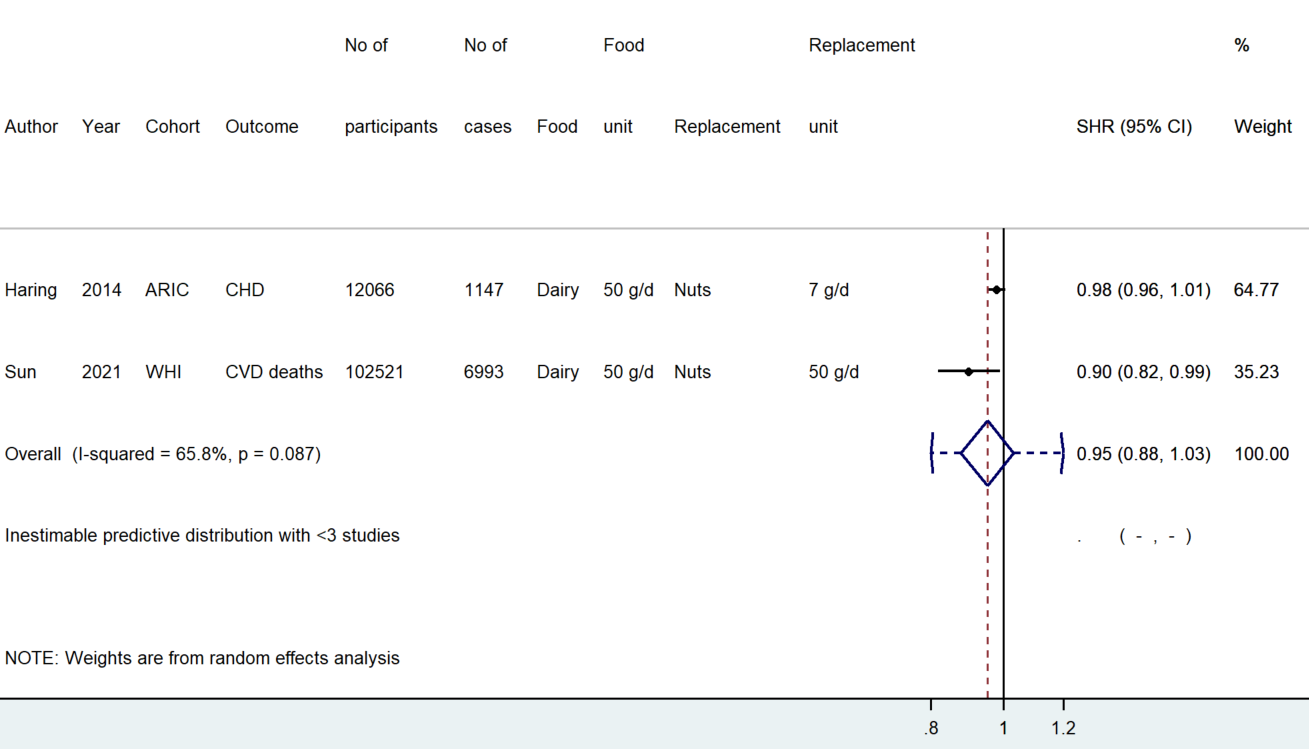  tau^2^ = 0.0000 |
| P) | 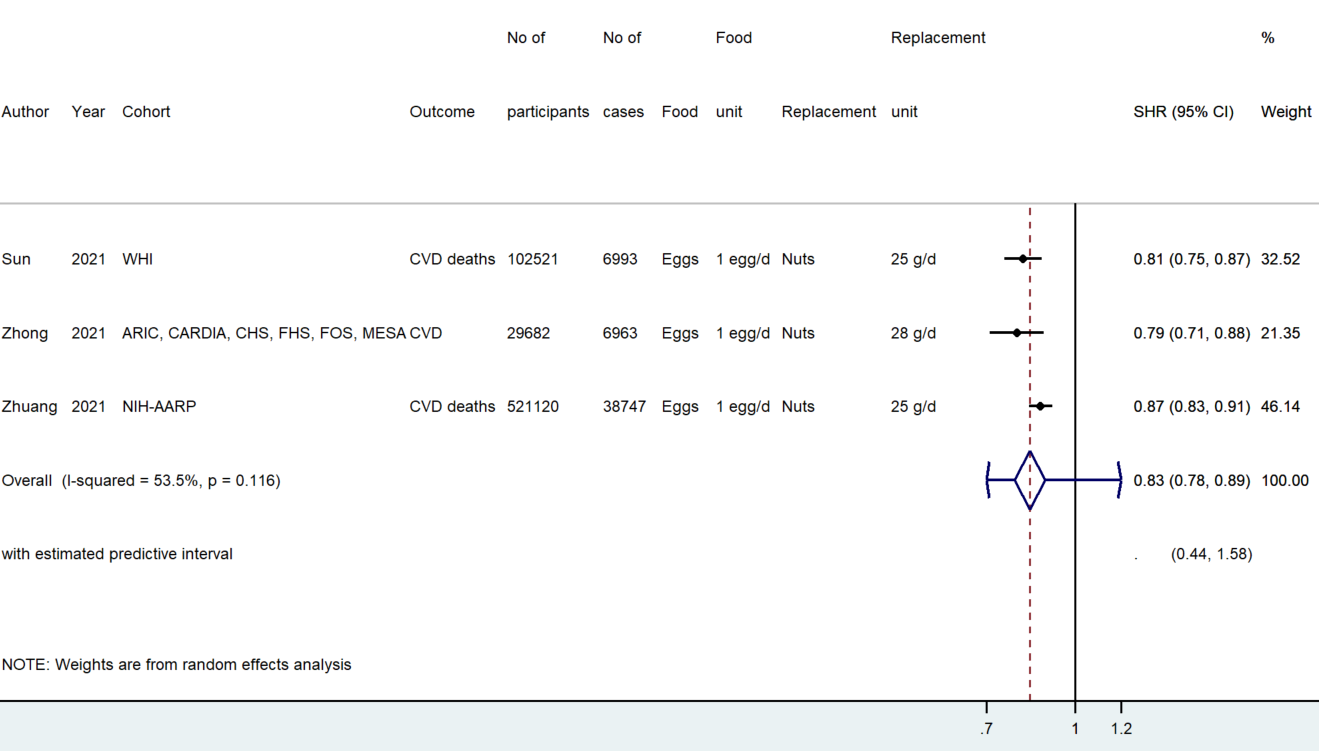  tau^2^ = 0.0016 |
| Q) | 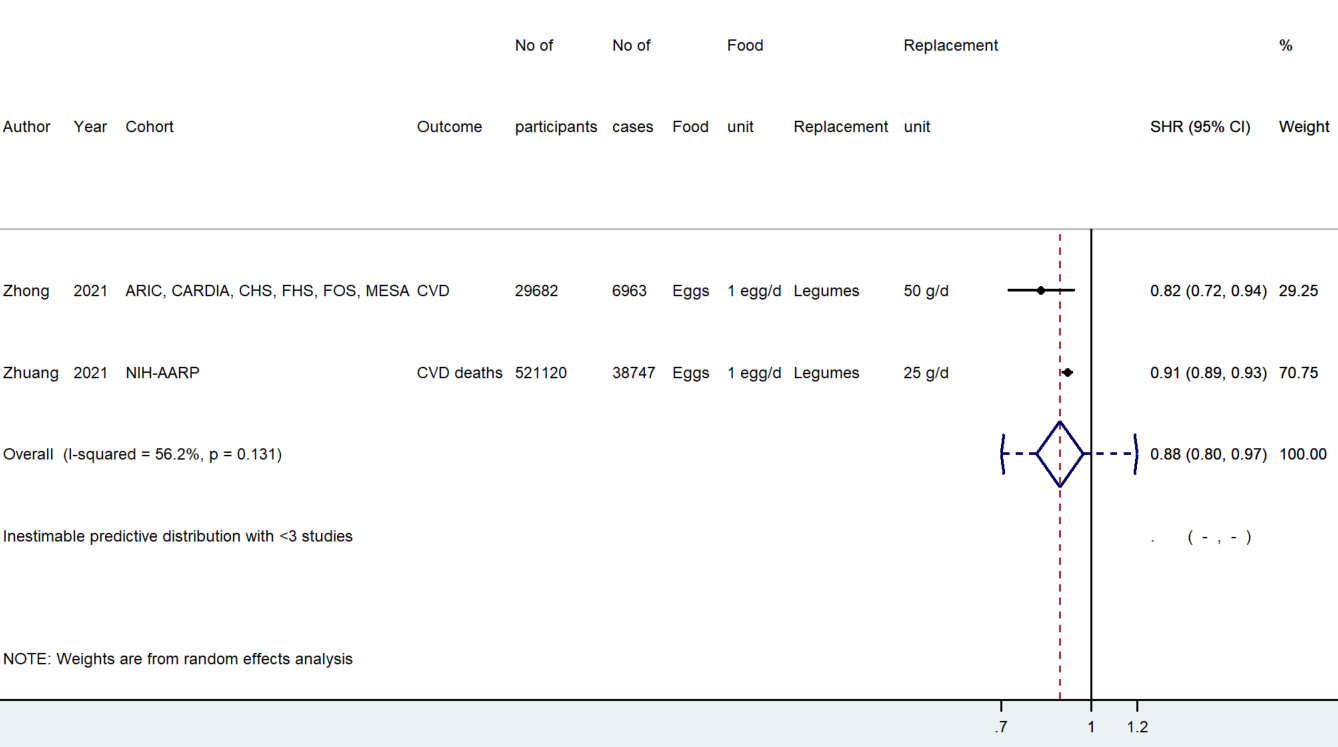  tau^2^ = 0.0030 |
| R) | 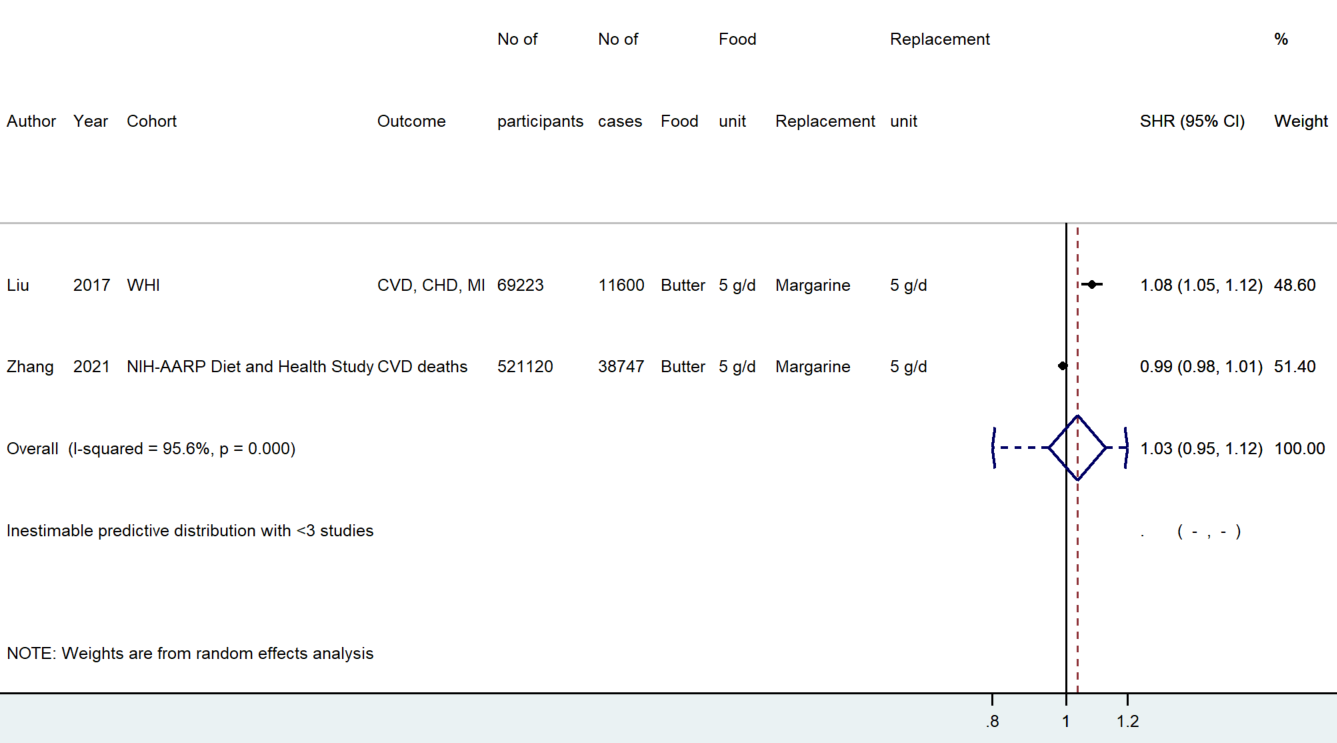  tau^2^ = 0.0036 |
| S) | 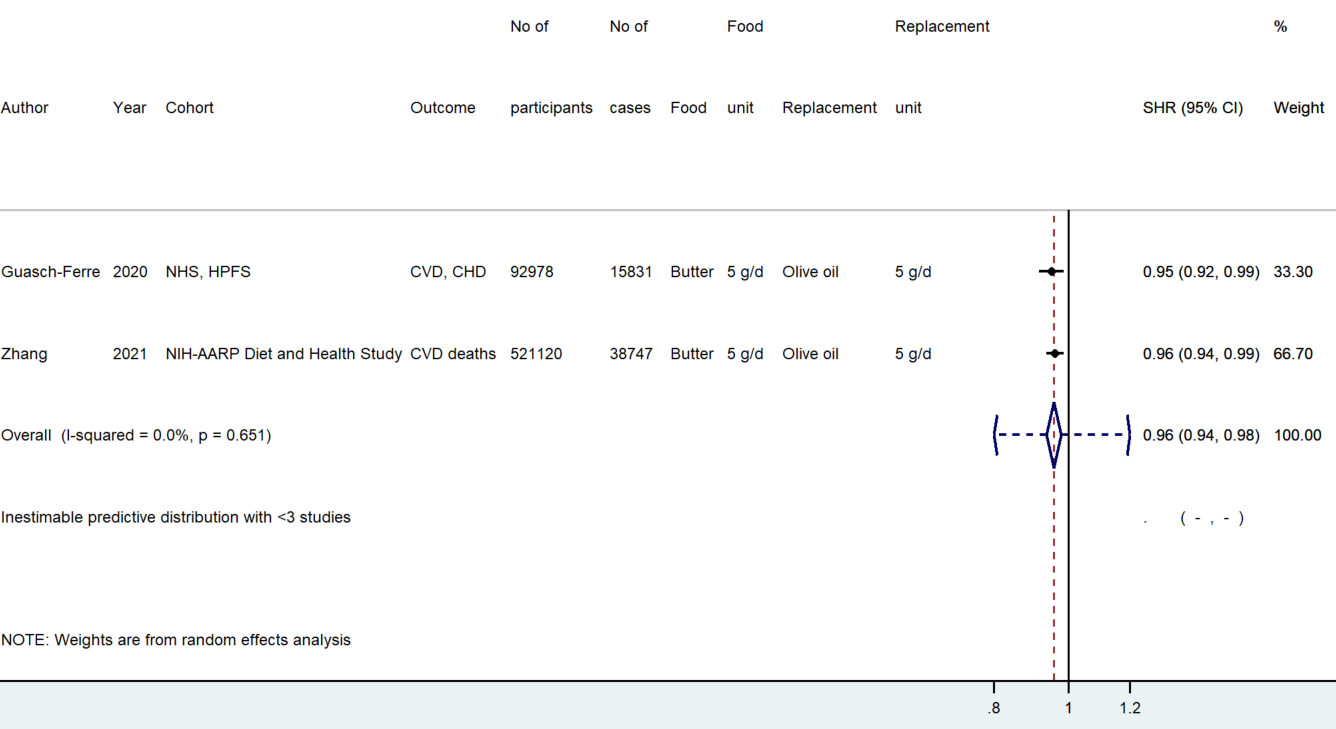  tau^2^ = 0.0000 |

**Fig. S4:** Forest plots regarding CVD mortality for the substitution of A) red and processed meat with nuts, B) red meat with whole grains / cereals, C) eggs with nuts and D) butter with olive oil

| A) | 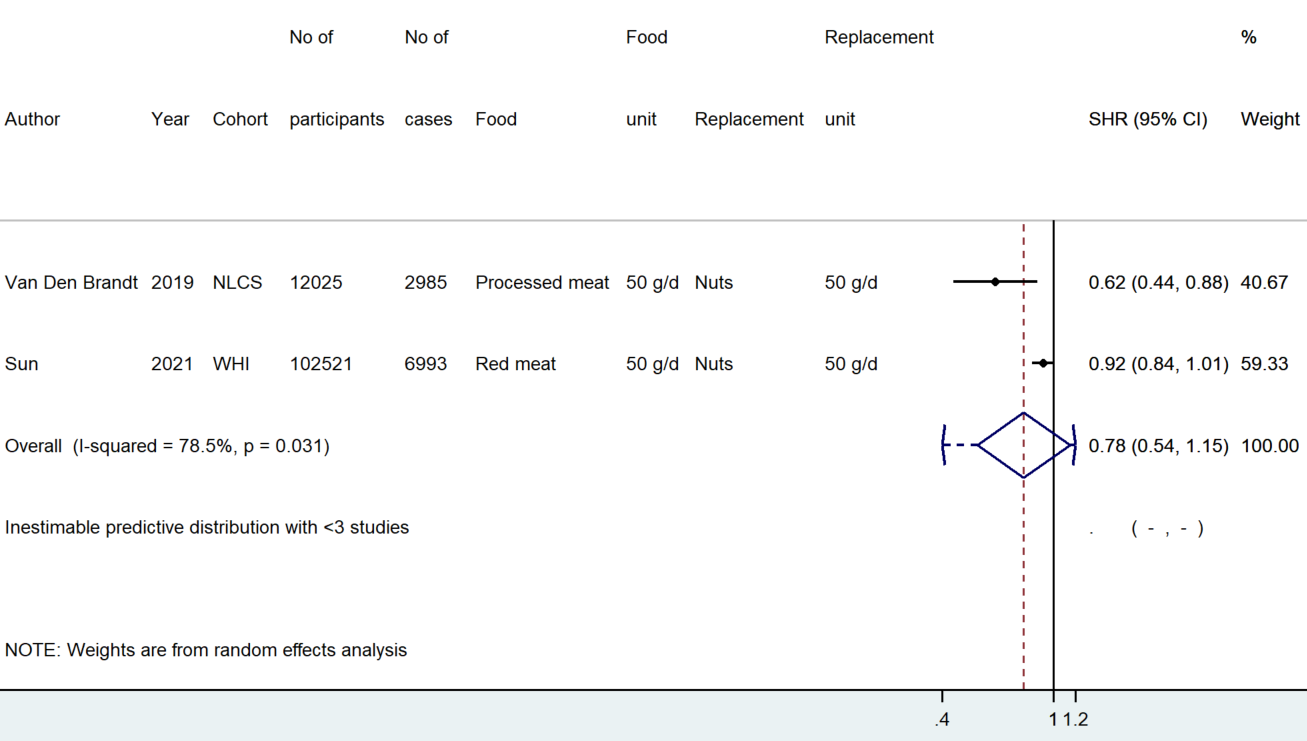  tau^2^ = 0.0611 |
| --- | --- |
| B) | 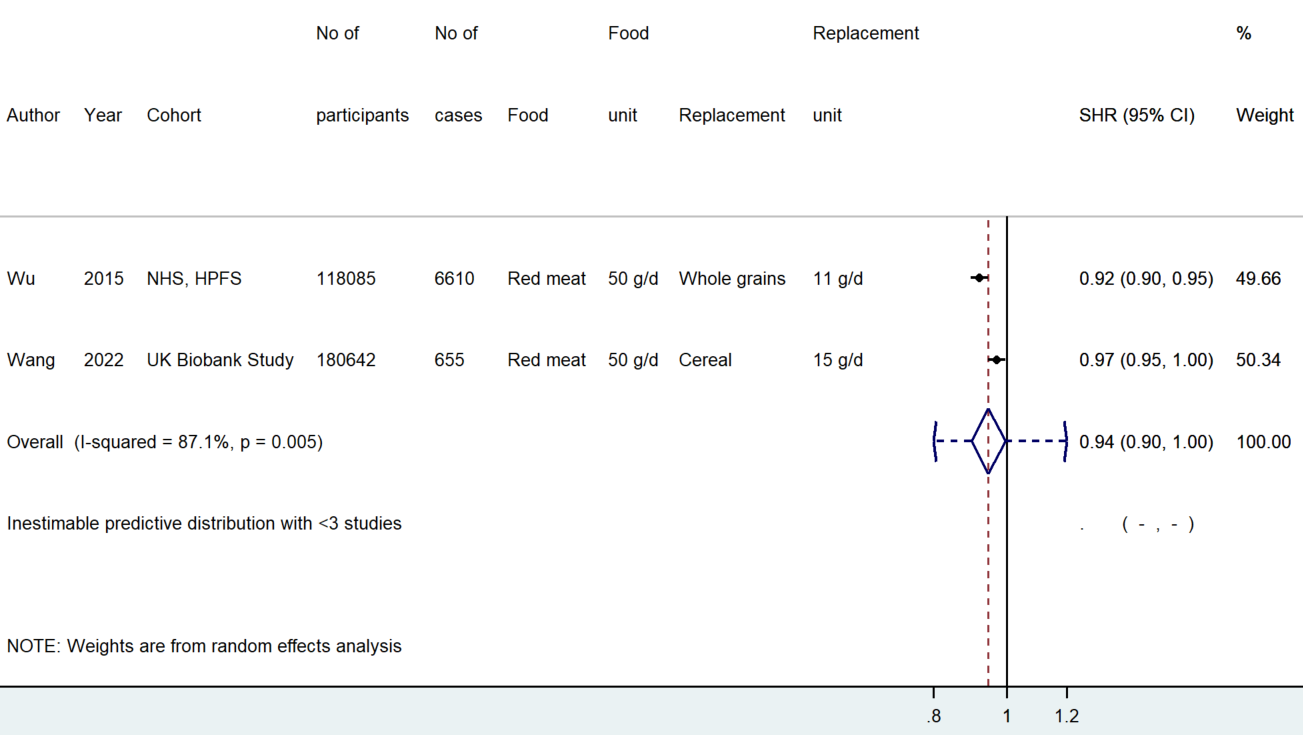  tau^2^ = 0.0012 |
| C) | 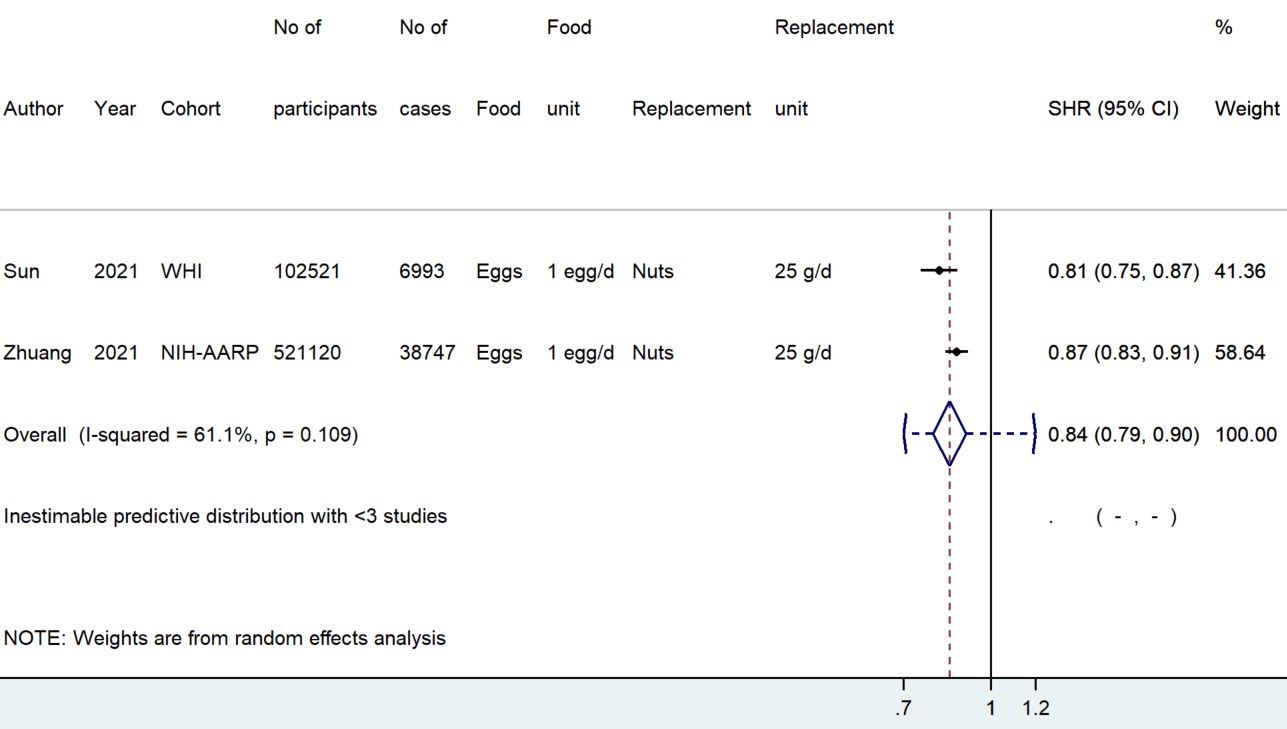  tau^2^ = 0.0016 |
| D) | 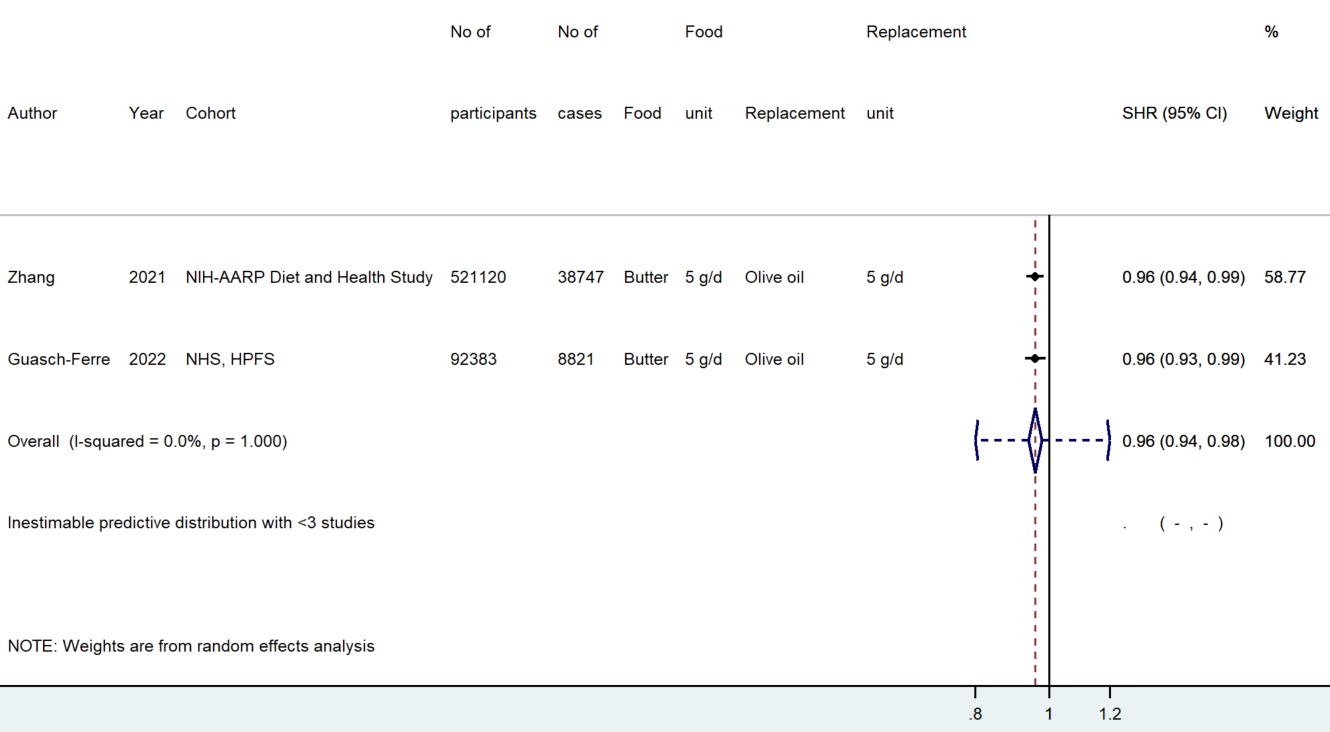  tau^2^ = 0.0000 |

**Fig. S5:** Forest plots regarding CHD incidence for the substitution of A) red meat with nuts, B) red meat with legumes, C) processed meat with nuts, D) processed meat with legumes, E) poultry with nuts, F) poultry with legumes, G) fish / seafood with nuts and H) fish / seafood with legumes

| A) | 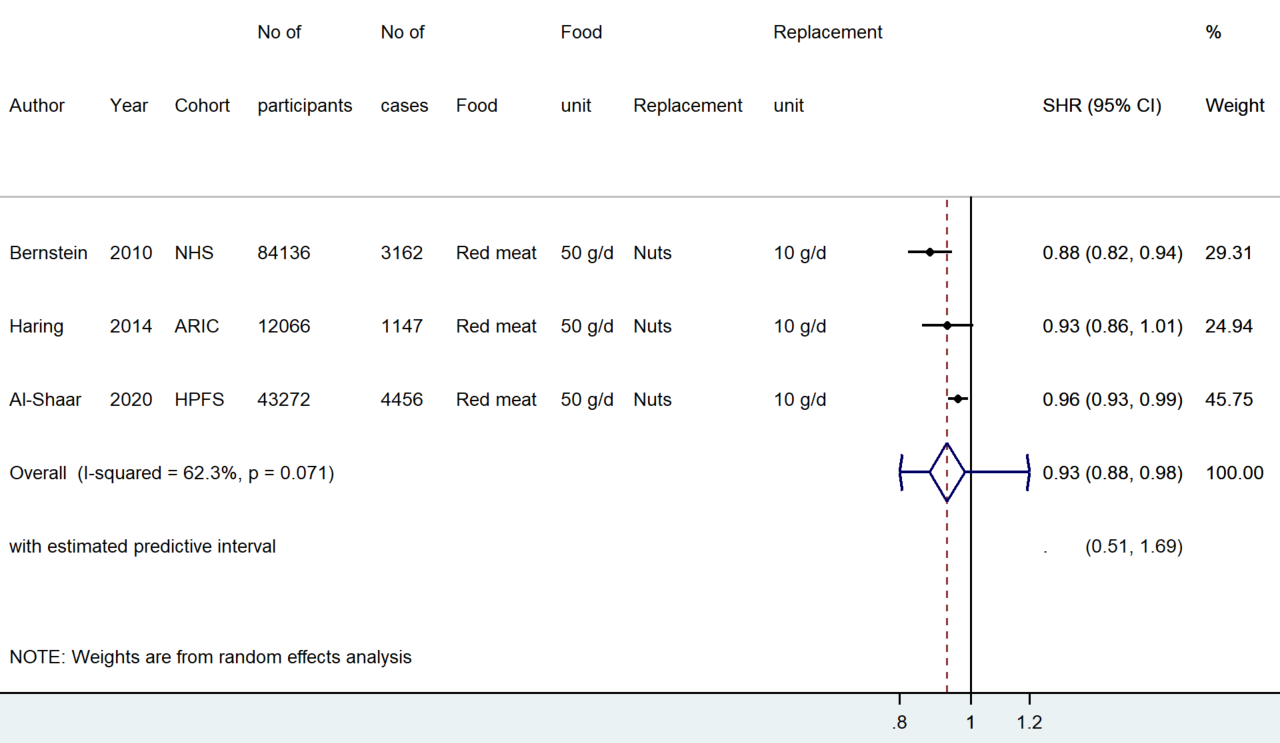  tau^2^ = 0.0015 |
| --- | --- |
| B) | 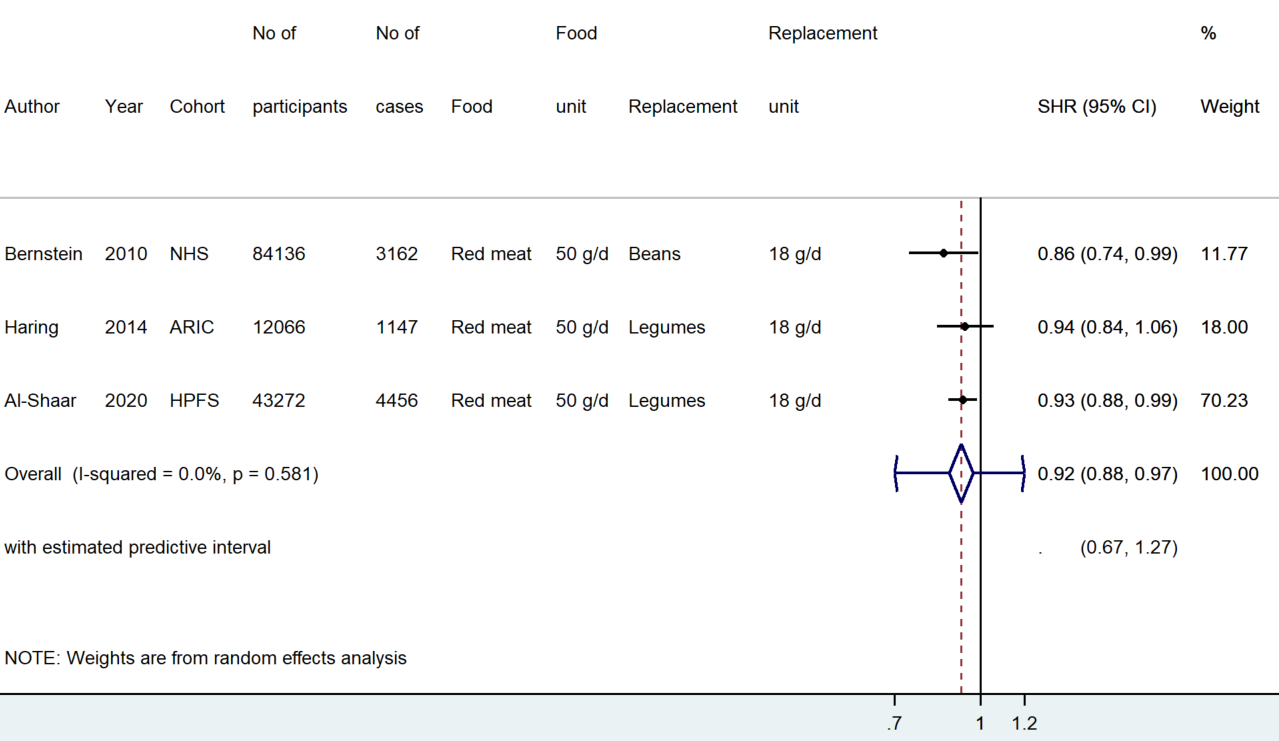  tau^2^ = 0.0000 |
| C) | 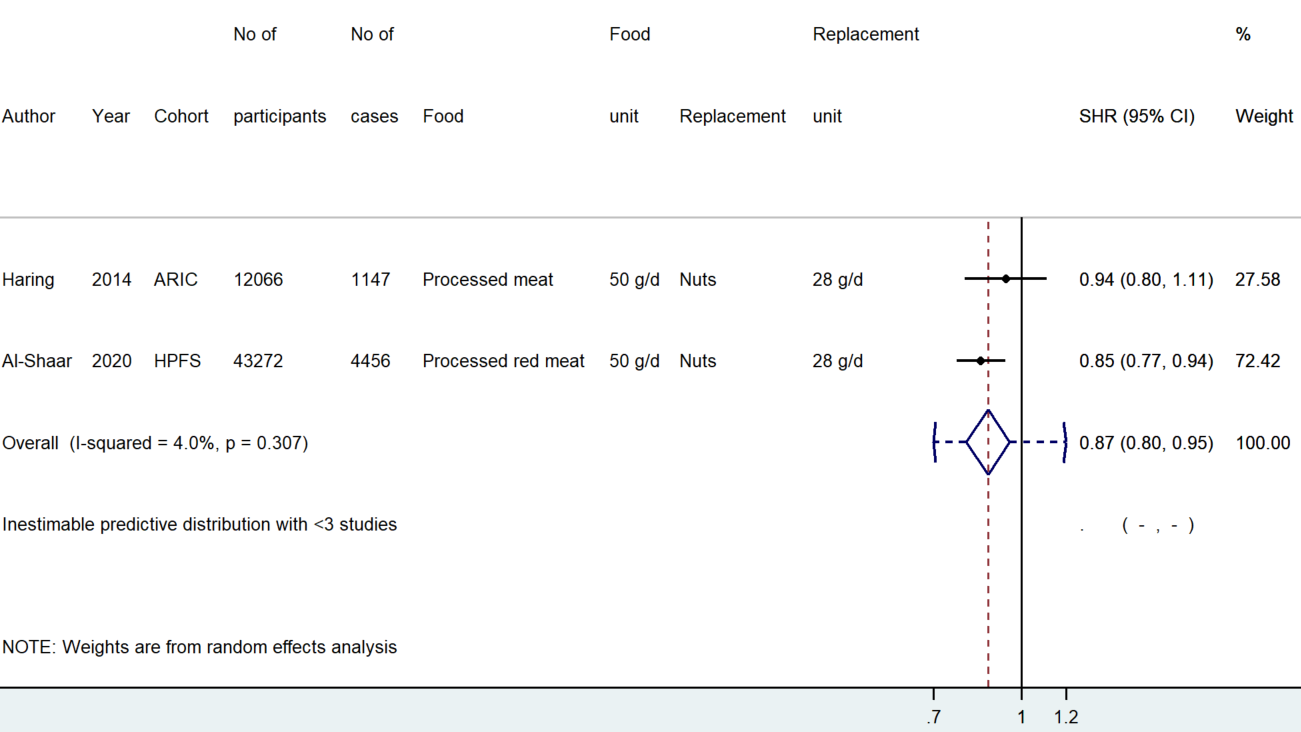  tau^2^ = 0.0002 |
| D) | 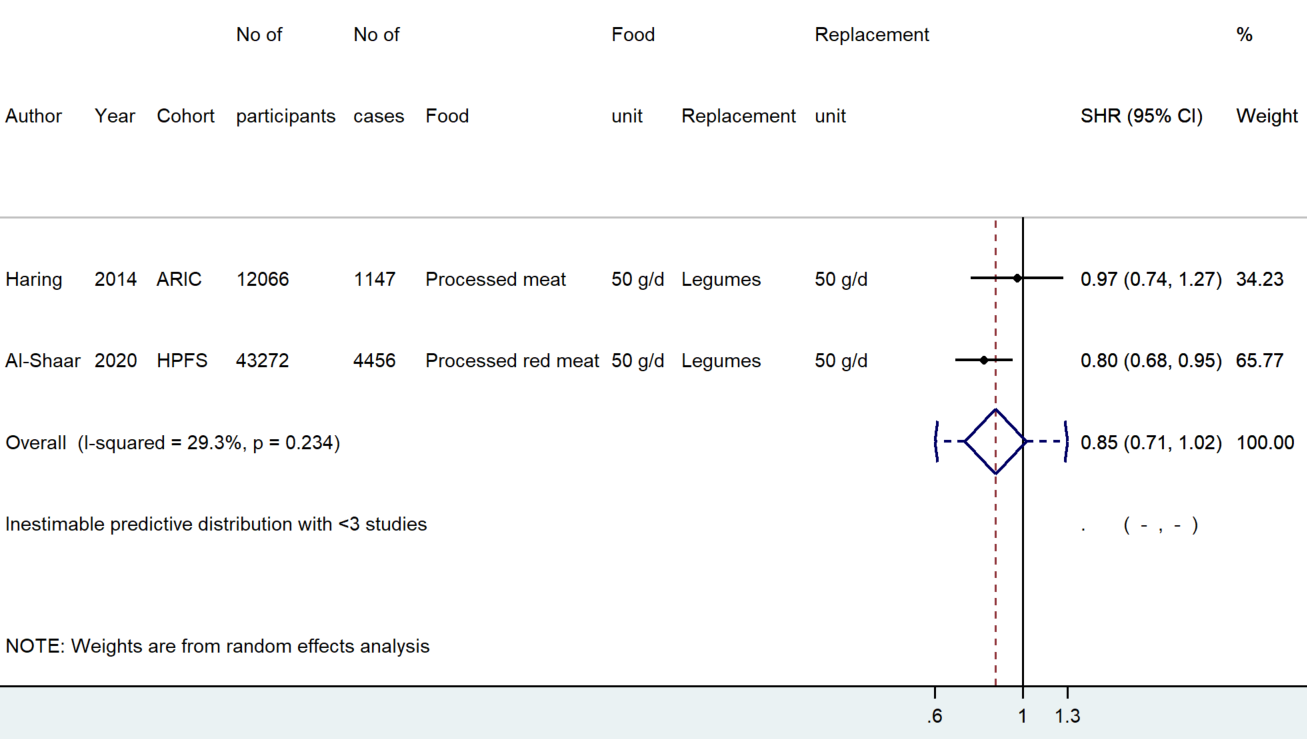  tau^2^ = 0.0054 |
| E) | 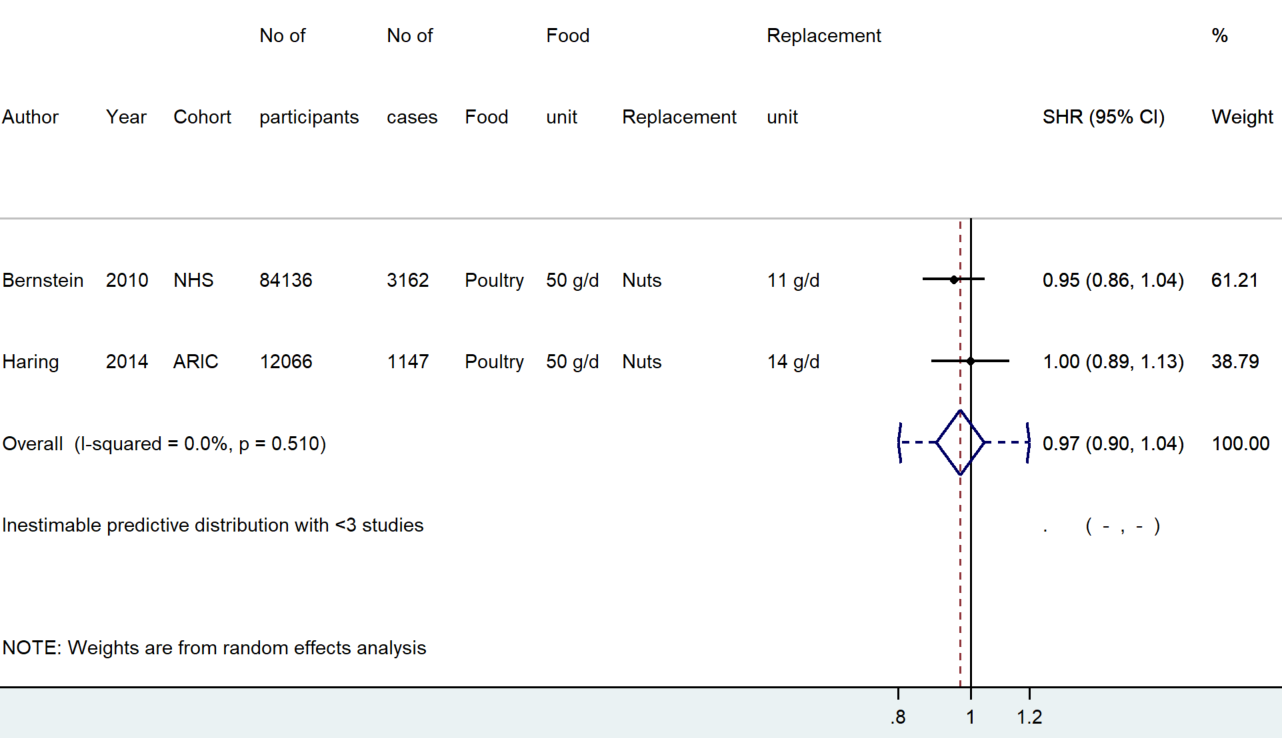  tau^2^ = 0.0000 |
| F) | 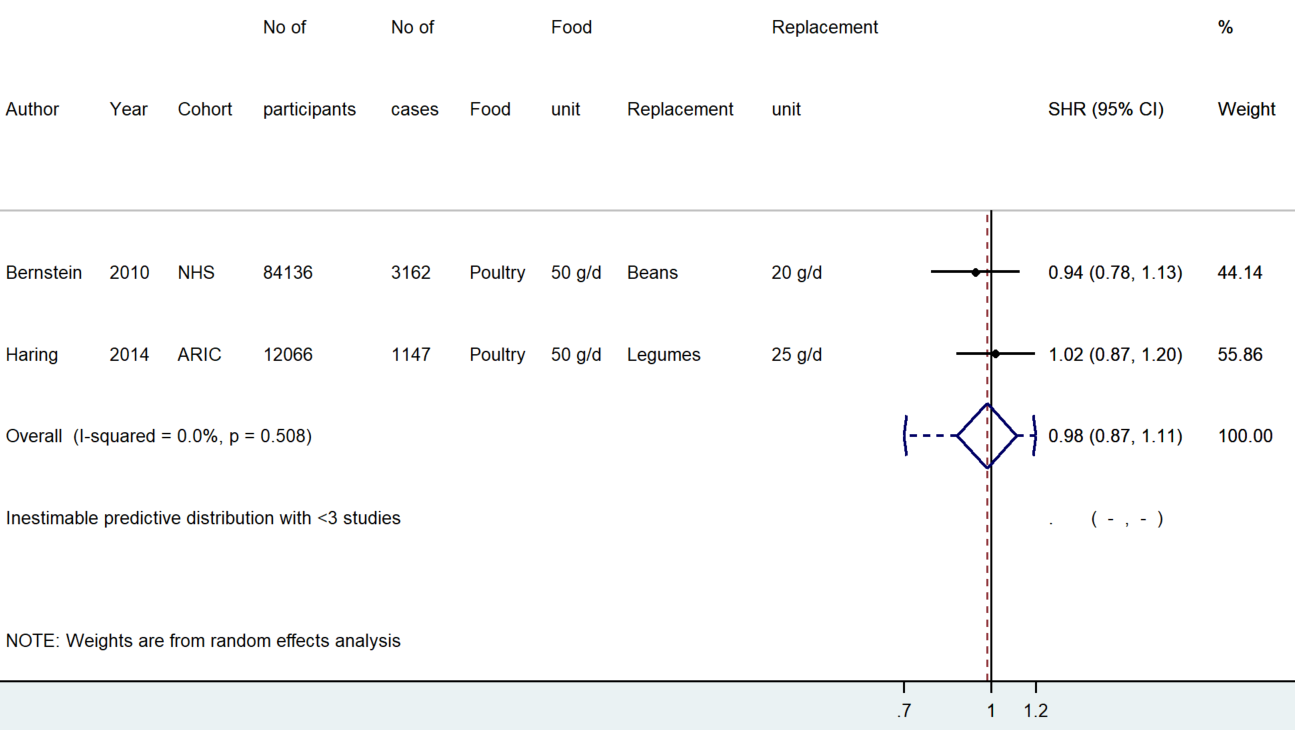  tau^2^ = 0.0000 |
| G) | 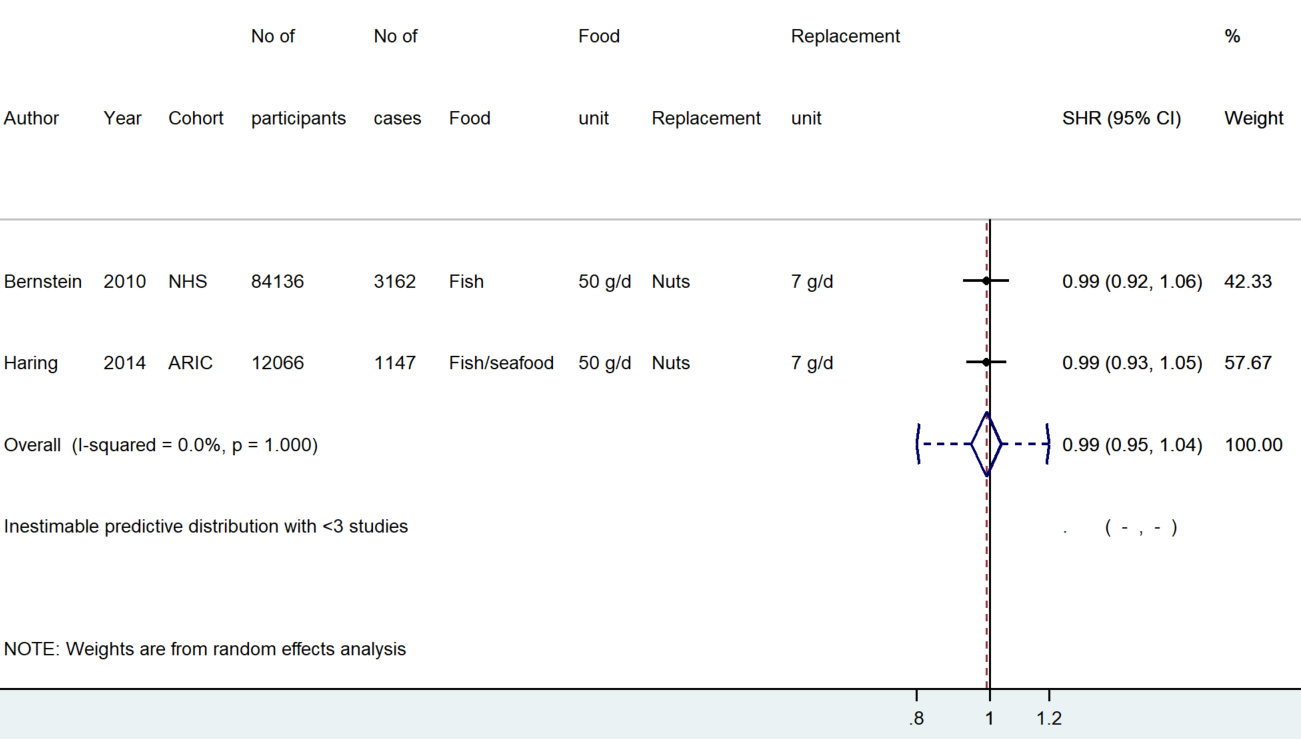  tau^2^ = 0.0000 |
| H) | 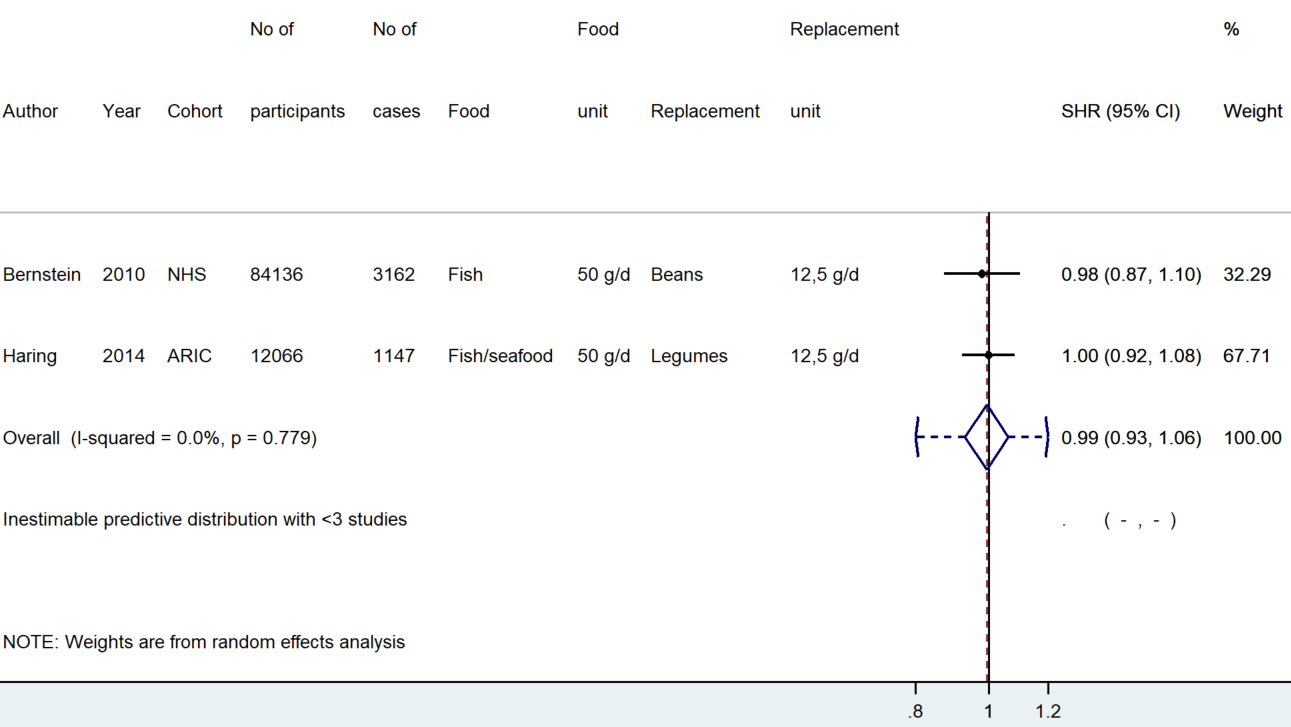  tau^2^ = 0.0000 |

**Fig. S6:** Forest plot regarding total diabetes for the substitution of A) eggs with nuts, B) eggs with legumes, and C) butter with olive oil

| A) | 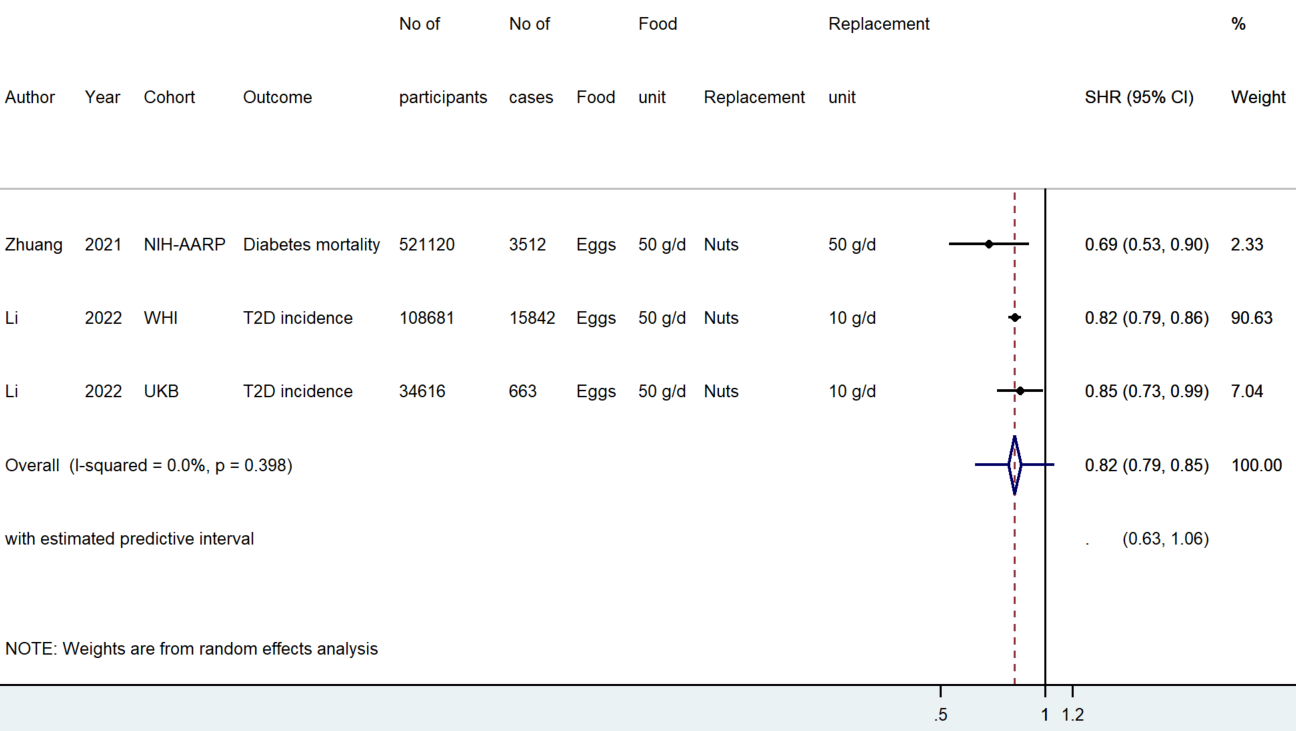  tau^2^ = 0.0002 |
| --- | --- |
| B) | 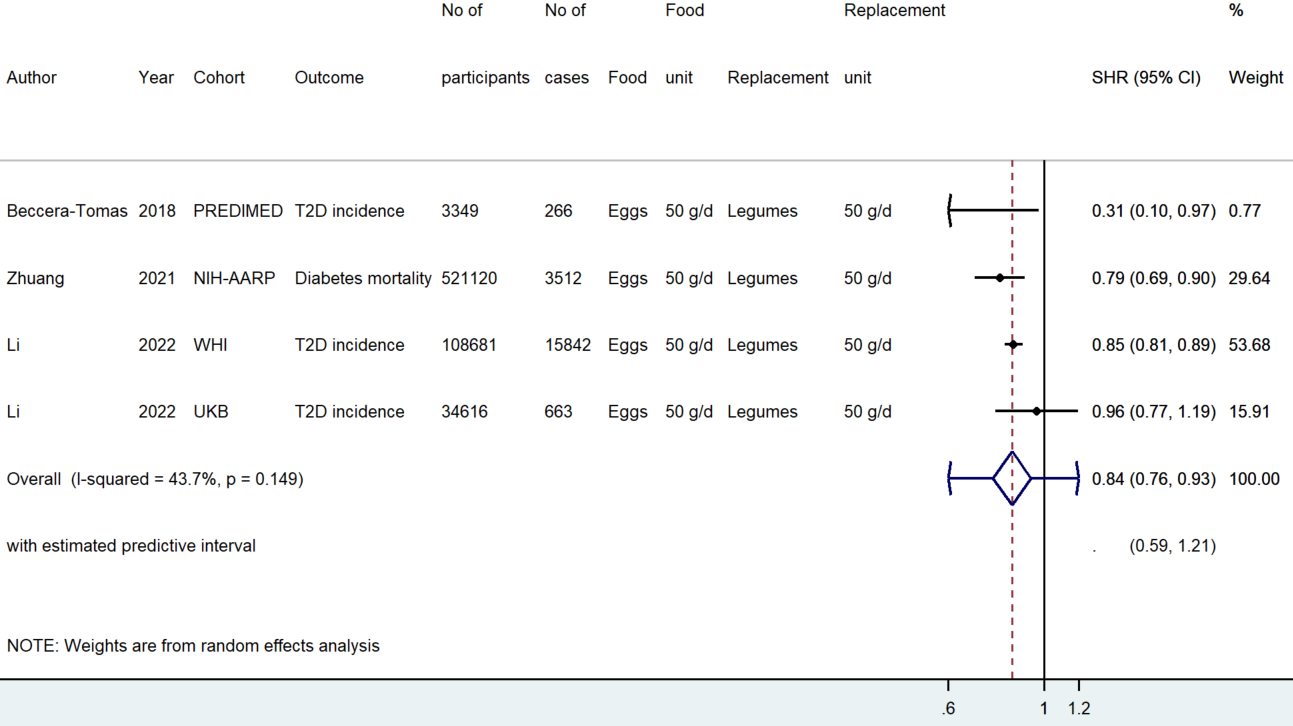  tau^2^ = 0.0044 |
| C) | 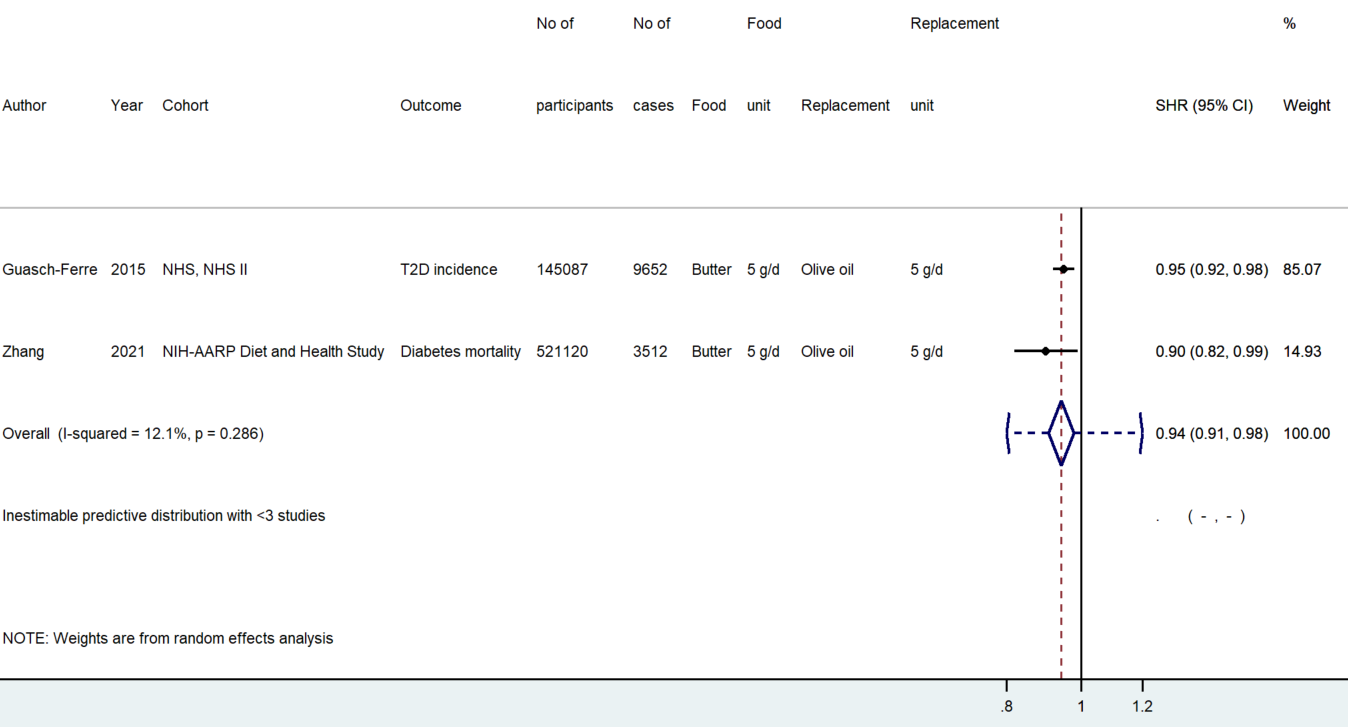  tau^2^ = 0.0002 |

**Fig. S7:** Forest plots regarding incidence of type 2 diabetes for the substitution of A) red meat with nuts, B) red meat with legumes, C) red meat with whole grains / cereals, D) processed meat with nuts, E) processed meat with legumes, F) processed meat with whole grain / cereals, G) poultry with nuts, H) poultry with legumes, I) poultry with whole grains, J) eggs with nuts, K) eggs with legumes and L) eggs with whole grains

| A) | 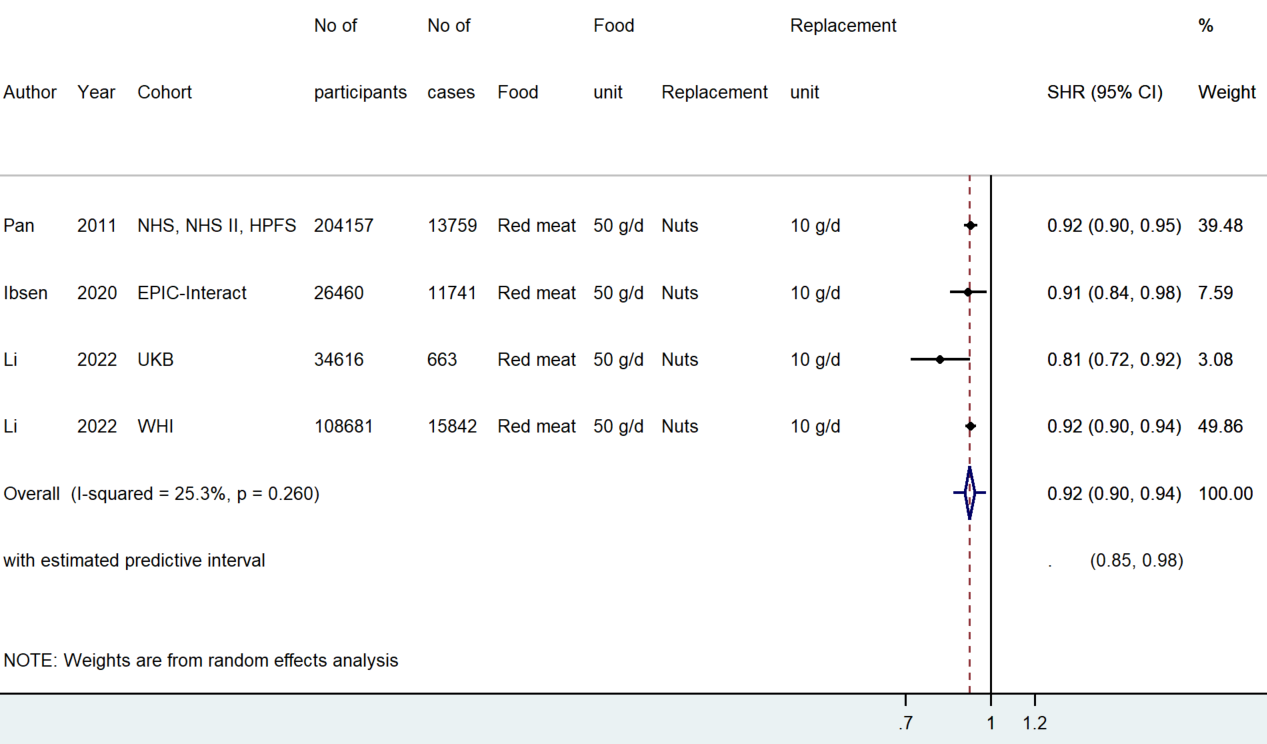  tau^2^ = 0.0001 |
| --- | --- |
| B) | 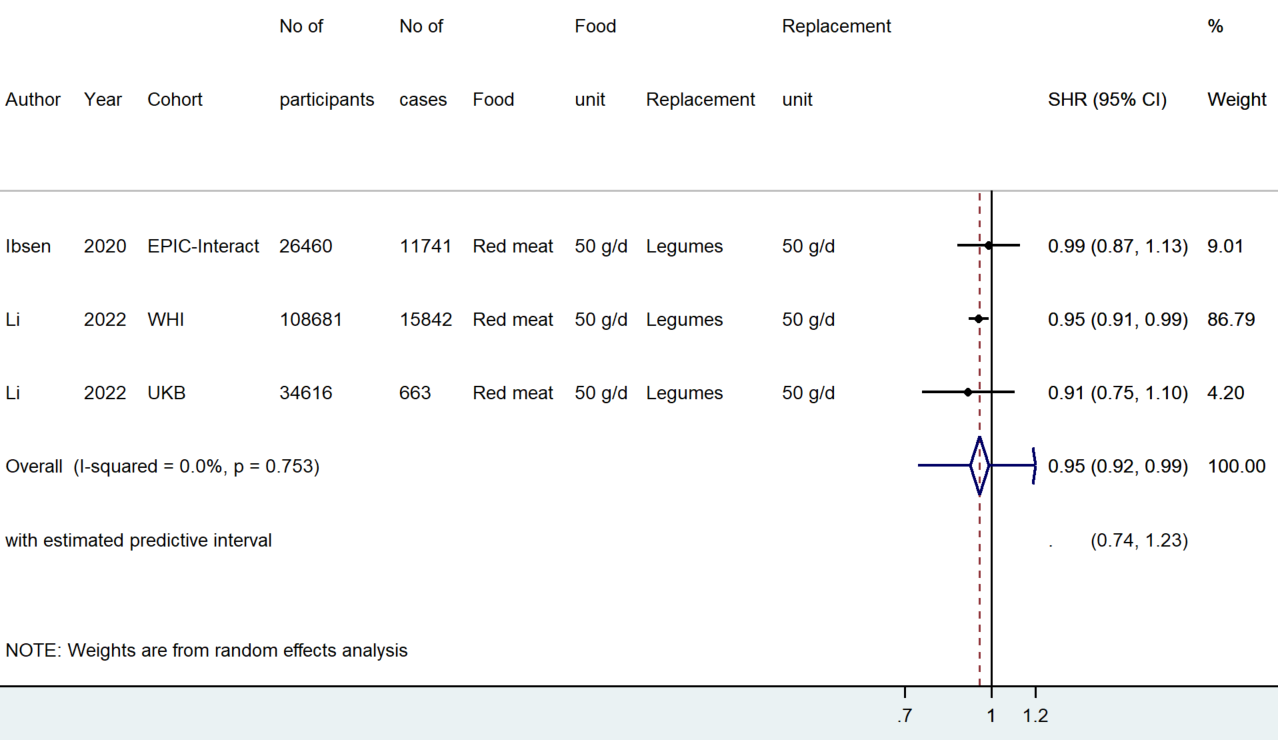  tau^2^ = 0.0000 |
| C) | 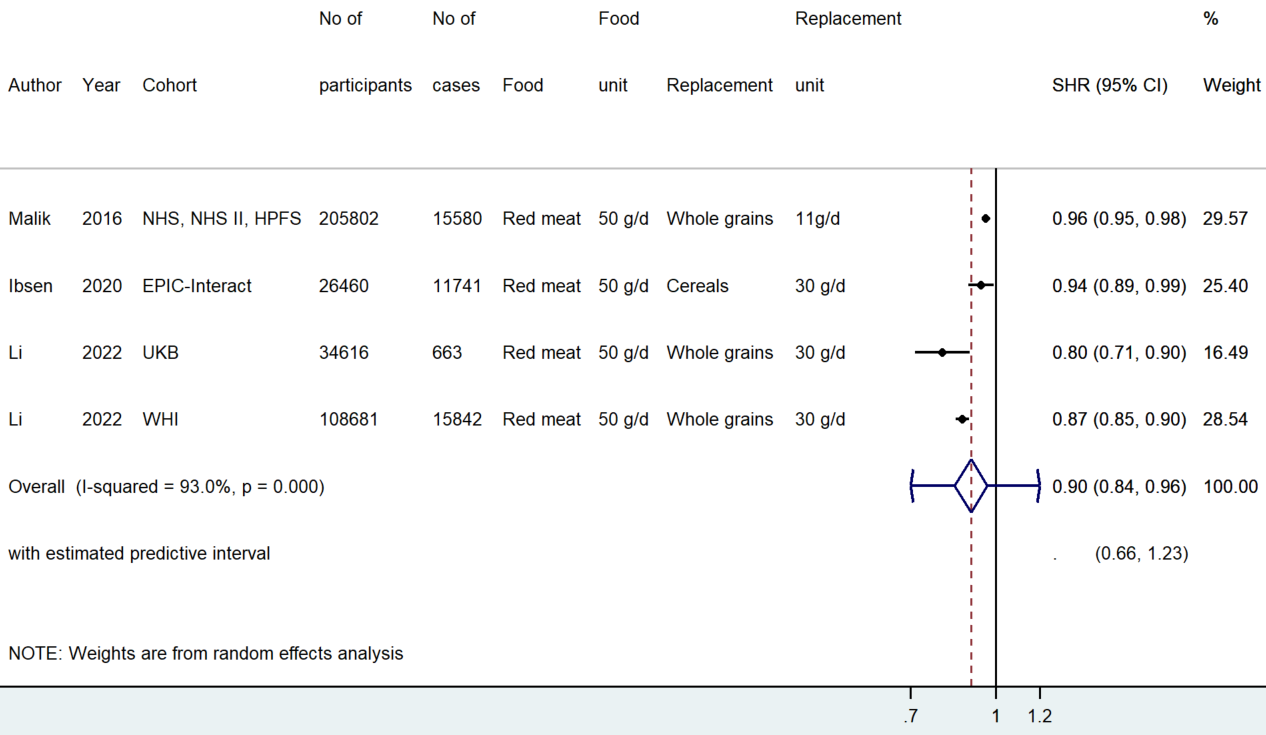  tau^2^ = 0.0040 |
| D) | 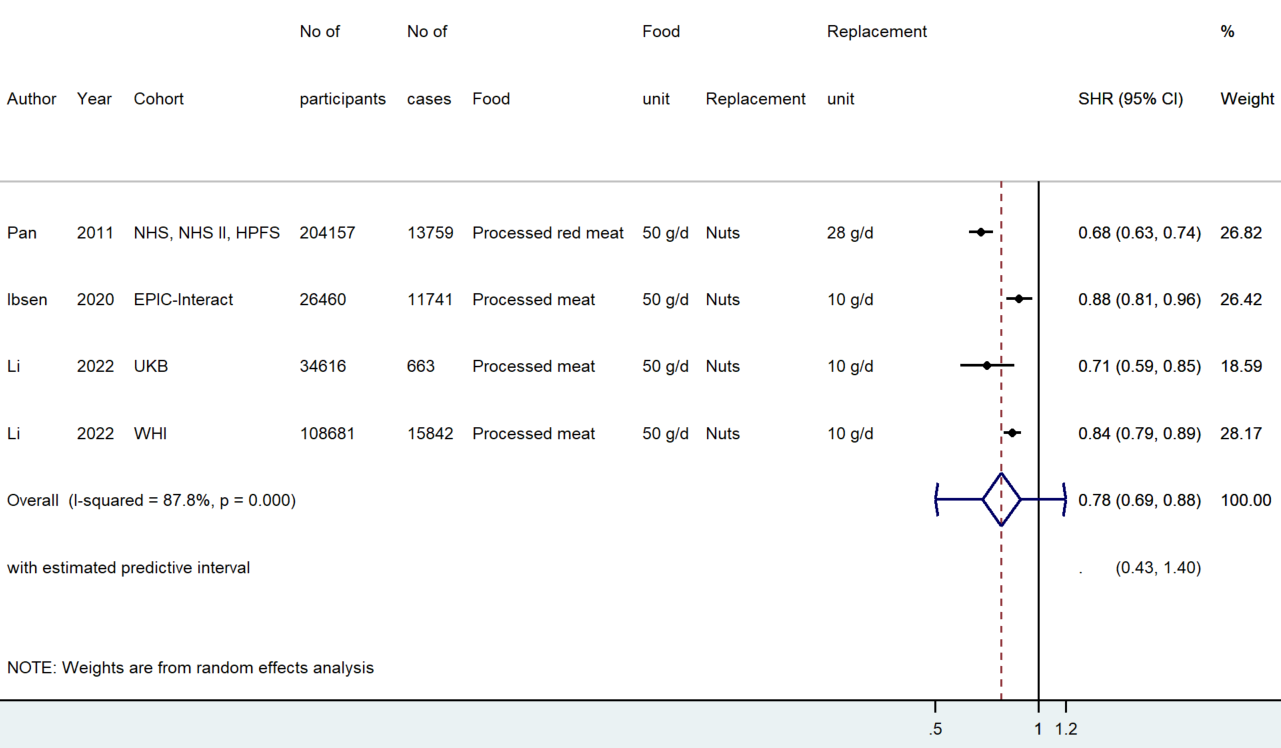  tau^2^ = 0.0141 |
| E) | 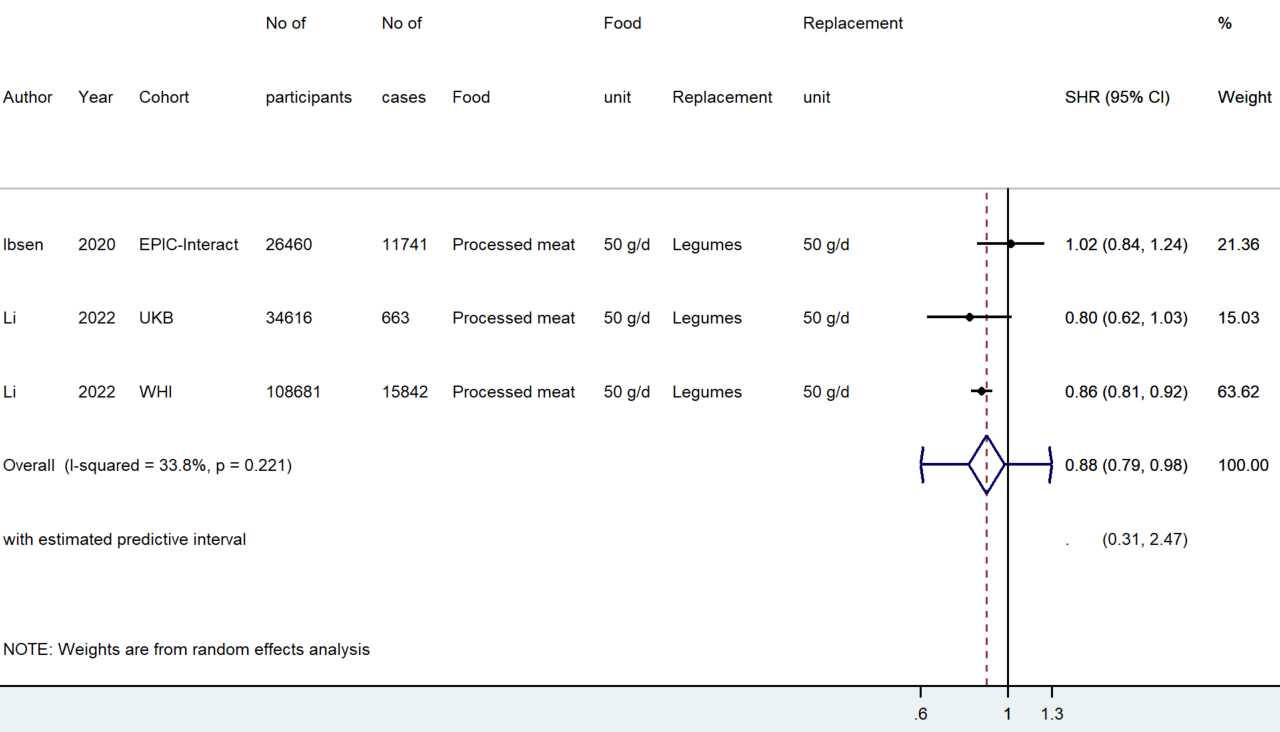  tau^2^ = 0.0036 |
| F) | 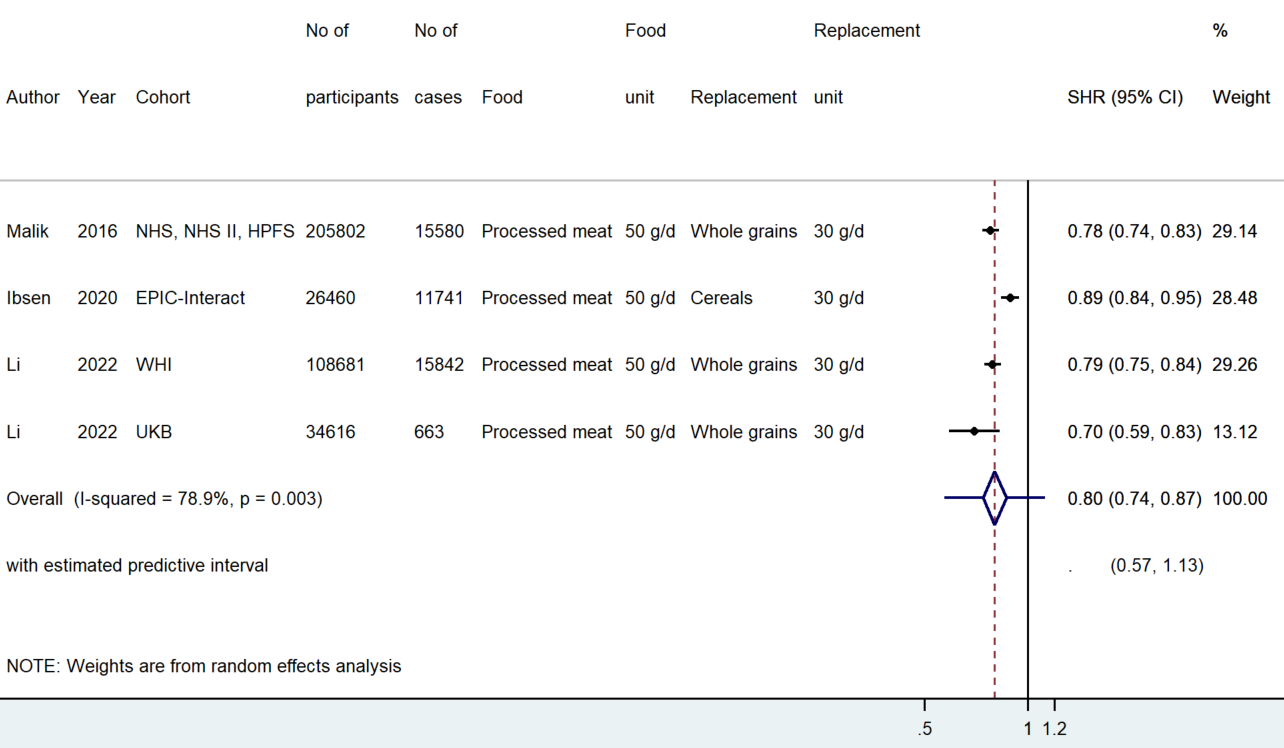  tau^2^ = 0.0046 |
| G) | 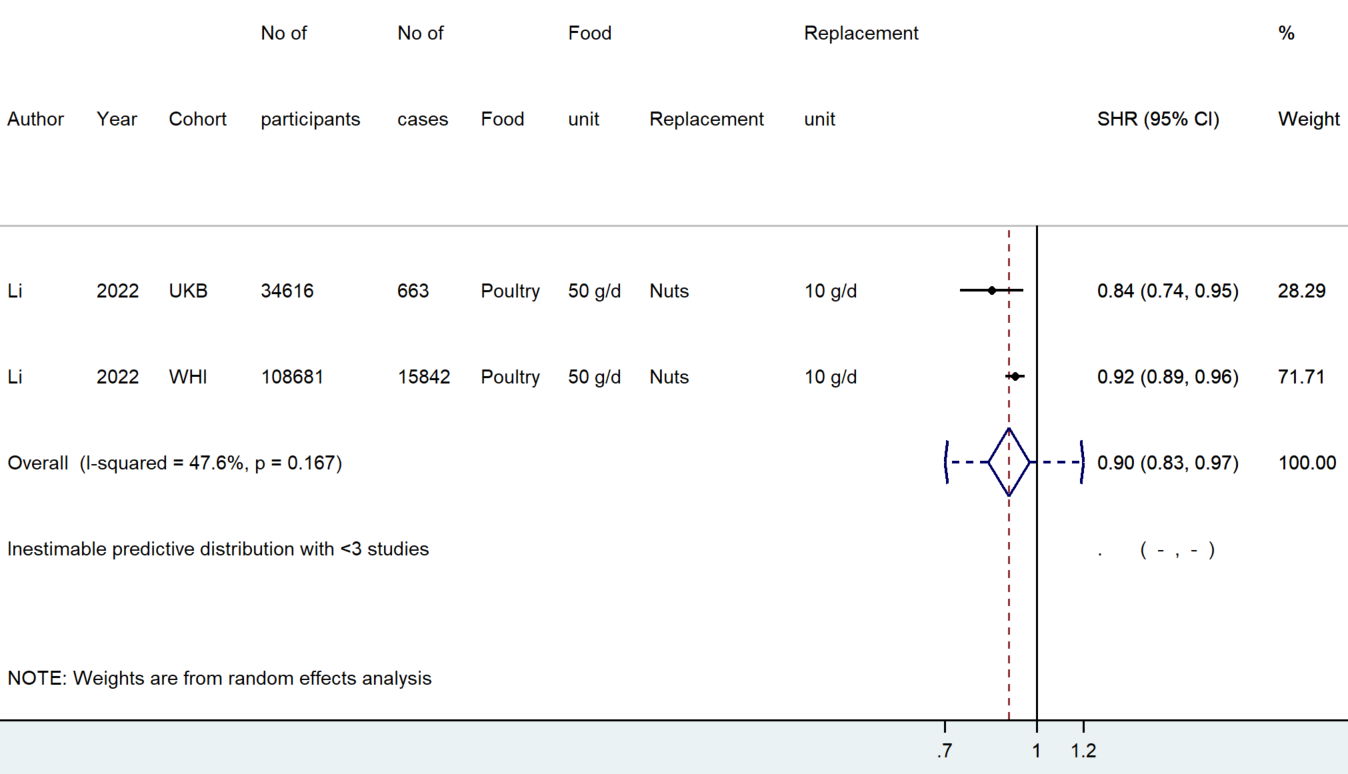  tau^2^ = 0.0020 |
| H) | 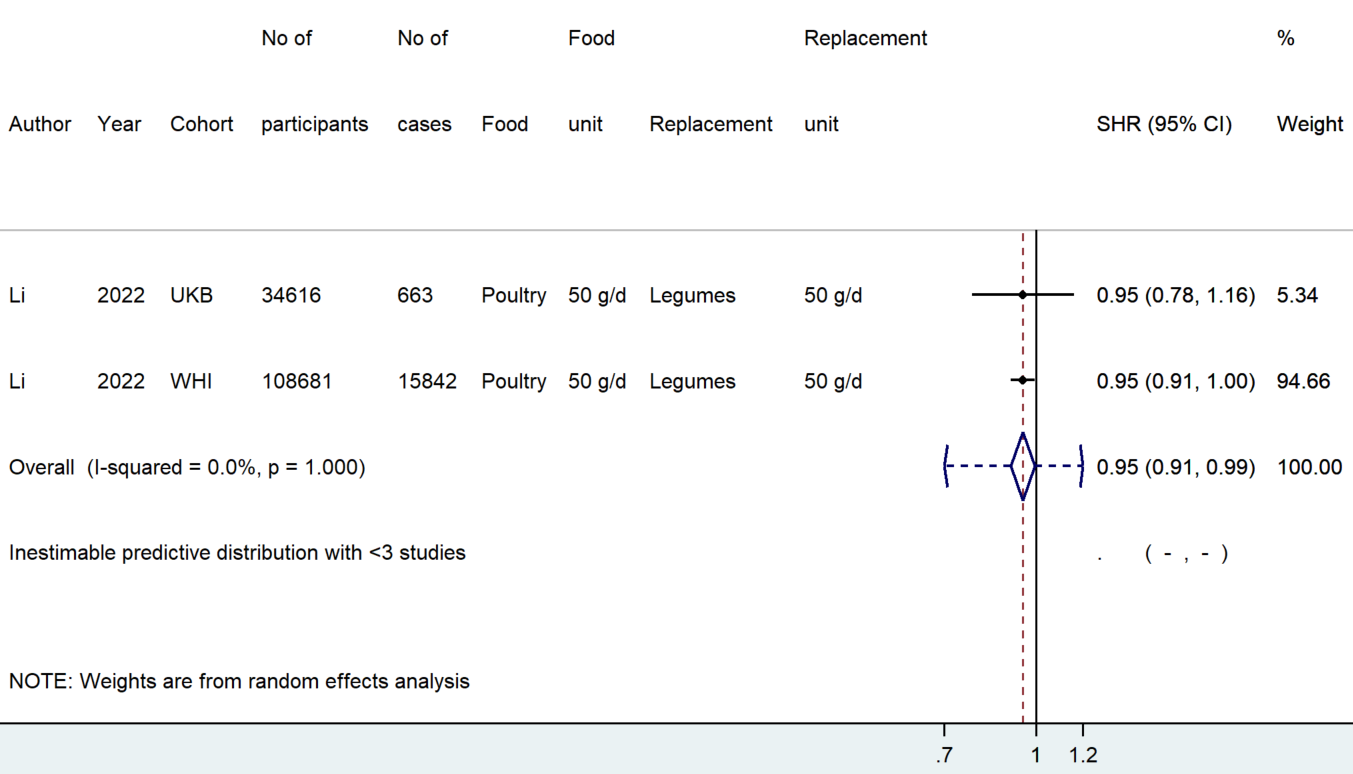  tau^2^ = 0.0000 |
| I) | 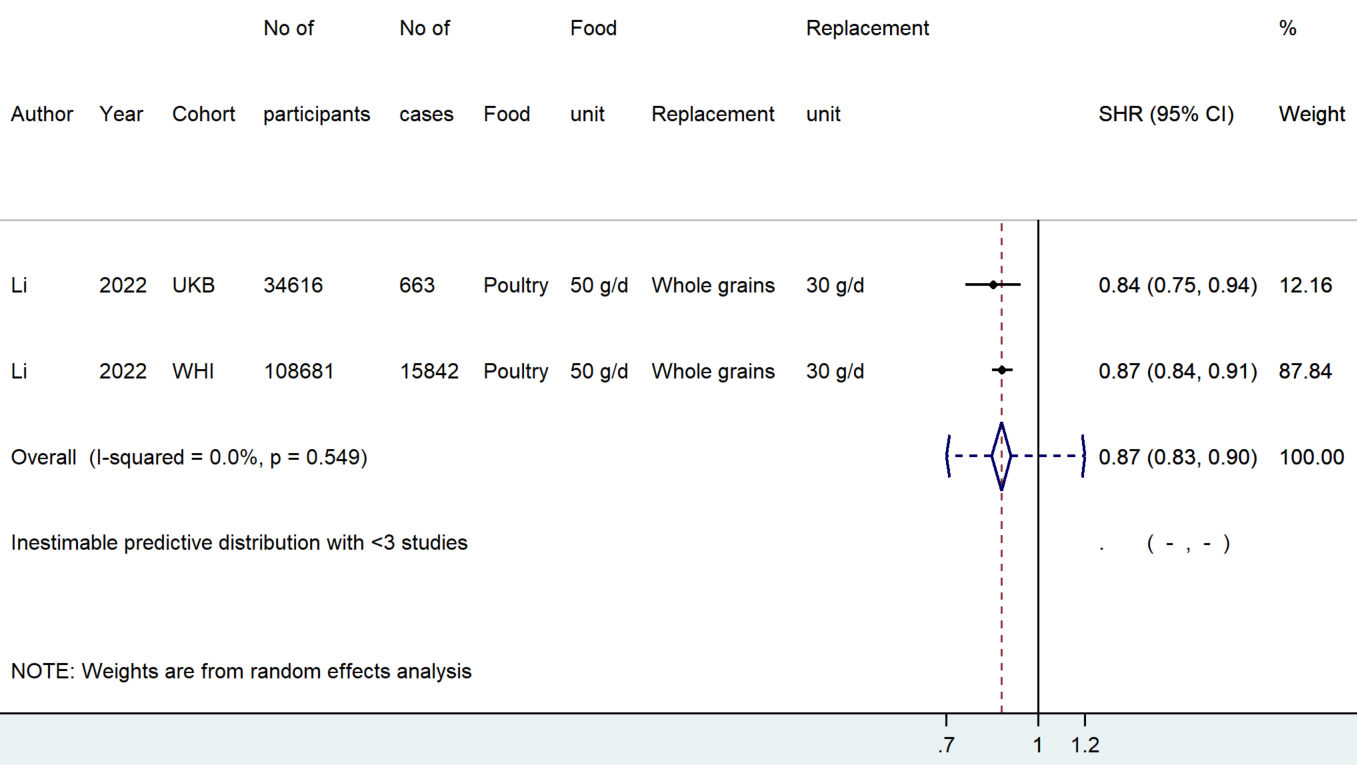  tau^2^ = 0.0000 |
| J) | 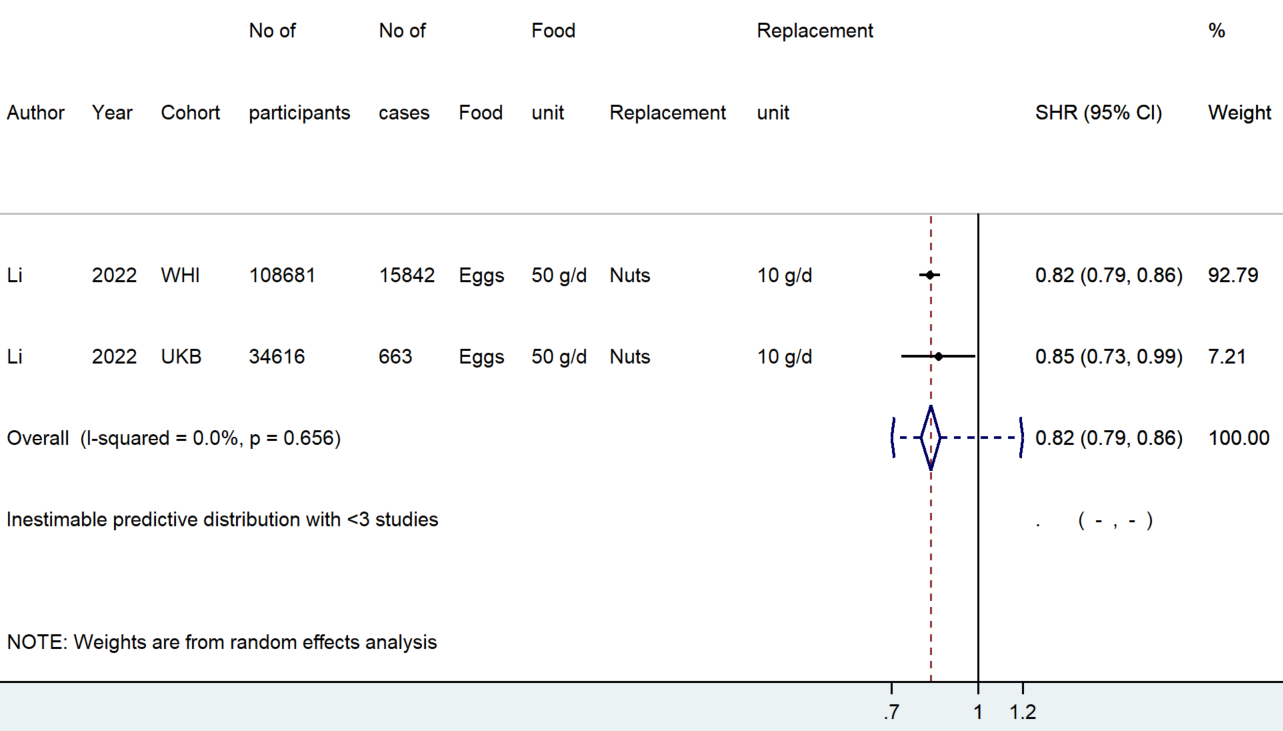  tau^2^ = 0.0000 |
| K) | 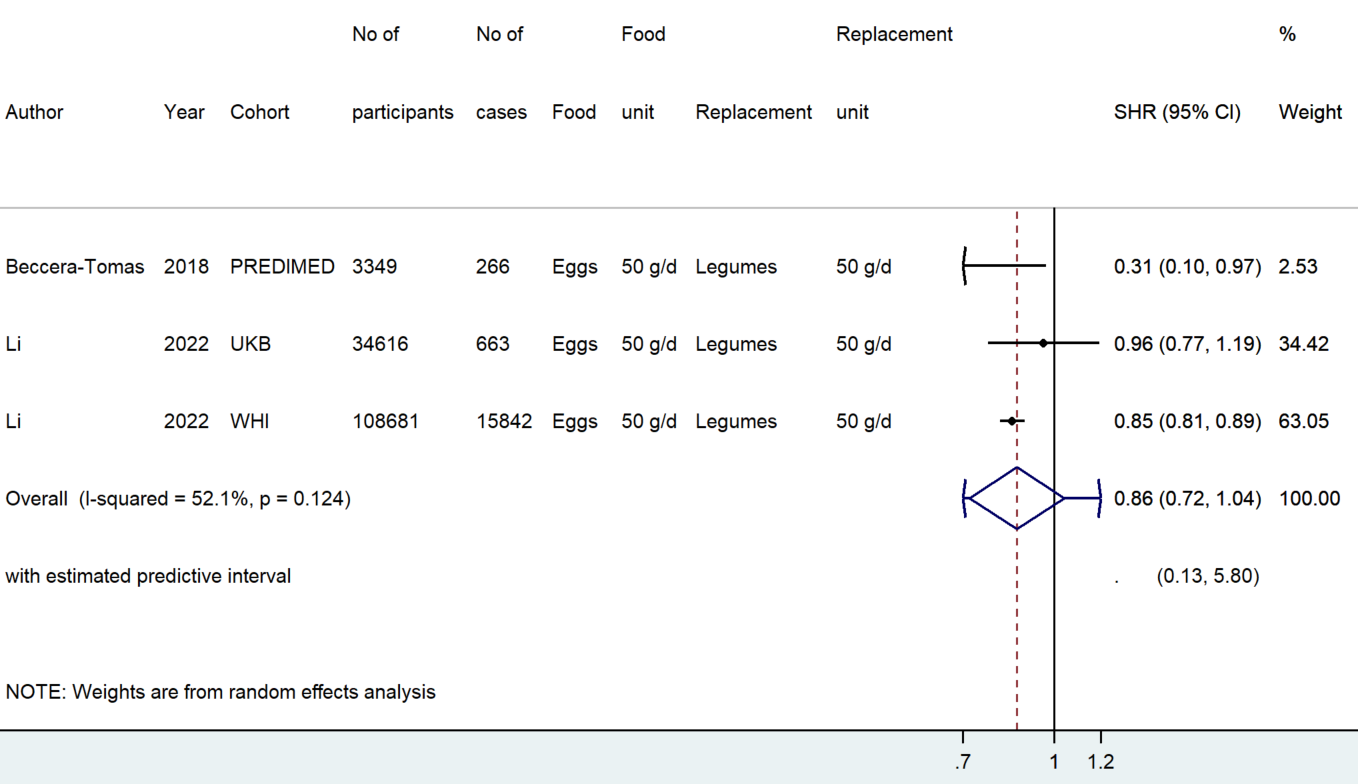  tau^2^ = 0.0136 |
| L) | 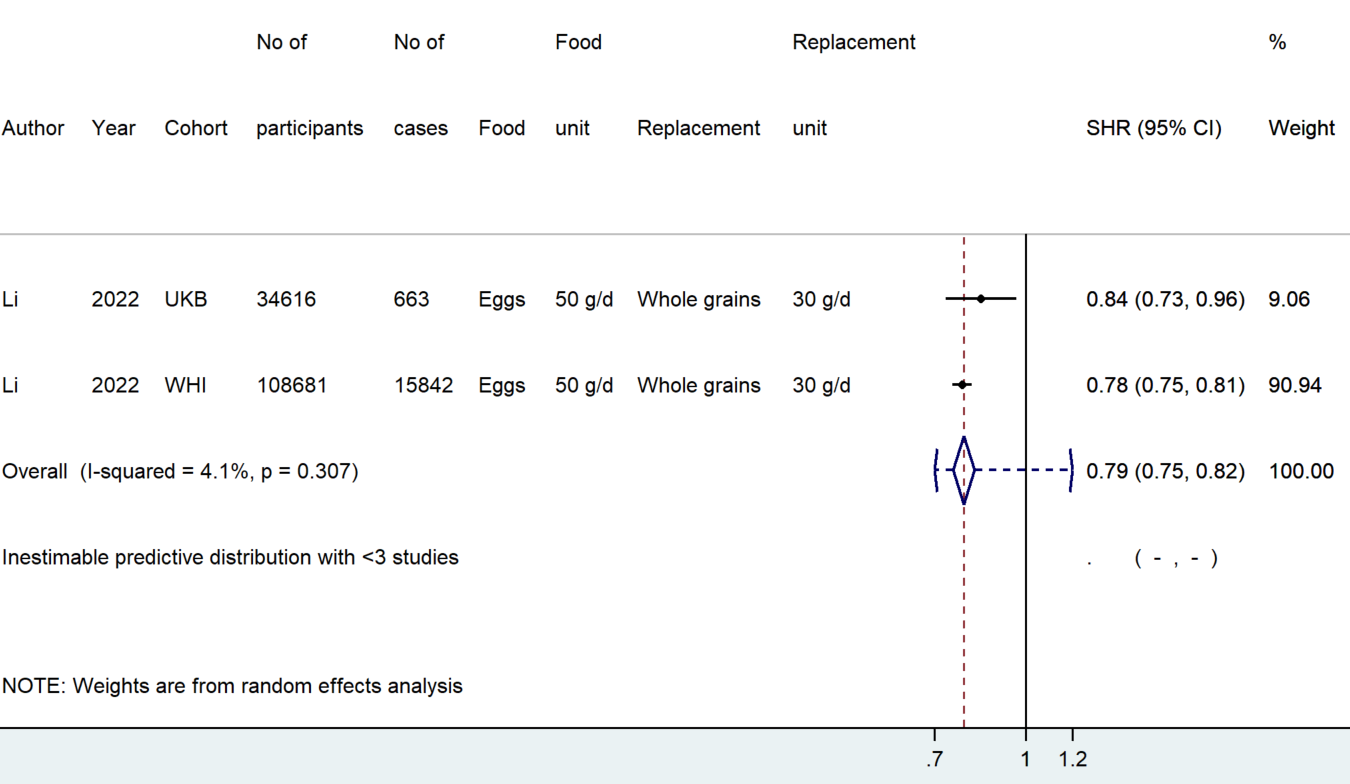  tau^2^ = 0.0001 |

**Fig. S8:** Forest plots regarding all-cause mortality for the substitution of A) red meat with nuts, B) red meat with legumes, C) red meat with whole grains, D) red meat with cereals, E) red meat with vegetables, F) processed meat with nuts, G) processed meat with legumes, H) unprocessed red meat with nuts, I) unprocessed red meat with legumes, J) poultry with nuts, K) fish with nuts, L) dairy with nuts / legumes, M) dairy with nuts, N) eggs with nuts, O) eggs with legumes, P) butter with olive oil

| A) | 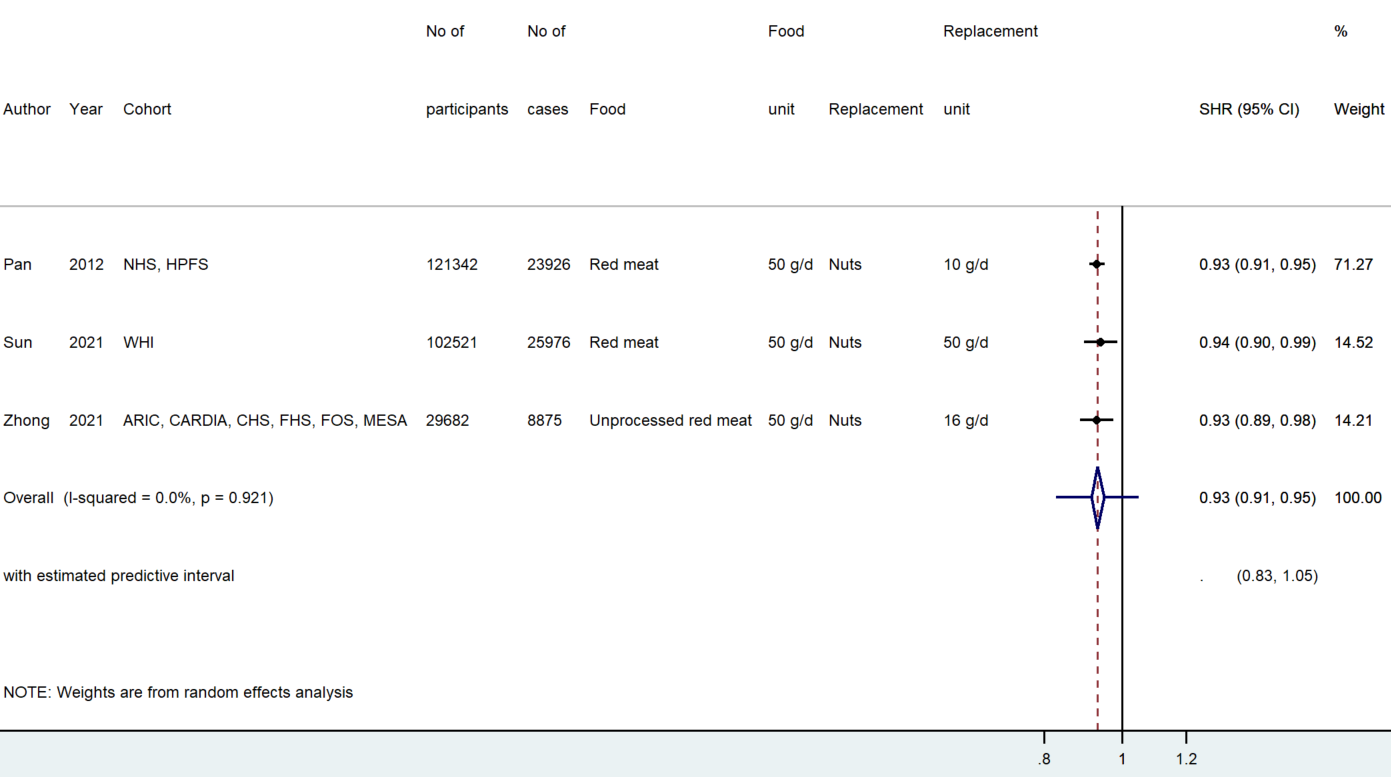  tau^2^ = 0.0010 |
| --- | --- |
| B) | 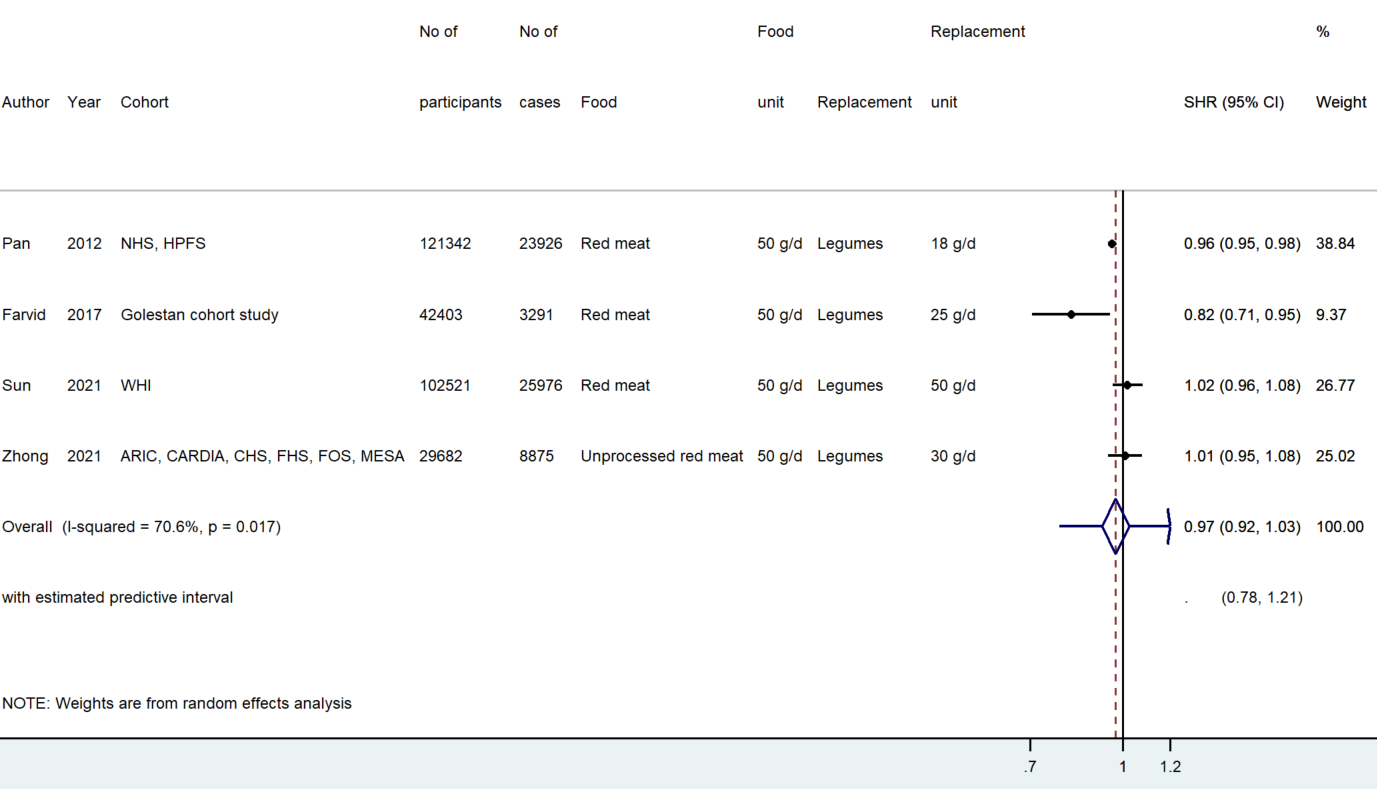  tau^2^ = 0.0018 |
| C) | 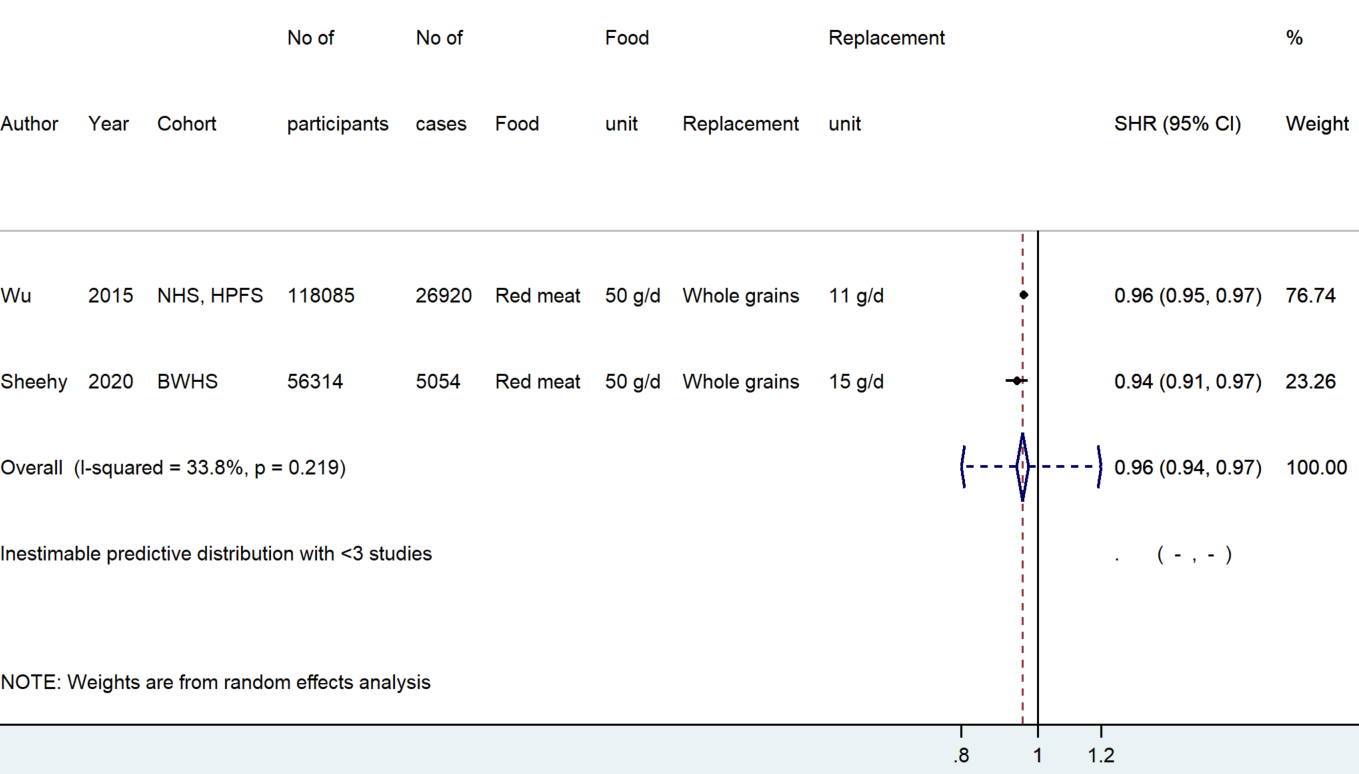  tau^2^ = 0.0001 |
| D) | 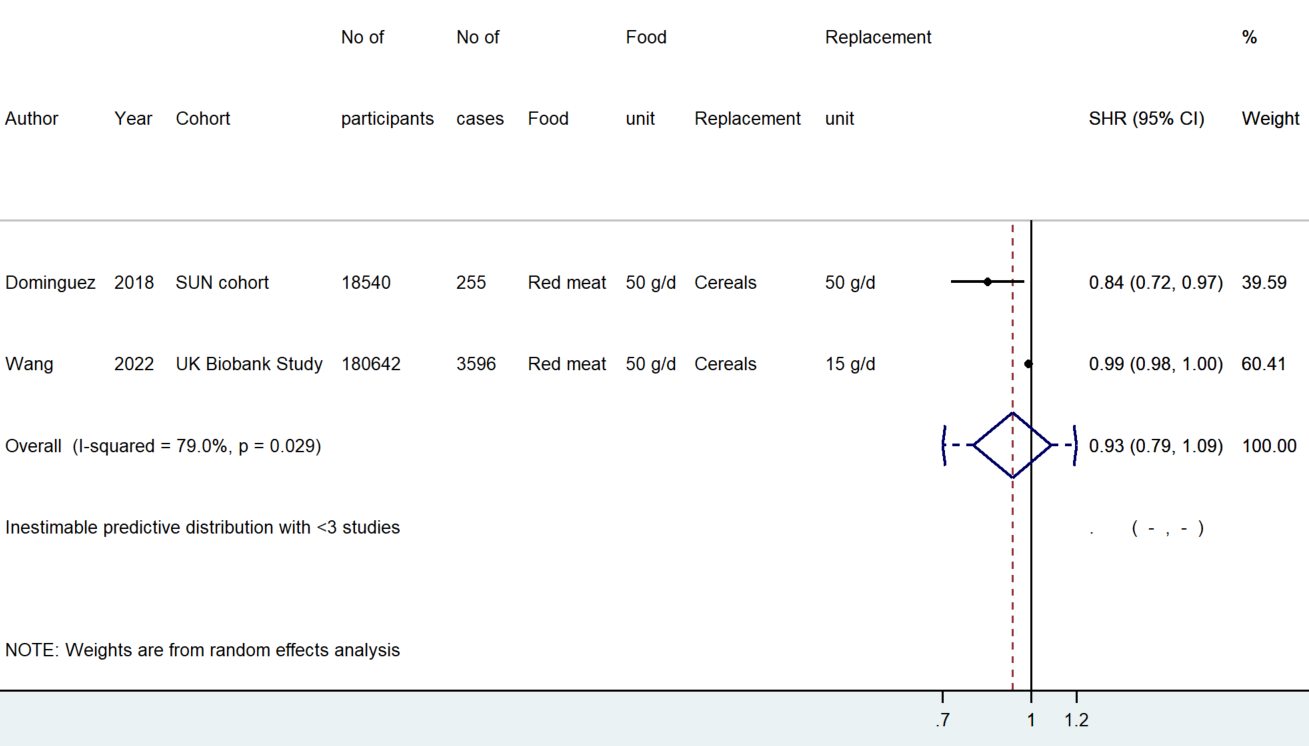  tau^2^ = 0.0107 |
| E) | 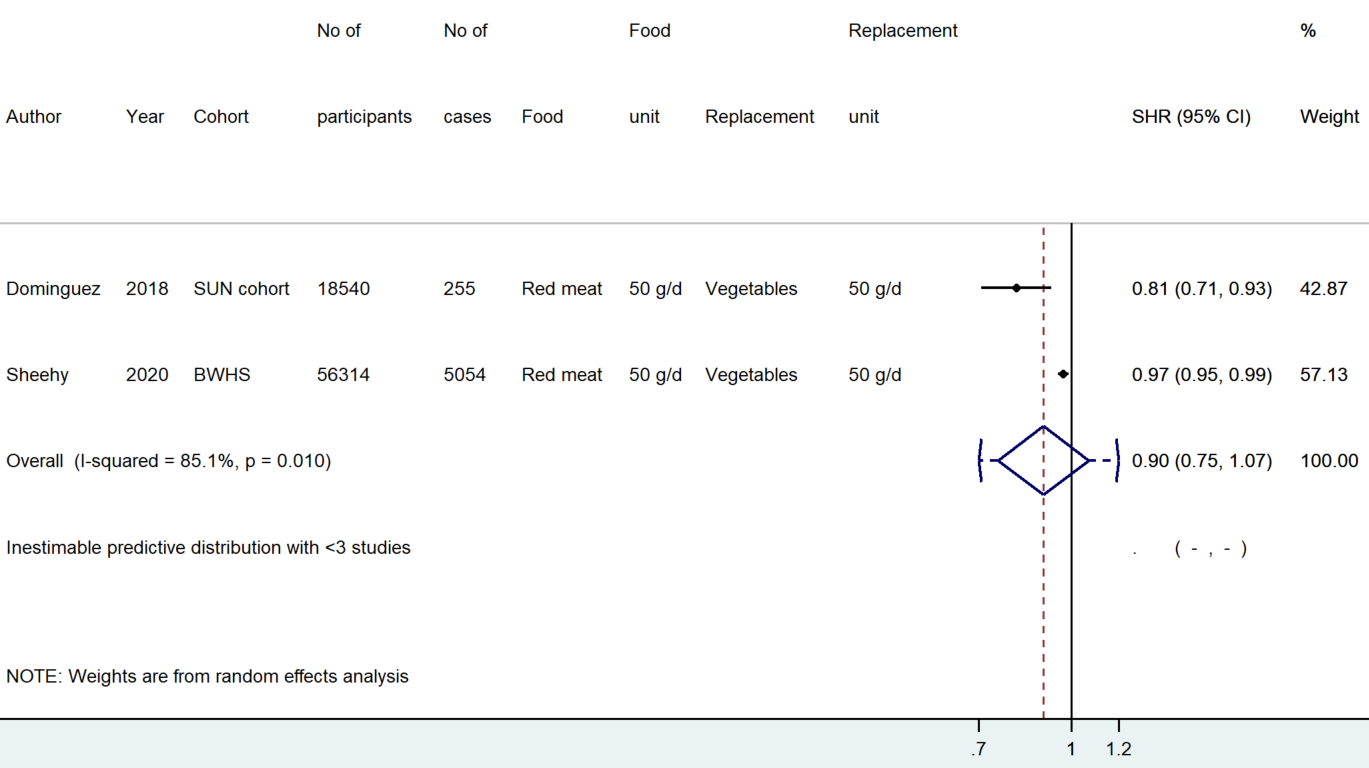  tau^2^ = 0.0138 |
| F) | 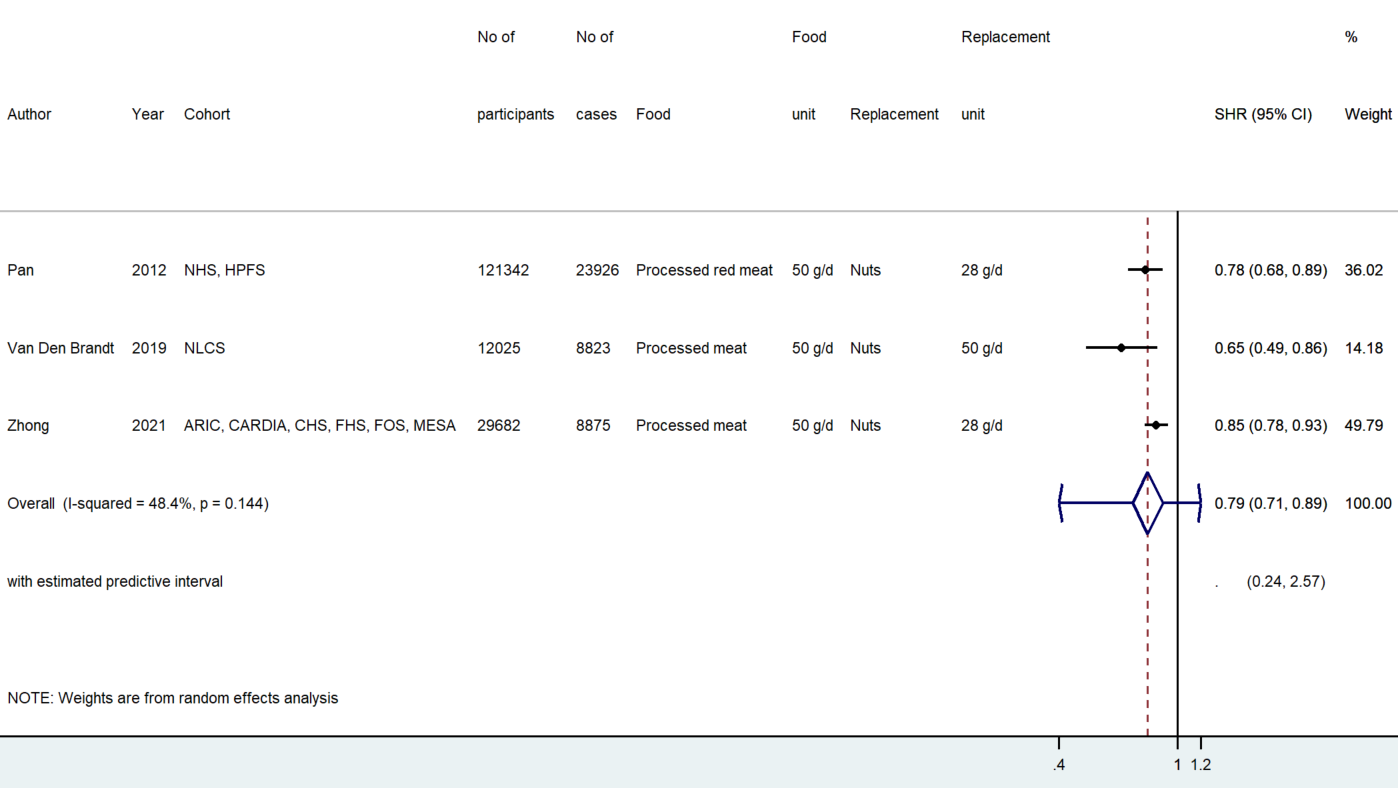  tau^2^ = 0.0050 |
| G) | 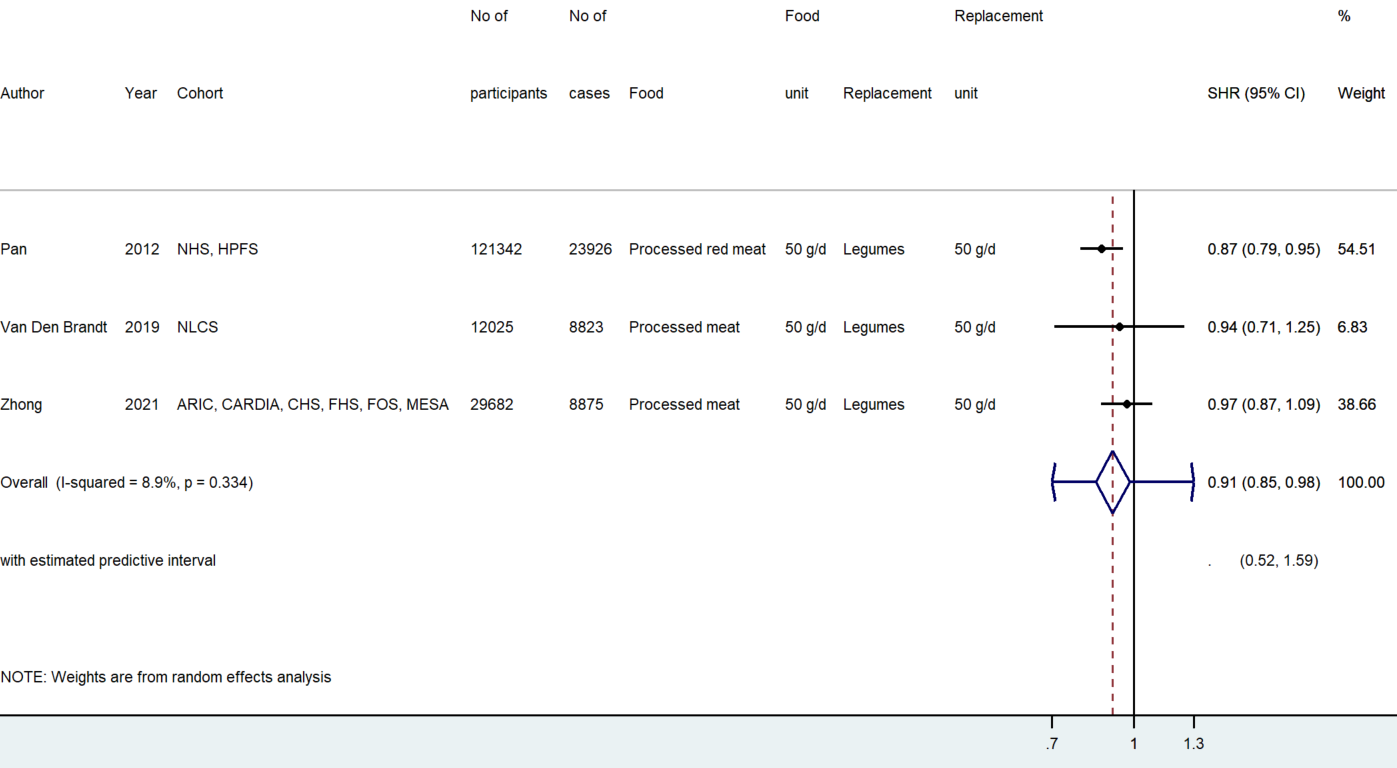  tau^2^ = 0.0005 |
| H) | 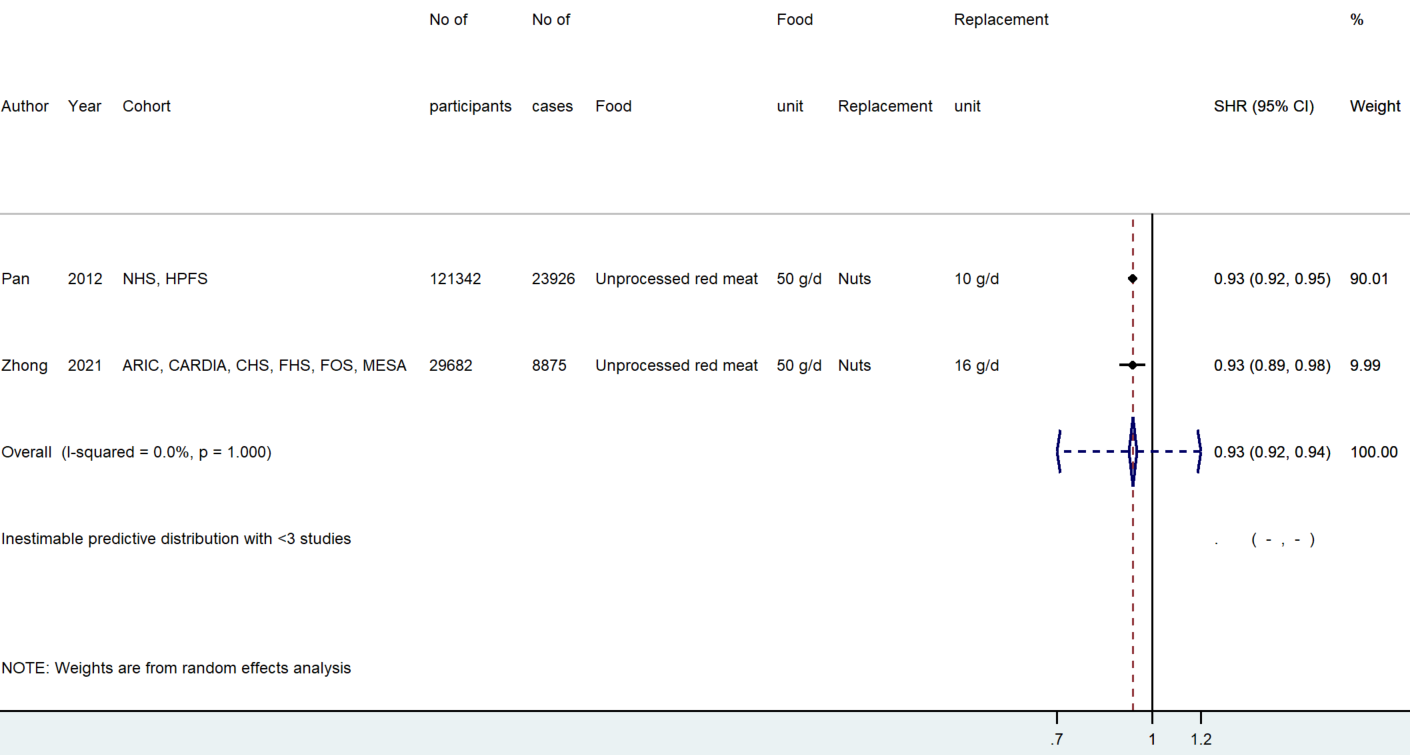  tau^2^ = 0.0000 |
| I) | 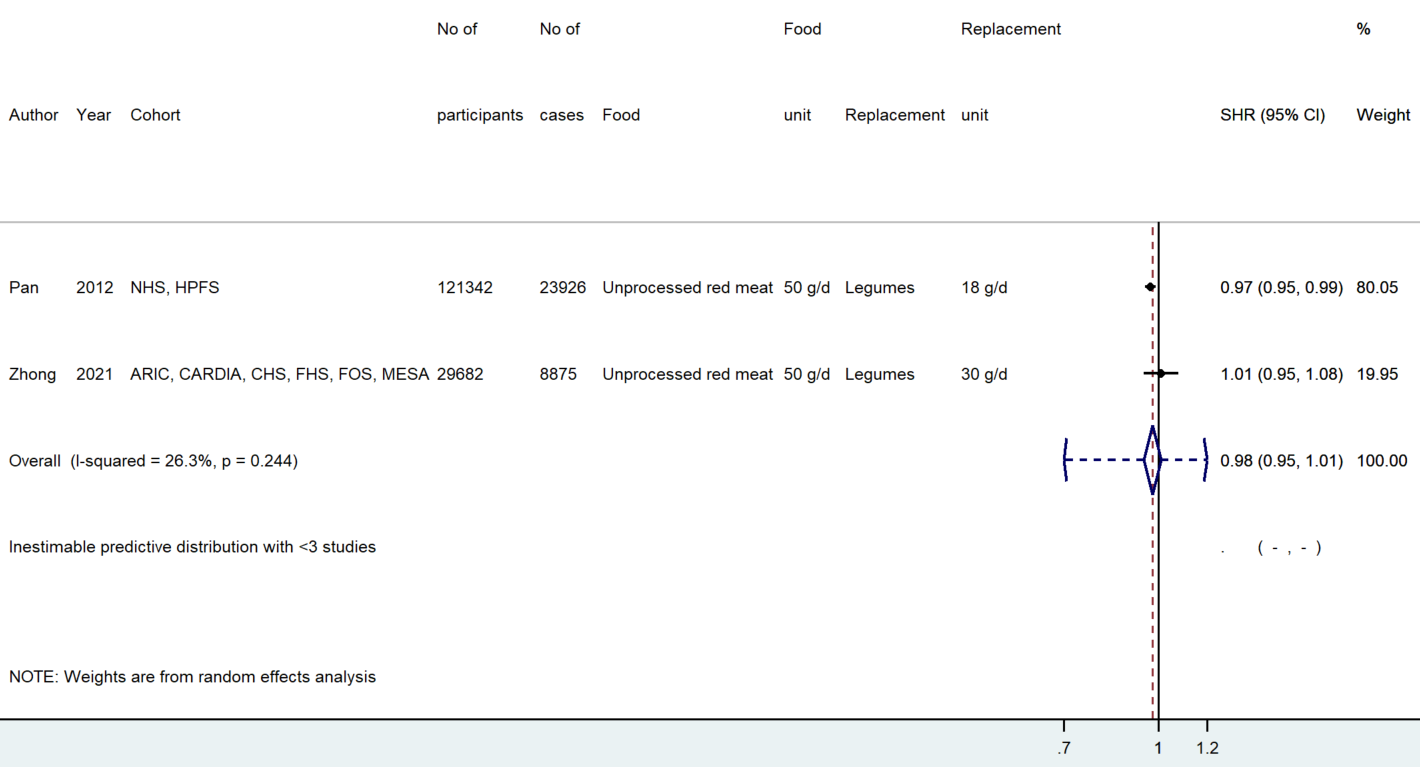  tau^2^ = 0.0002 |
| J) | 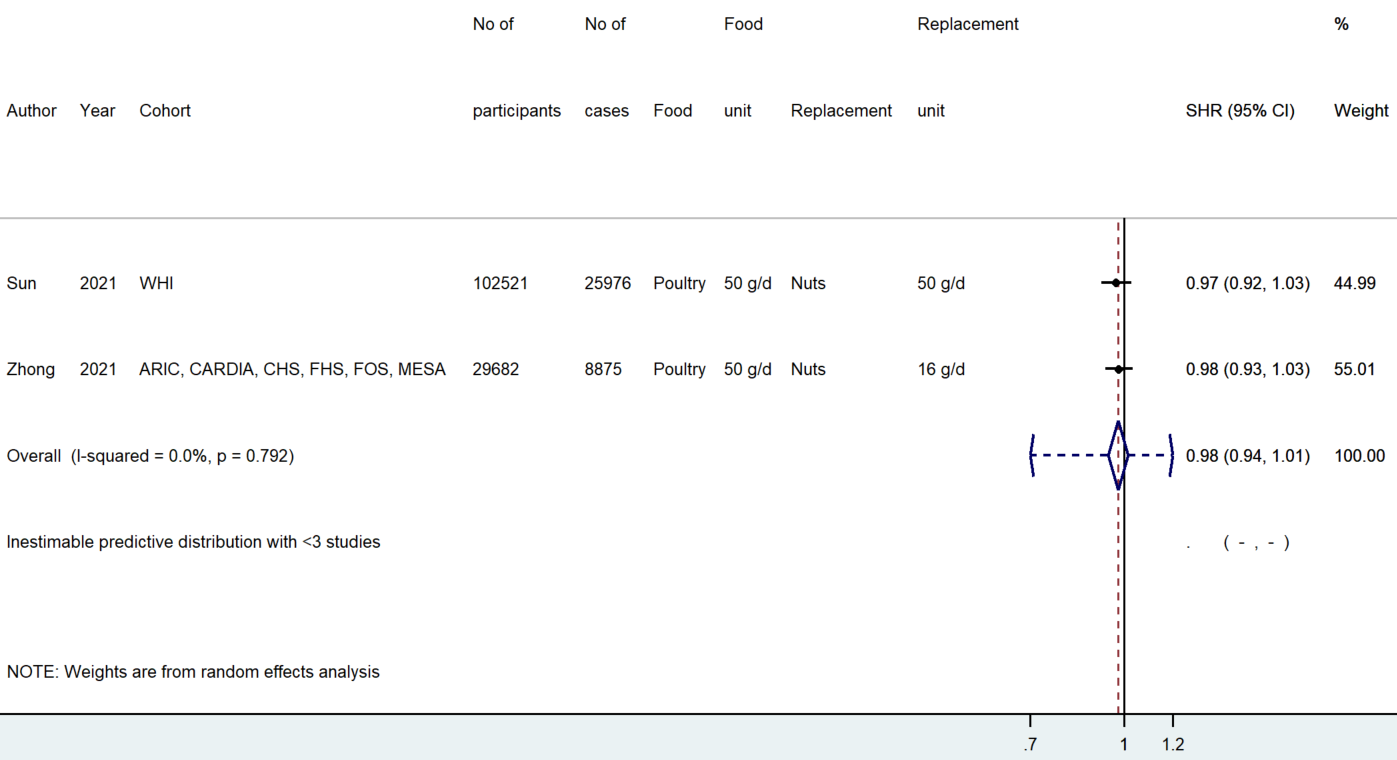  tau^2^ = 0.0000 |
| K) | 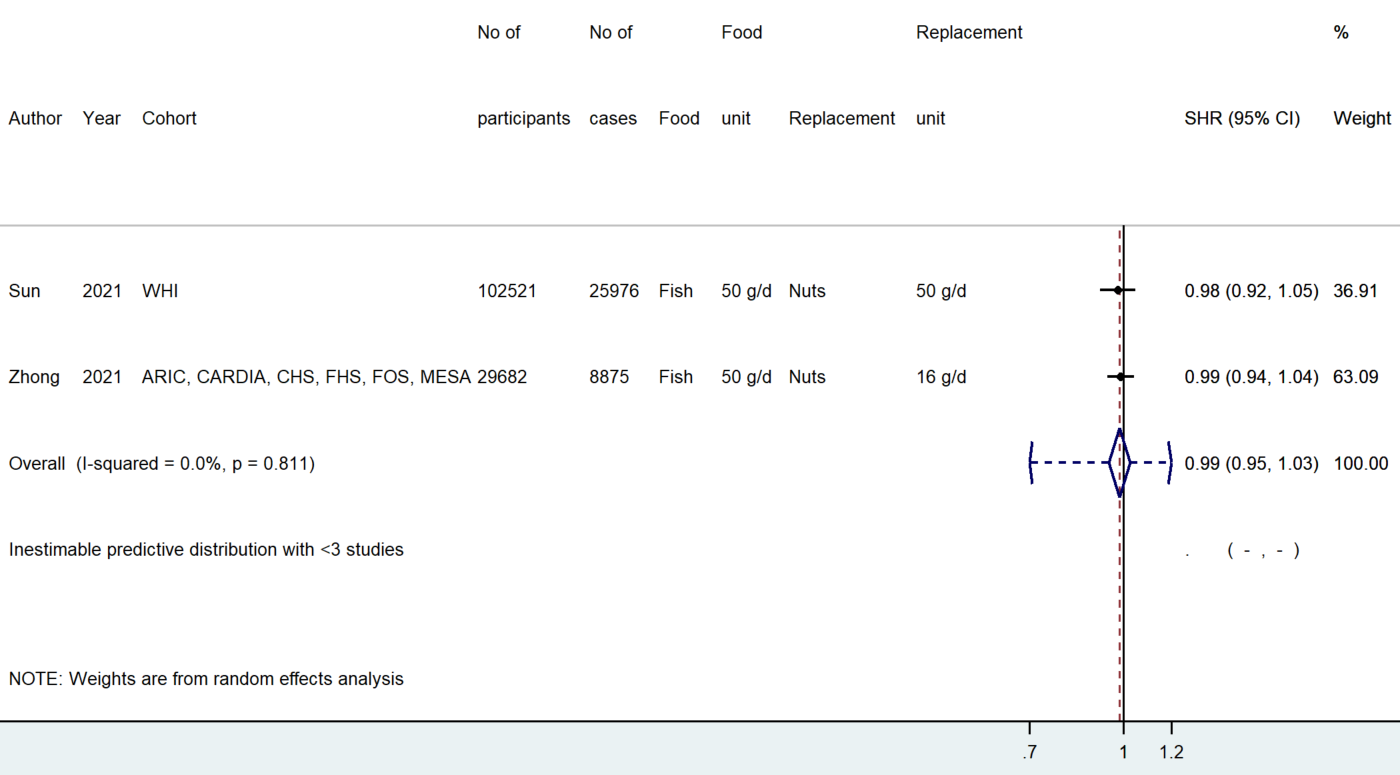  tau^2^ = 0.0000 |
| L) | 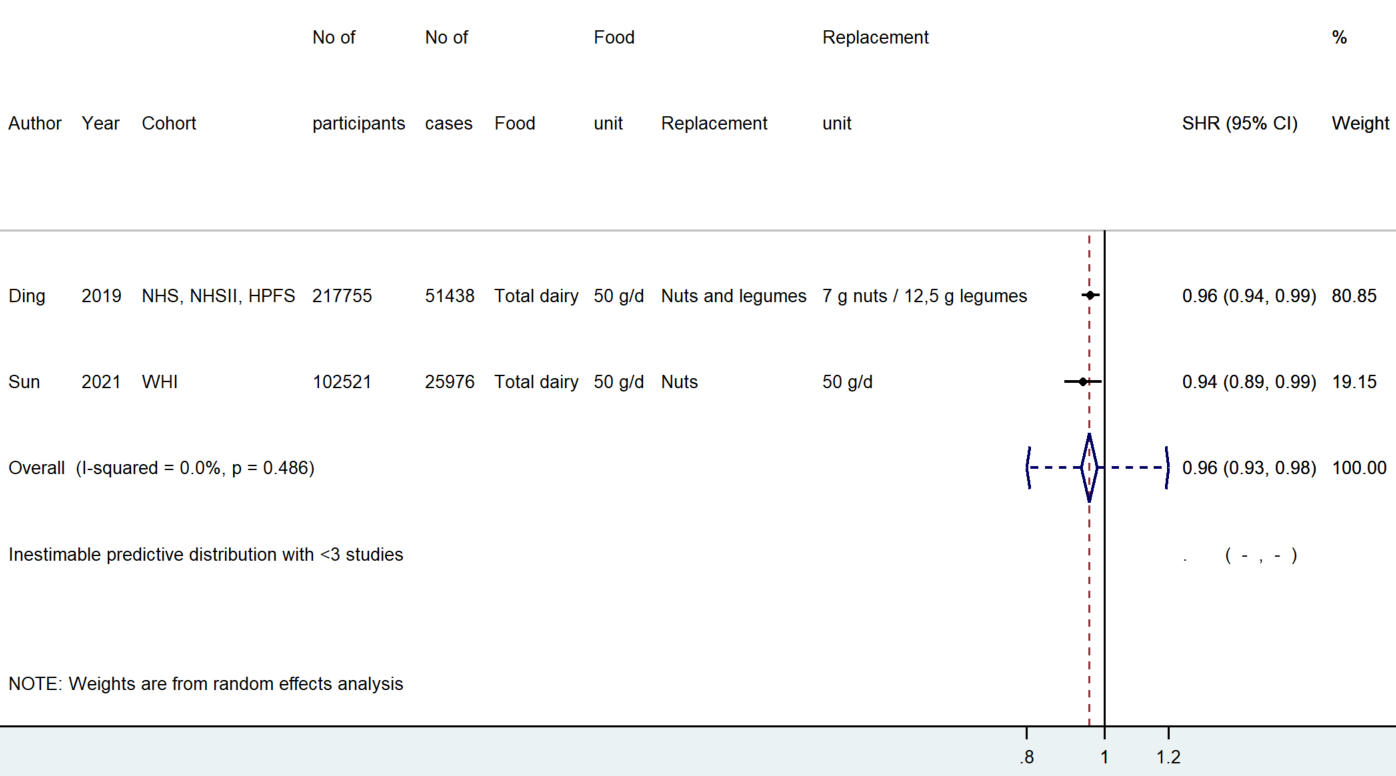  tau^2^ = 0.0000 |
| M) | 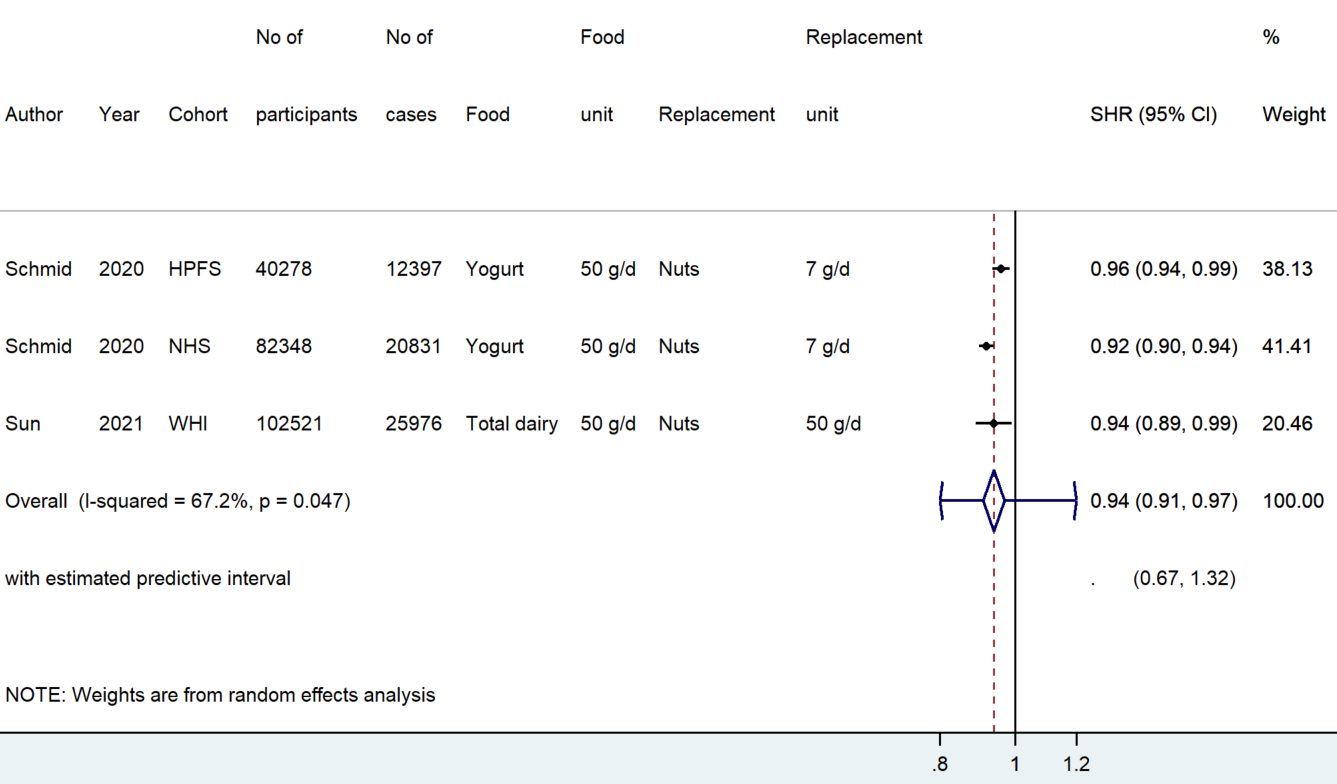  tau^2^ = 0.0005 |
| N) | 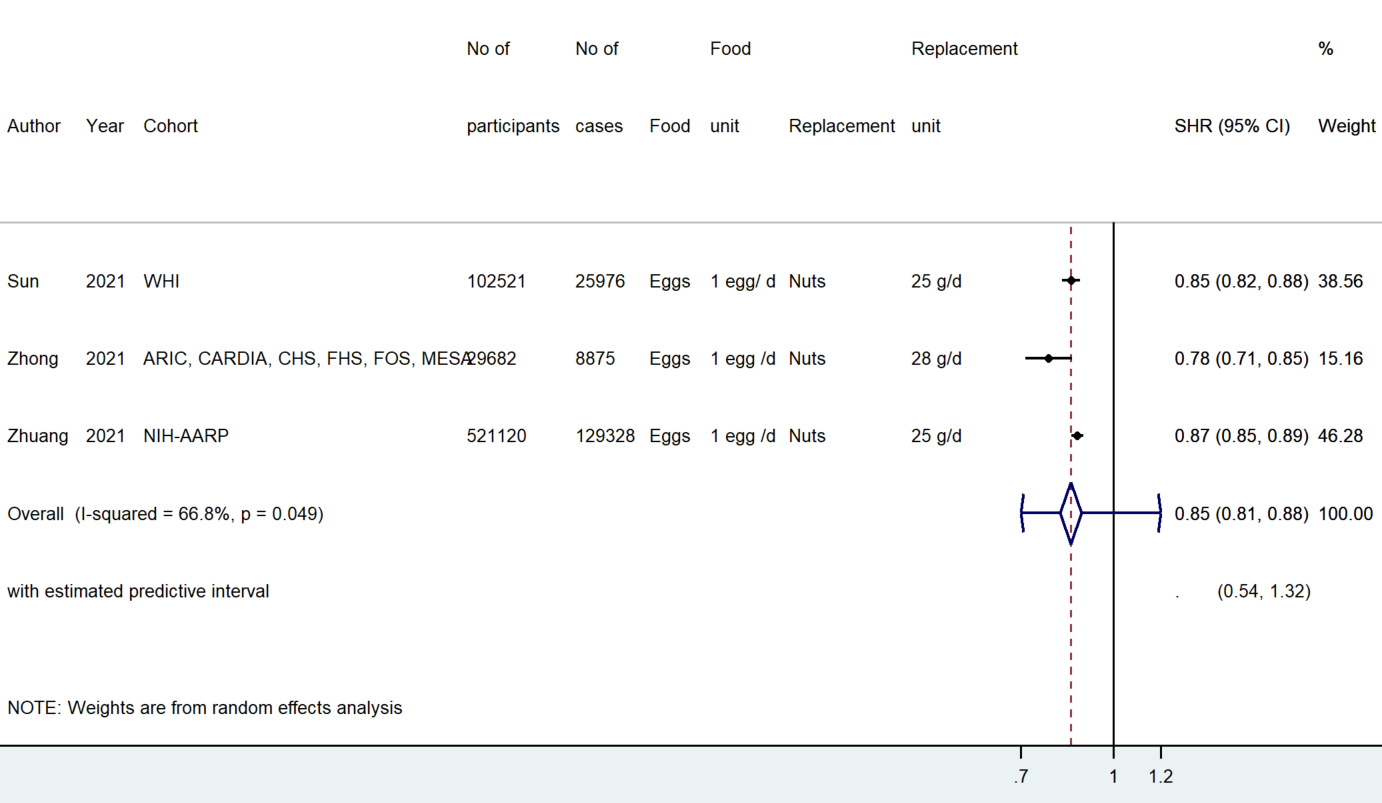  tau^2^ = 0.0008 |
| O) | 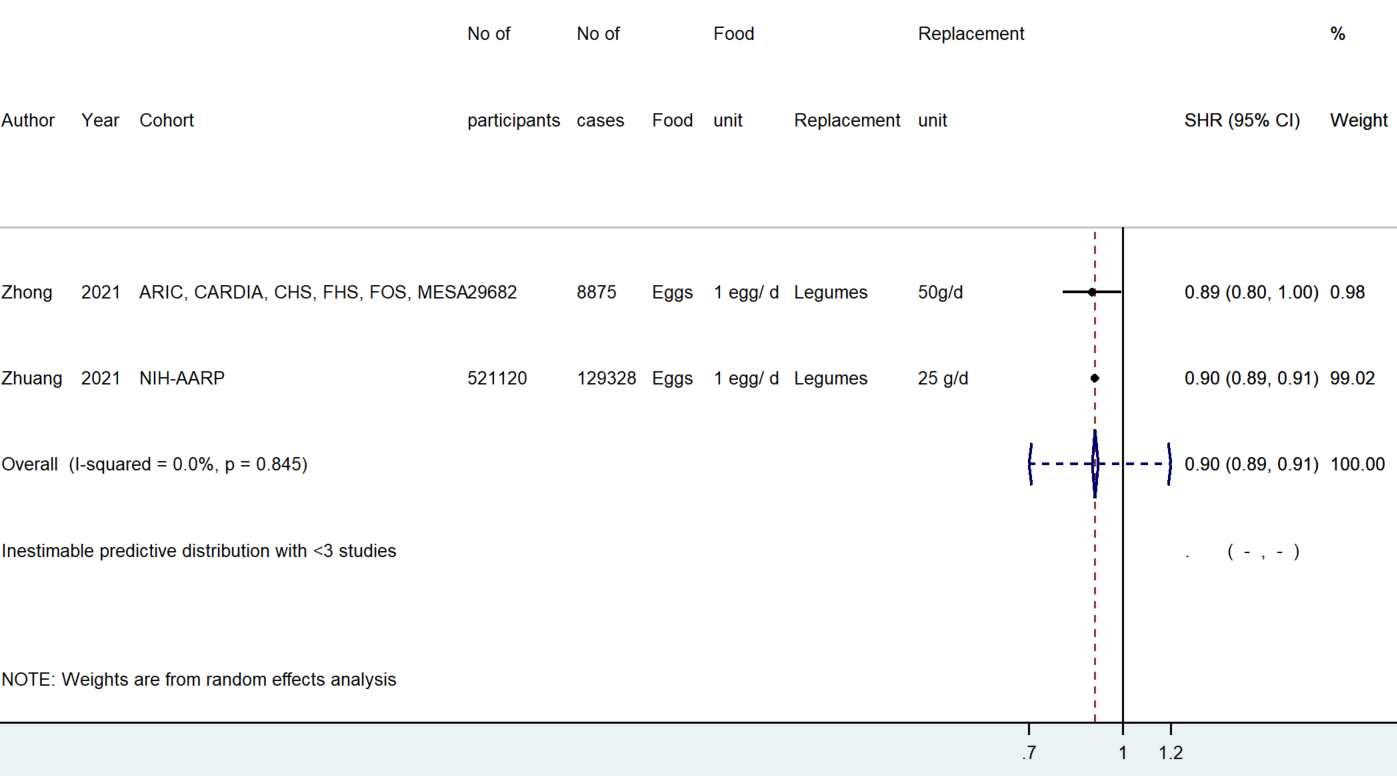  tau^2^ = 0.0000 |
| P) | 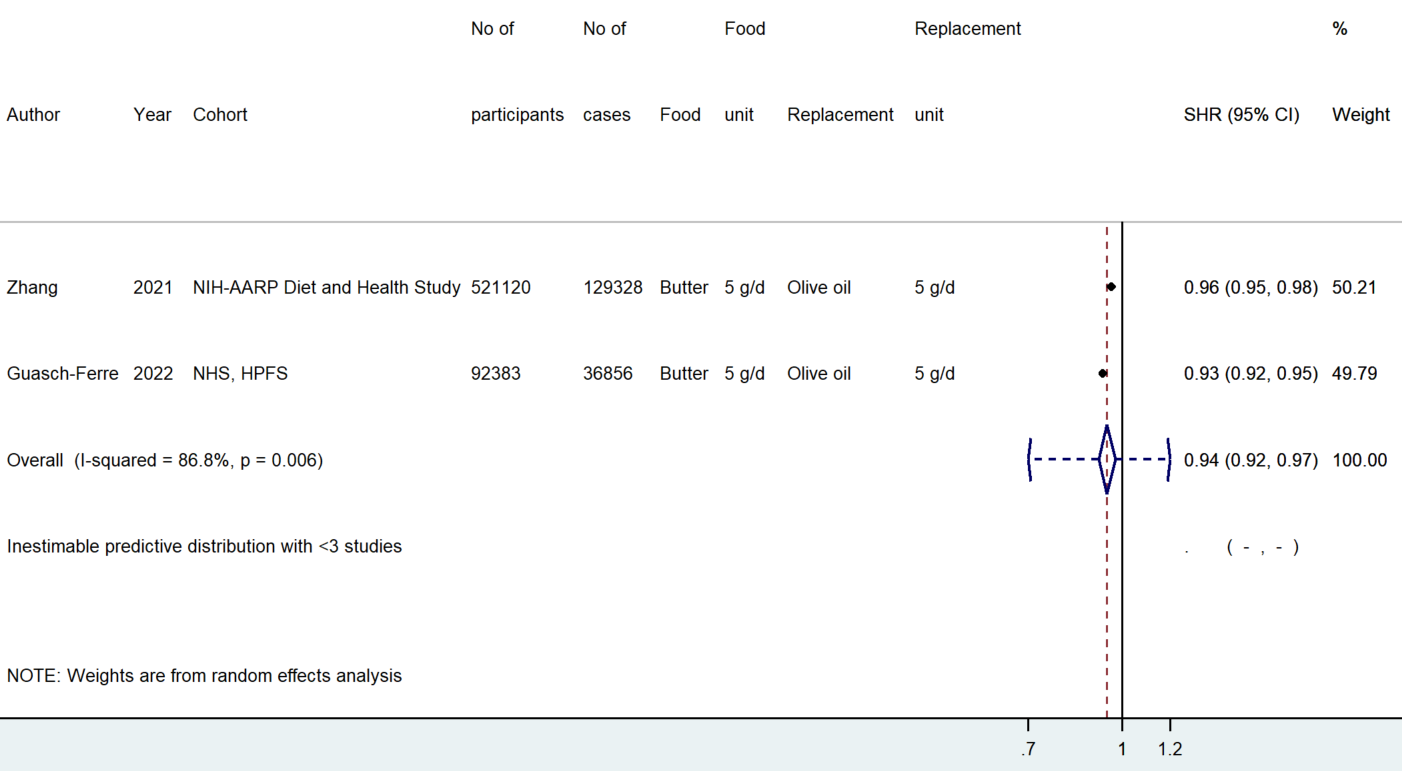  tau^2^ = 0.0004 |

**Fig. S9:** Forest plot showing the results from extracted pooled analyses regarding CVD mortality and CVD incidence for the substitution of animal-based with plant-based food


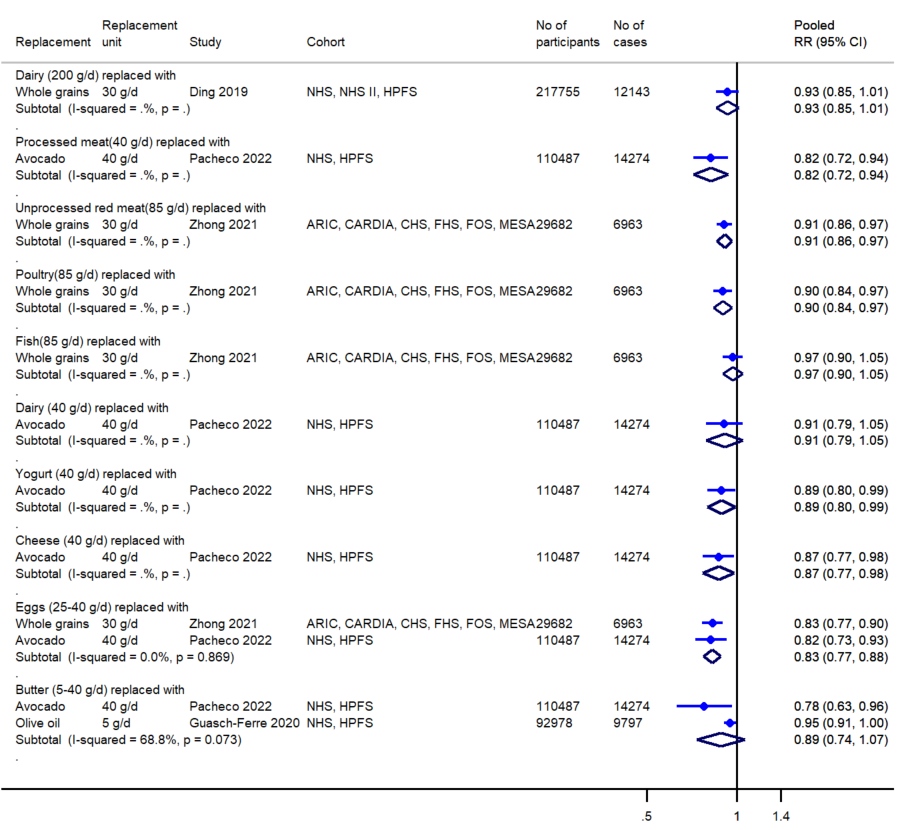

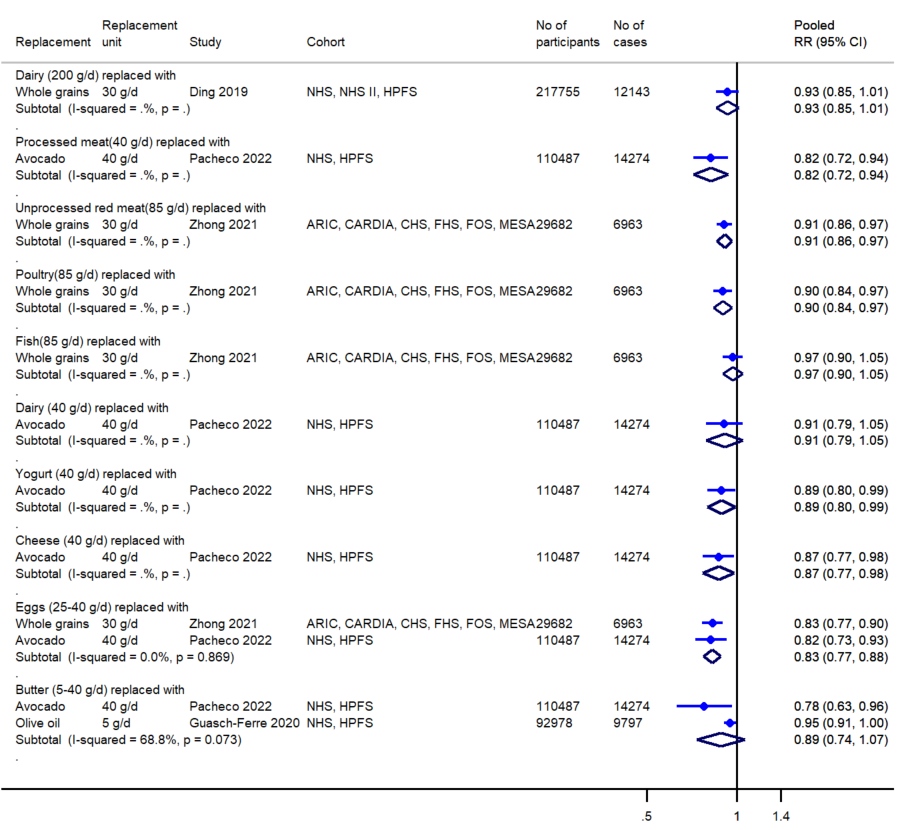

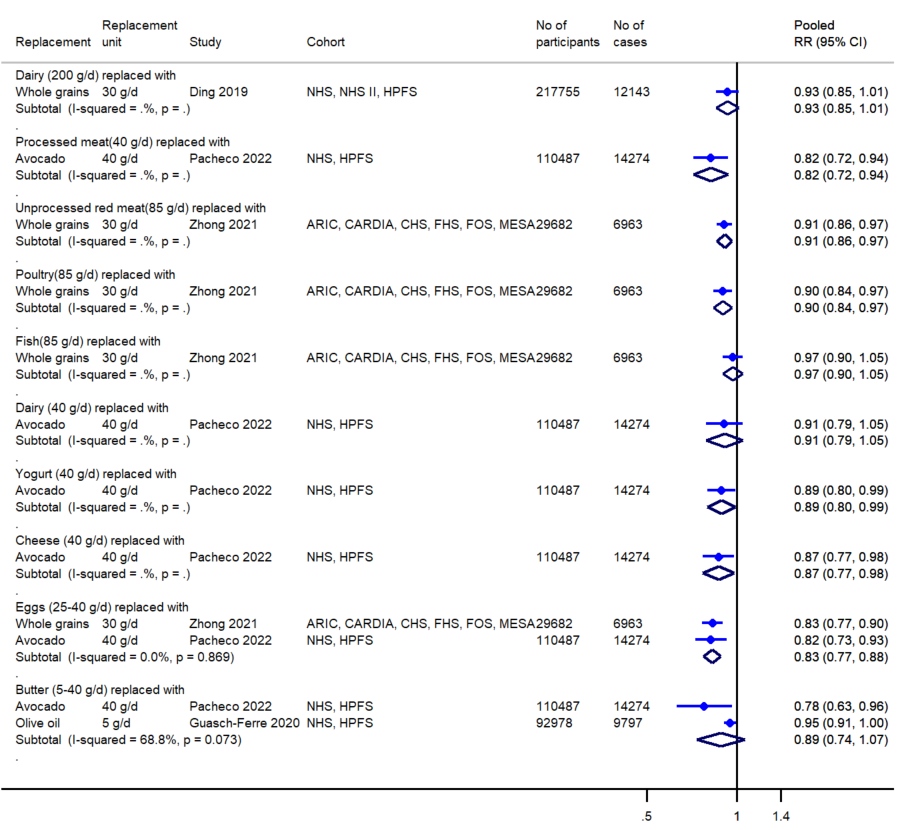

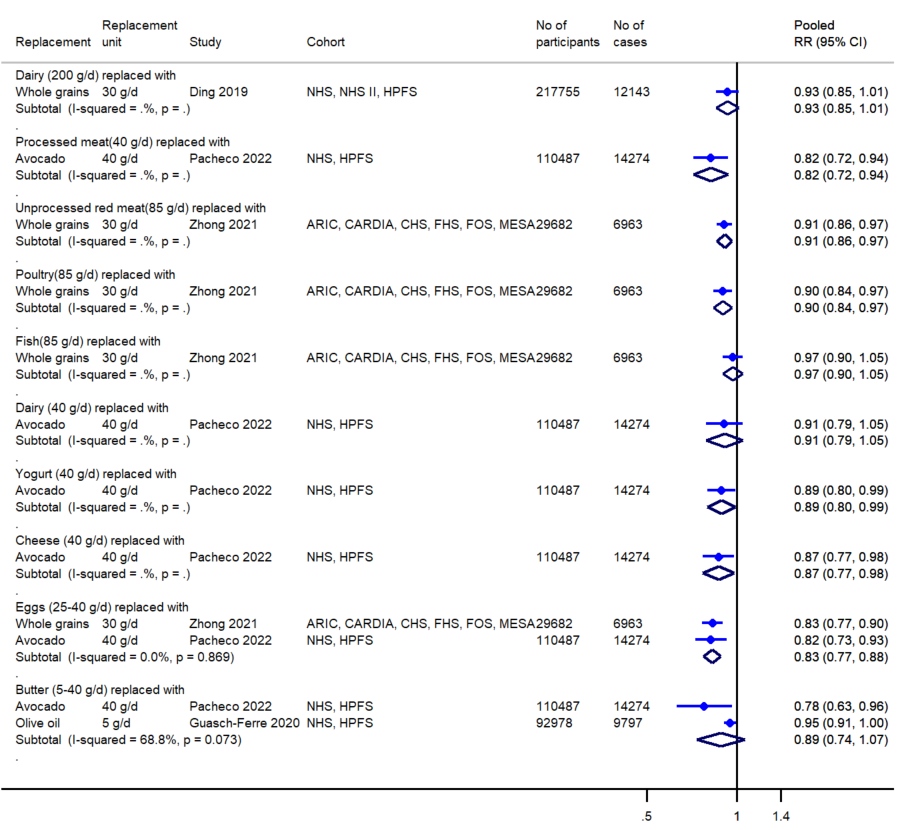

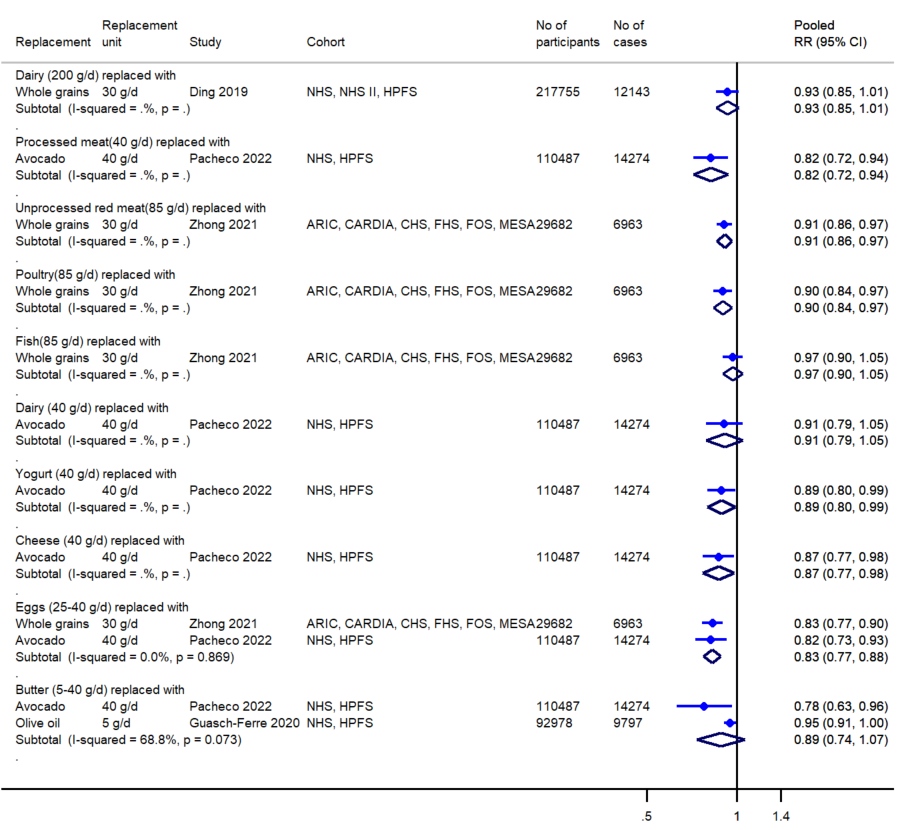

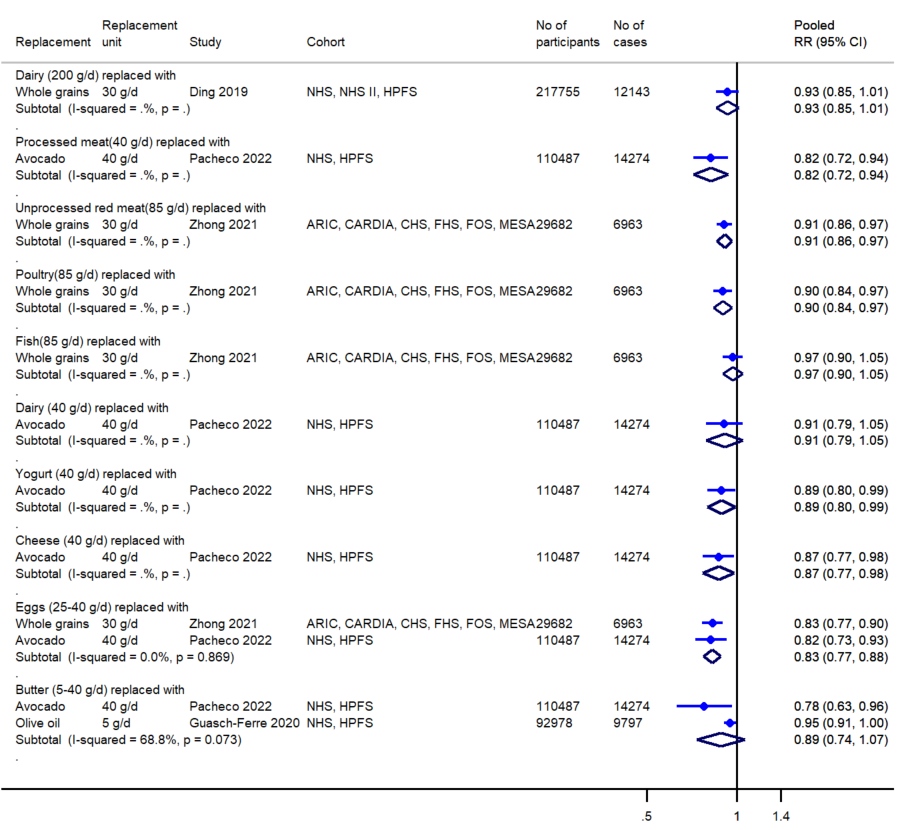

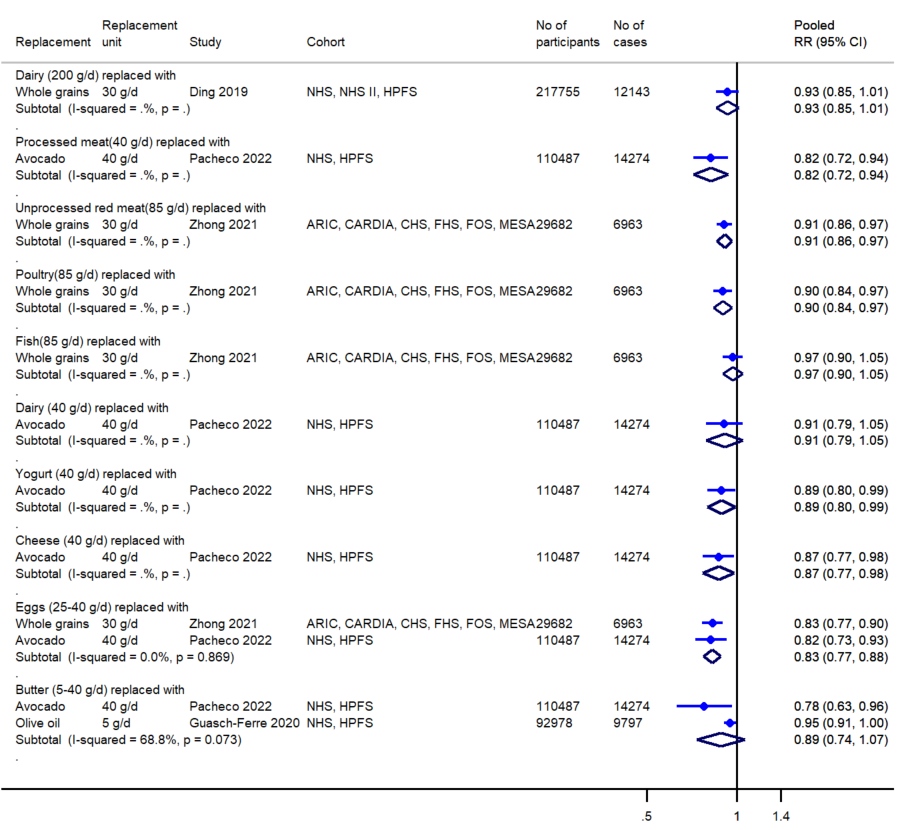

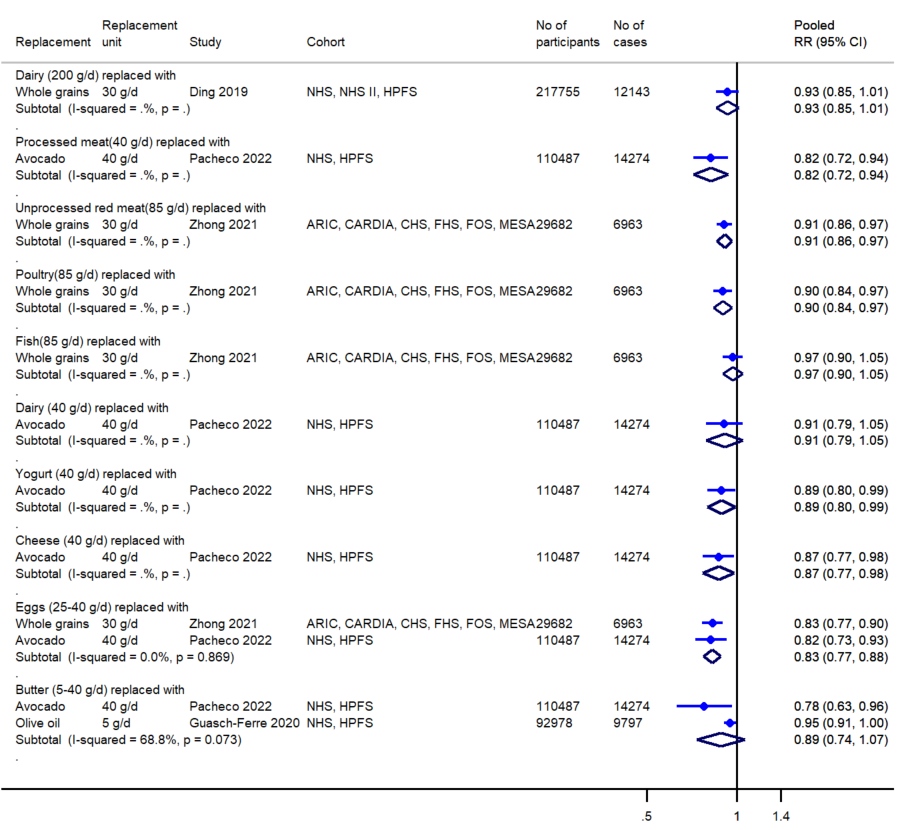

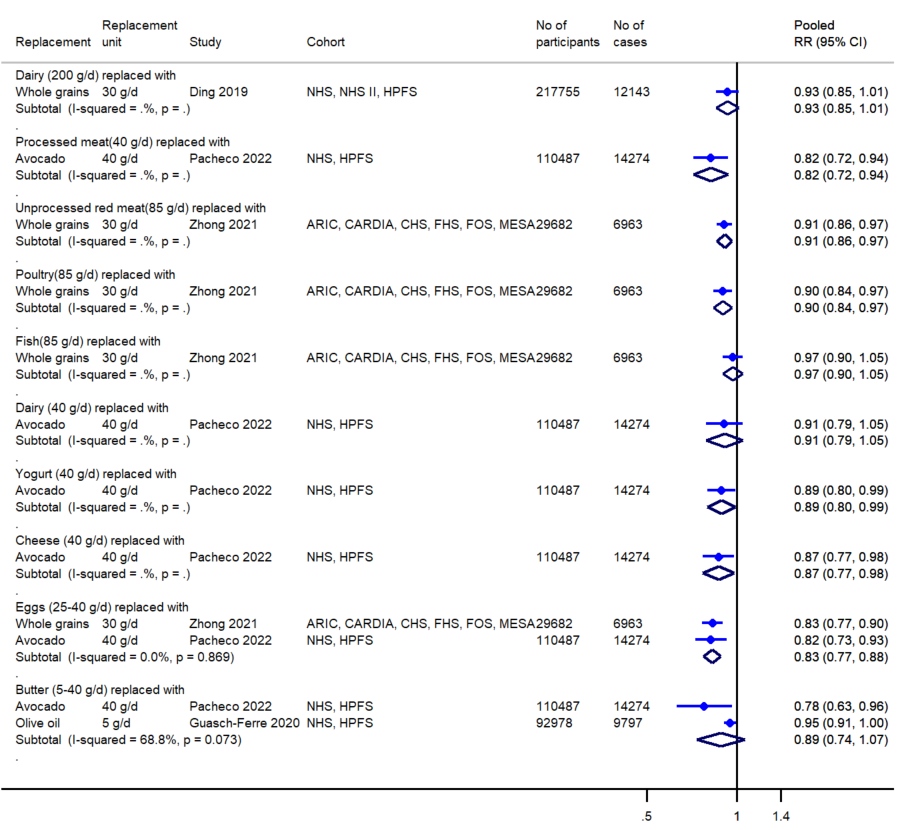

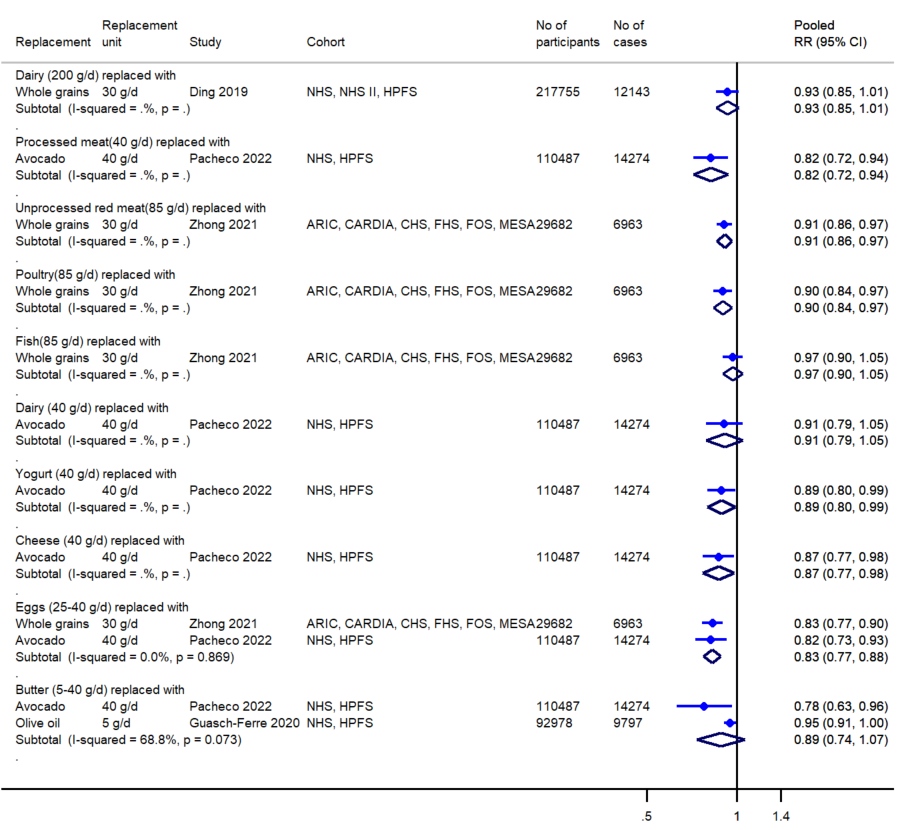

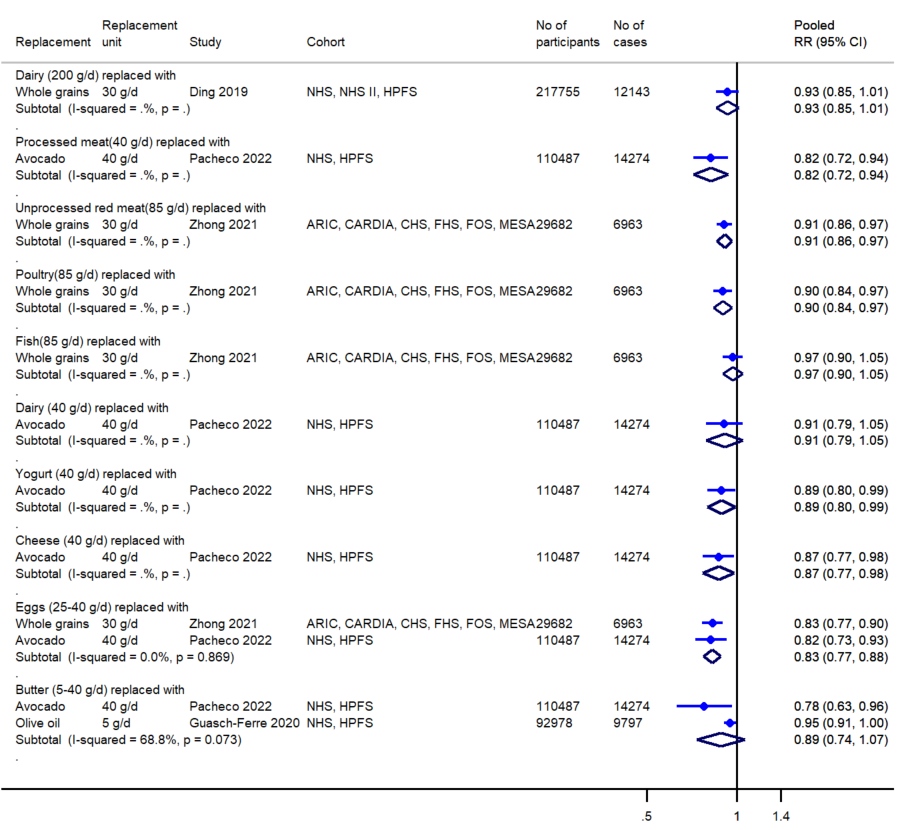

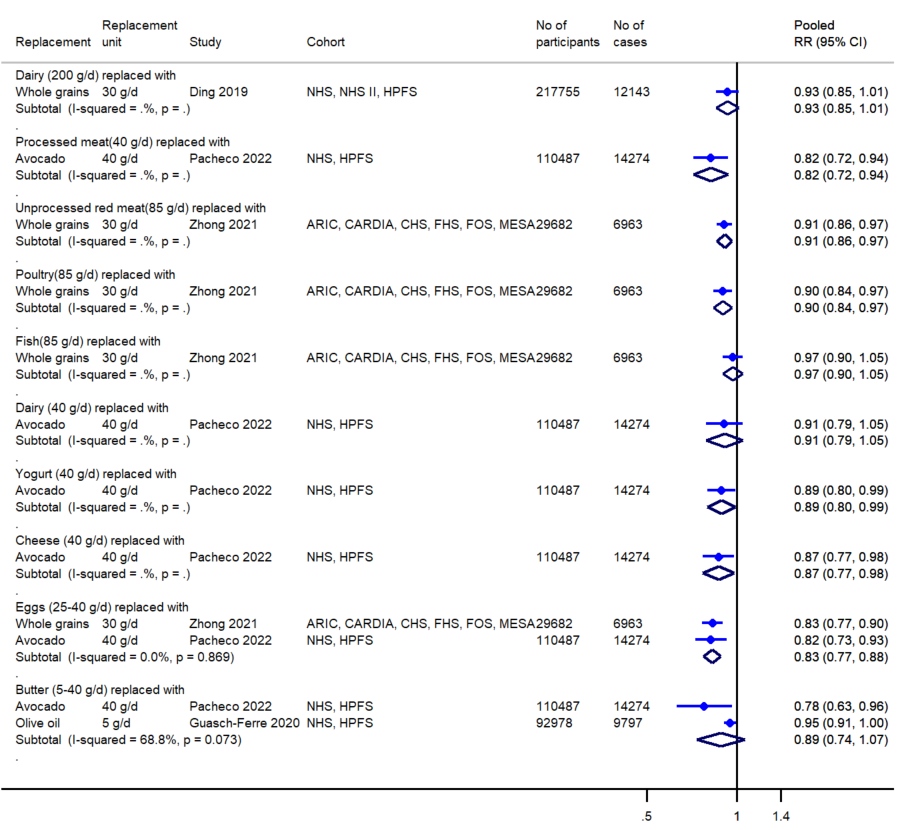


**CVD mortality**

**CVD incidence**


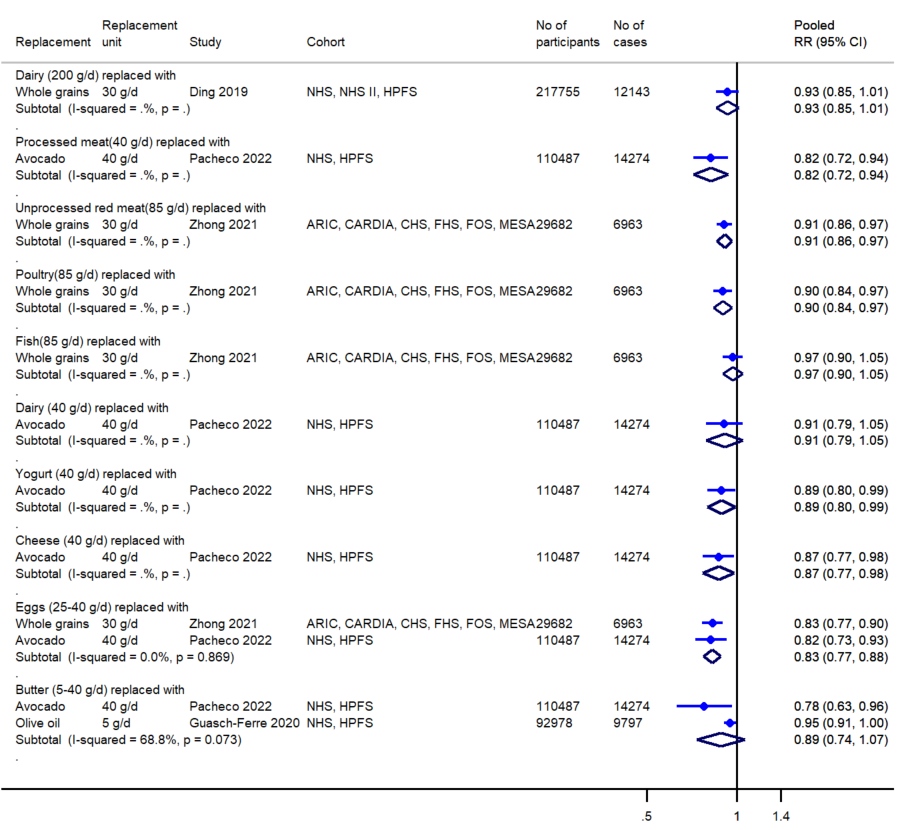

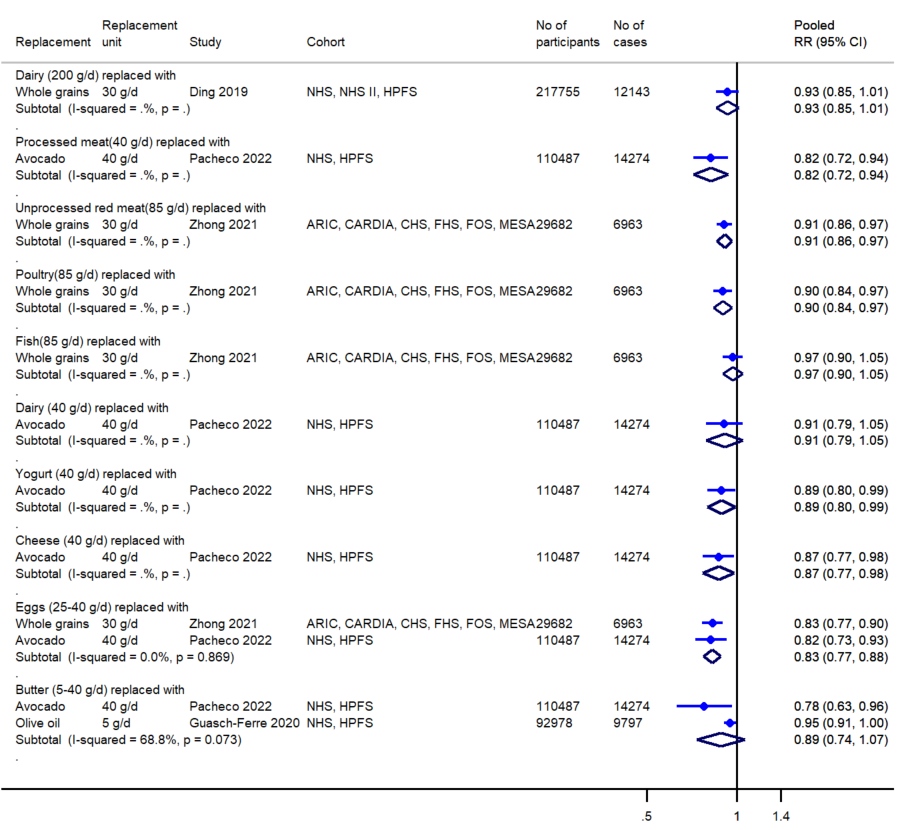


**Fig. S10:** Forest plot showing the results from extracted pooled analyses regarding CHD and stroke incidence for the substitution of animal-based with plant-based food


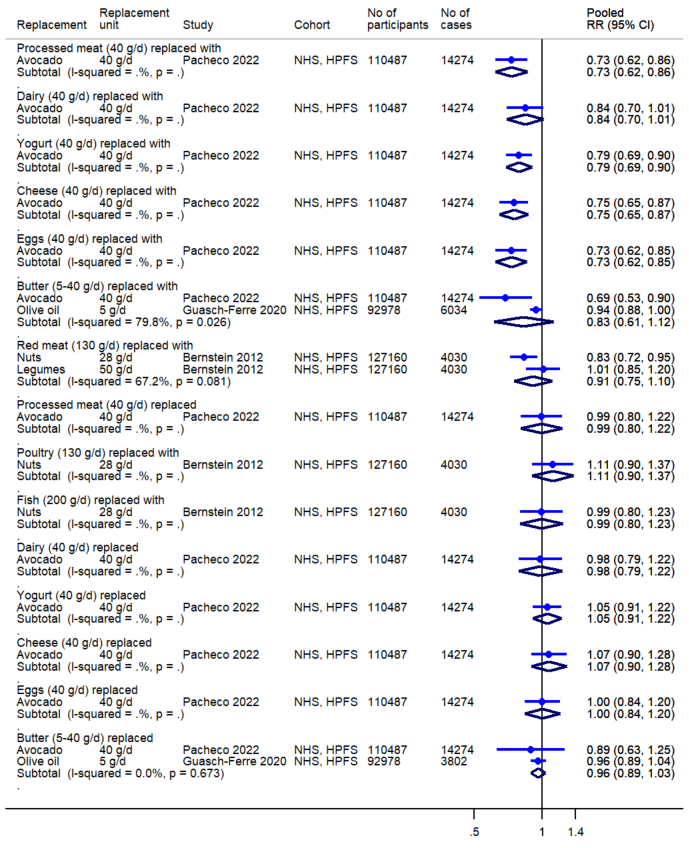

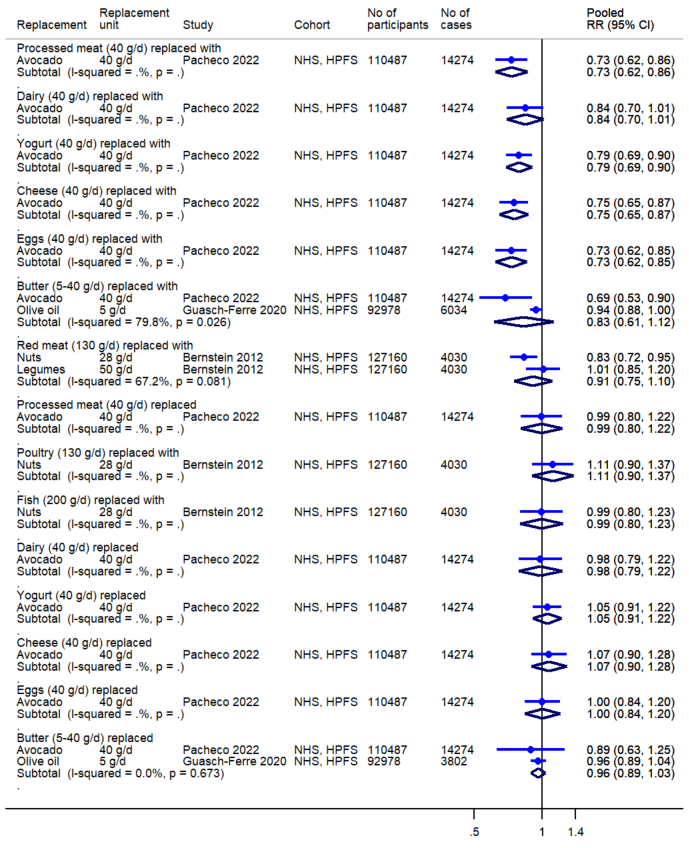

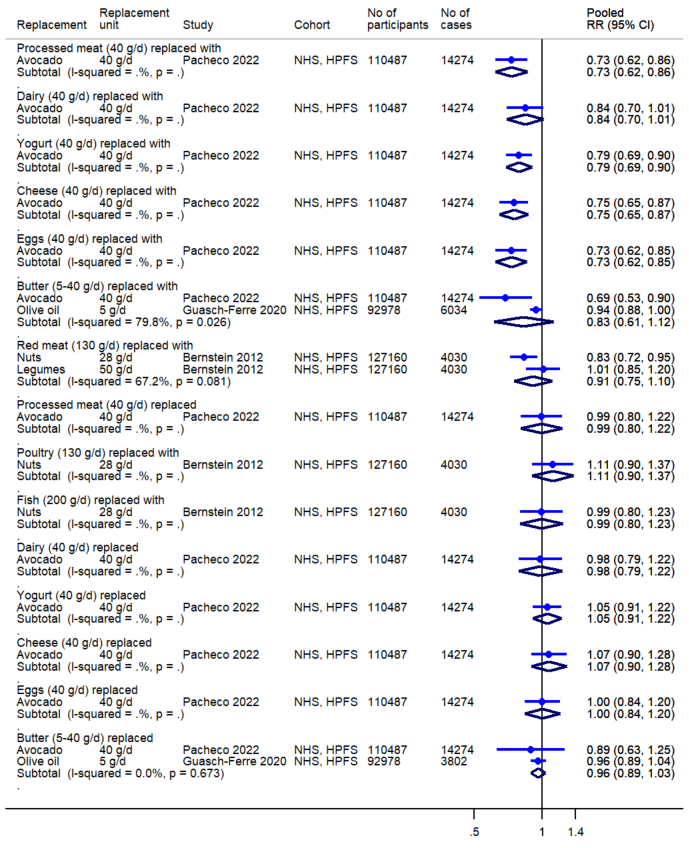

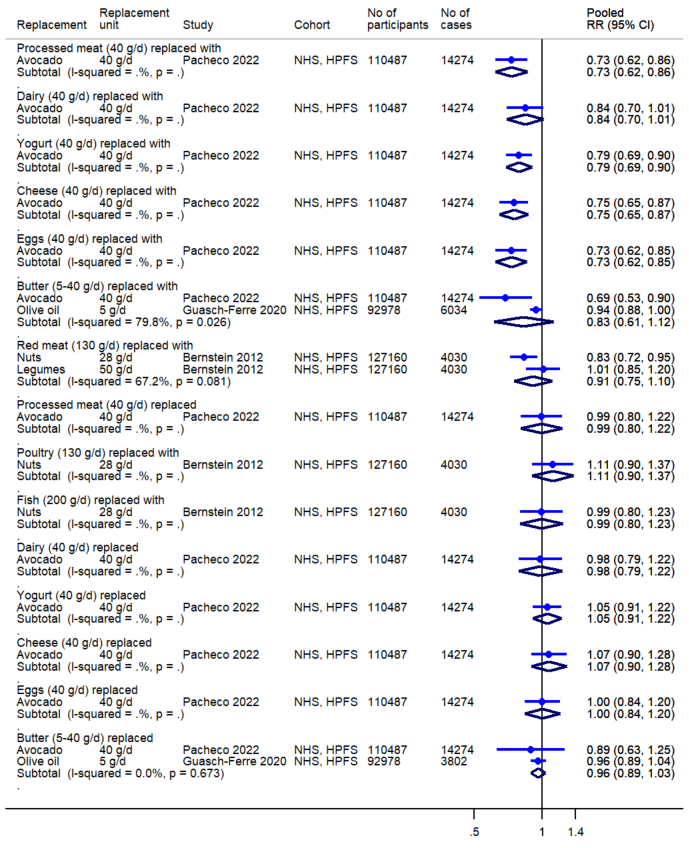

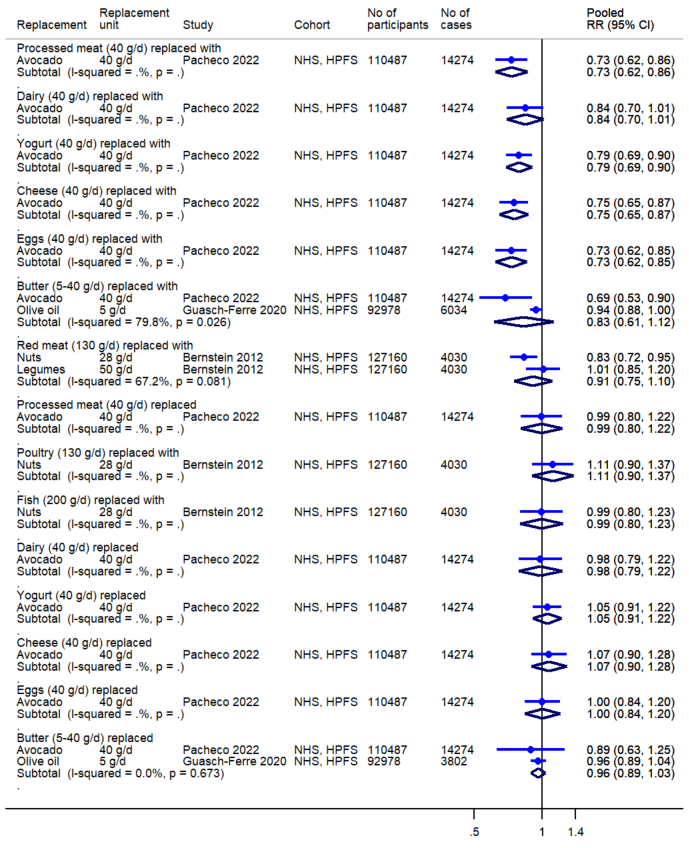

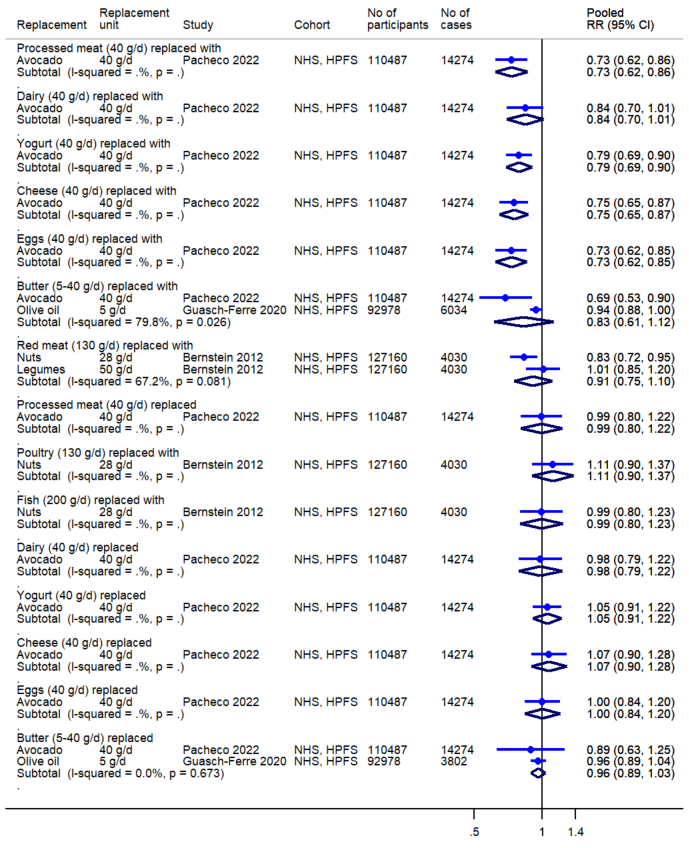

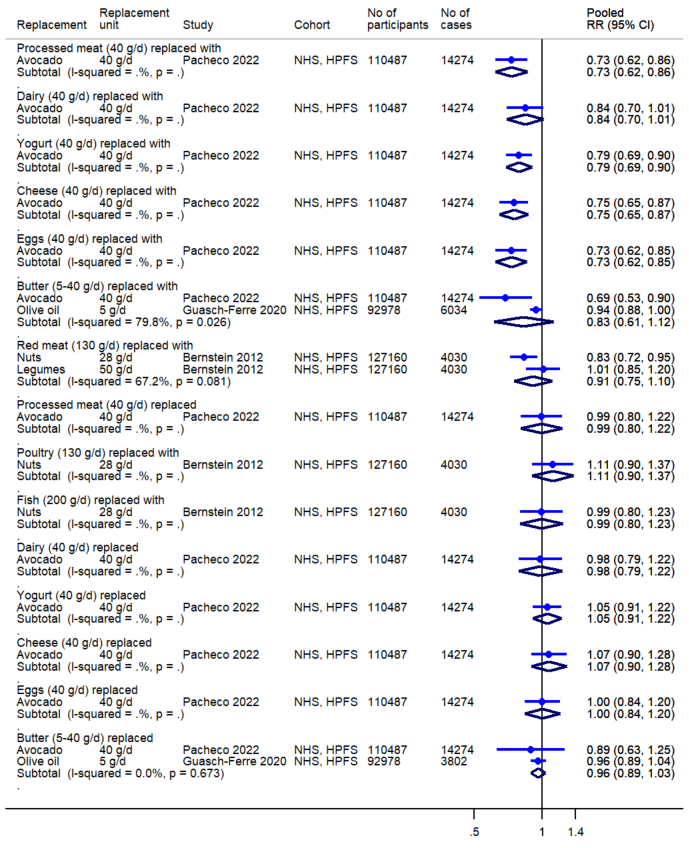

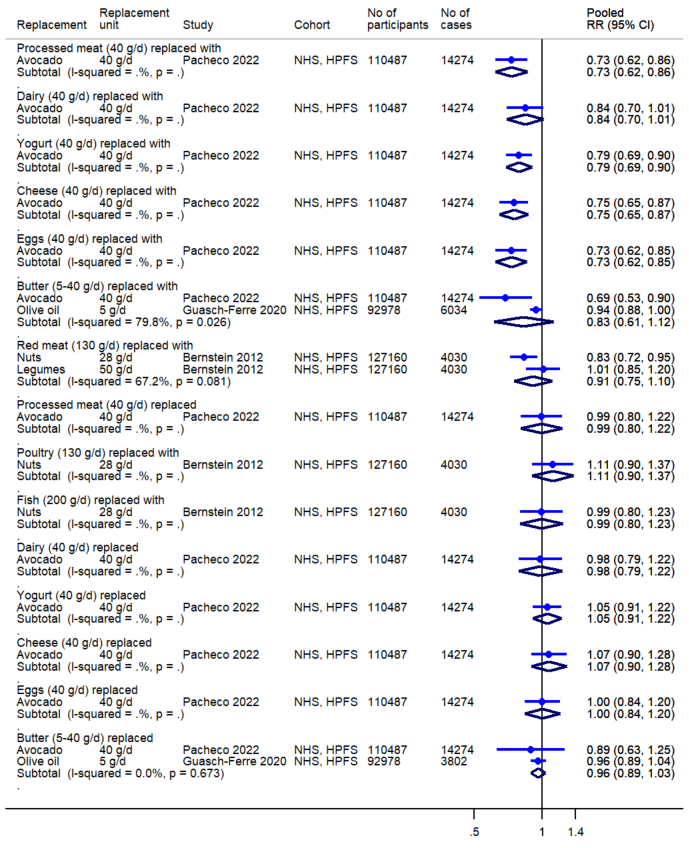

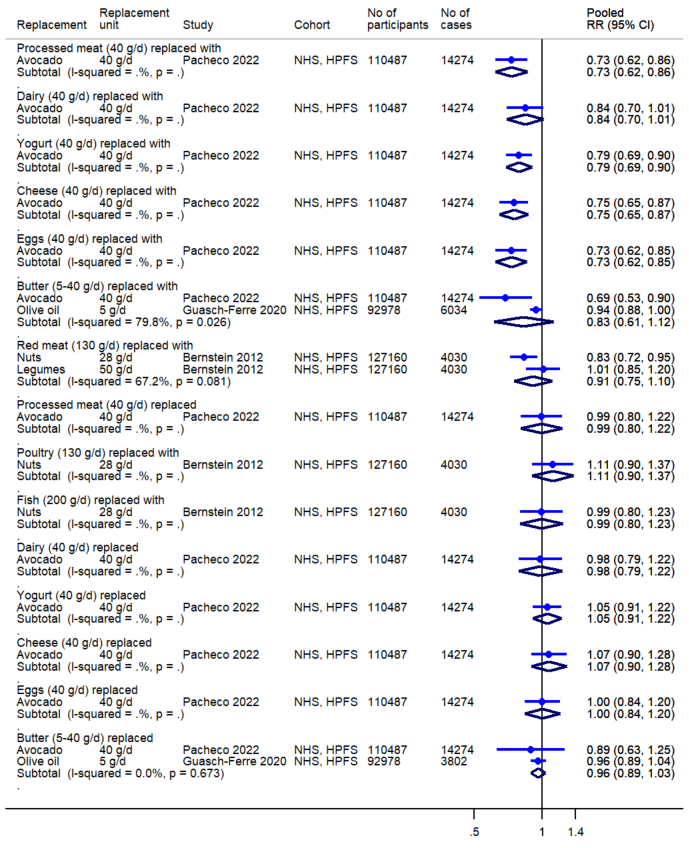

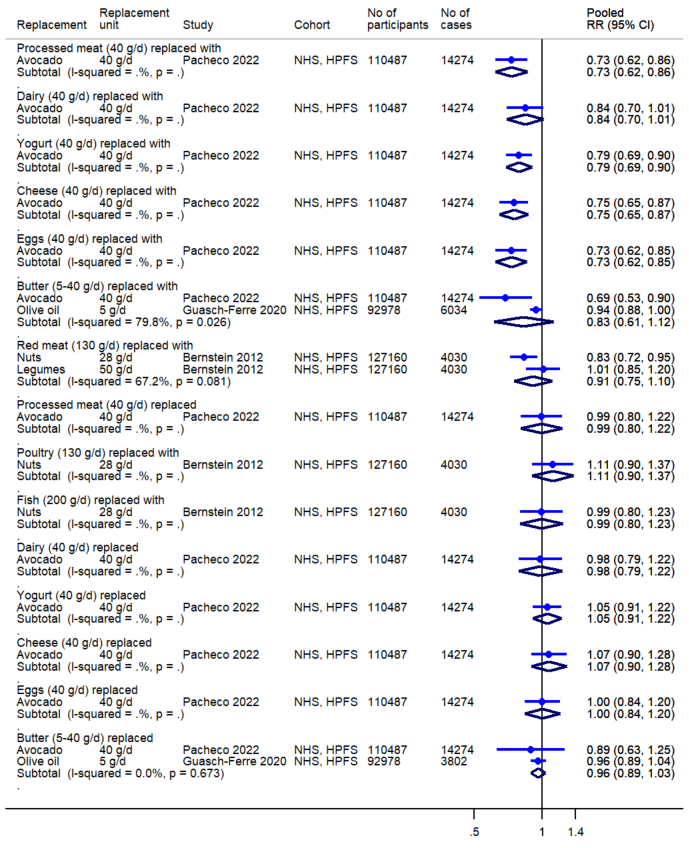

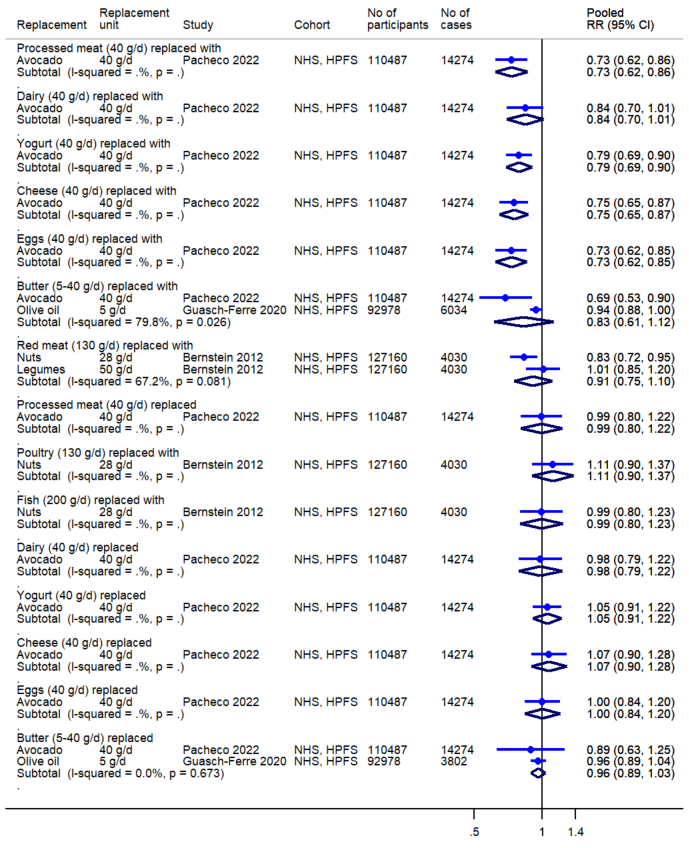

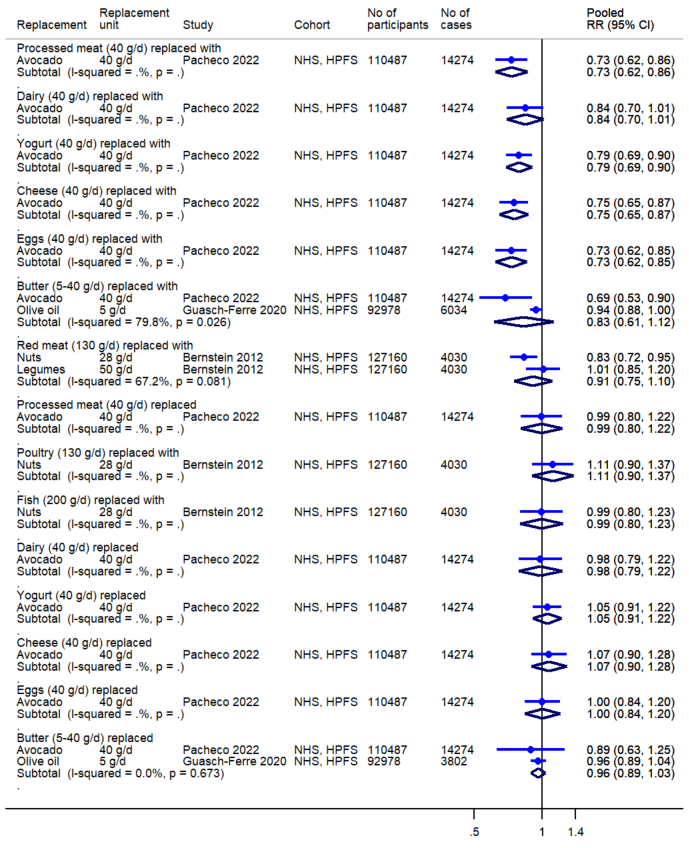

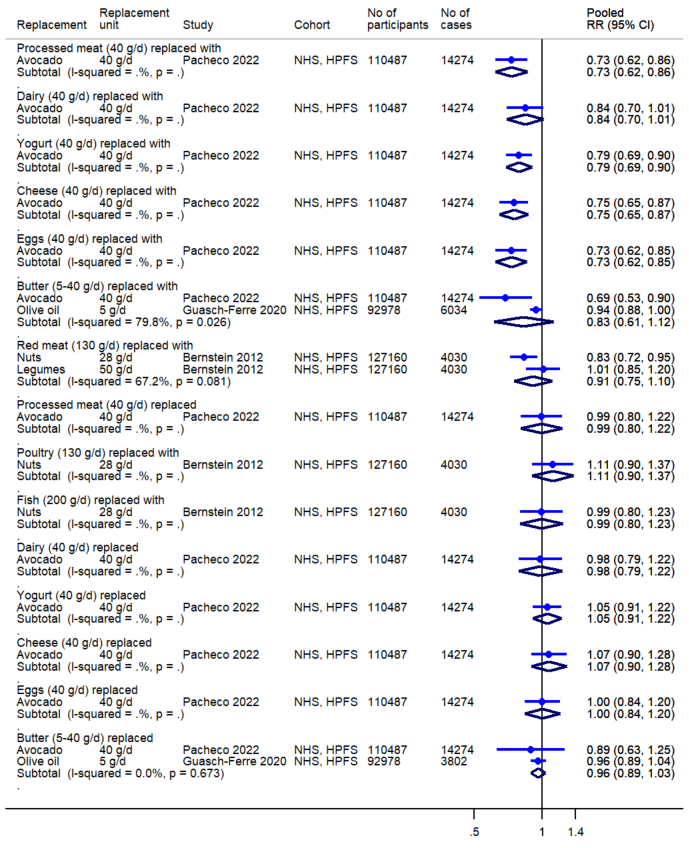

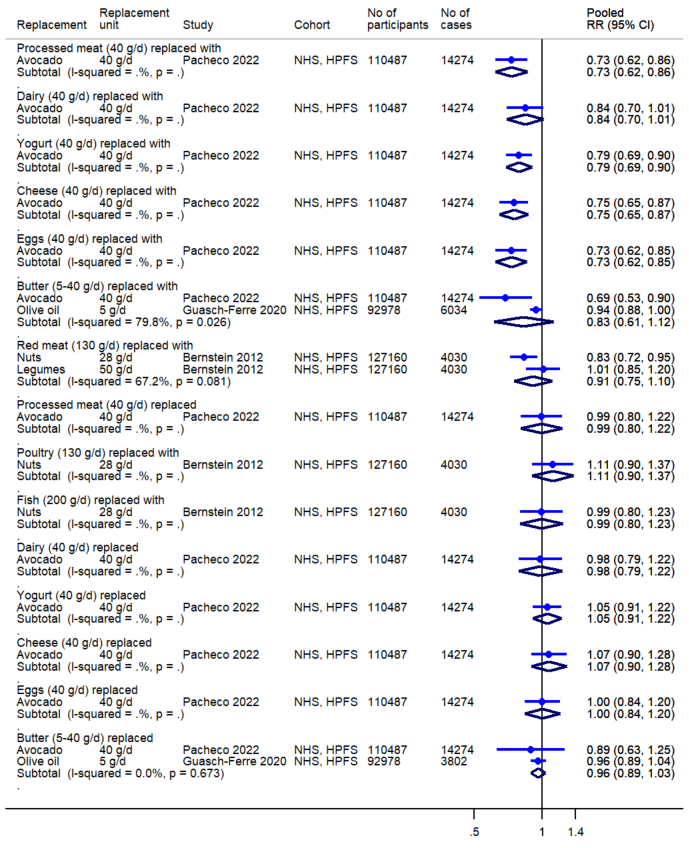

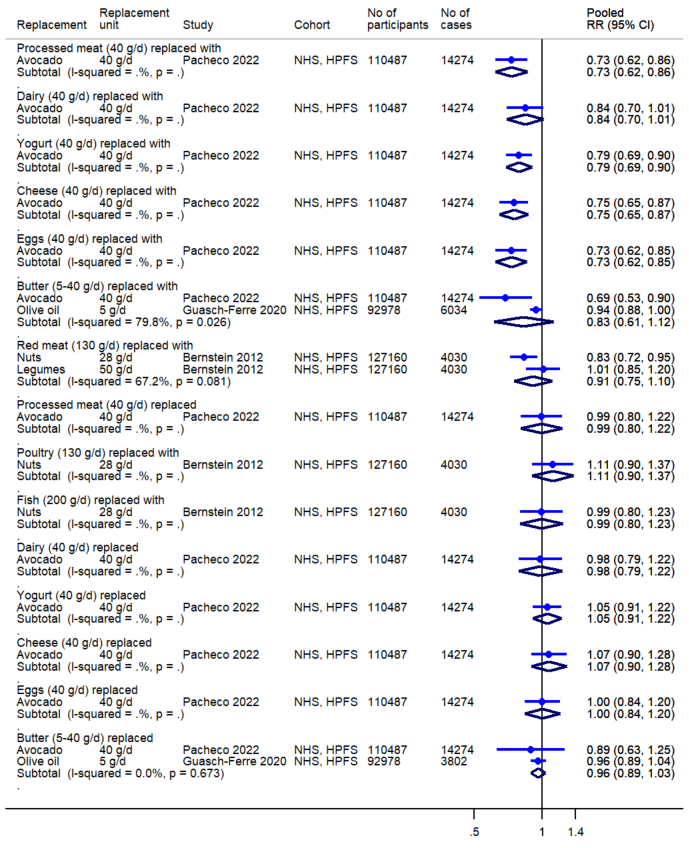

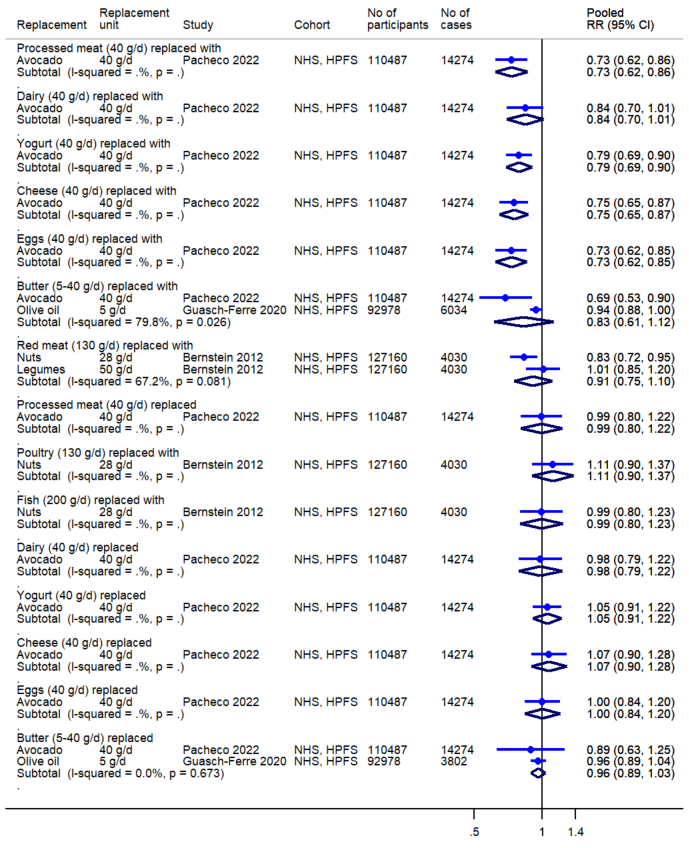

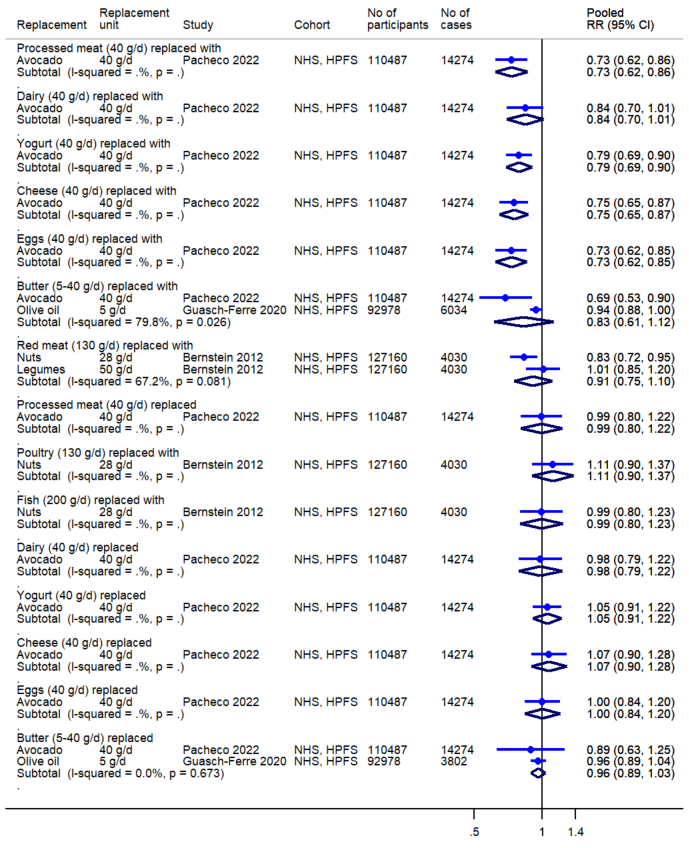


**CHD incidence**

**Stroke incidence**


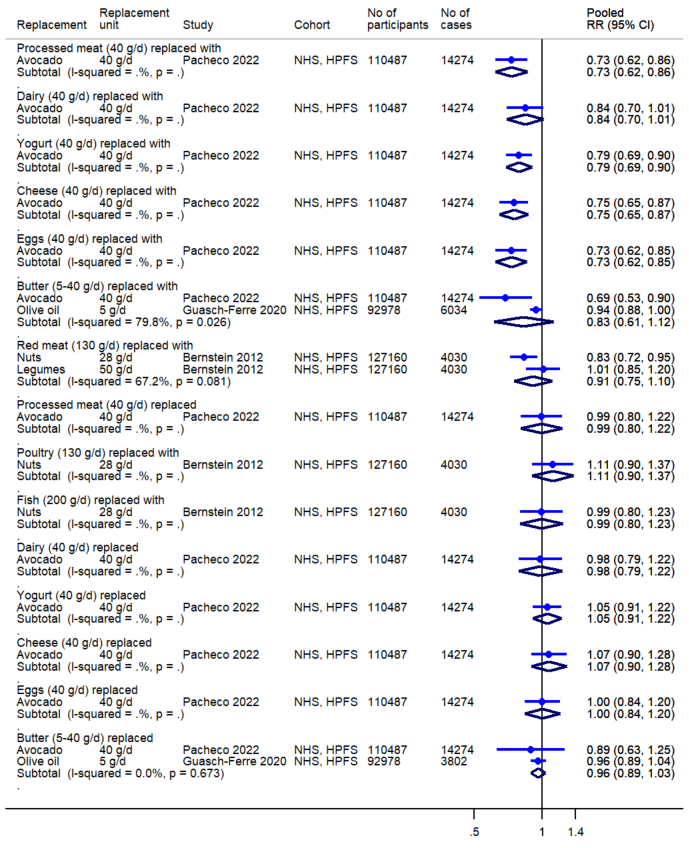

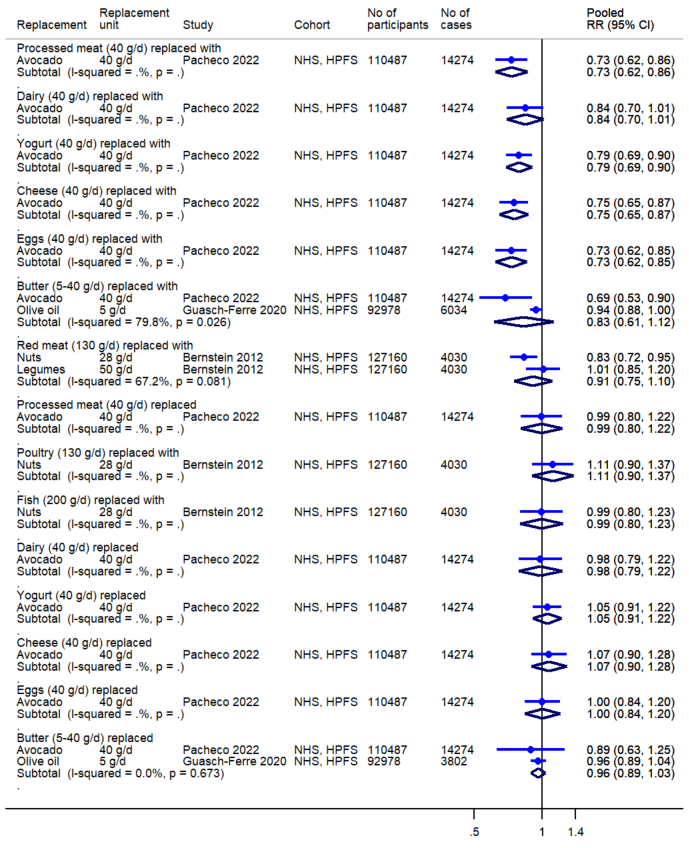

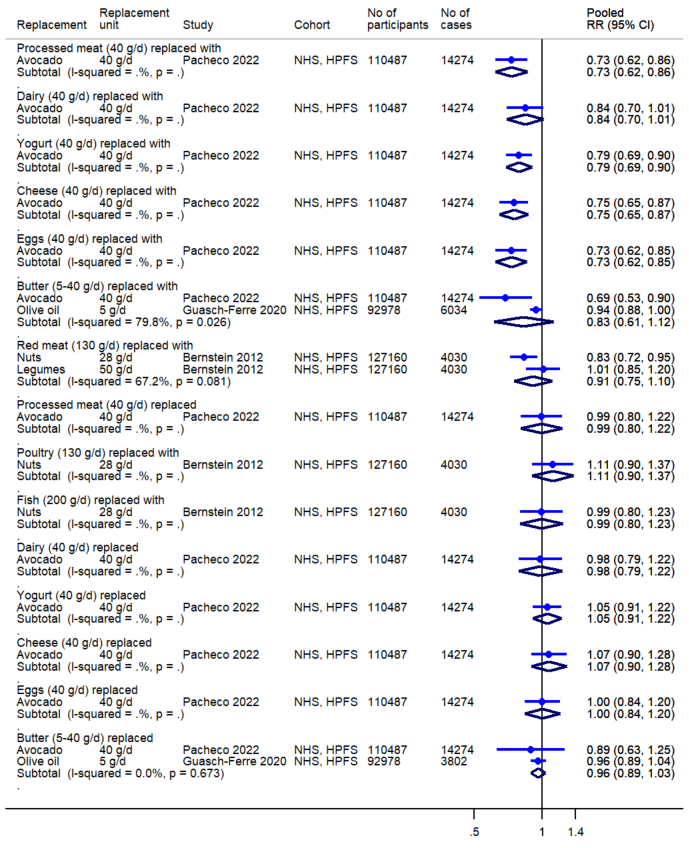

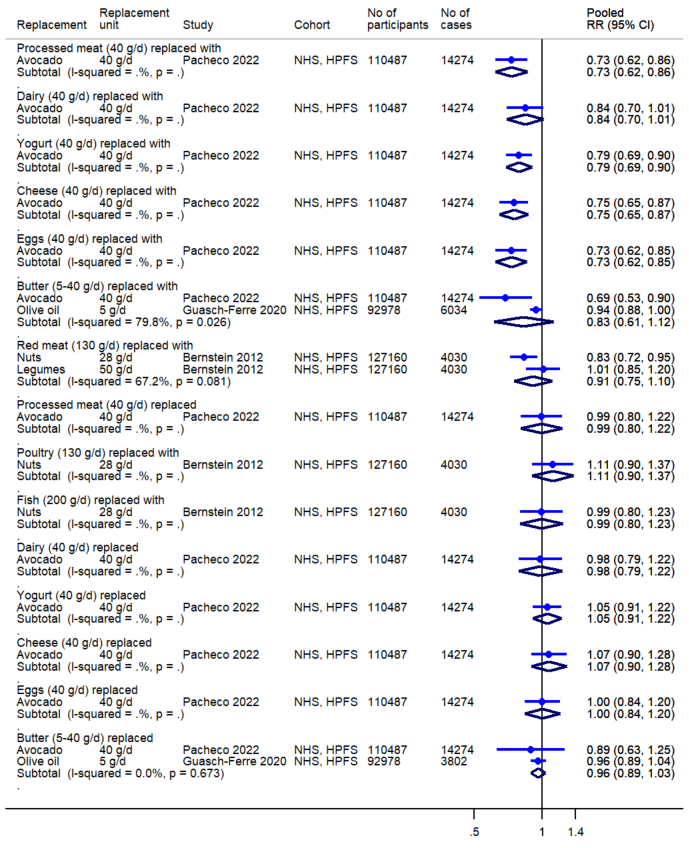


**Fig. S11:** Forest plot showing the results from extracted pooled analyses regarding diabetes for the substitution of animal-based with plant-based food

**Type 2 diabetes incidence**

**Change**

**Fig. S12:** Forest plot showing the results from extracted pooled analyses regarding all-cause mortality for the substitution of animal-based with plant-based food

**All-cause mortality**

**Change**

**Fig. S13:** Forest plot showing the results from single cohorts regarding CVD mortality and CVD incidence for the substitution of animal-based with plant-based food

**CVD mortality**

**CVD incidence**

**Fig. S14:** Forest plot showing the results from single cohorts regarding CHD, MI and stroke incidence for the substitution of animal-based with plant-based food

**CHD incidence**

**Myocardial infarction incidence**

**Stroke incidence**

**Fig. S15:** Forest plot showing the results from single cohorts regarding diabetes for the substitution of animal-based with plant-based food

**Type 2 diabetes incidence**

**Diabetes mortality**

**Change**

**Fig. S16:** Forest plot showing the results from single cohorts regarding all-cause mortality for the substitution of animal-based with plant-based food

**All-cause mortality**
